# Supplementary figures and images for: BICD2 promotes ciliogenesis by facilitating CP110 removal from the mother centriole (part 1 of 2)
Source: EMBO Rep. 2025 Oct 16;26(22):5567–88. doi: 10.1038/s44319-025-00597-0 (PMC12635215; doi:10.1038/s44319-025-00597-0)

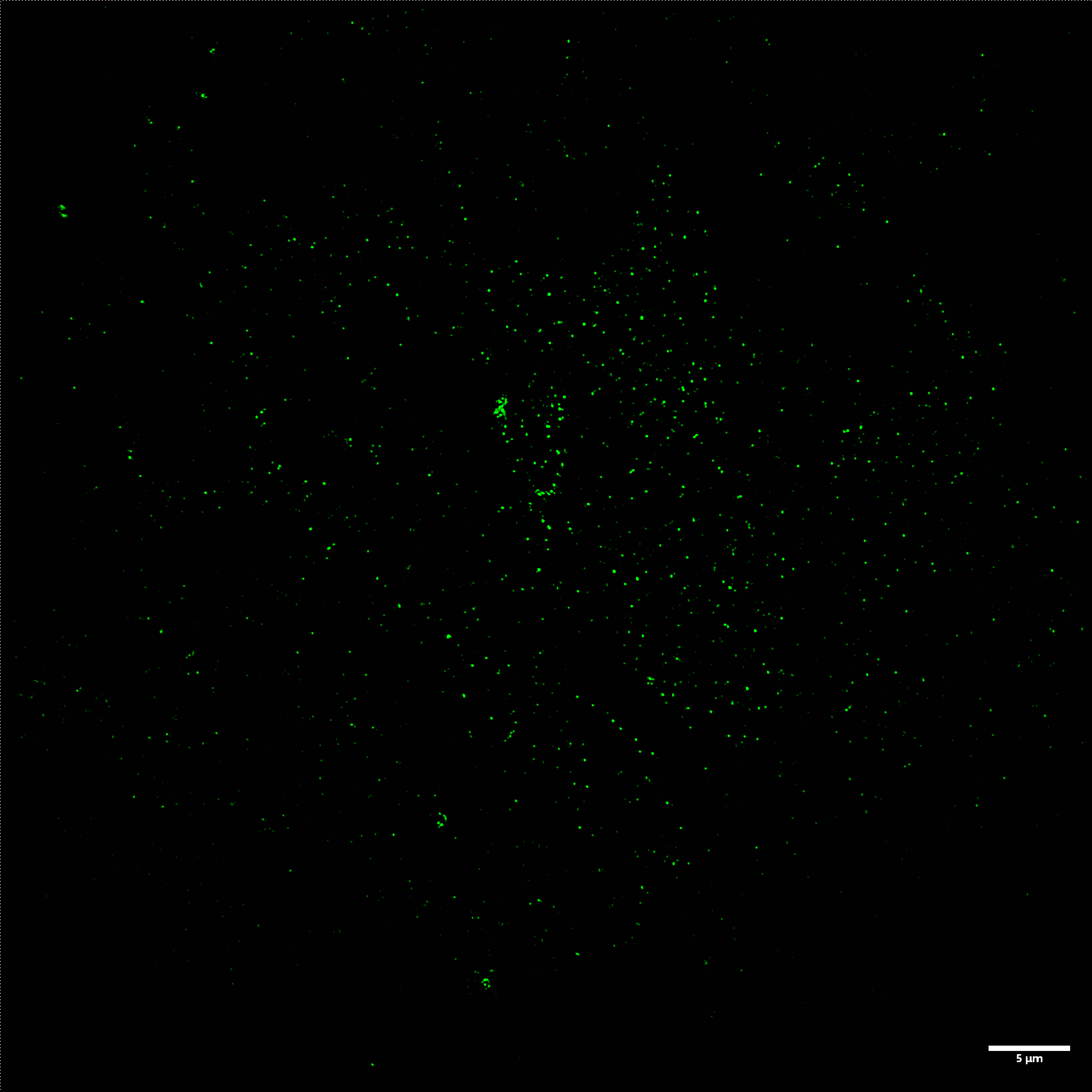

Supplement: Supplementary file 2 — Source data Fig. 1 [file 44319_2025_597_MOESM2_ESM.zip › Figure 1/1A/BICD2+C-Nap1/BICD2.bmp]

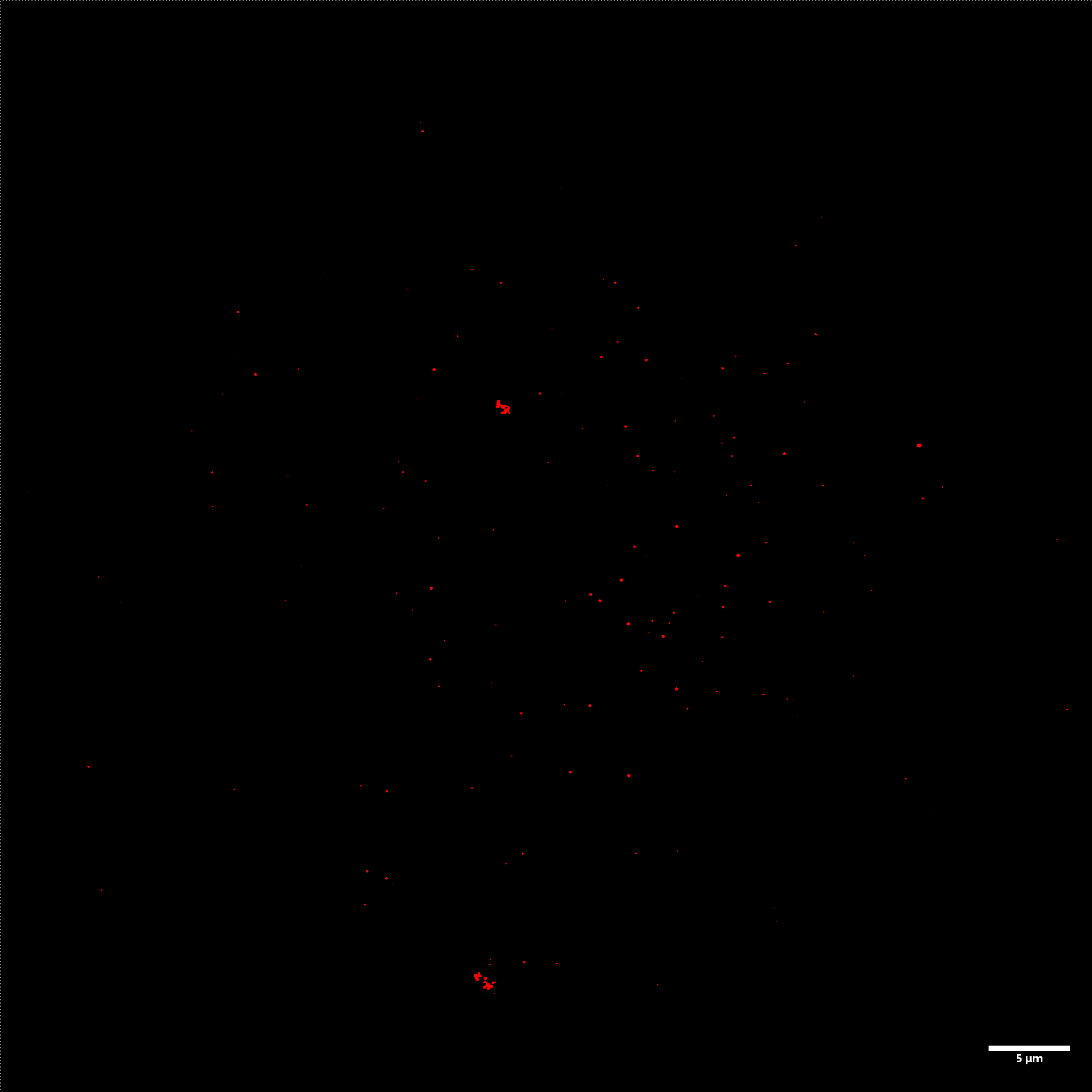

Supplement: Supplementary file 2 — Source data Fig. 1 [file 44319_2025_597_MOESM2_ESM.zip › Figure 1/1A/BICD2+C-Nap1/C-Nap1.bmp]

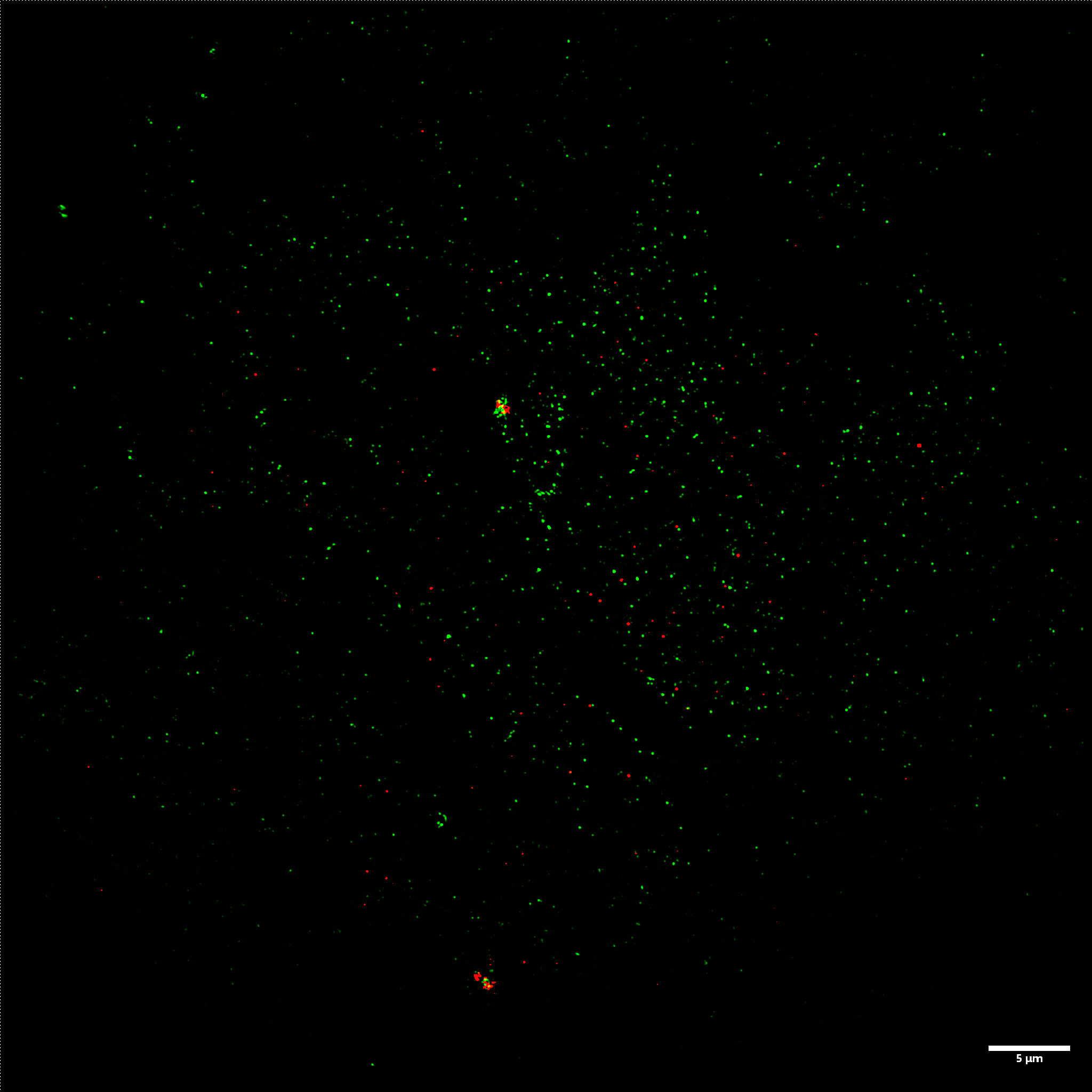

Supplement: Supplementary file 2 — Source data Fig. 1 [file 44319_2025_597_MOESM2_ESM.zip › Figure 1/1A/BICD2+C-Nap1/ICD2+C-Nap1.bmp]

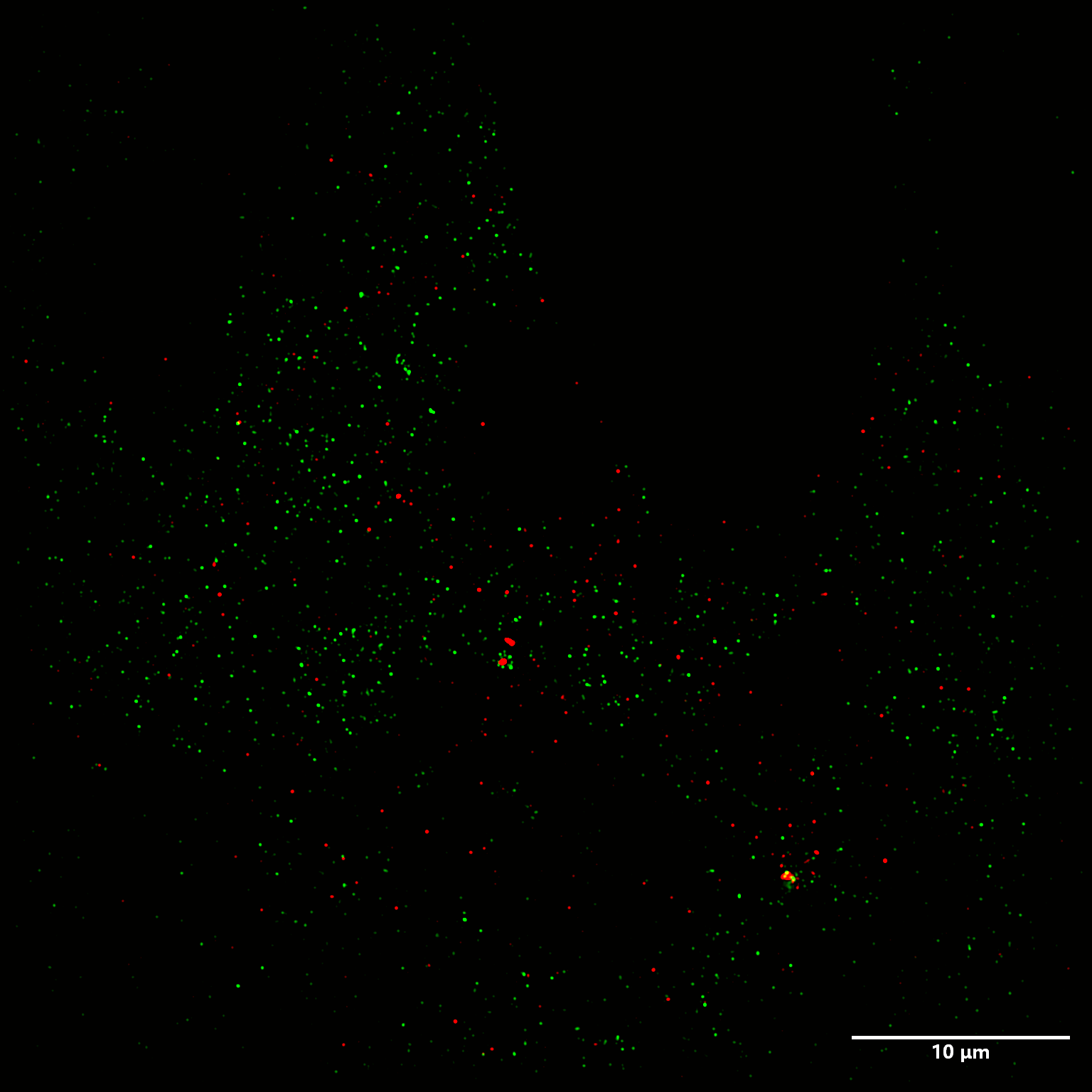

Supplement: Supplementary file 2 — Source data Fig. 1 [file 44319_2025_597_MOESM2_ESM.zip › Figure 1/1A/BICD2+Centrin/BICD2+Centrin.bmp]

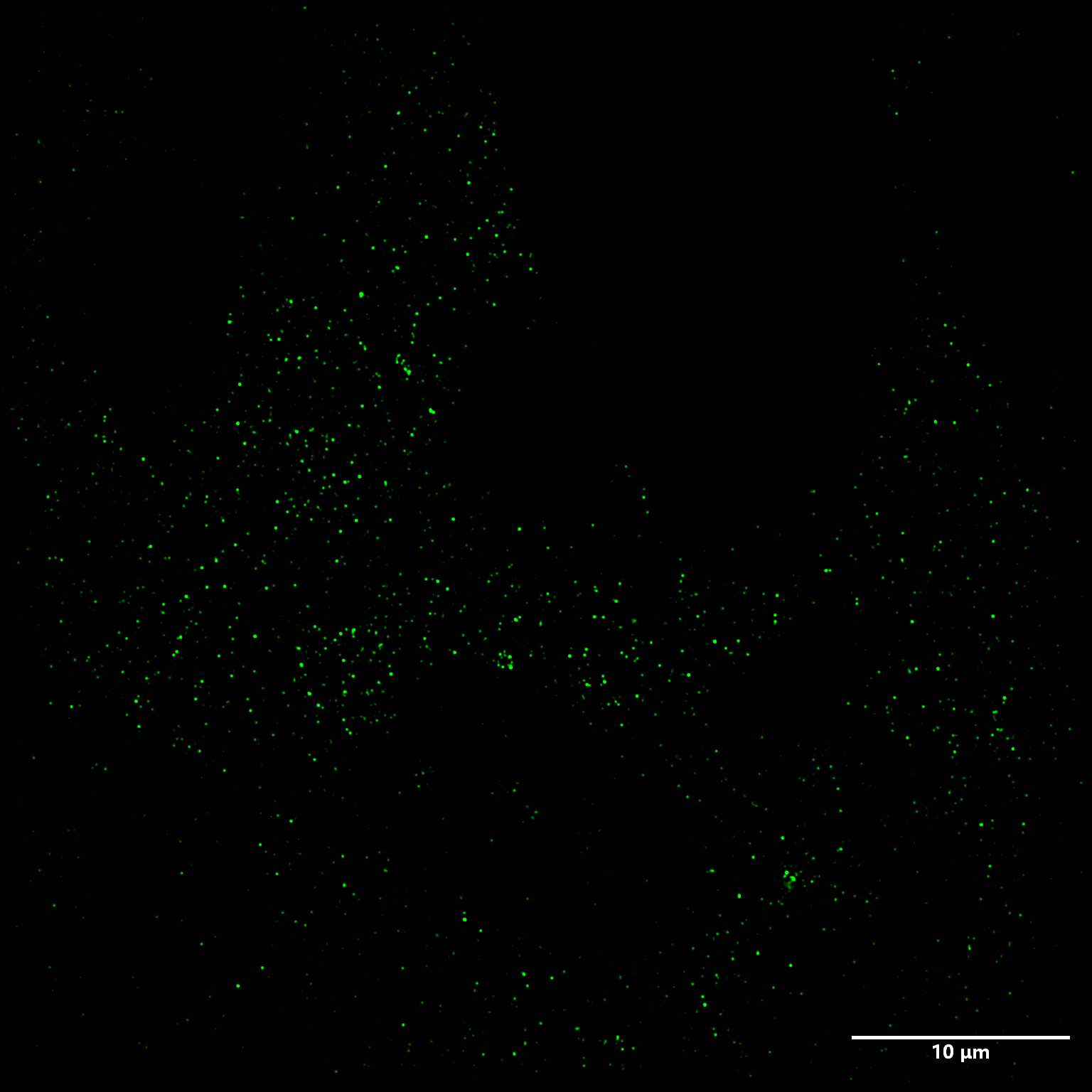

Supplement: Supplementary file 2 — Source data Fig. 1 [file 44319_2025_597_MOESM2_ESM.zip › Figure 1/1A/BICD2+Centrin/BICD2.bmp]

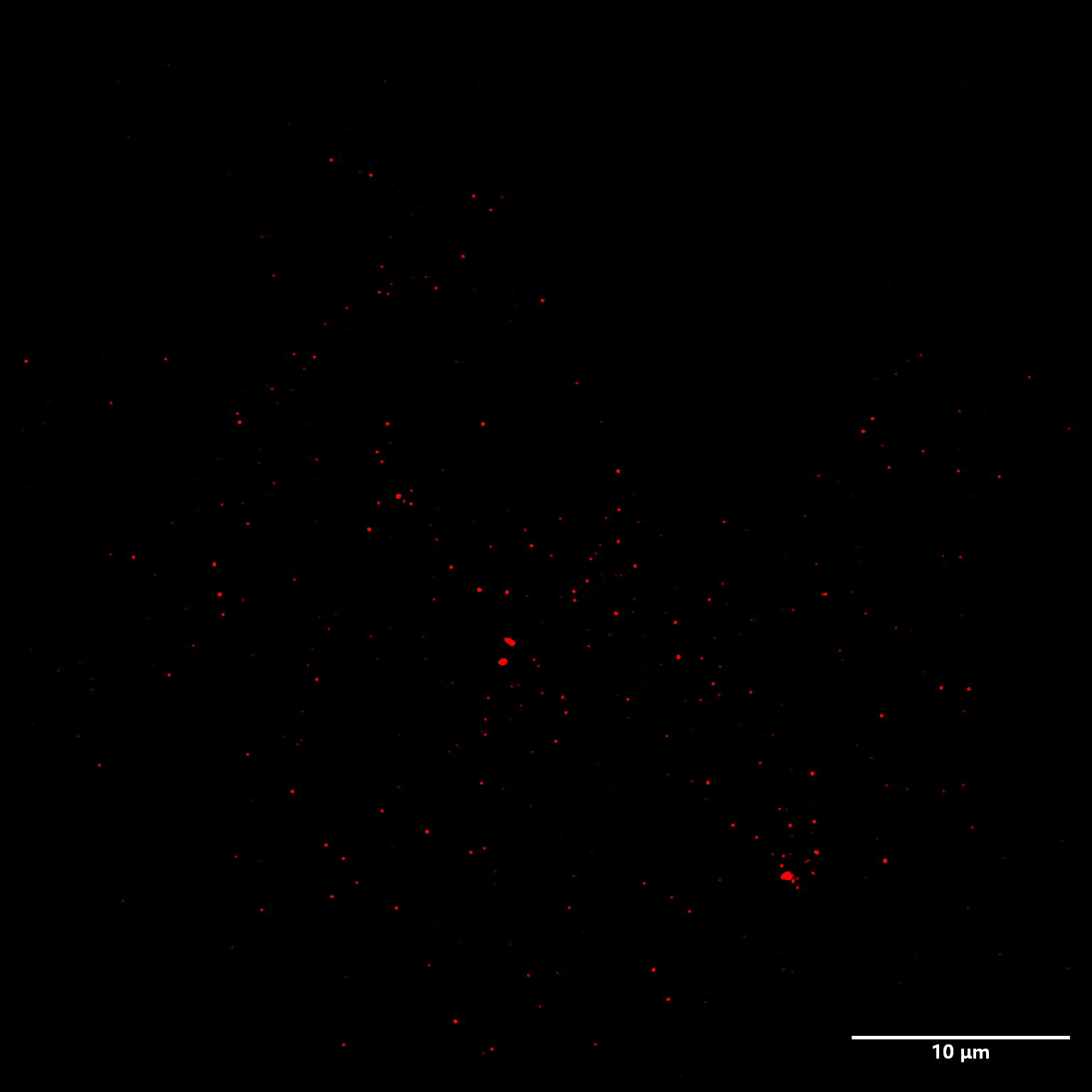

Supplement: Supplementary file 2 — Source data Fig. 1 [file 44319_2025_597_MOESM2_ESM.zip › Figure 1/1A/BICD2+Centrin/Centrin.bmp]

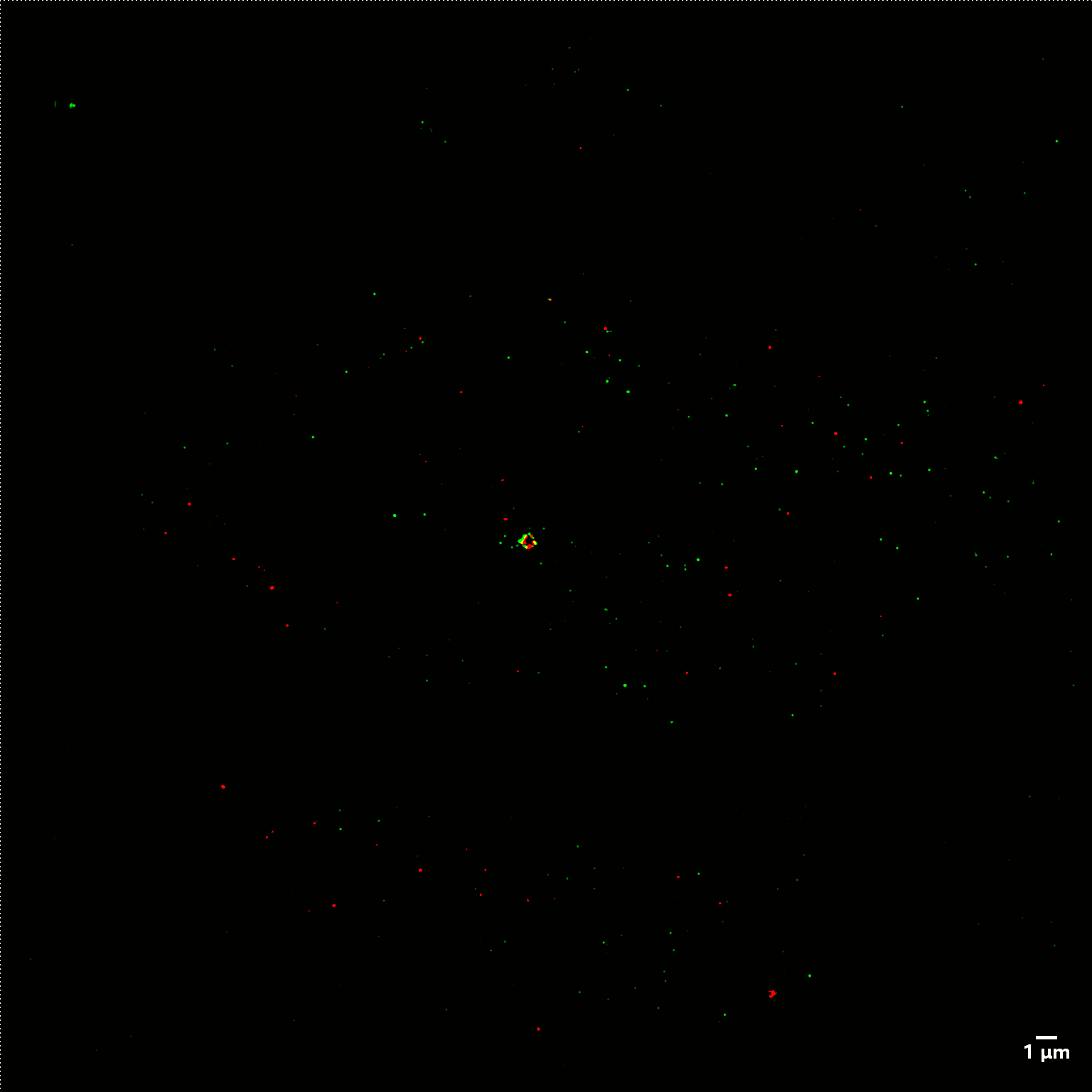

Supplement: Supplementary file 2 — Source data Fig. 1 [file 44319_2025_597_MOESM2_ESM.zip › Figure 1/1A/BICD2+CEP164/BICD2+CEP164.bmp]

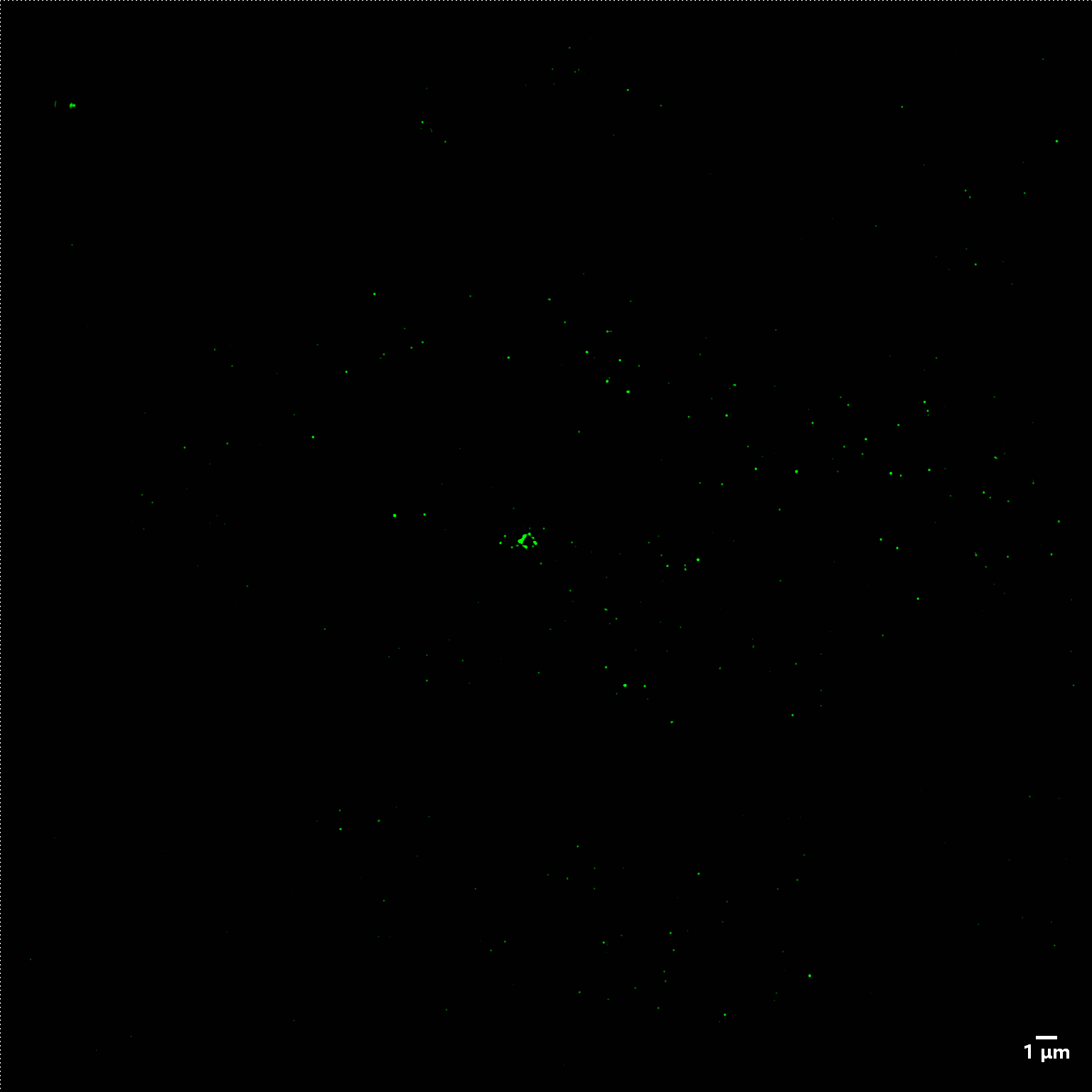

Supplement: Supplementary file 2 — Source data Fig. 1 [file 44319_2025_597_MOESM2_ESM.zip › Figure 1/1A/BICD2+CEP164/BICD2.bmp]

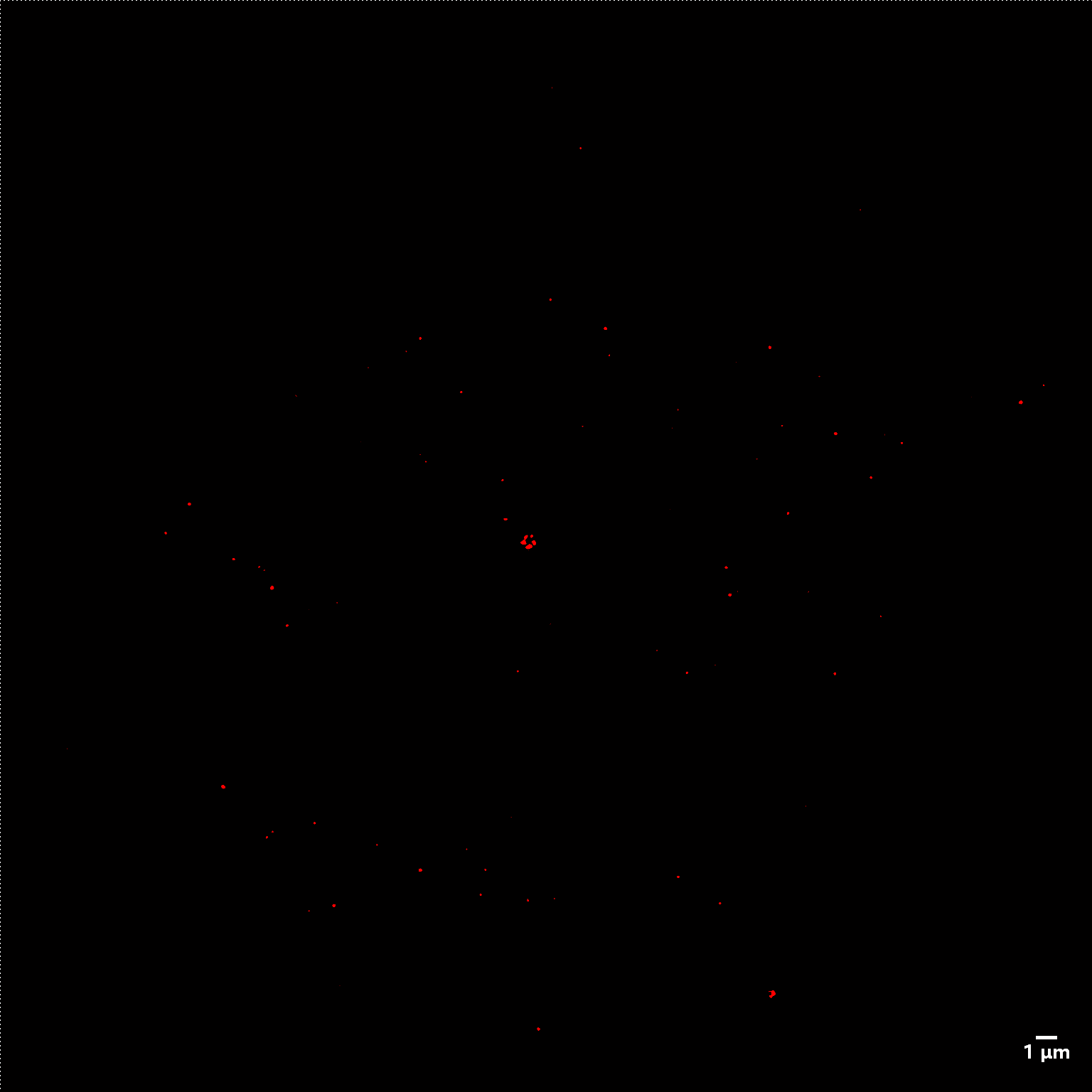

Supplement: Supplementary file 2 — Source data Fig. 1 [file 44319_2025_597_MOESM2_ESM.zip › Figure 1/1A/BICD2+CEP164/CEP164.bmp]

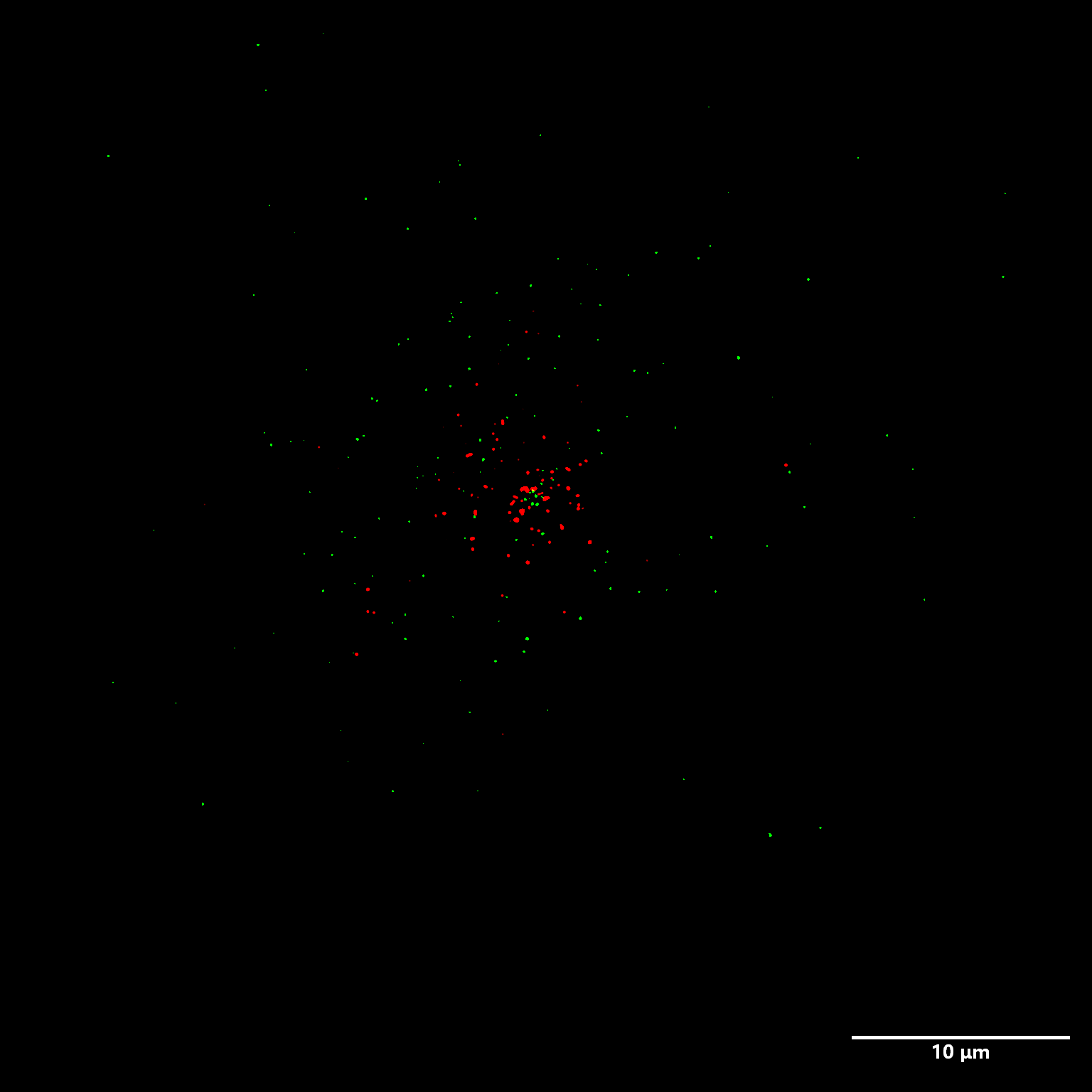

Supplement: Supplementary file 2 — Source data Fig. 1 [file 44319_2025_597_MOESM2_ESM.zip › Figure 1/1A/BICD2+PCM1/BICD2+PCM1.bmp]

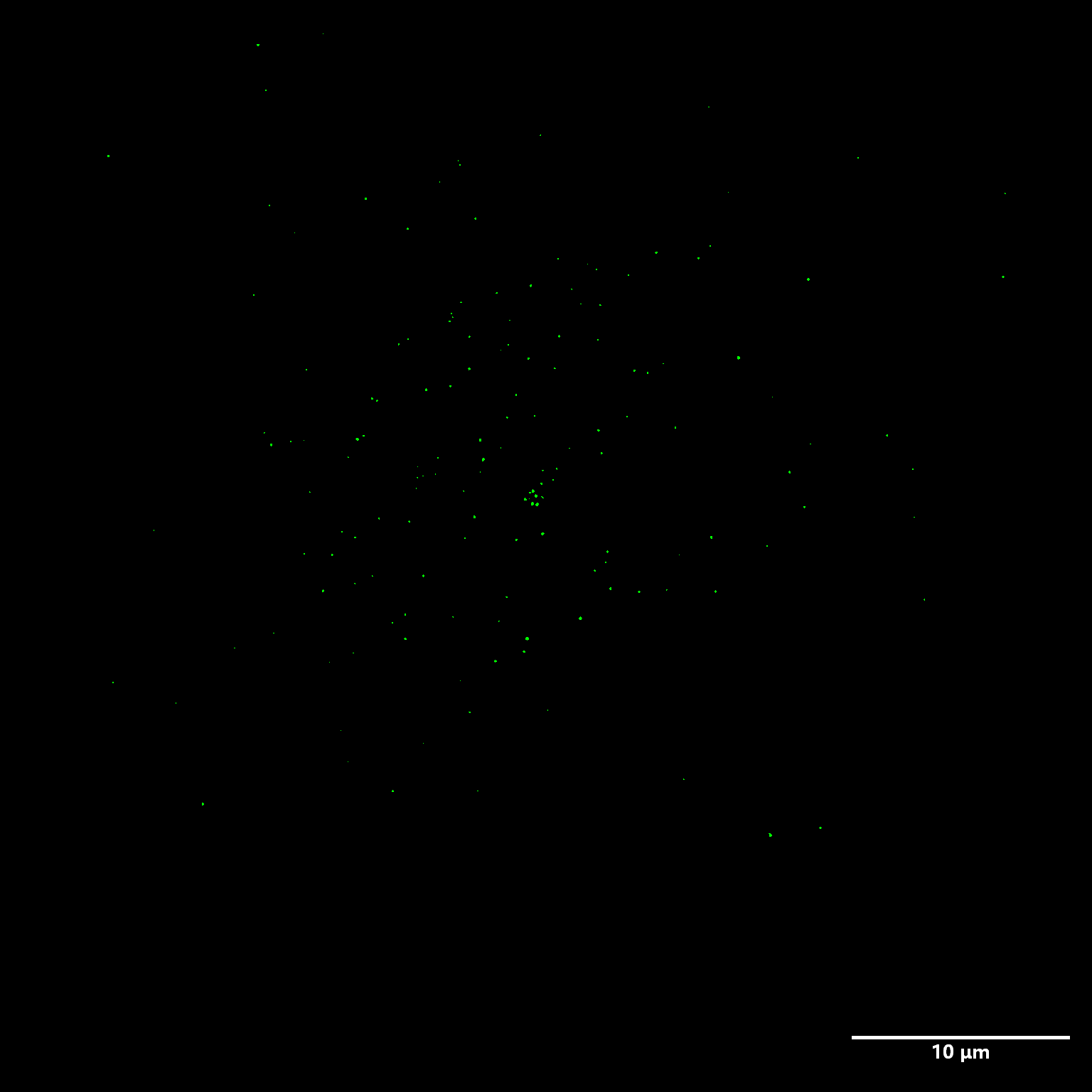

Supplement: Supplementary file 2 — Source data Fig. 1 [file 44319_2025_597_MOESM2_ESM.zip › Figure 1/1A/BICD2+PCM1/BICD2.bmp]

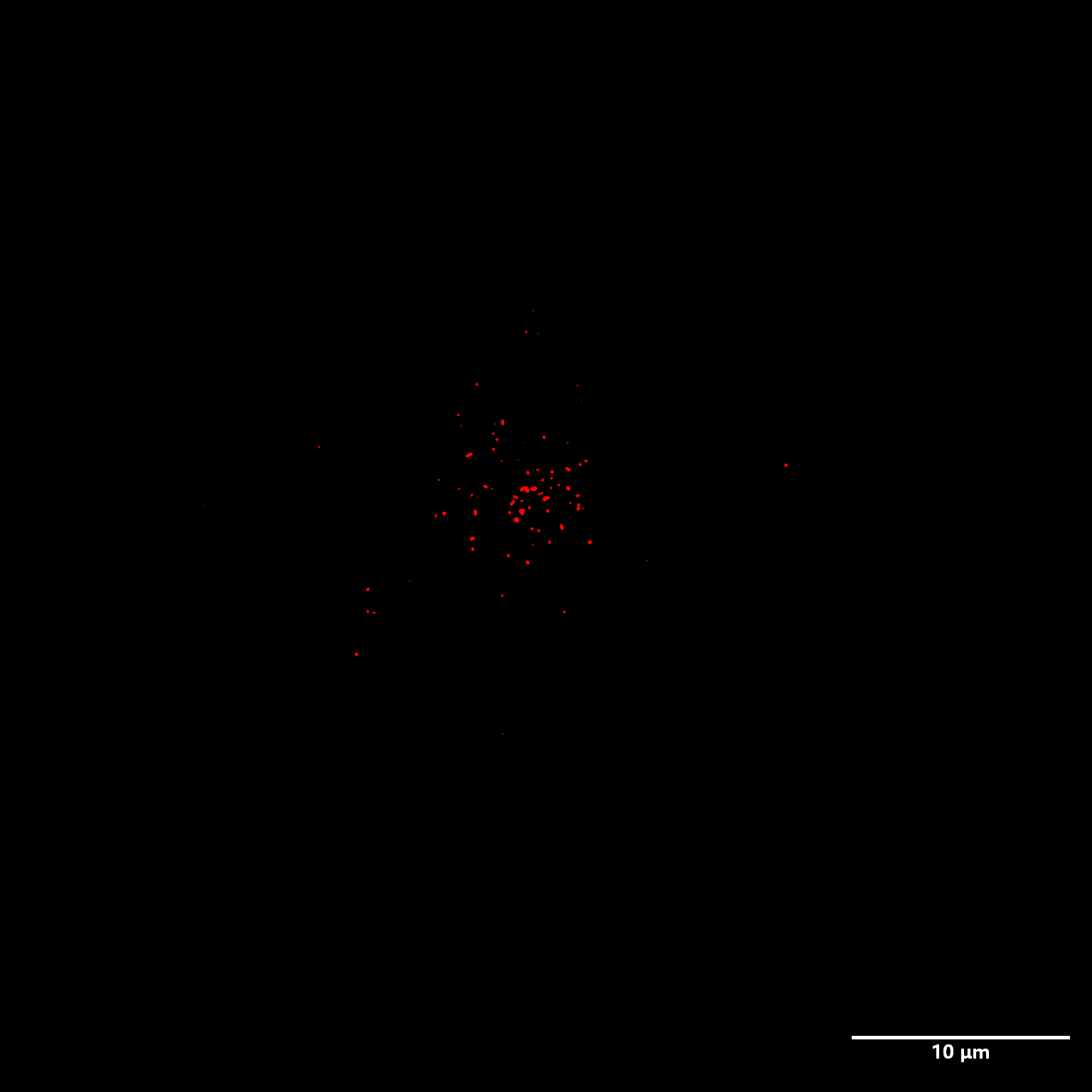

Supplement: Supplementary file 2 — Source data Fig. 1 [file 44319_2025_597_MOESM2_ESM.zip › Figure 1/1A/BICD2+PCM1/PCM1.bmp]

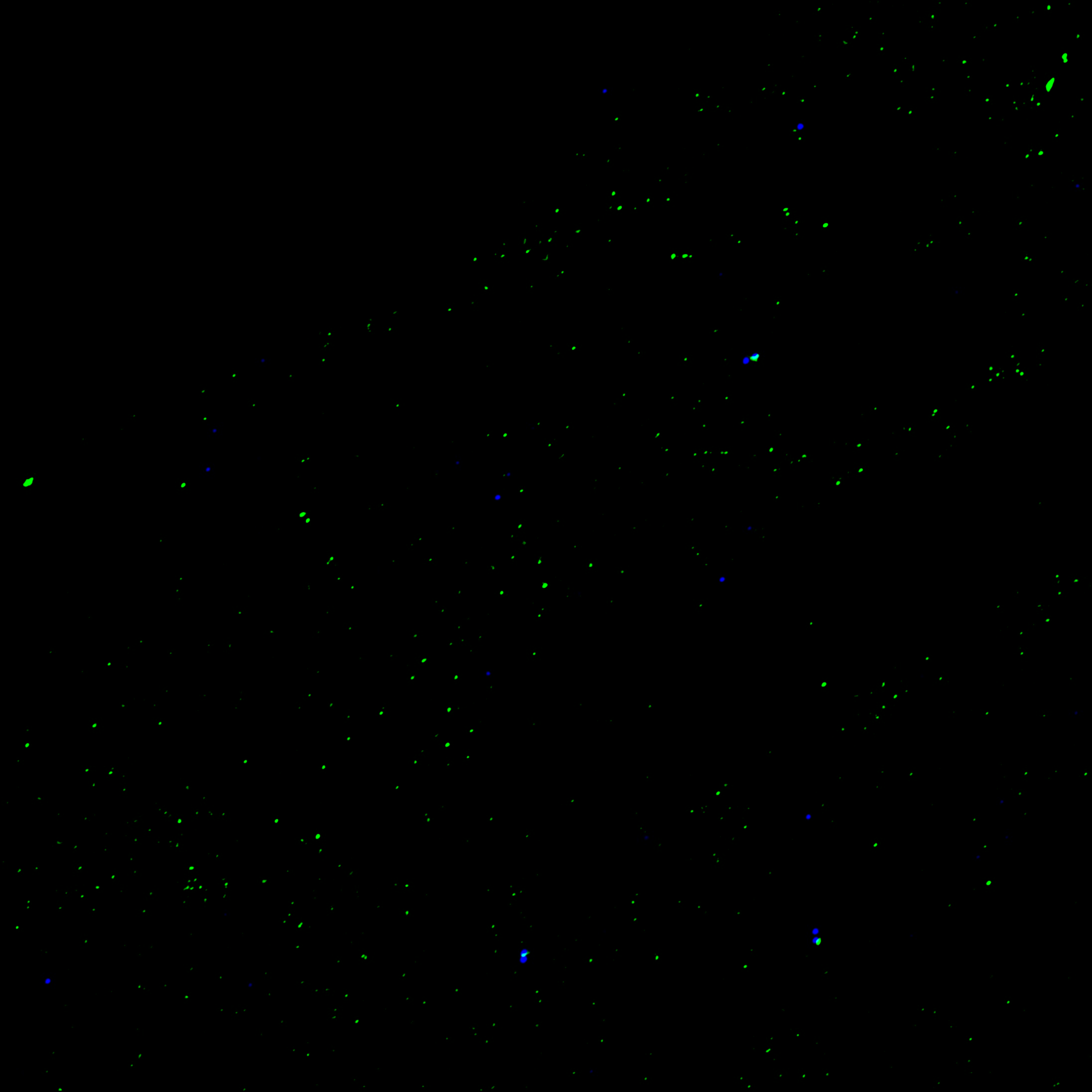

Supplement: Supplementary file 2 — Source data Fig. 1 [file 44319_2025_597_MOESM2_ESM.zip › Figure 1/1B/BICD2+Centrin.tif]

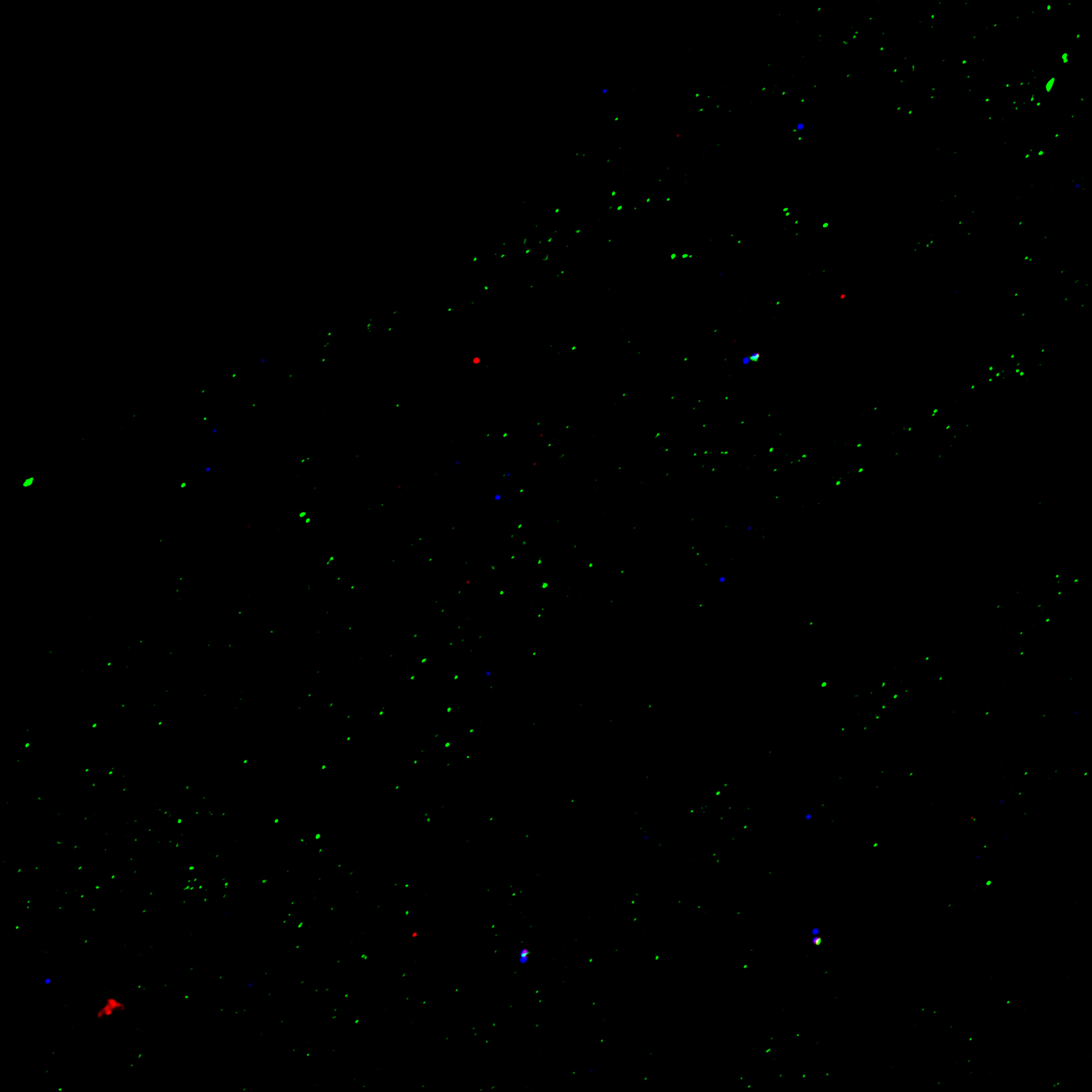

Supplement: Supplementary file 2 — Source data Fig. 1 [file 44319_2025_597_MOESM2_ESM.zip › Figure 1/1B/BICD2+CEP164+Centrin.tif]

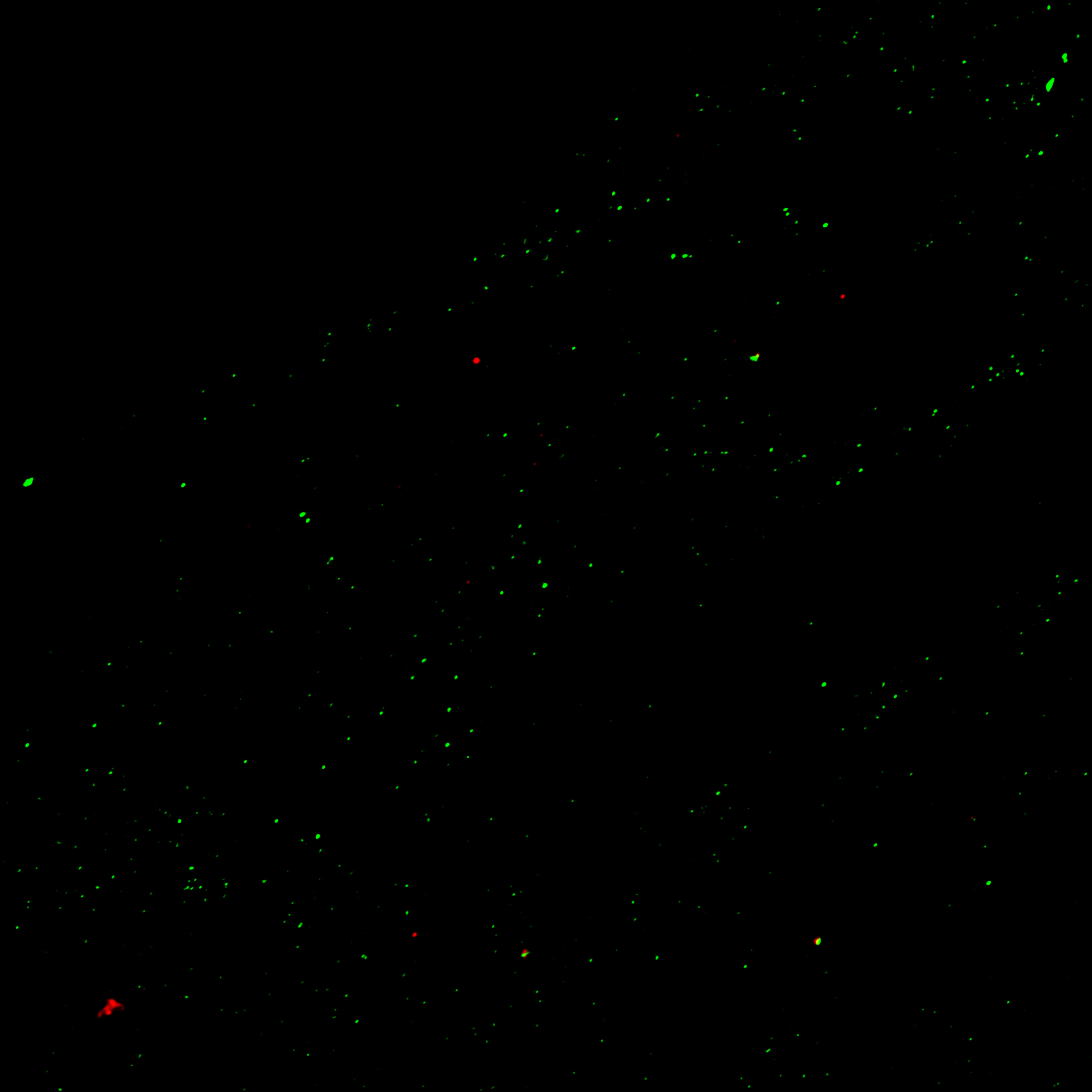

Supplement: Supplementary file 2 — Source data Fig. 1 [file 44319_2025_597_MOESM2_ESM.zip › Figure 1/1B/BICD2+CEP164.tif]

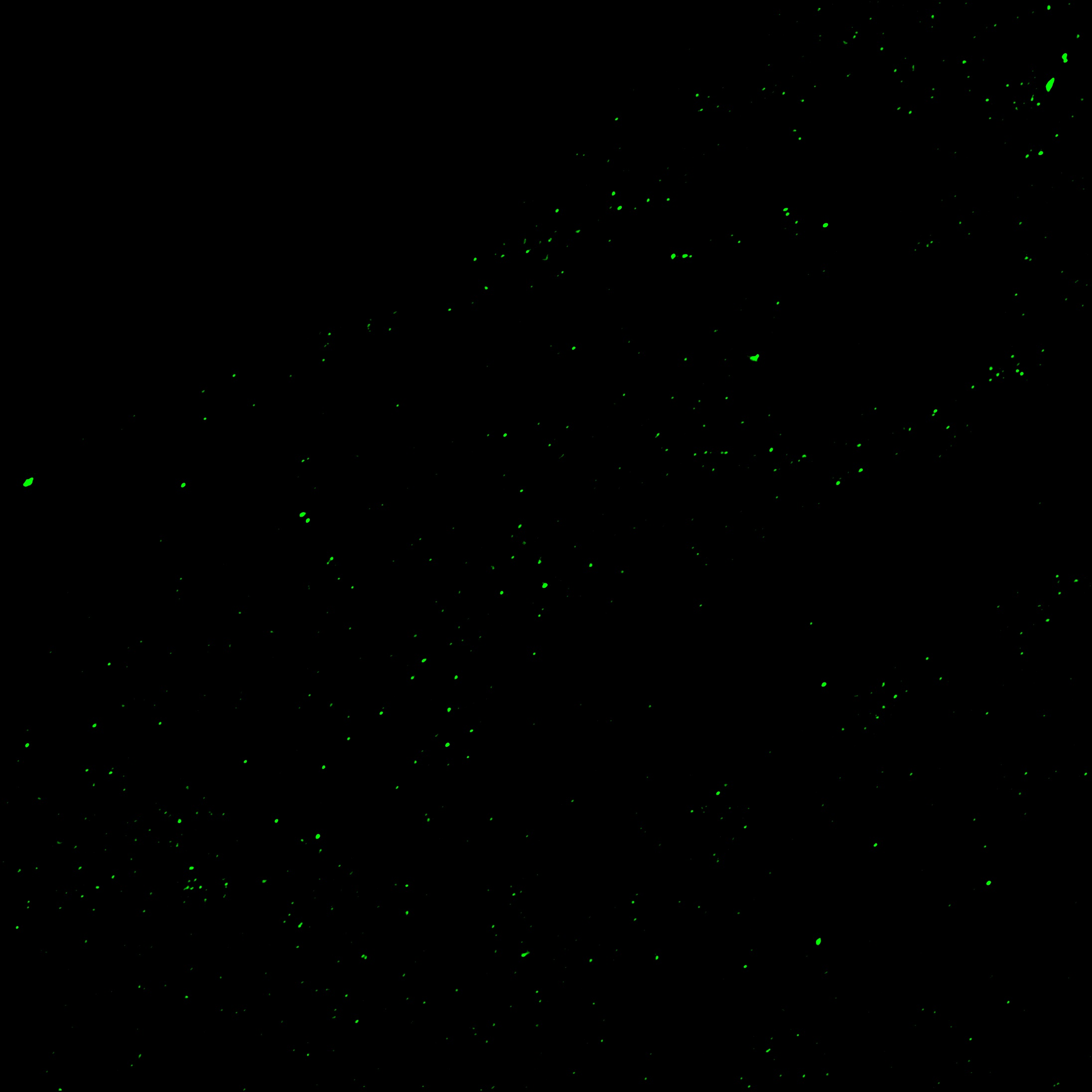

Supplement: Supplementary file 2 — Source data Fig. 1 [file 44319_2025_597_MOESM2_ESM.zip › Figure 1/1B/BICD2.tif]

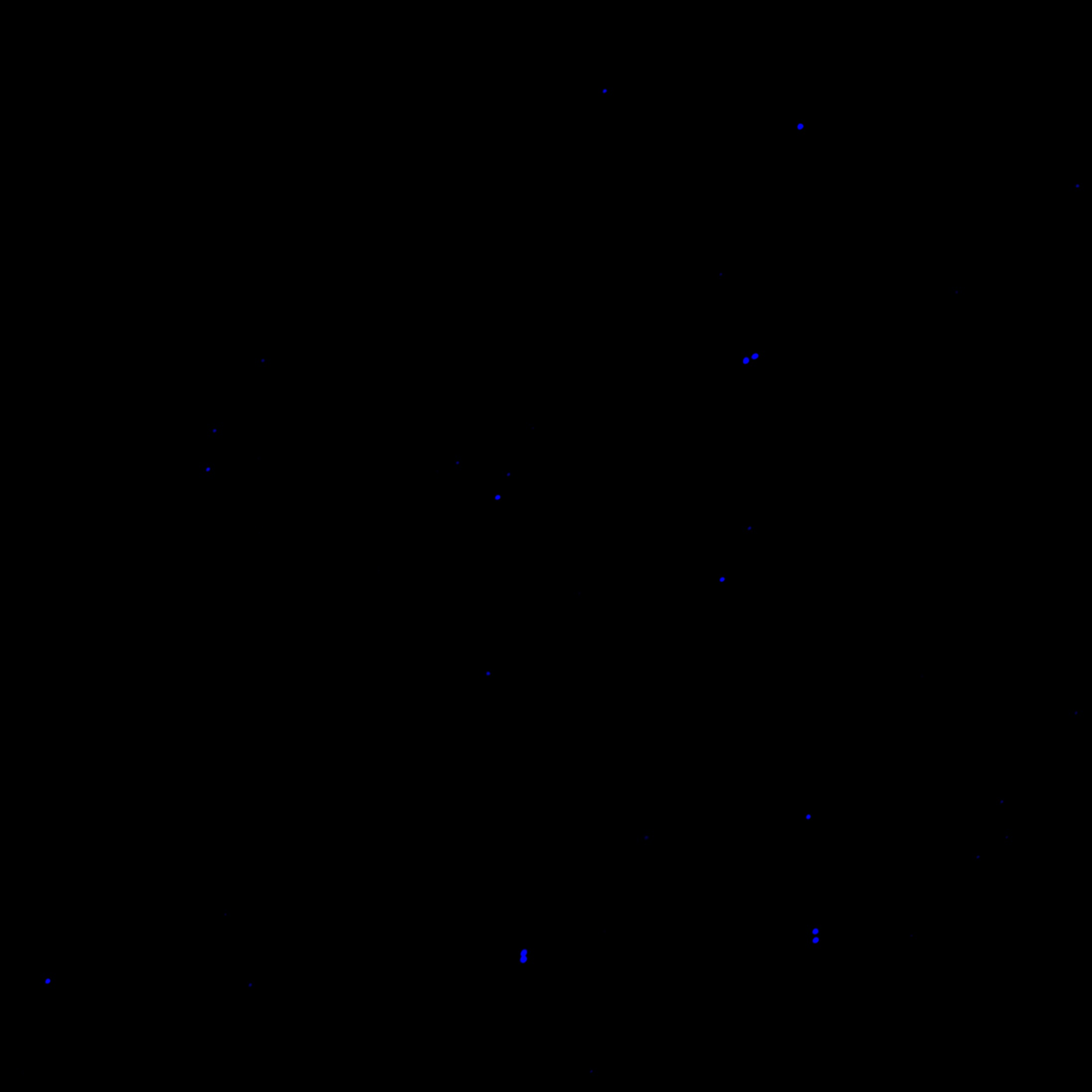

Supplement: Supplementary file 2 — Source data Fig. 1 [file 44319_2025_597_MOESM2_ESM.zip › Figure 1/1B/Centrin.tif]

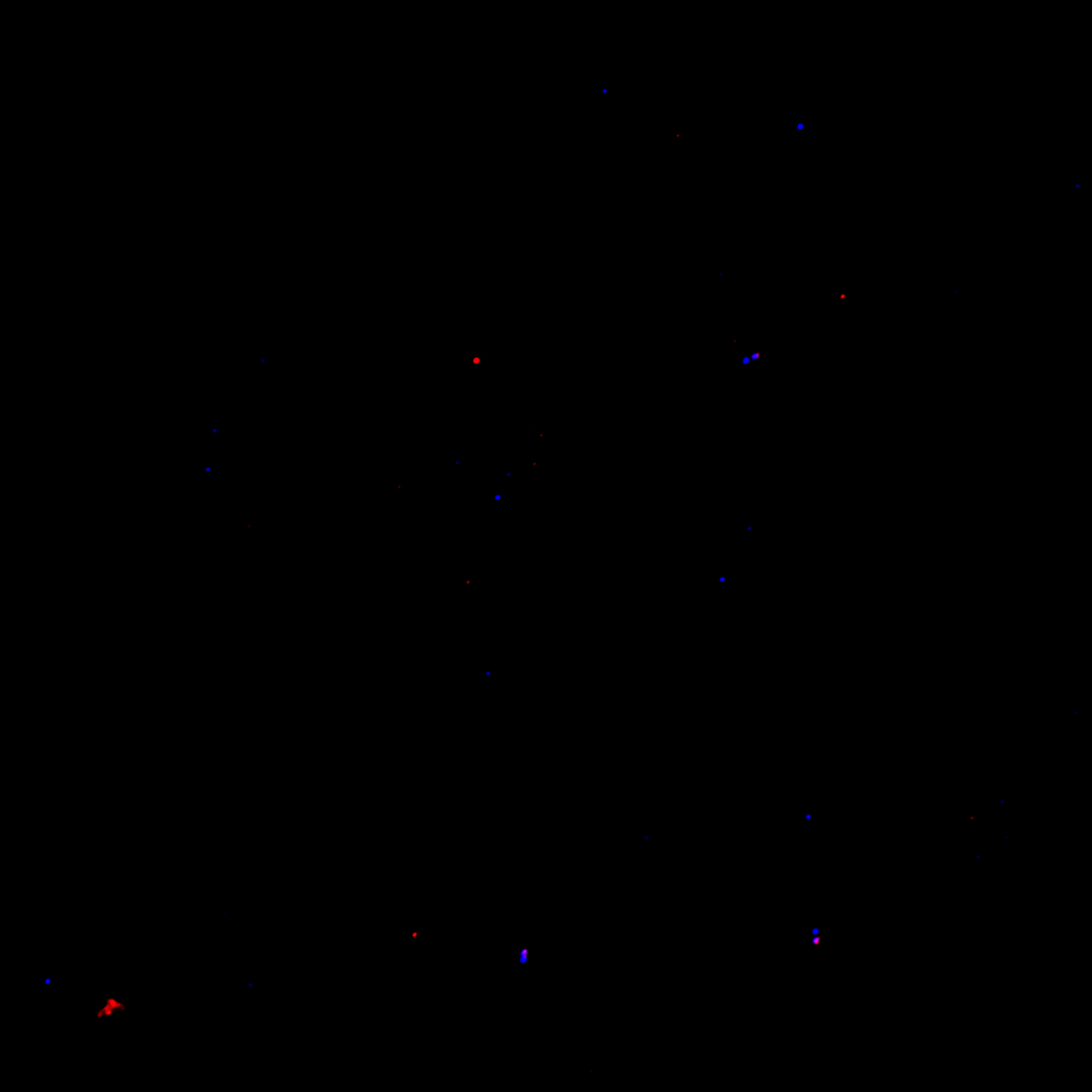

Supplement: Supplementary file 2 — Source data Fig. 1 [file 44319_2025_597_MOESM2_ESM.zip › Figure 1/1B/CEP164+Centrin.tif]

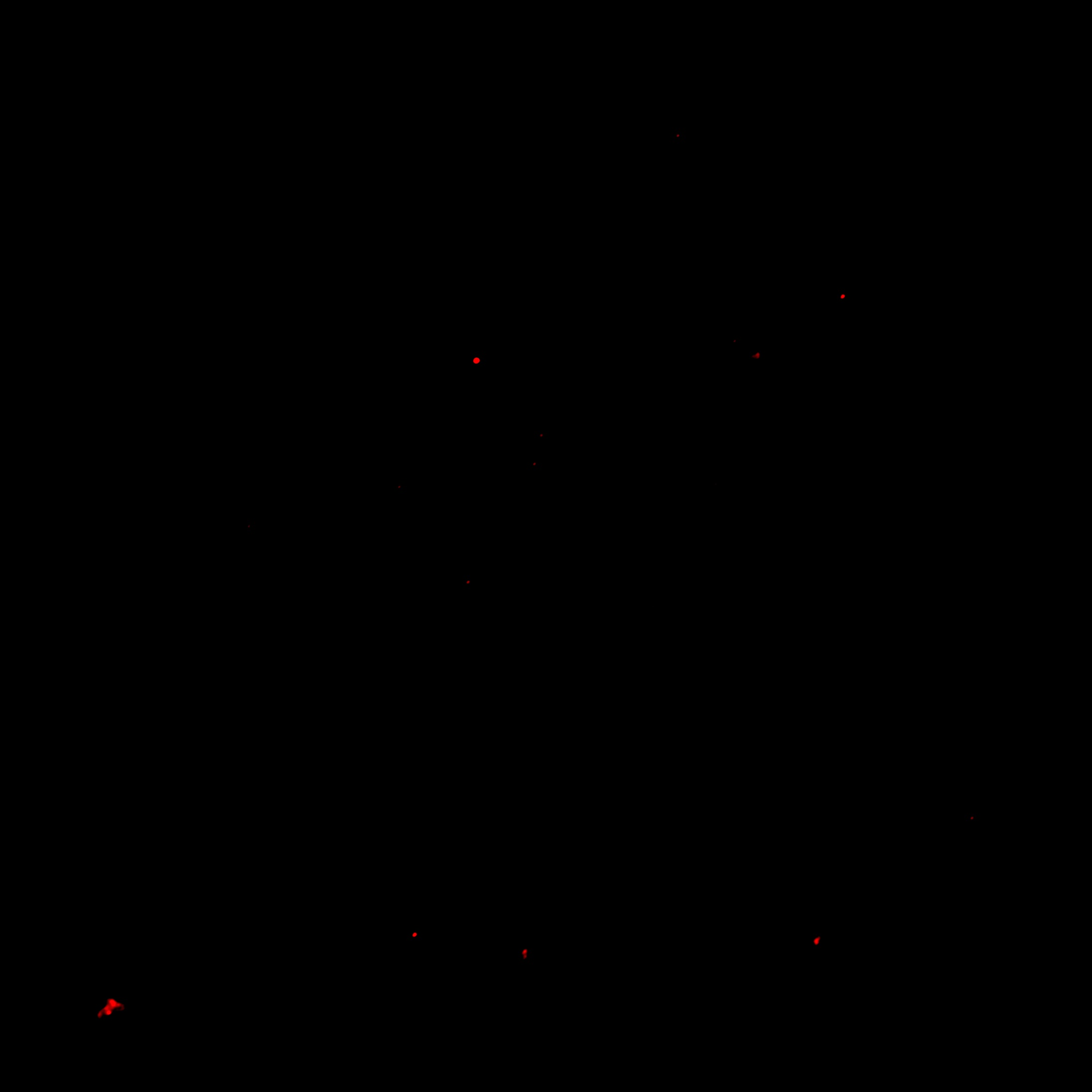

Supplement: Supplementary file 2 — Source data Fig. 1 [file 44319_2025_597_MOESM2_ESM.zip › Figure 1/1B/CEP164.tif]

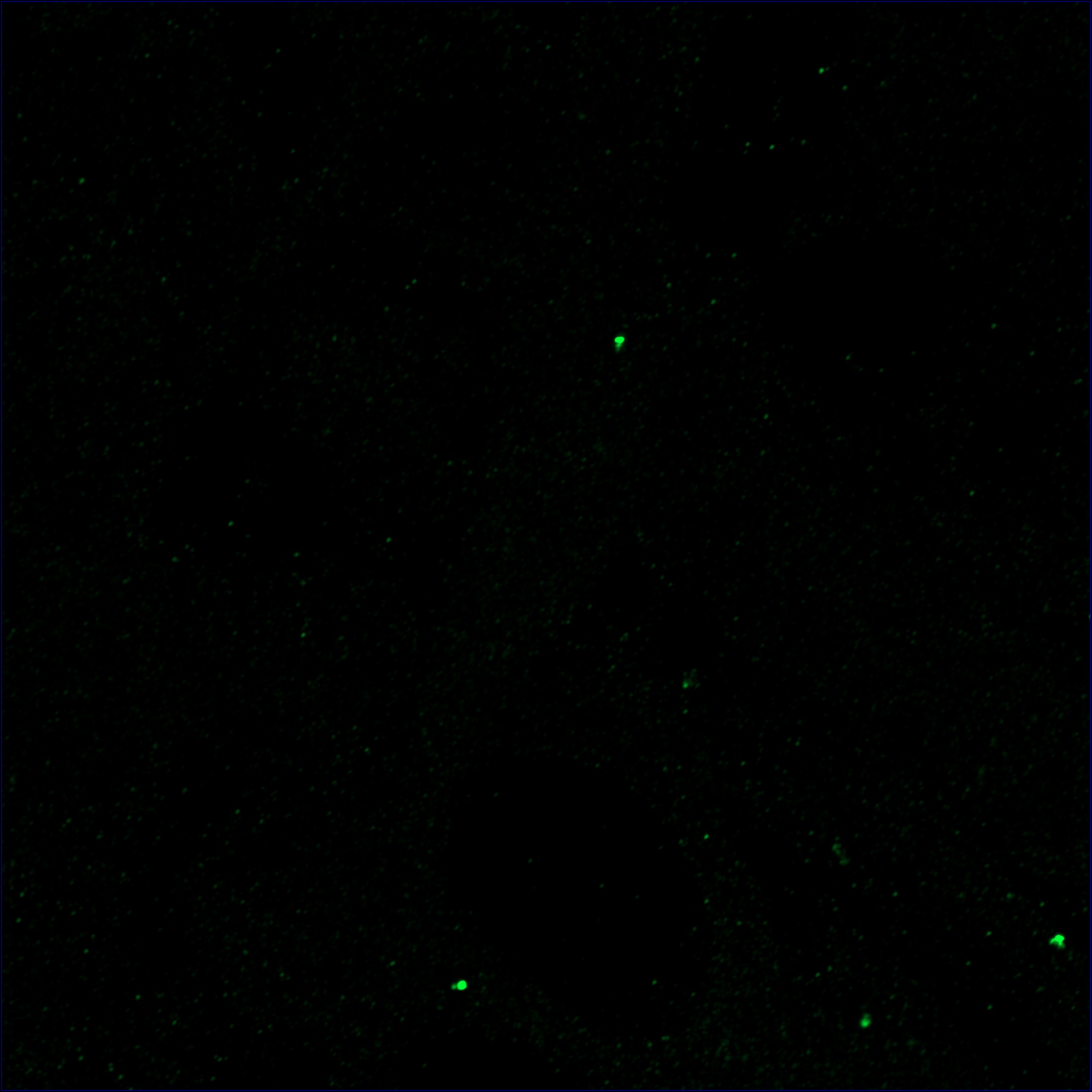

Supplement: Supplementary file 2 — Source data Fig. 1 [file 44319_2025_597_MOESM2_ESM.zip › Figure 1/1E/BICD2-flag-1-271/CEP164.jpg]

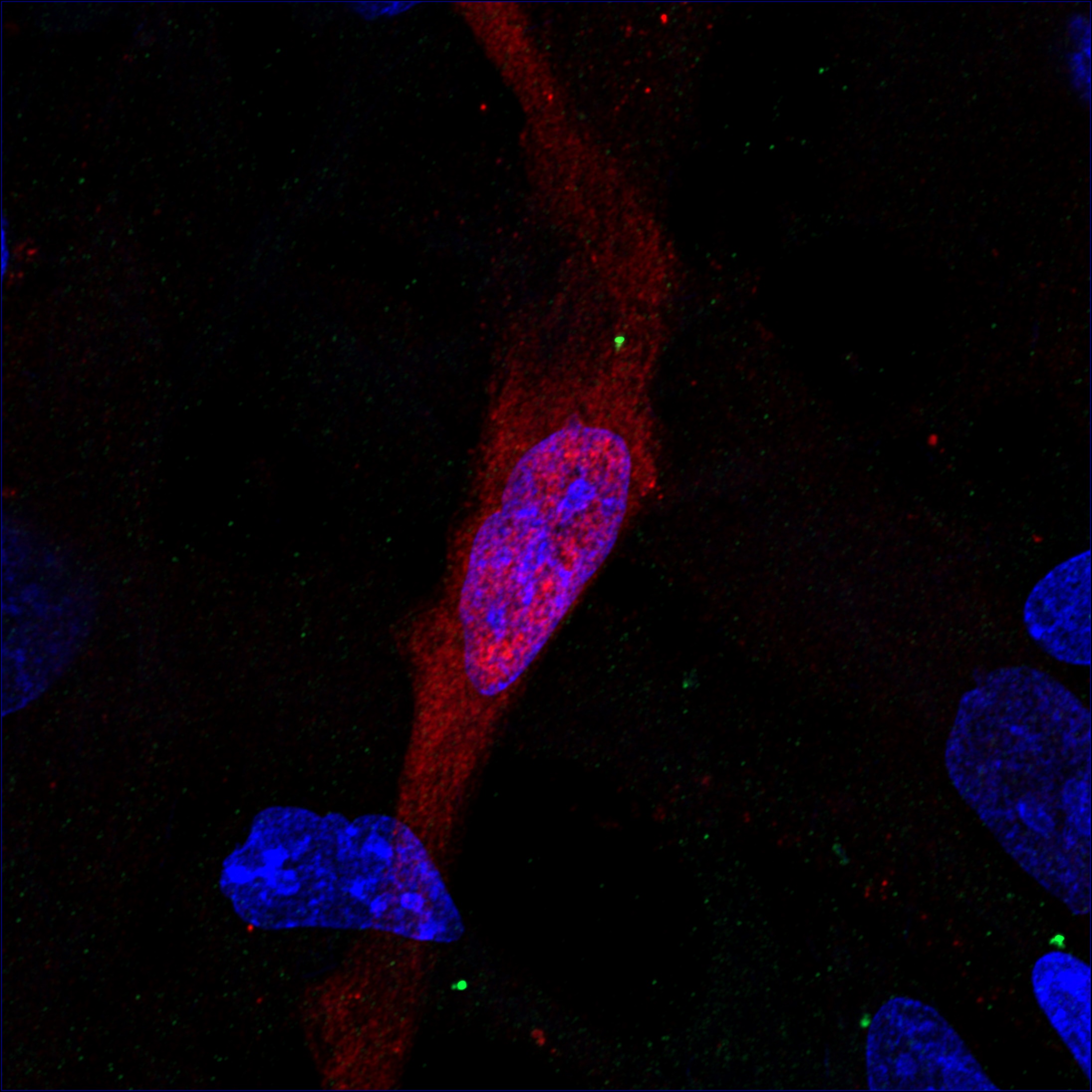

Supplement: Supplementary file 2 — Source data Fig. 1 [file 44319_2025_597_MOESM2_ESM.zip › Figure 1/1E/BICD2-flag-1-271/Flag+CEP164+DAPI.jpg]

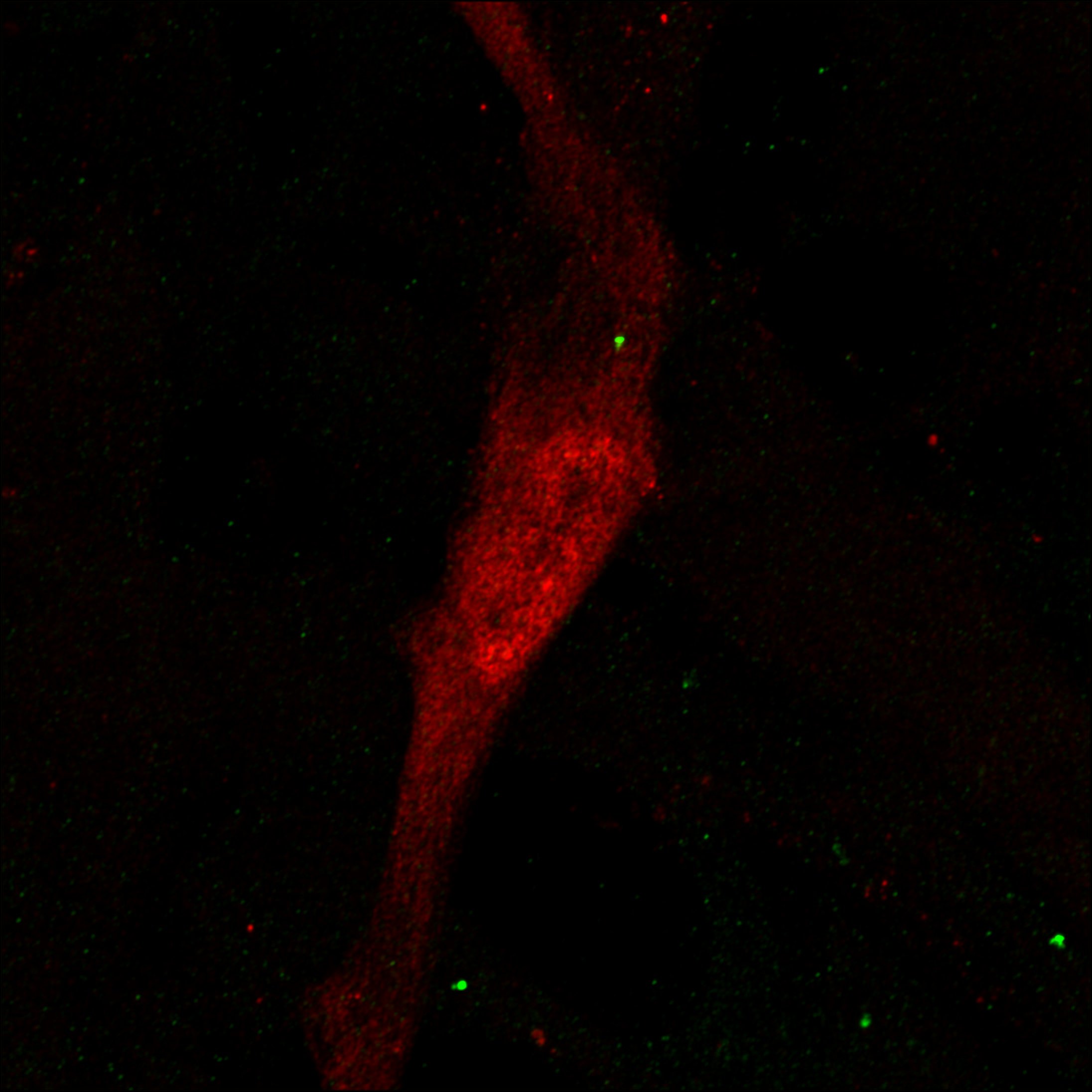

Supplement: Supplementary file 2 — Source data Fig. 1 [file 44319_2025_597_MOESM2_ESM.zip › Figure 1/1E/BICD2-flag-1-271/Flag+CEP164.jpg]

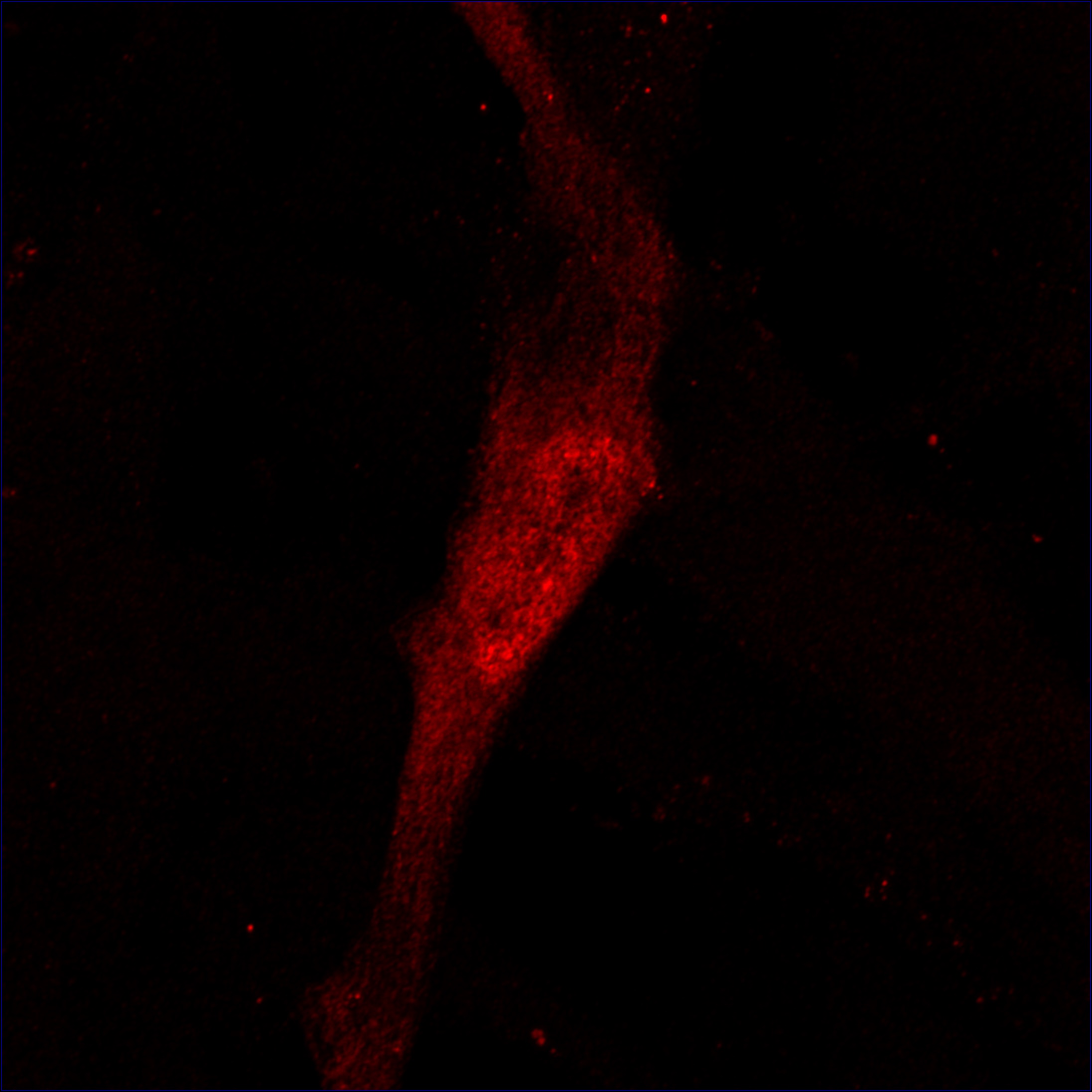

Supplement: Supplementary file 2 — Source data Fig. 1 [file 44319_2025_597_MOESM2_ESM.zip › Figure 1/1E/BICD2-flag-1-271/Flag.jpg]

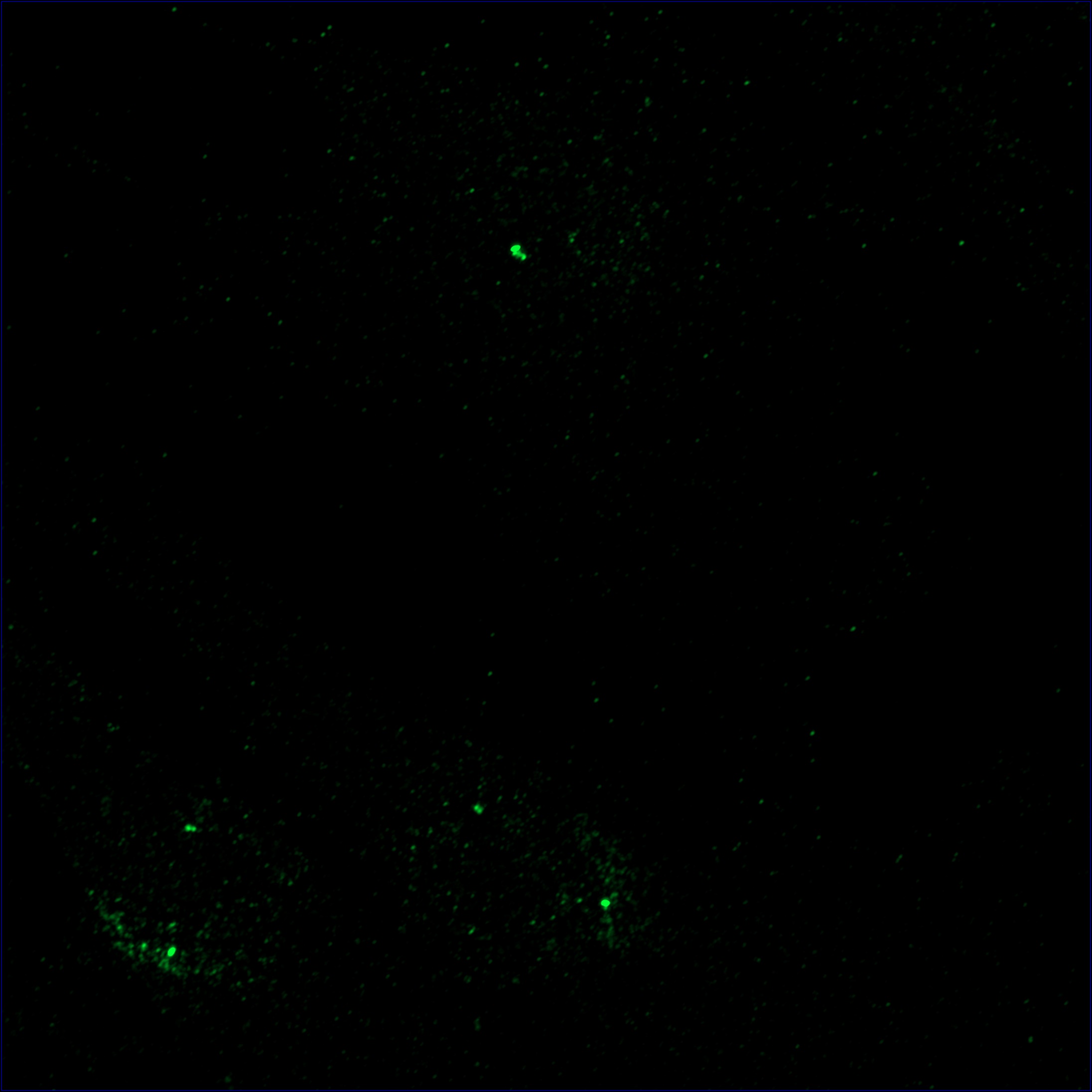

Supplement: Supplementary file 2 — Source data Fig. 1 [file 44319_2025_597_MOESM2_ESM.zip › Figure 1/1E/BICD2-flag-1-271+541-824/CEP164.jpg]

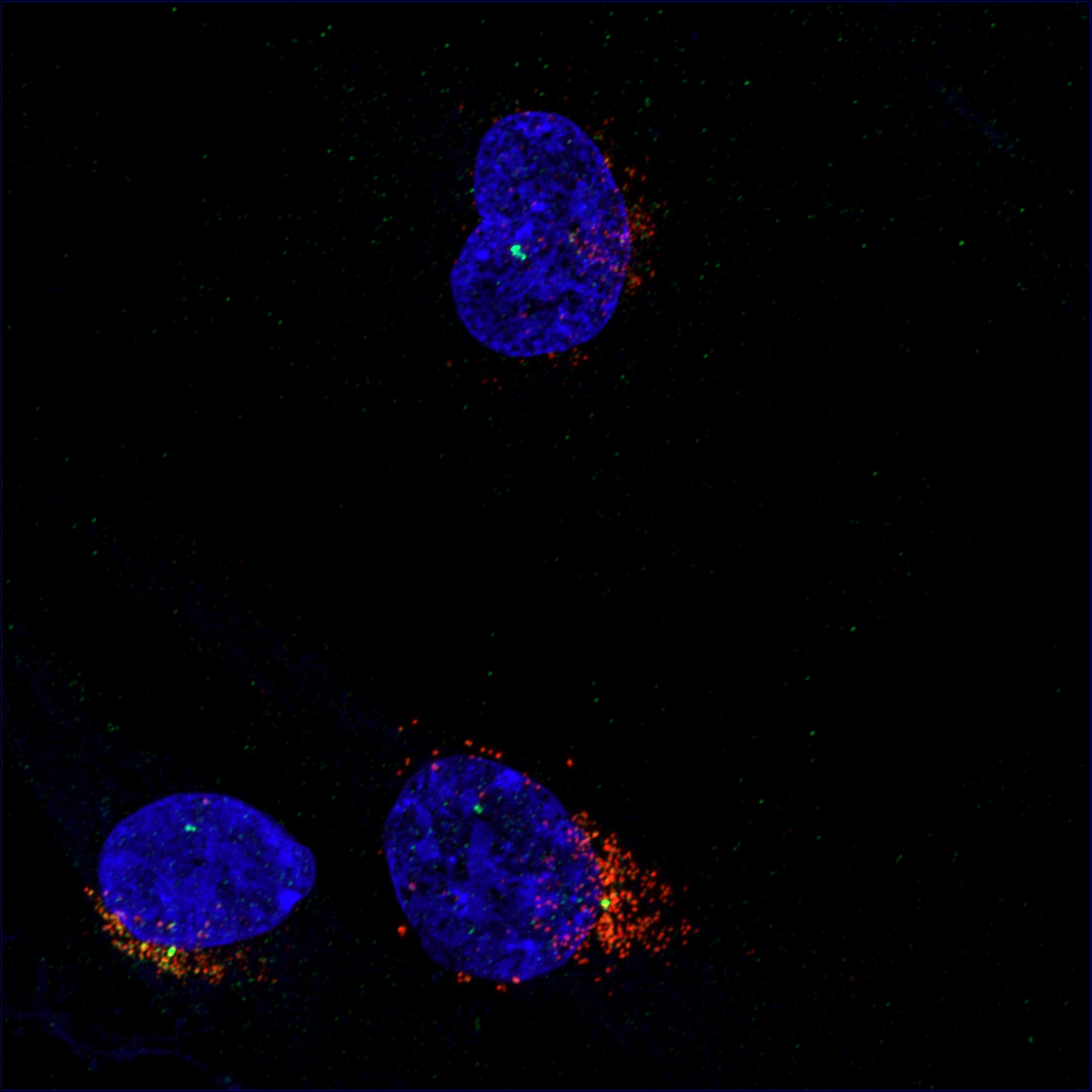

Supplement: Supplementary file 2 — Source data Fig. 1 [file 44319_2025_597_MOESM2_ESM.zip › Figure 1/1E/BICD2-flag-1-271+541-824/Flag+CEP164+DAPI.jpg]

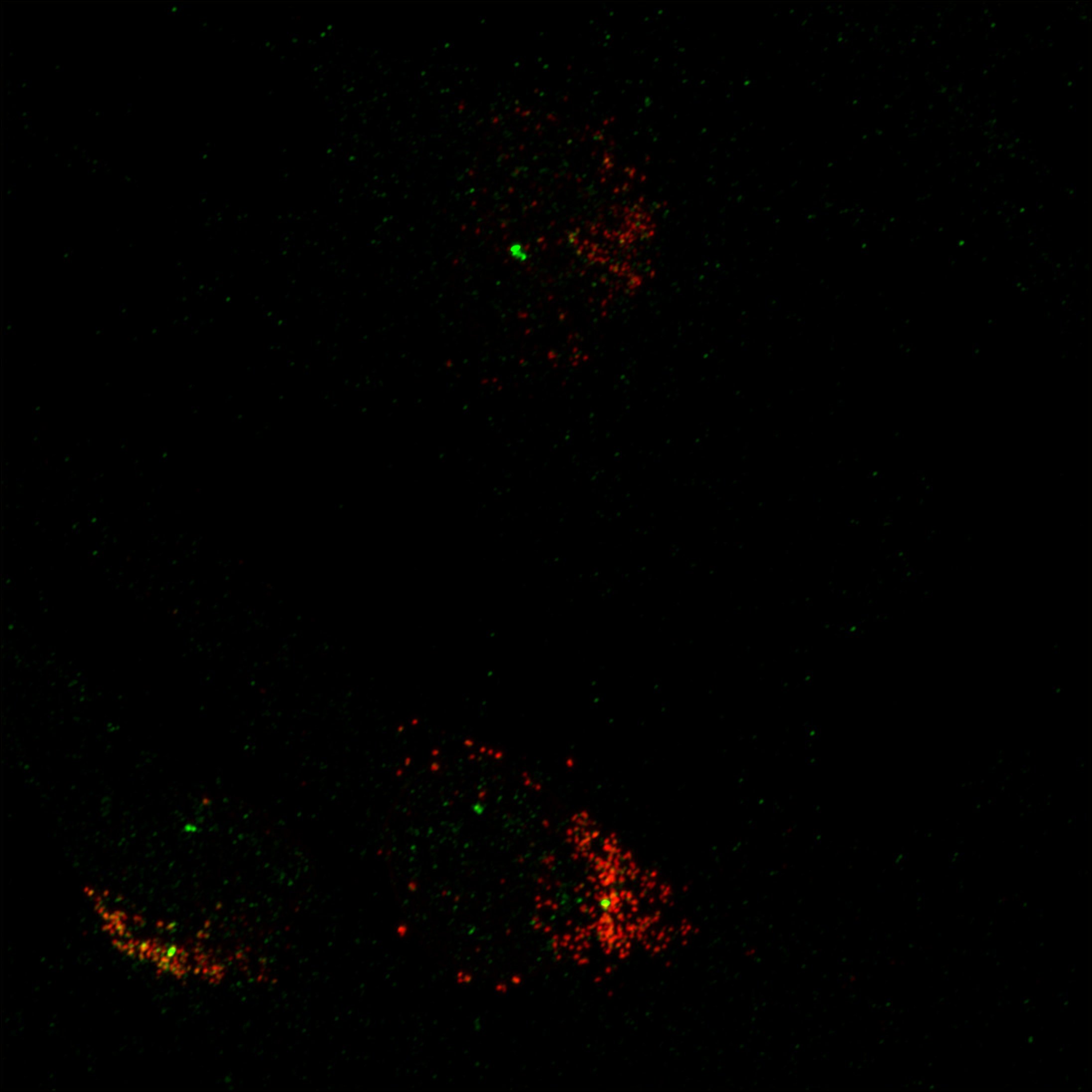

Supplement: Supplementary file 2 — Source data Fig. 1 [file 44319_2025_597_MOESM2_ESM.zip › Figure 1/1E/BICD2-flag-1-271+541-824/Flag+CEP164.jpg]

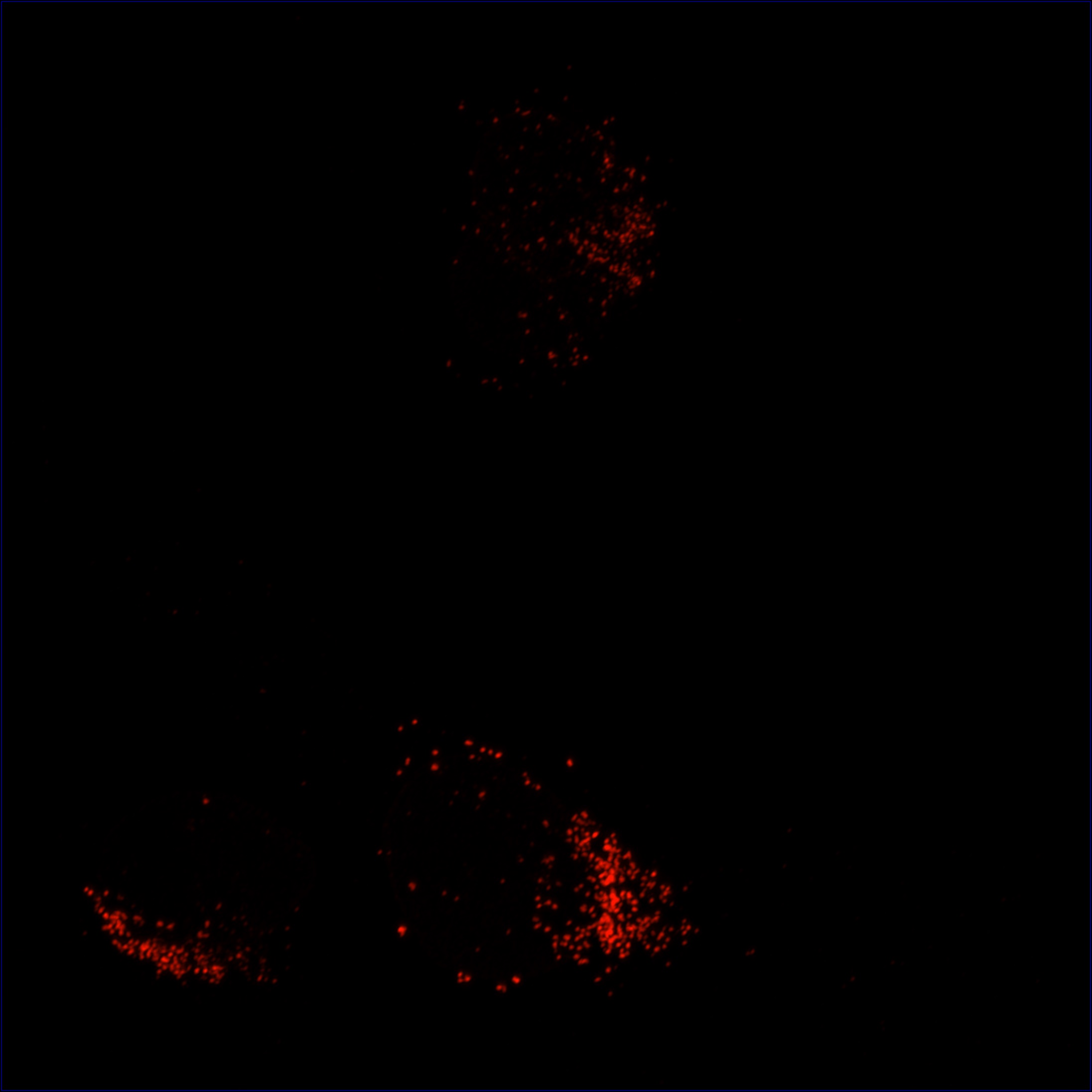

Supplement: Supplementary file 2 — Source data Fig. 1 [file 44319_2025_597_MOESM2_ESM.zip › Figure 1/1E/BICD2-flag-1-271+541-824/Flag.jpg]

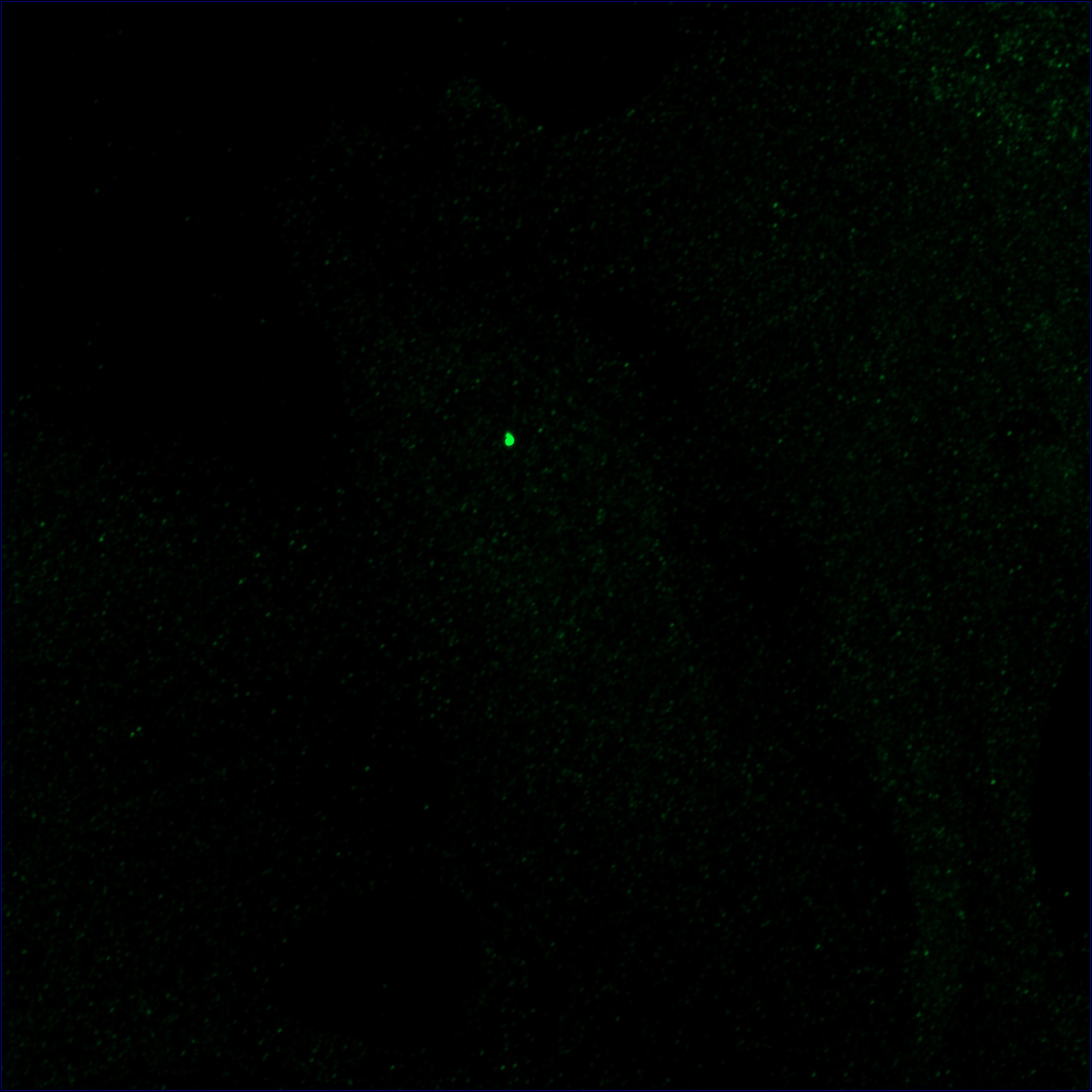

Supplement: Supplementary file 2 — Source data Fig. 1 [file 44319_2025_597_MOESM2_ESM.zip › Figure 1/1E/BICD2-flag-1-540/CEP164.jpg]

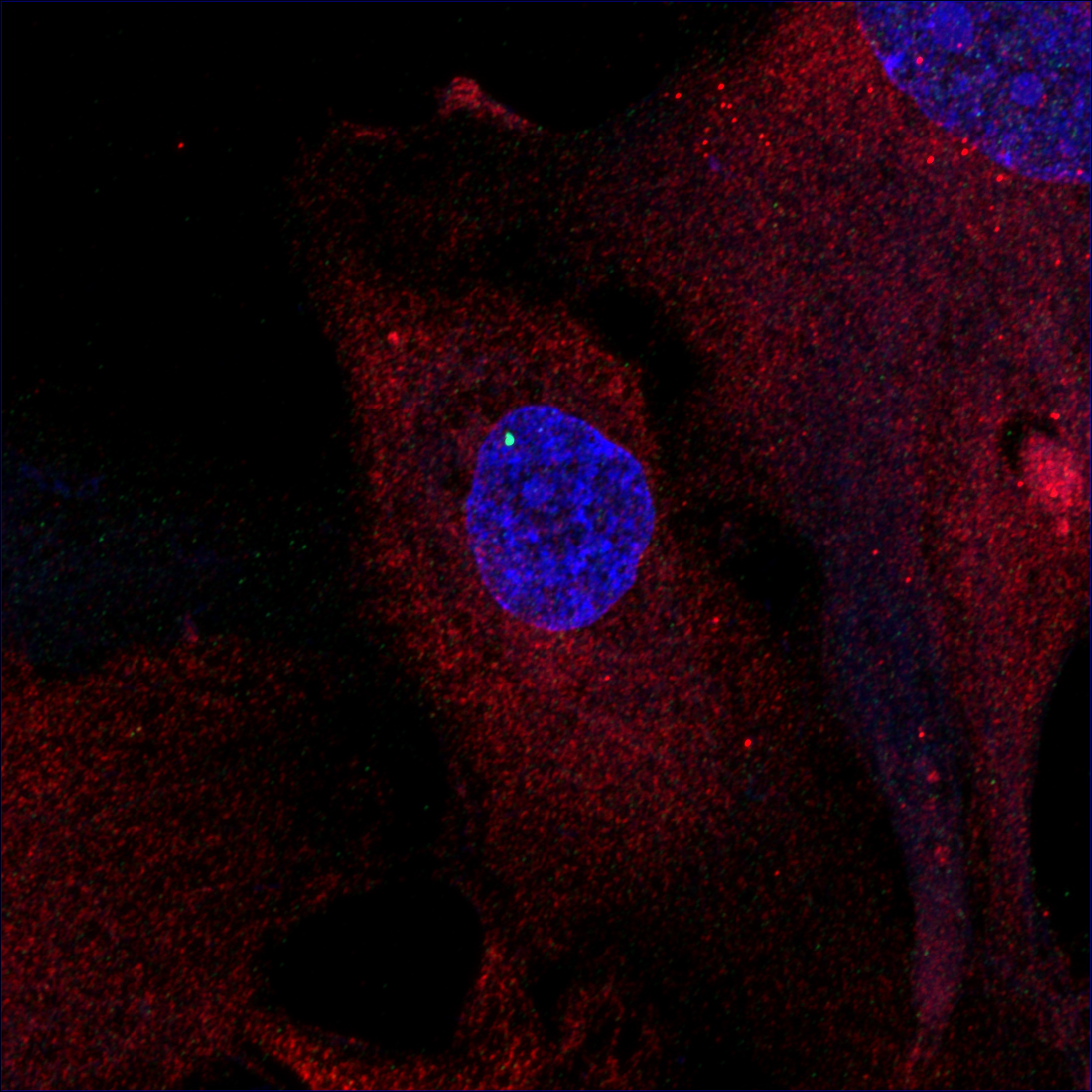

Supplement: Supplementary file 2 — Source data Fig. 1 [file 44319_2025_597_MOESM2_ESM.zip › Figure 1/1E/BICD2-flag-1-540/Flag+CEP164+DAPI.jpg]

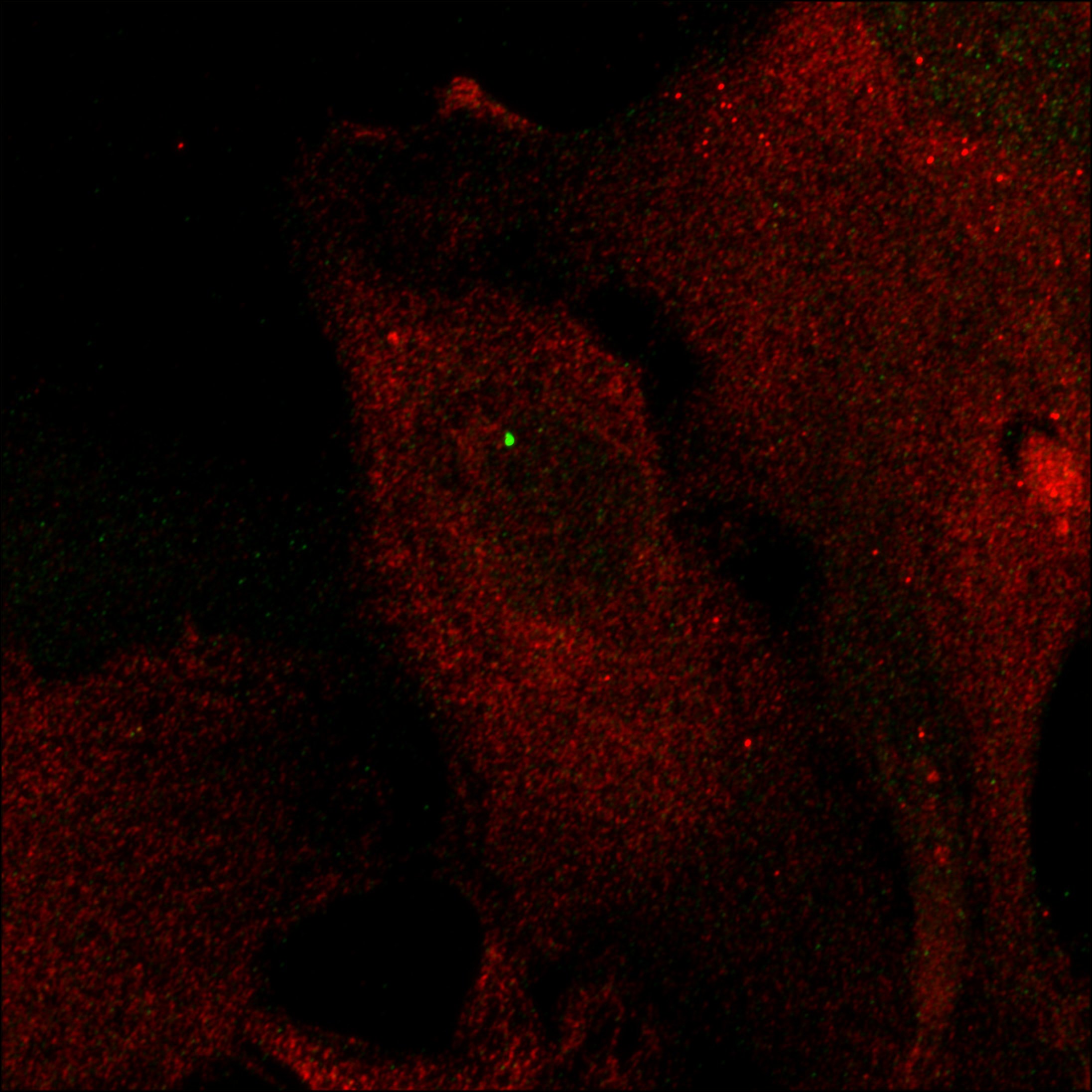

Supplement: Supplementary file 2 — Source data Fig. 1 [file 44319_2025_597_MOESM2_ESM.zip › Figure 1/1E/BICD2-flag-1-540/Flag+CEP164.jpg]

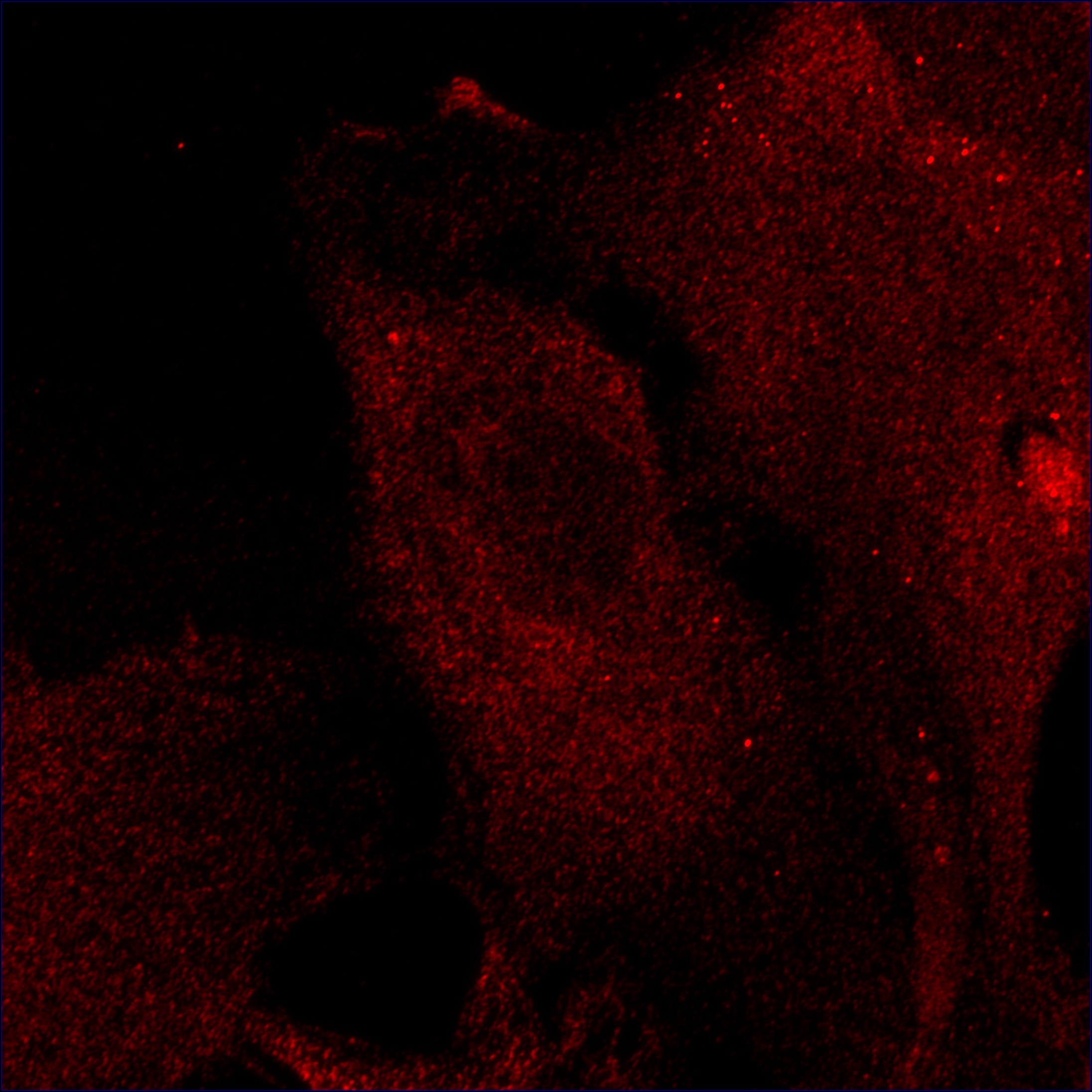

Supplement: Supplementary file 2 — Source data Fig. 1 [file 44319_2025_597_MOESM2_ESM.zip › Figure 1/1E/BICD2-flag-1-540/Flag.jpg]

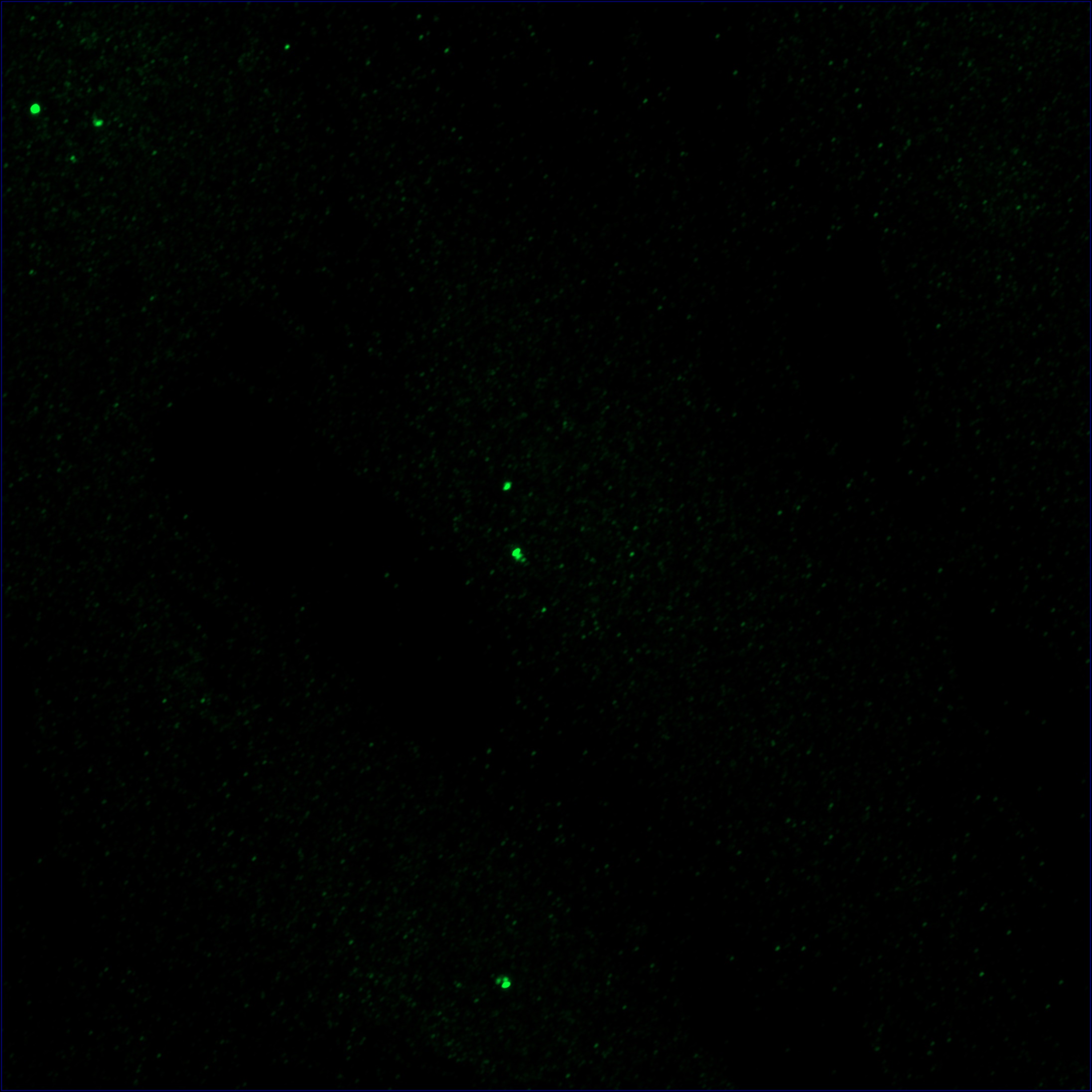

Supplement: Supplementary file 2 — Source data Fig. 1 [file 44319_2025_597_MOESM2_ESM.zip › Figure 1/1E/BICD2-flag-1-824/CEP164.jpg]

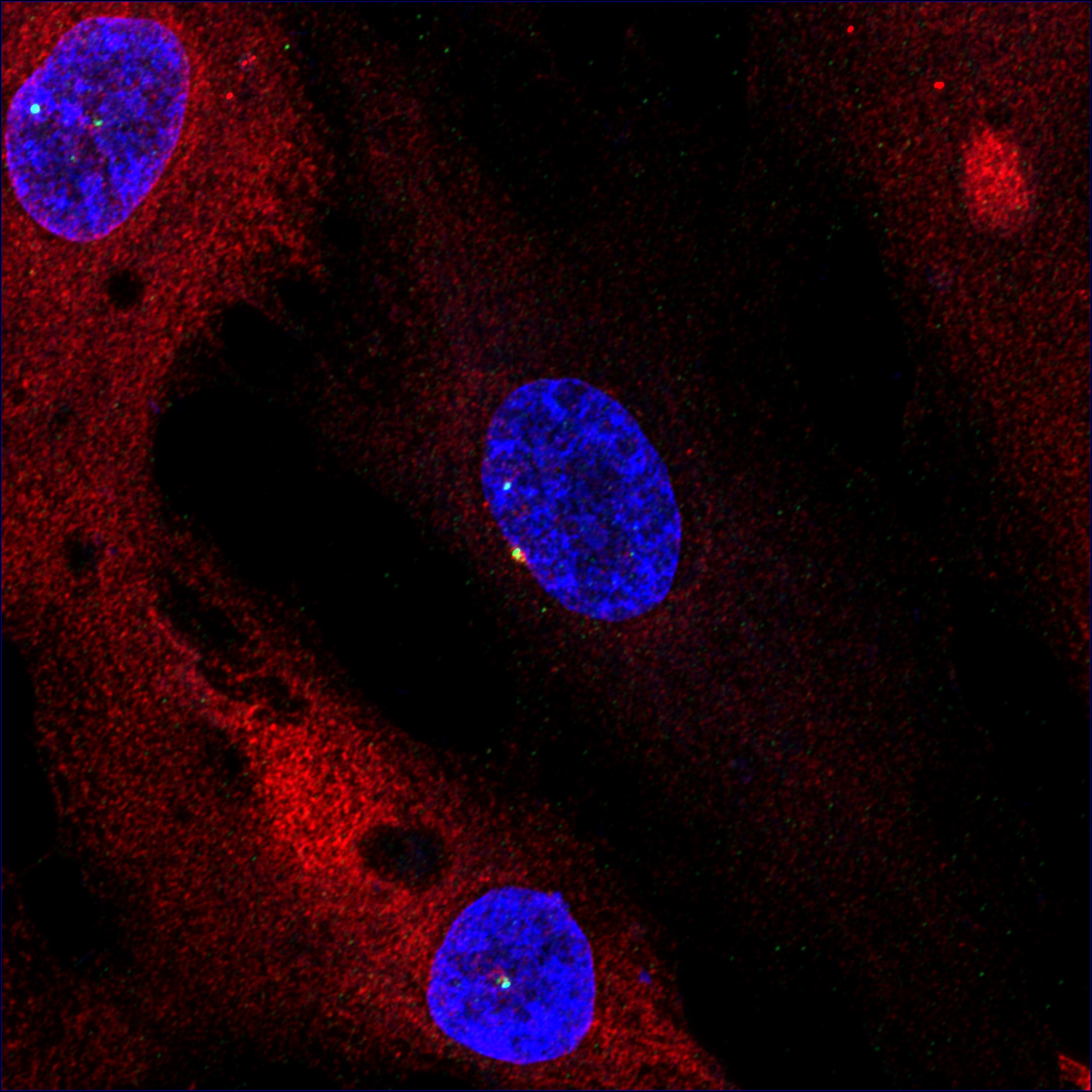

Supplement: Supplementary file 2 — Source data Fig. 1 [file 44319_2025_597_MOESM2_ESM.zip › Figure 1/1E/BICD2-flag-1-824/Flag+CEP164+DAPI.jpg]

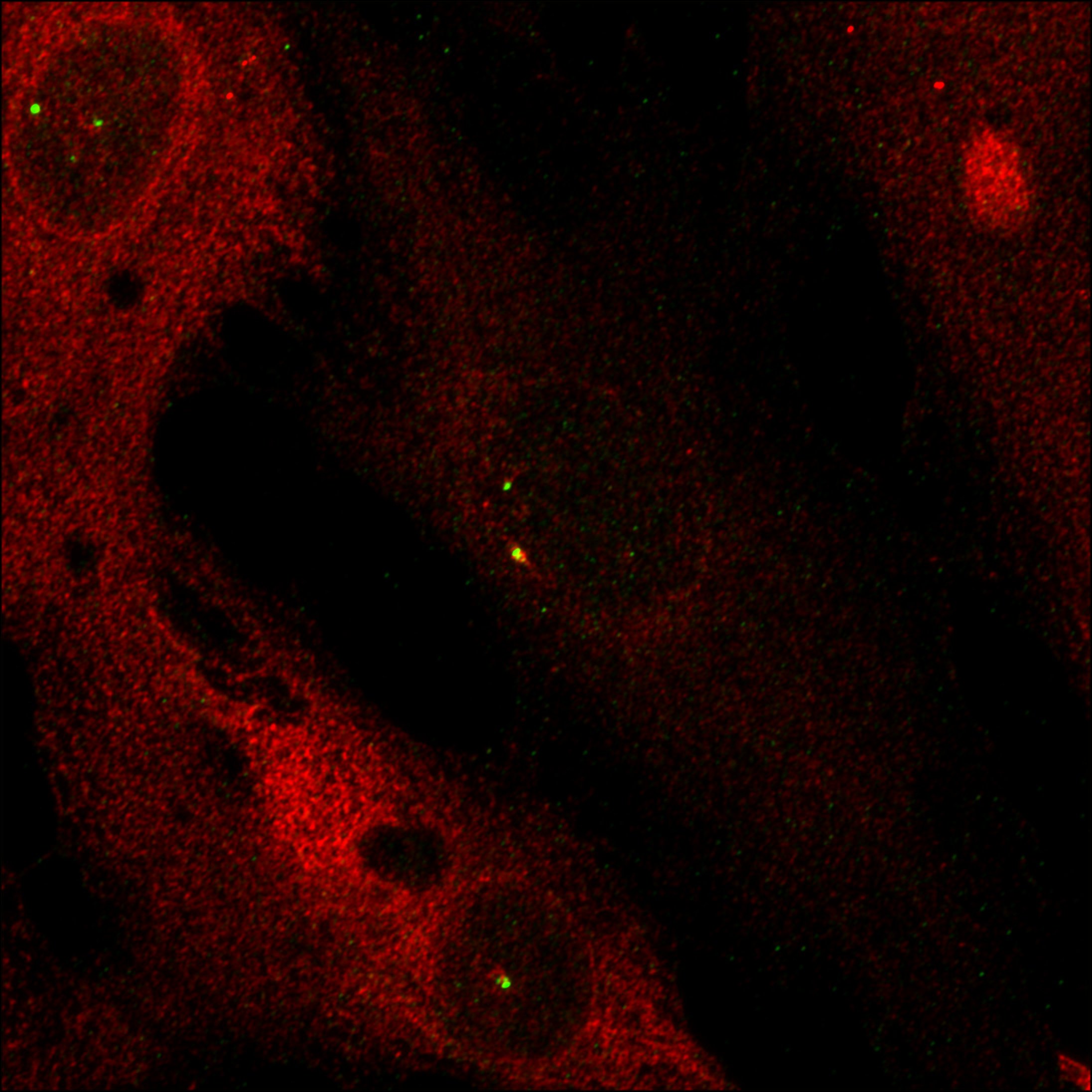

Supplement: Supplementary file 2 — Source data Fig. 1 [file 44319_2025_597_MOESM2_ESM.zip › Figure 1/1E/BICD2-flag-1-824/Flag+CEP164.jpg]

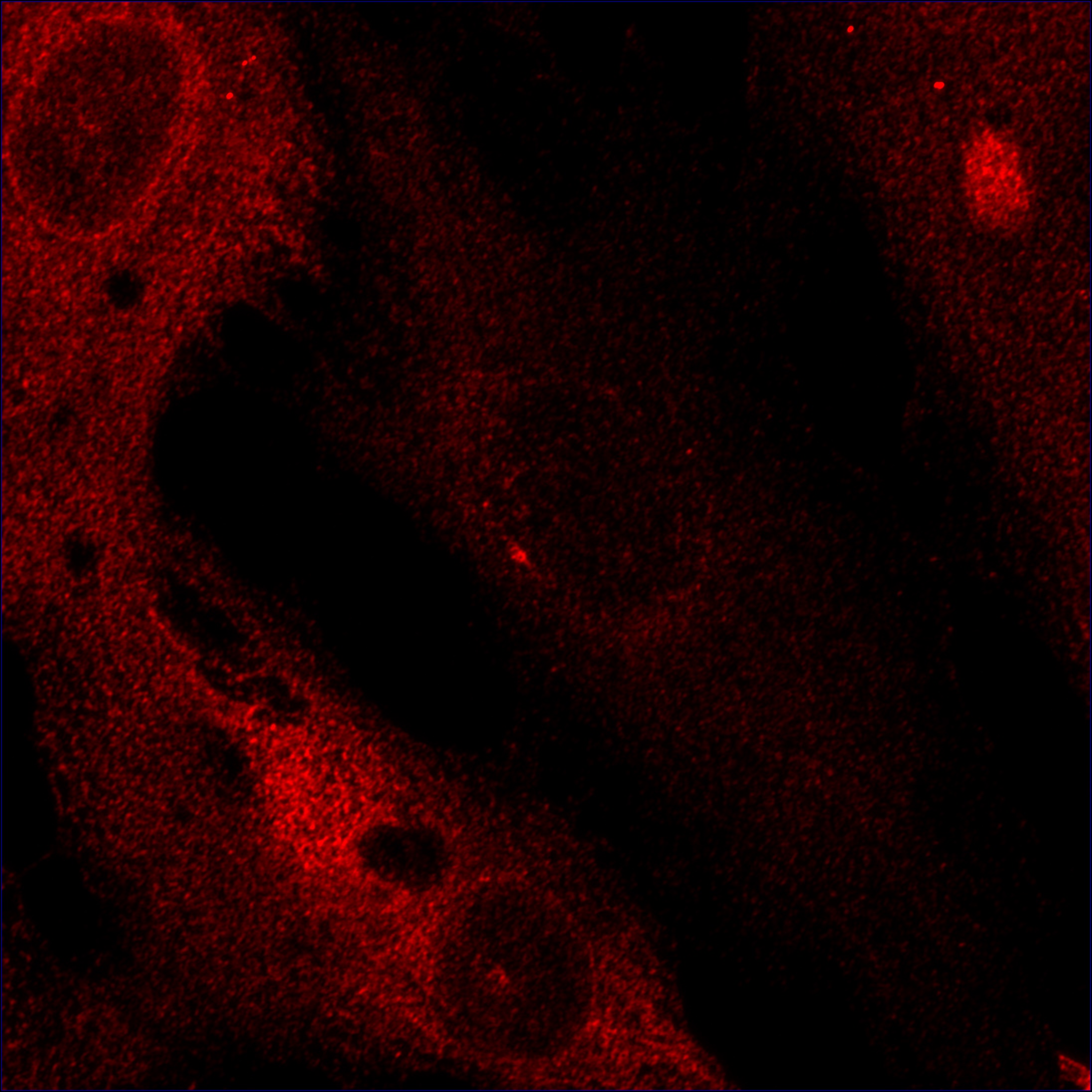

Supplement: Supplementary file 2 — Source data Fig. 1 [file 44319_2025_597_MOESM2_ESM.zip › Figure 1/1E/BICD2-flag-1-824/Flag.jpg]

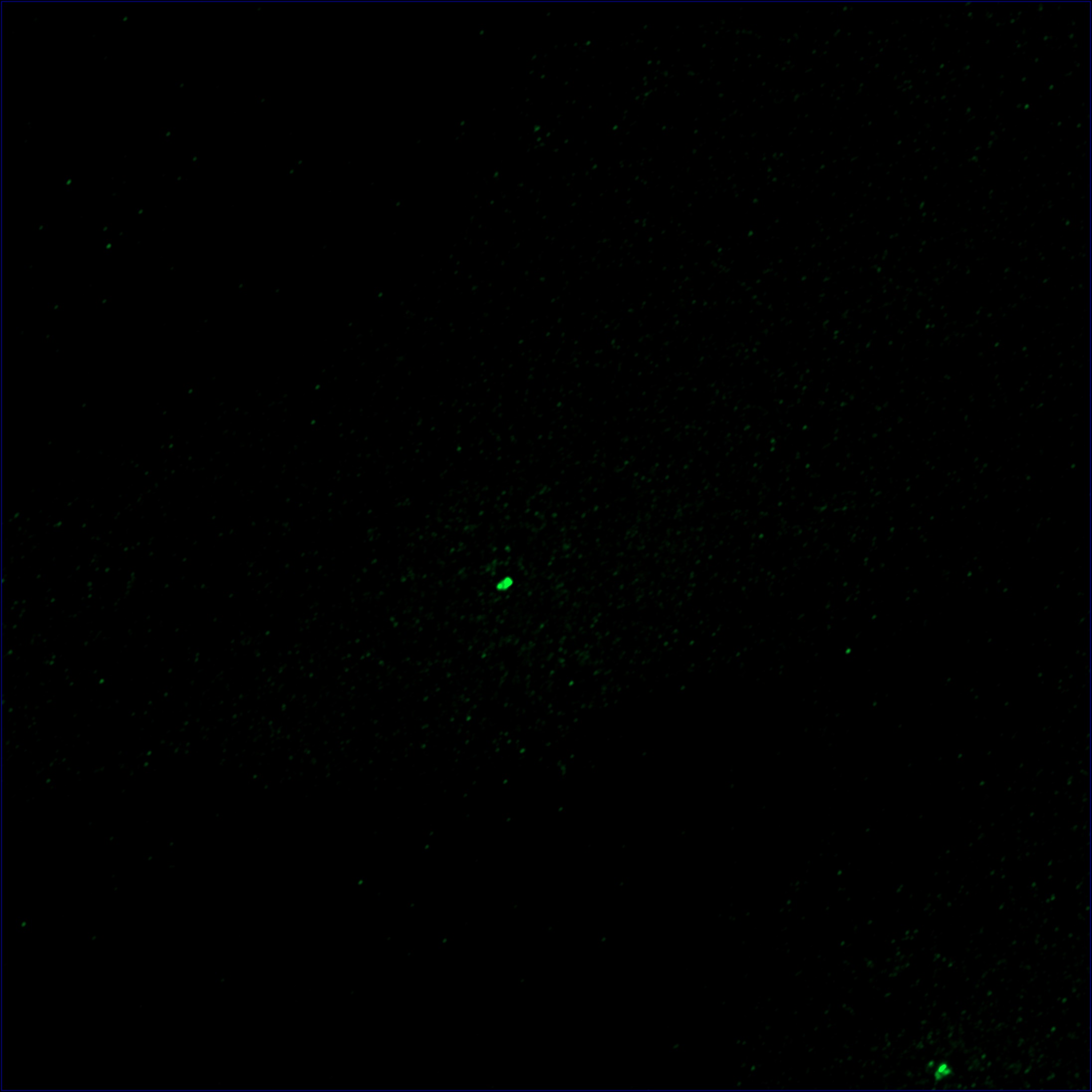

Supplement: Supplementary file 2 — Source data Fig. 1 [file 44319_2025_597_MOESM2_ESM.zip › Figure 1/1E/BICD2-flag-272-540/CEP164.jpg]

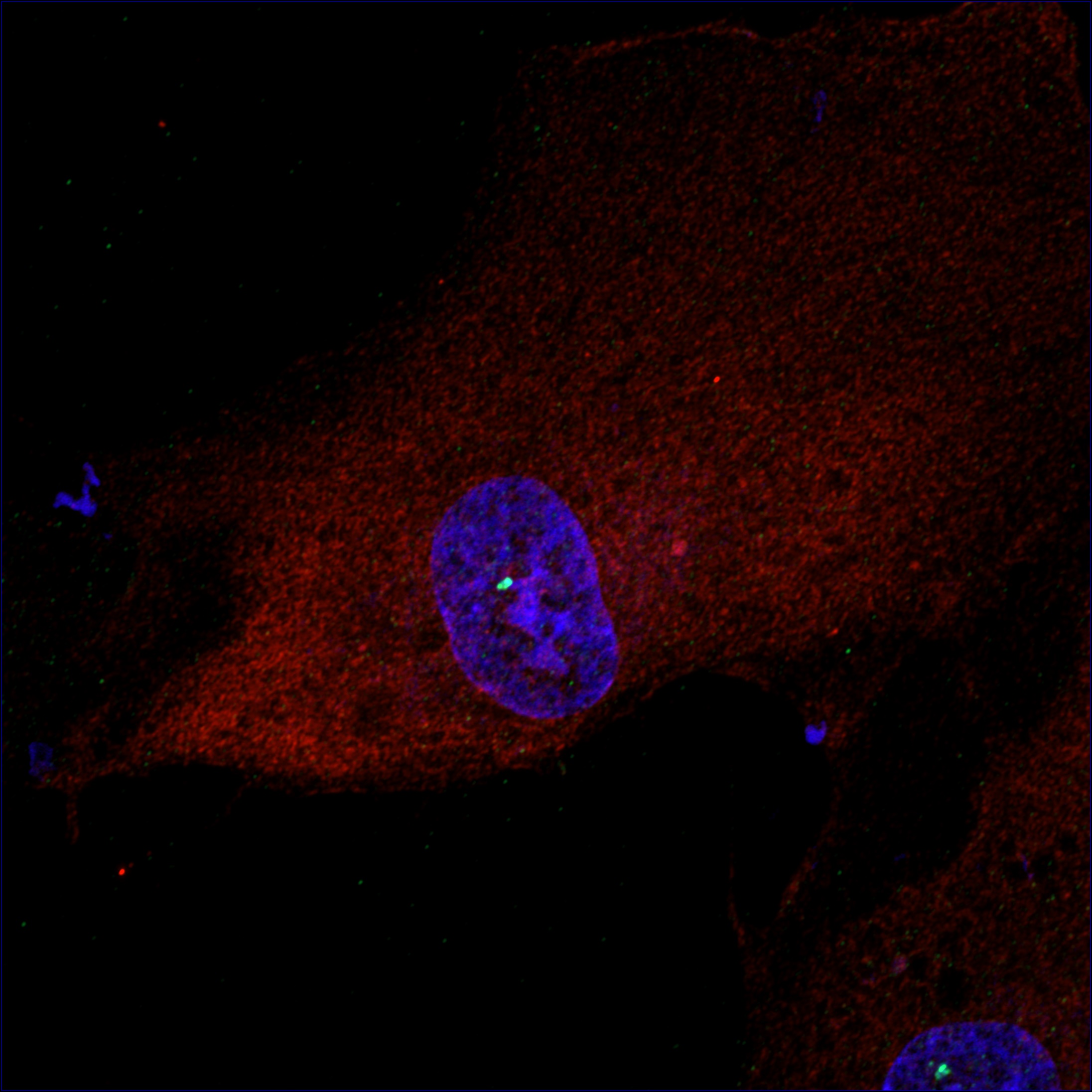

Supplement: Supplementary file 2 — Source data Fig. 1 [file 44319_2025_597_MOESM2_ESM.zip › Figure 1/1E/BICD2-flag-272-540/Flag+CEP164+DAPI.jpg]

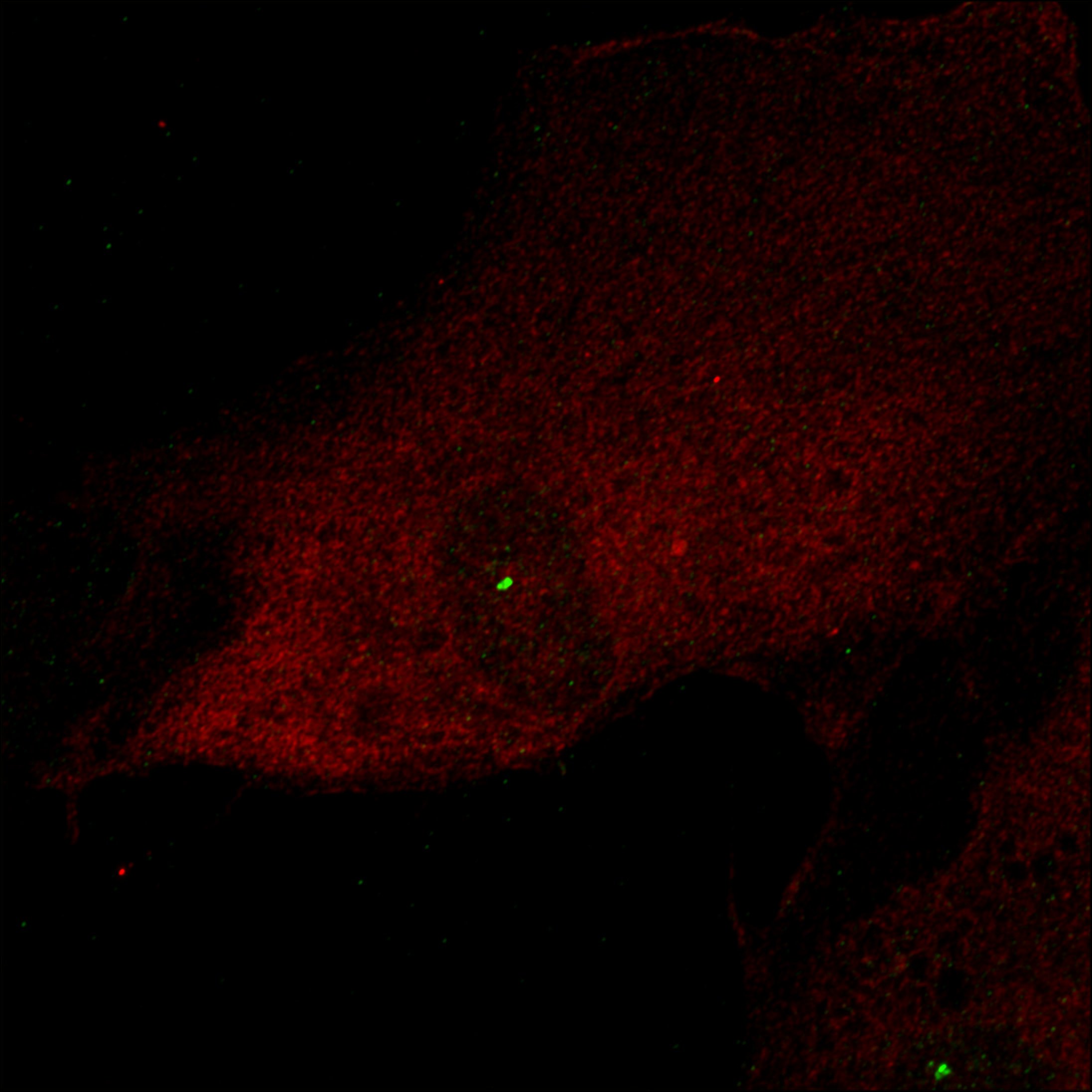

Supplement: Supplementary file 2 — Source data Fig. 1 [file 44319_2025_597_MOESM2_ESM.zip › Figure 1/1E/BICD2-flag-272-540/Flag+CEP164.jpg]

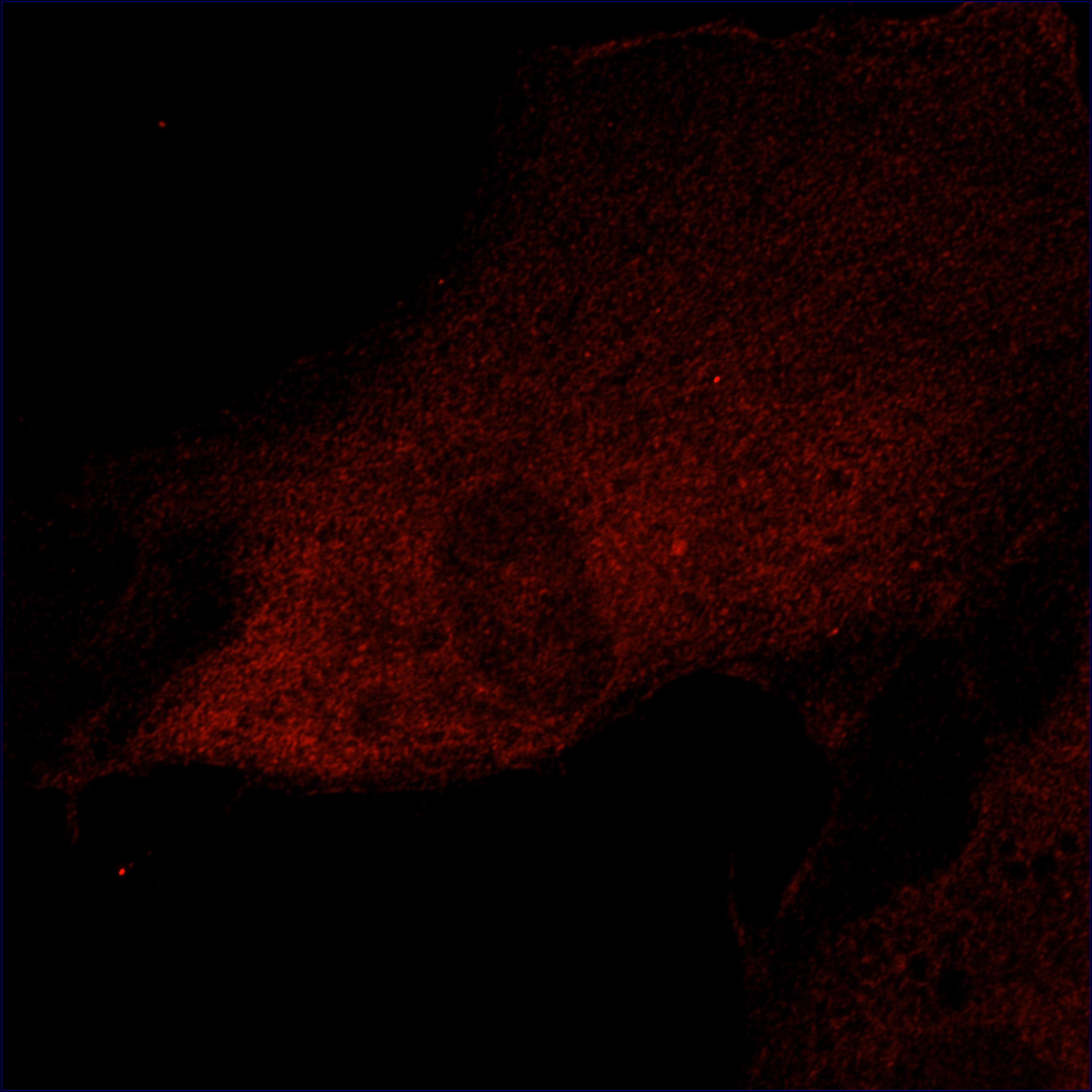

Supplement: Supplementary file 2 — Source data Fig. 1 [file 44319_2025_597_MOESM2_ESM.zip › Figure 1/1E/BICD2-flag-272-540/Flag.jpg]

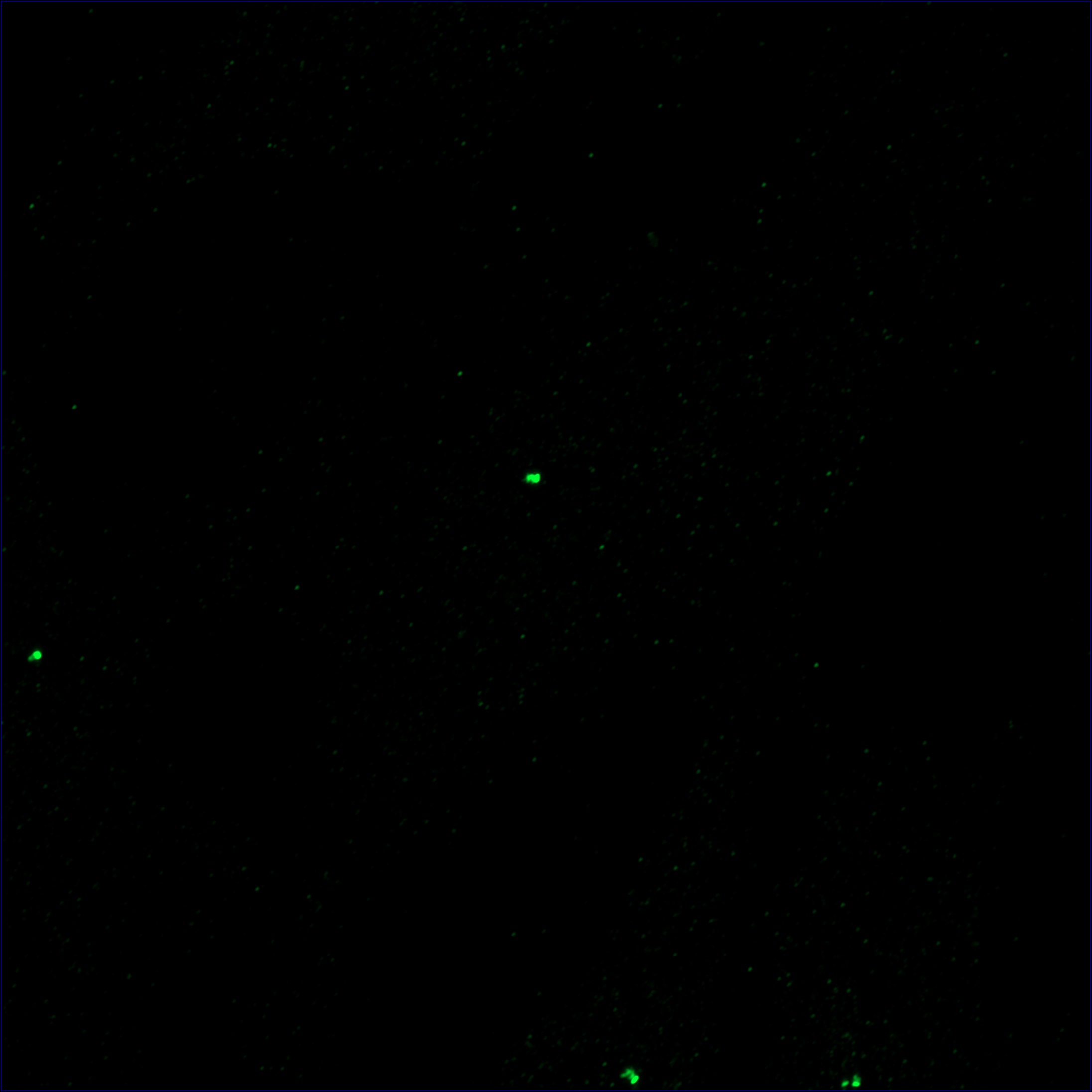

Supplement: Supplementary file 2 — Source data Fig. 1 [file 44319_2025_597_MOESM2_ESM.zip › Figure 1/1E/BICD2-flag-272-824/CEP164.jpg]

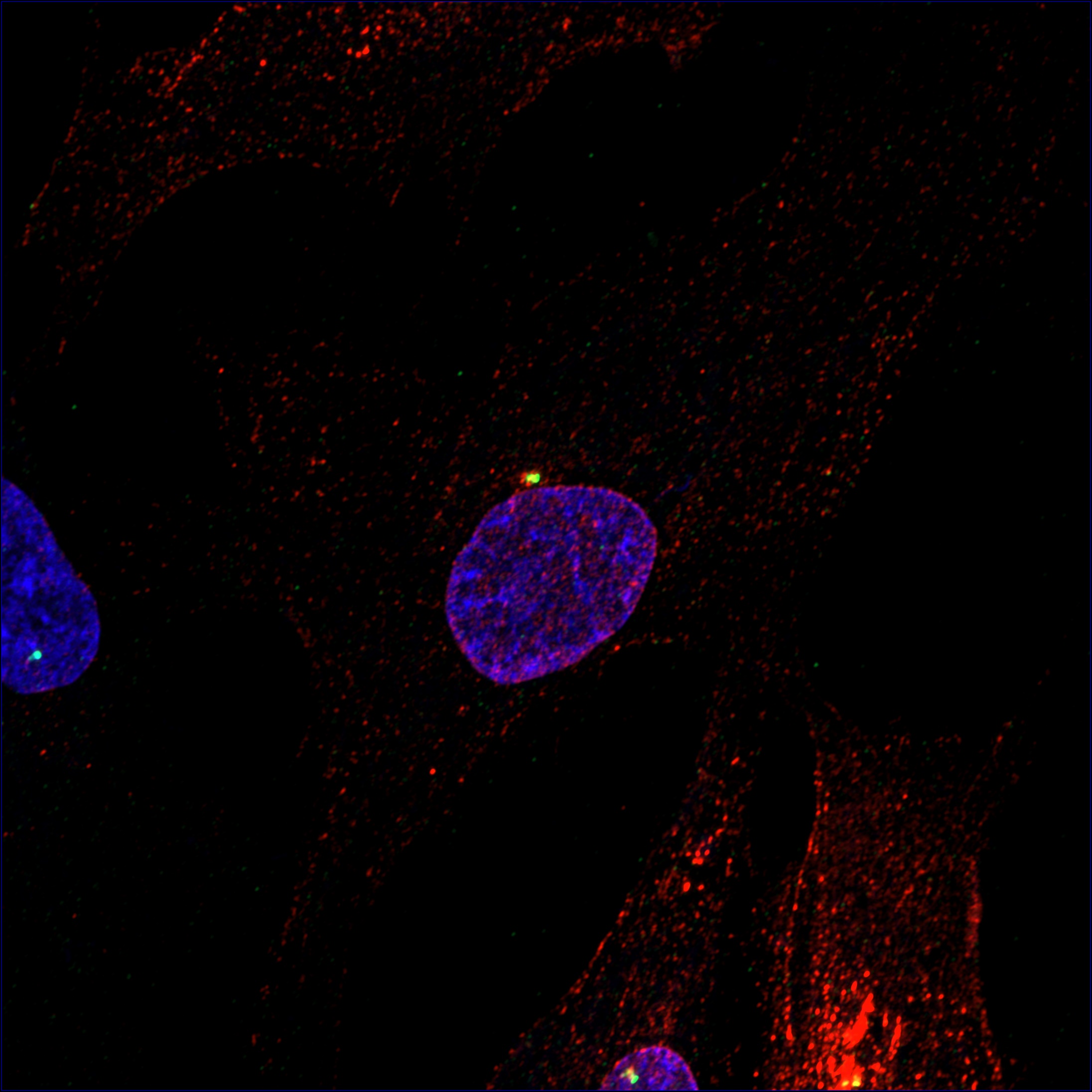

Supplement: Supplementary file 2 — Source data Fig. 1 [file 44319_2025_597_MOESM2_ESM.zip › Figure 1/1E/BICD2-flag-272-824/Flag+CEP164+DAPI.jpg]

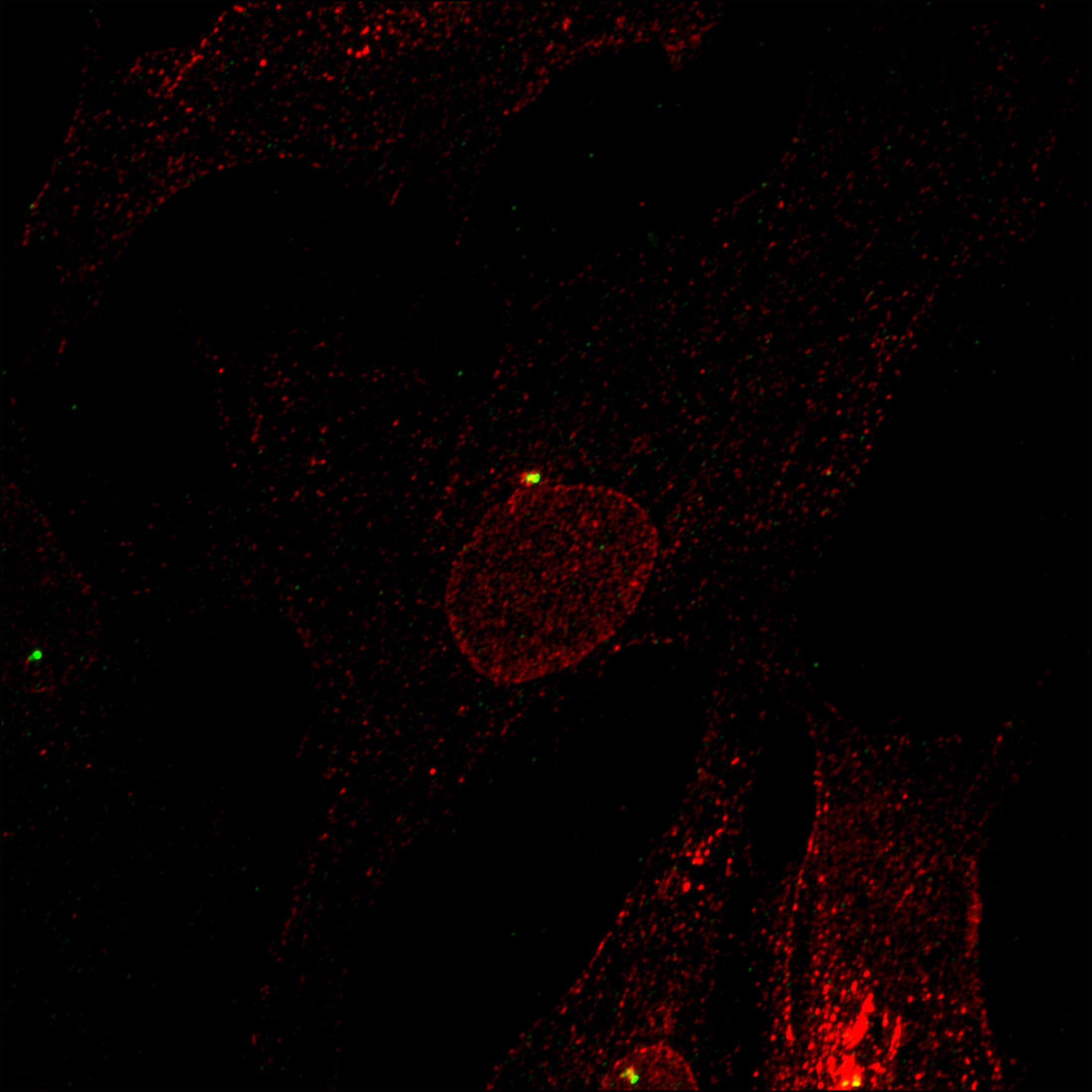

Supplement: Supplementary file 2 — Source data Fig. 1 [file 44319_2025_597_MOESM2_ESM.zip › Figure 1/1E/BICD2-flag-272-824/Flag+CEP164.jpg]

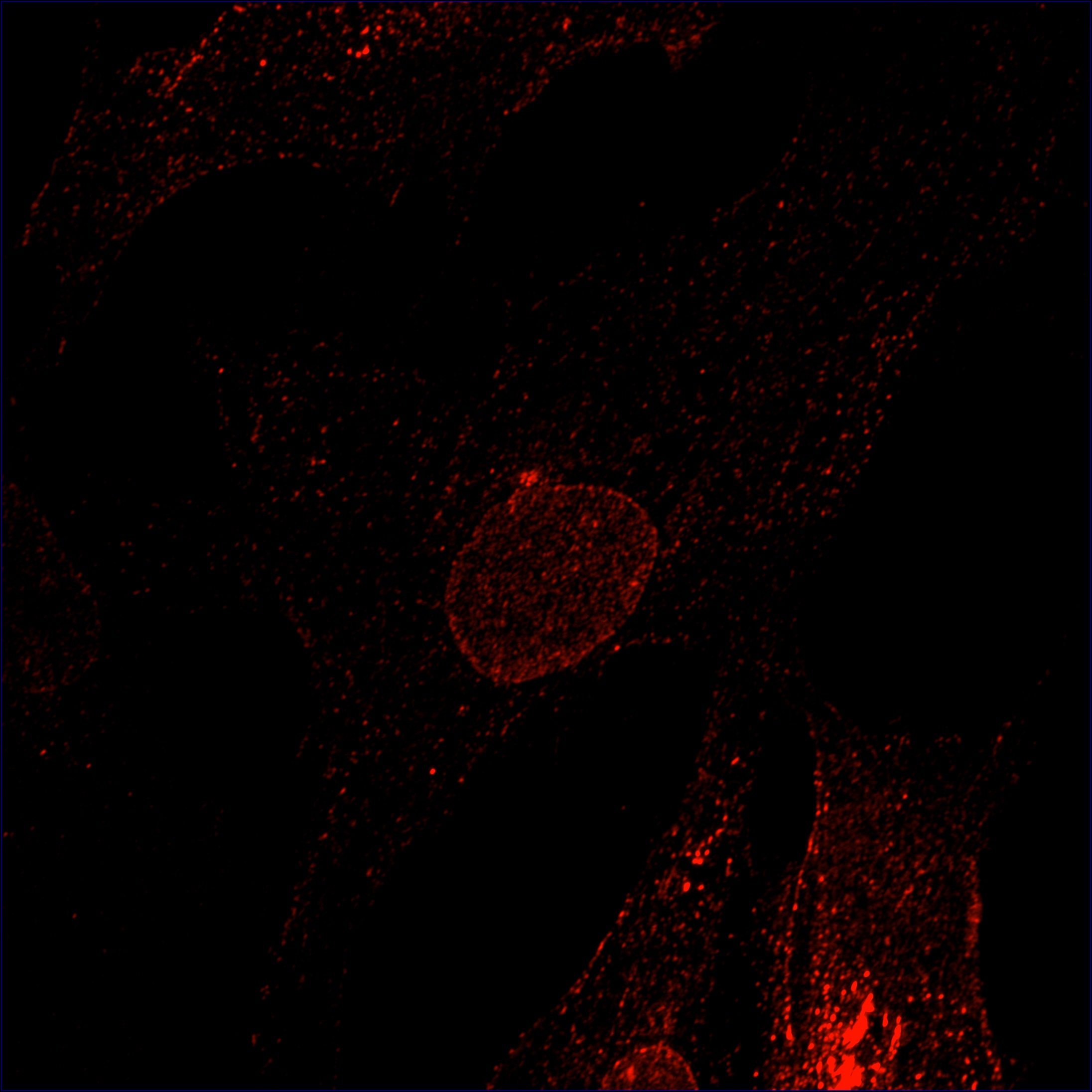

Supplement: Supplementary file 2 — Source data Fig. 1 [file 44319_2025_597_MOESM2_ESM.zip › Figure 1/1E/BICD2-flag-272-824/Flag.jpg]

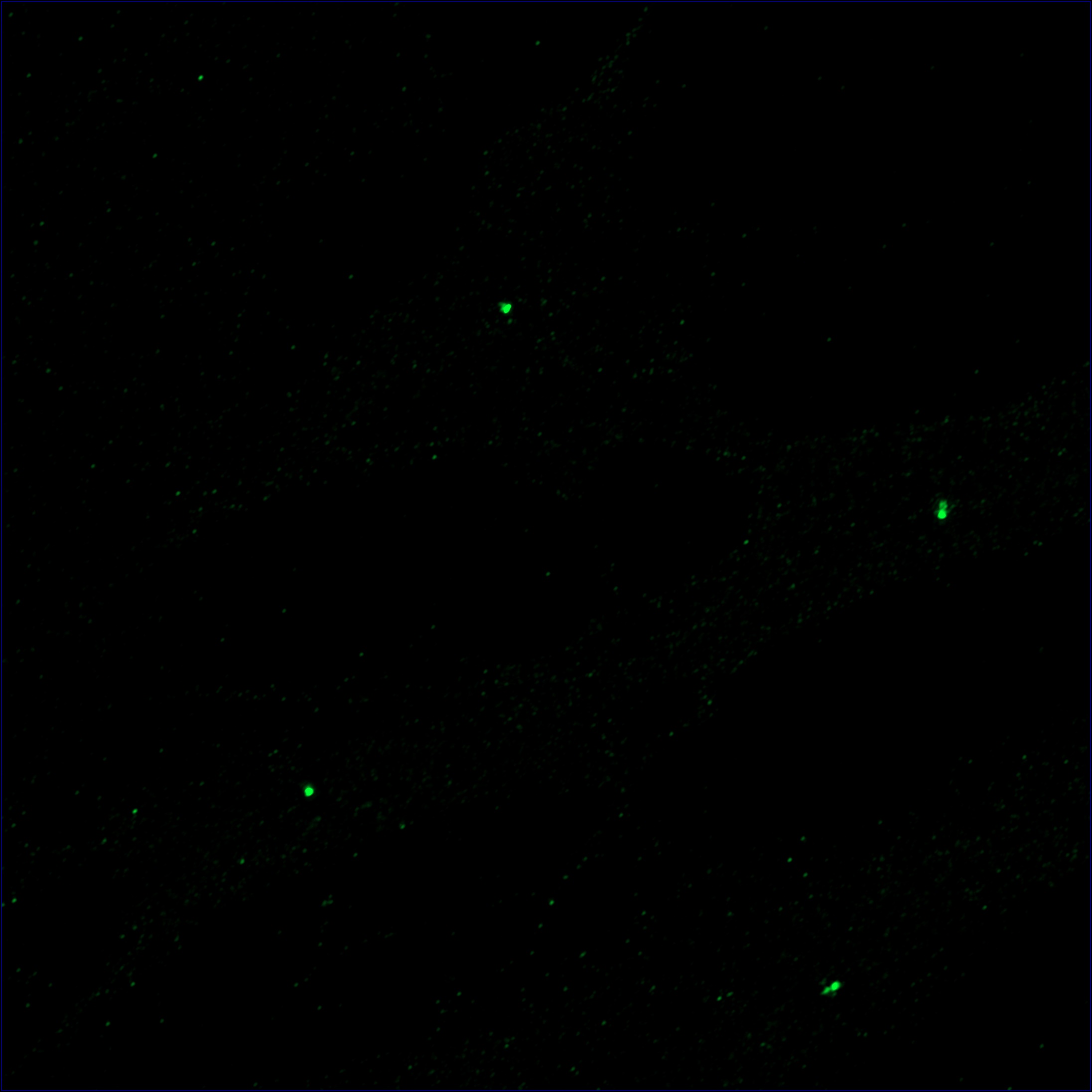

Supplement: Supplementary file 2 — Source data Fig. 1 [file 44319_2025_597_MOESM2_ESM.zip › Figure 1/1E/BICD2-flag-541-824/CEP164.jpg]

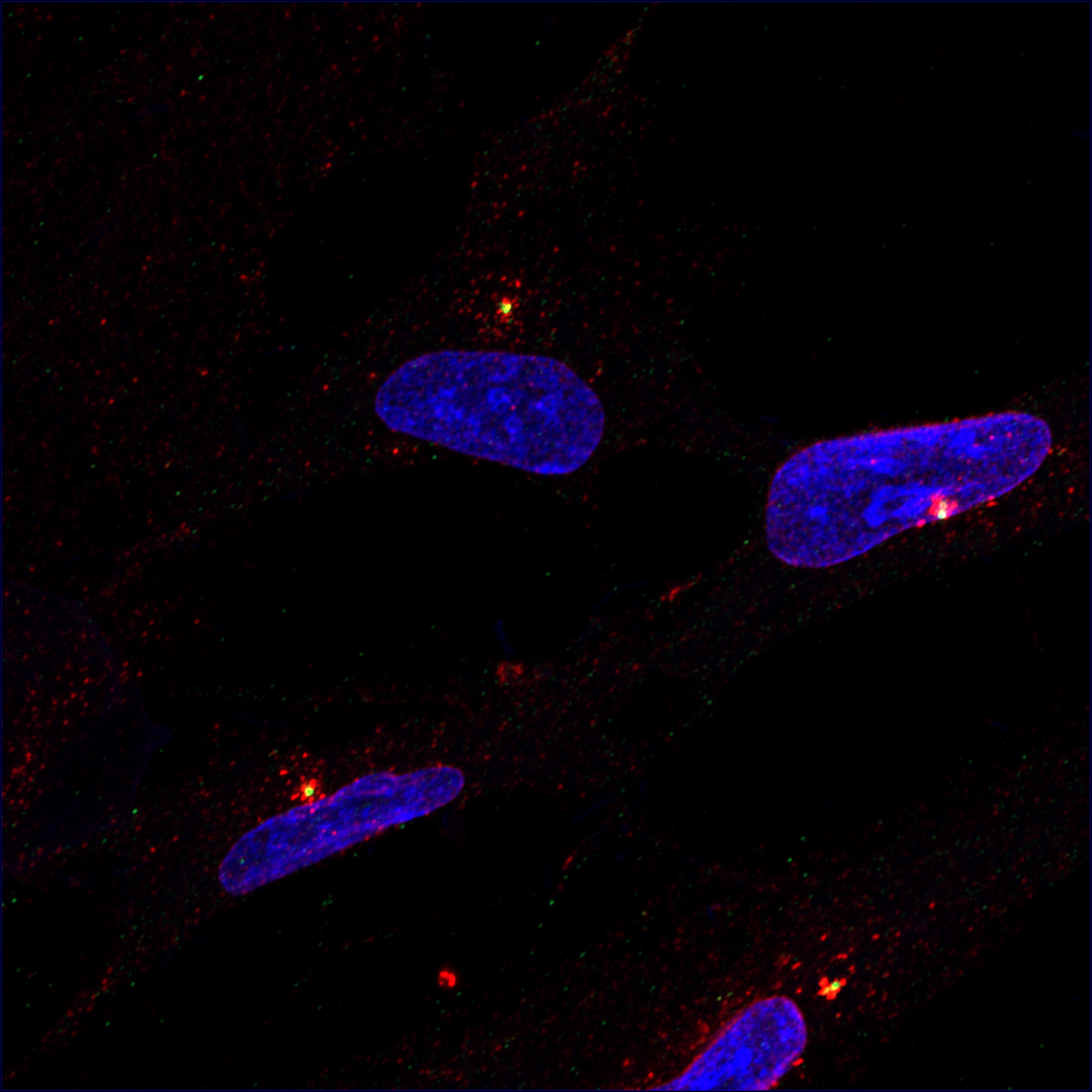

Supplement: Supplementary file 2 — Source data Fig. 1 [file 44319_2025_597_MOESM2_ESM.zip › Figure 1/1E/BICD2-flag-541-824/Flag+CEP164+DAPI.jpg]

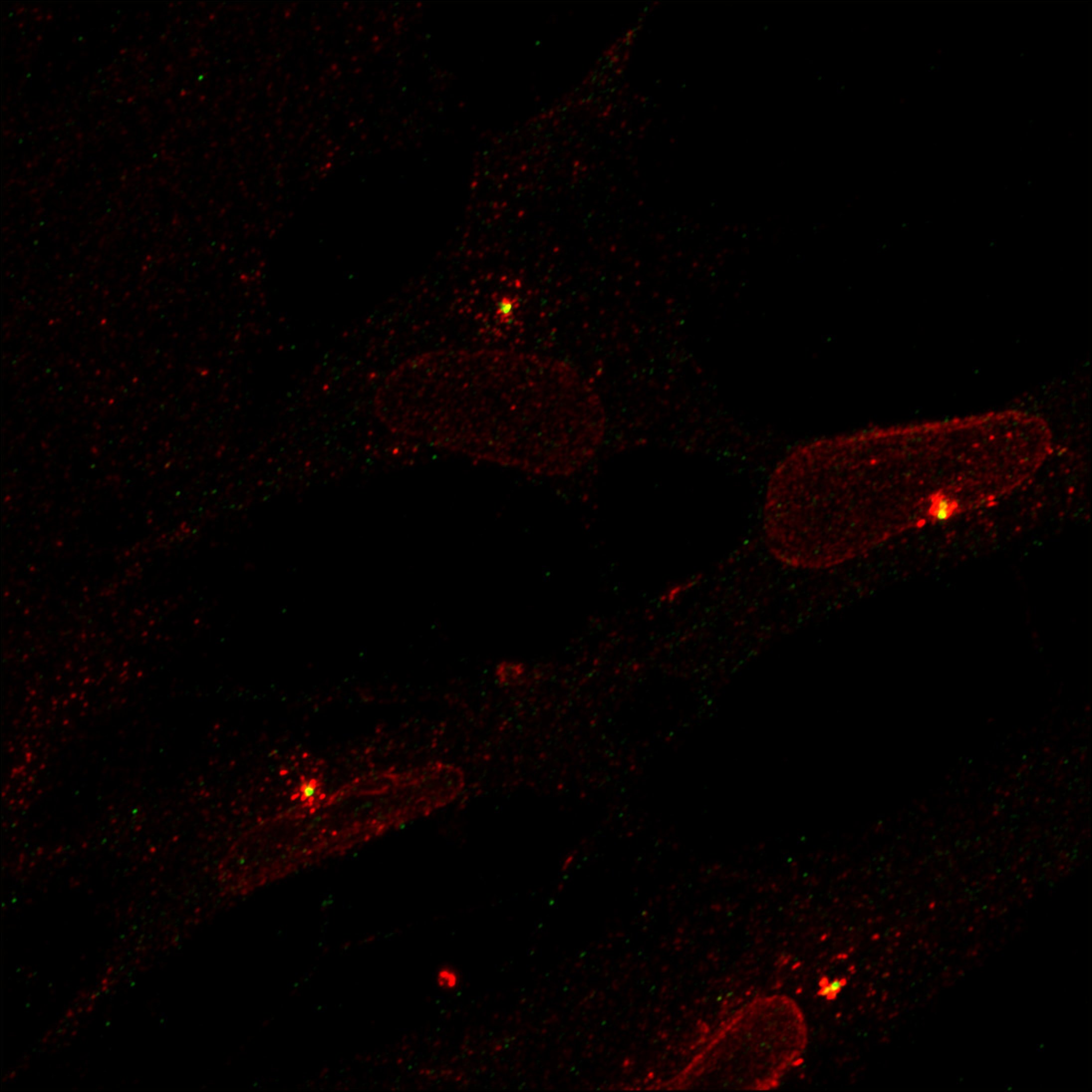

Supplement: Supplementary file 2 — Source data Fig. 1 [file 44319_2025_597_MOESM2_ESM.zip › Figure 1/1E/BICD2-flag-541-824/Flag+CEP164.jpg]

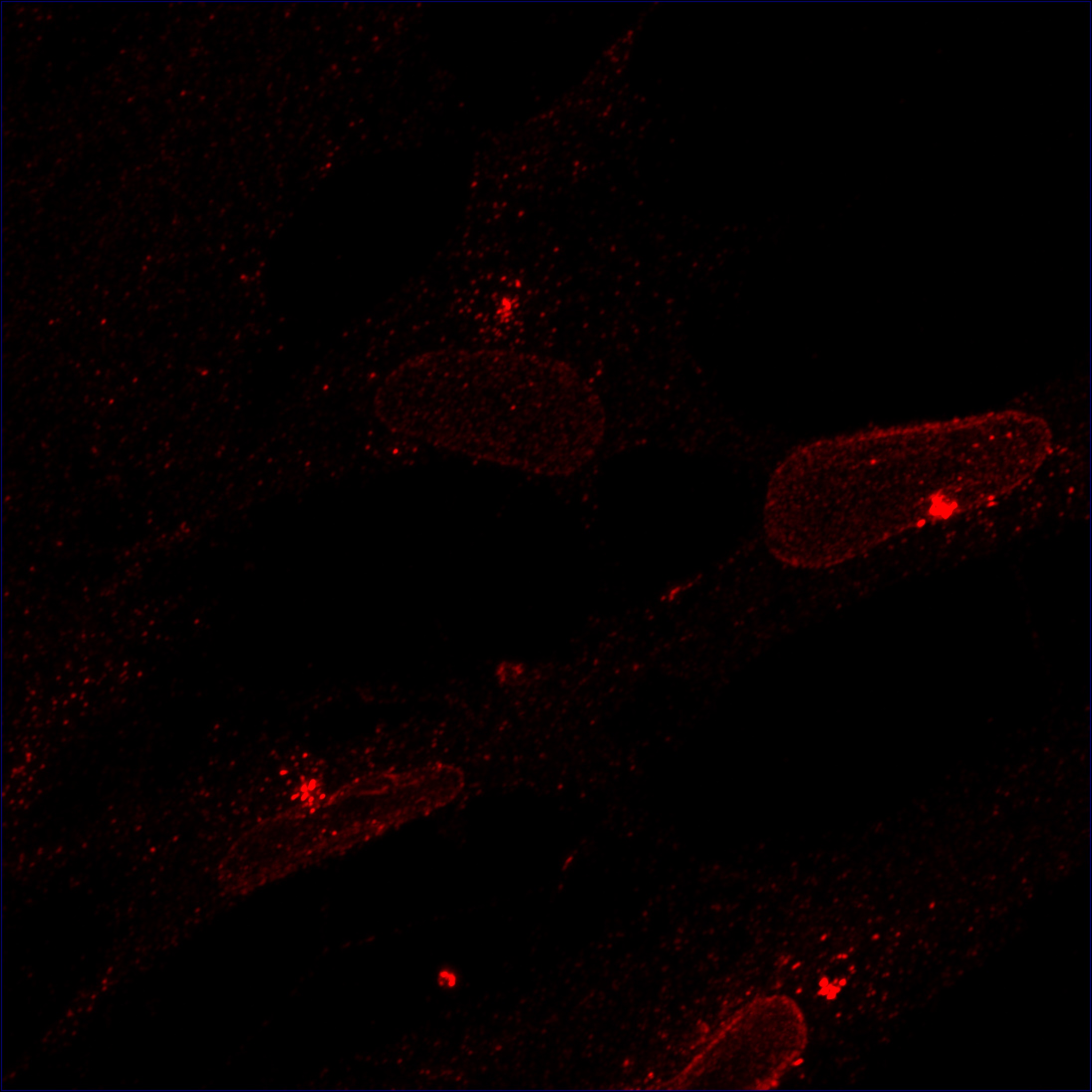

Supplement: Supplementary file 2 — Source data Fig. 1 [file 44319_2025_597_MOESM2_ESM.zip › Figure 1/1E/BICD2-flag-541-824/Flag.jpg]

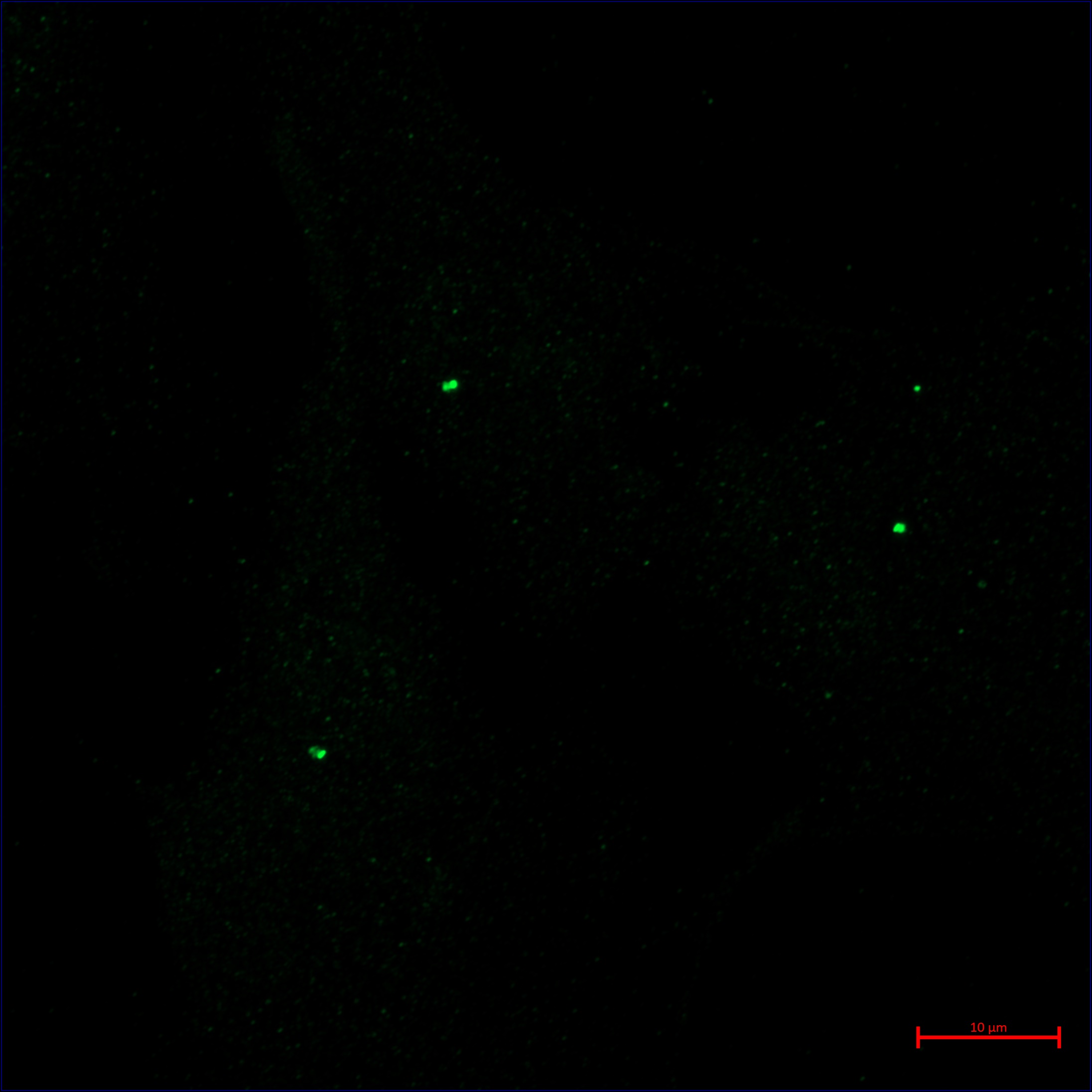

Supplement: Supplementary file 2 — Source data Fig. 1 [file 44319_2025_597_MOESM2_ESM.zip › Figure 1/1E/control/CEP164.jpg]

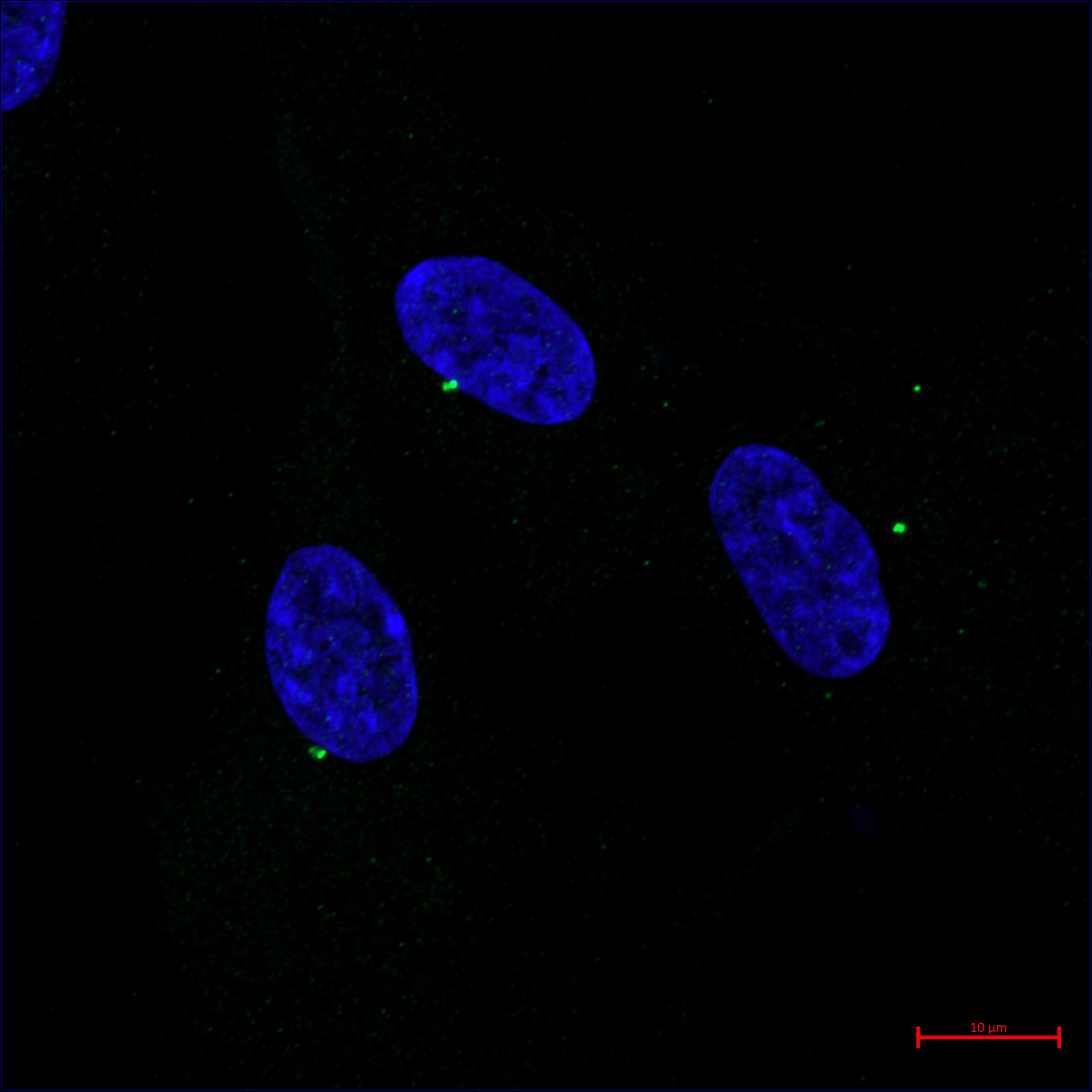

Supplement: Supplementary file 2 — Source data Fig. 1 [file 44319_2025_597_MOESM2_ESM.zip › Figure 1/1E/control/Flag+CEP164+DAPI.jpg]

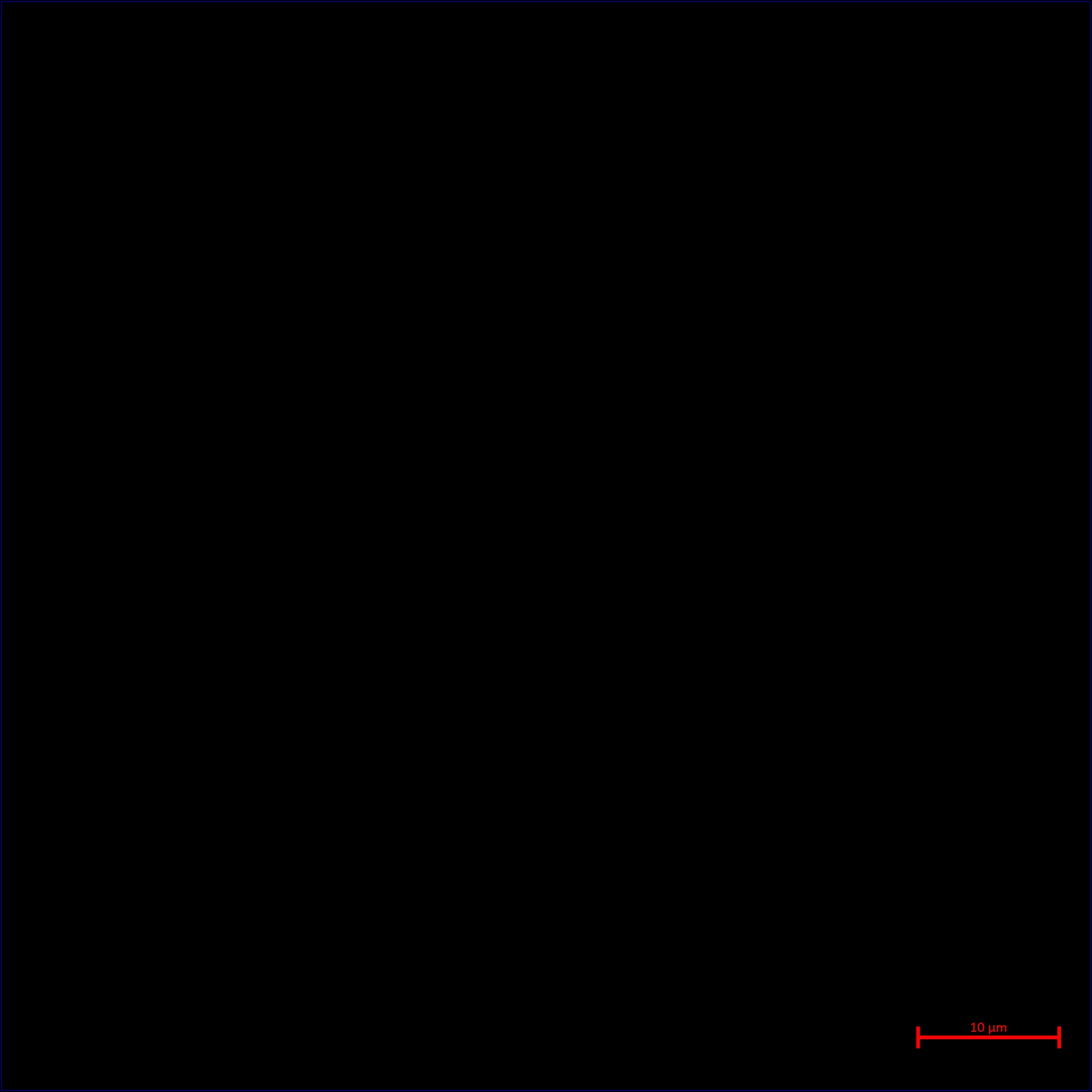

Supplement: Supplementary file 2 — Source data Fig. 1 [file 44319_2025_597_MOESM2_ESM.zip › Figure 1/1E/control/Flag.jpg]

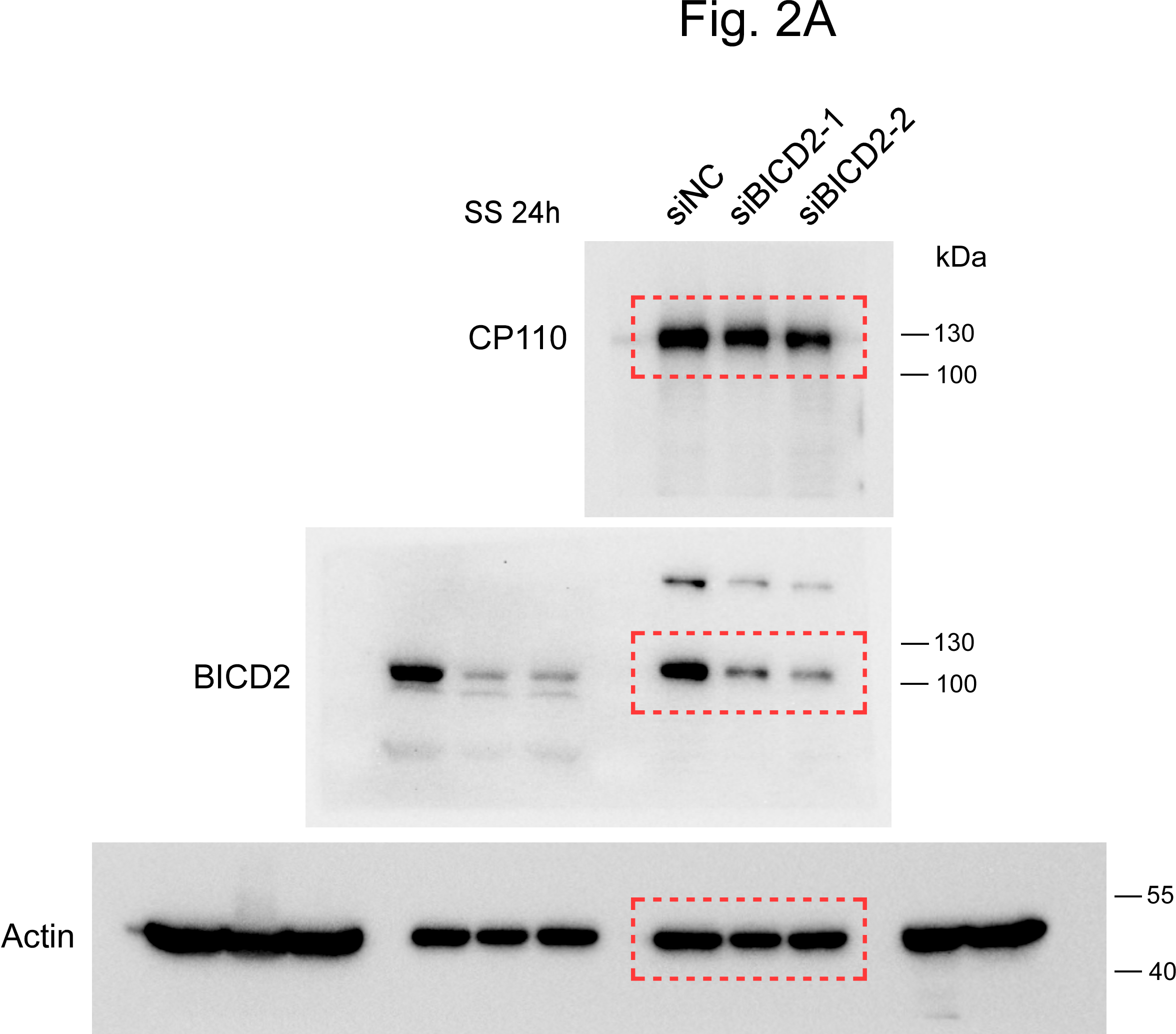

Supplement: Supplementary file 3 — Source data Fig. 2 [file 44319_2025_597_MOESM3_ESM.zip › Figure 2/2A.tif]

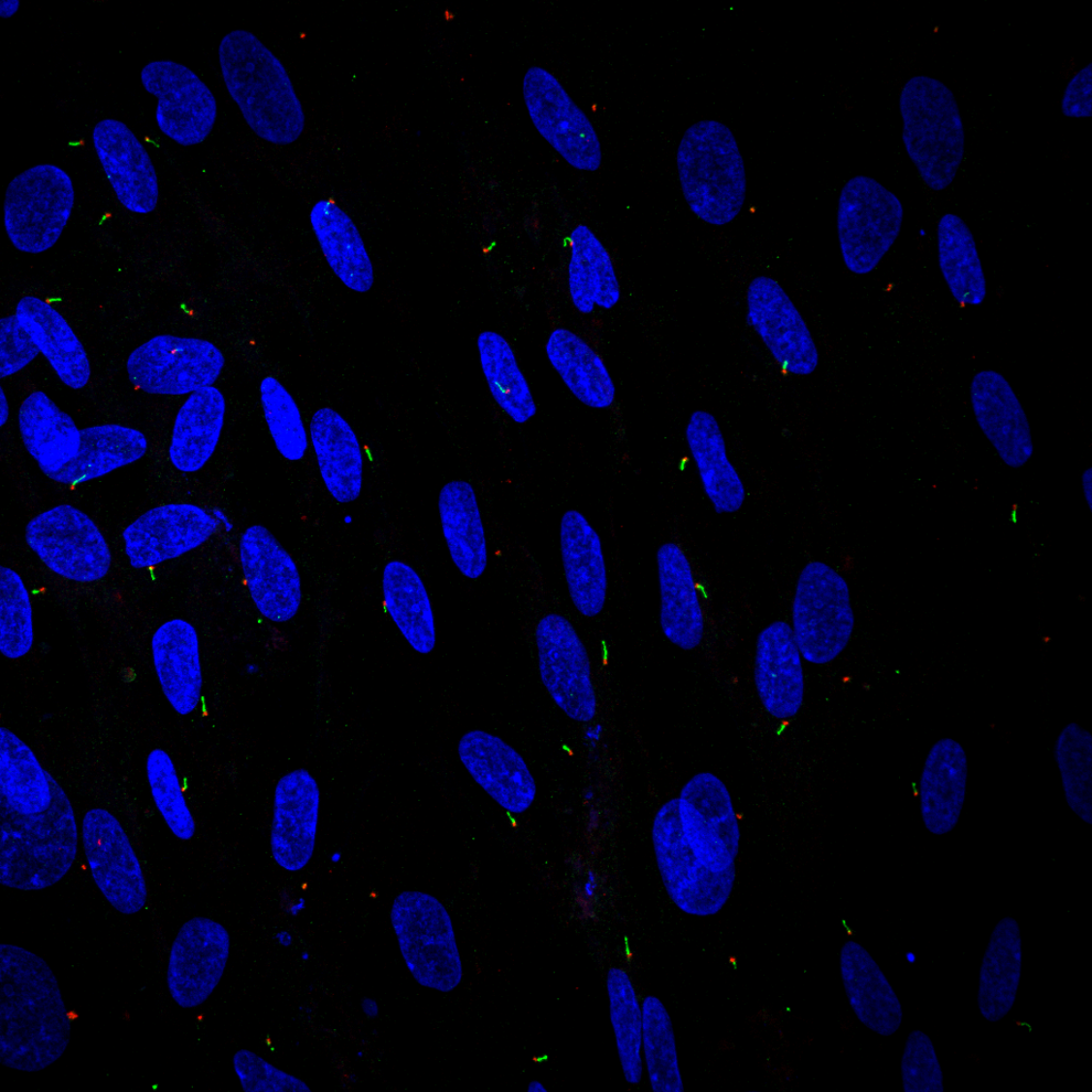

Supplement: Supplementary file 3 — Source data Fig. 2 [file 44319_2025_597_MOESM3_ESM.zip › Figure 2/2B/siBICD2-1.bmp]

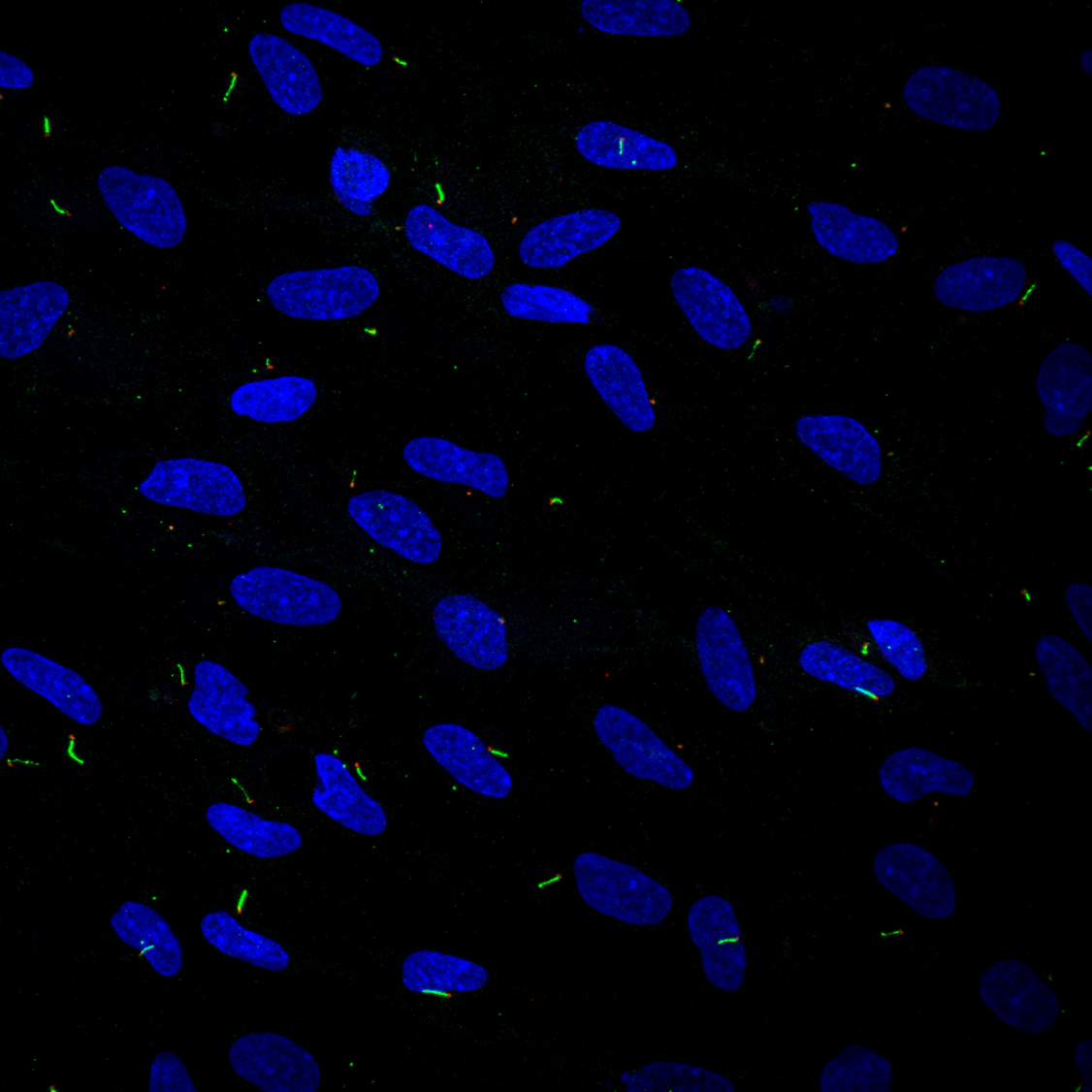

Supplement: Supplementary file 3 — Source data Fig. 2 [file 44319_2025_597_MOESM3_ESM.zip › Figure 2/2B/siBICD2-2.bmp]

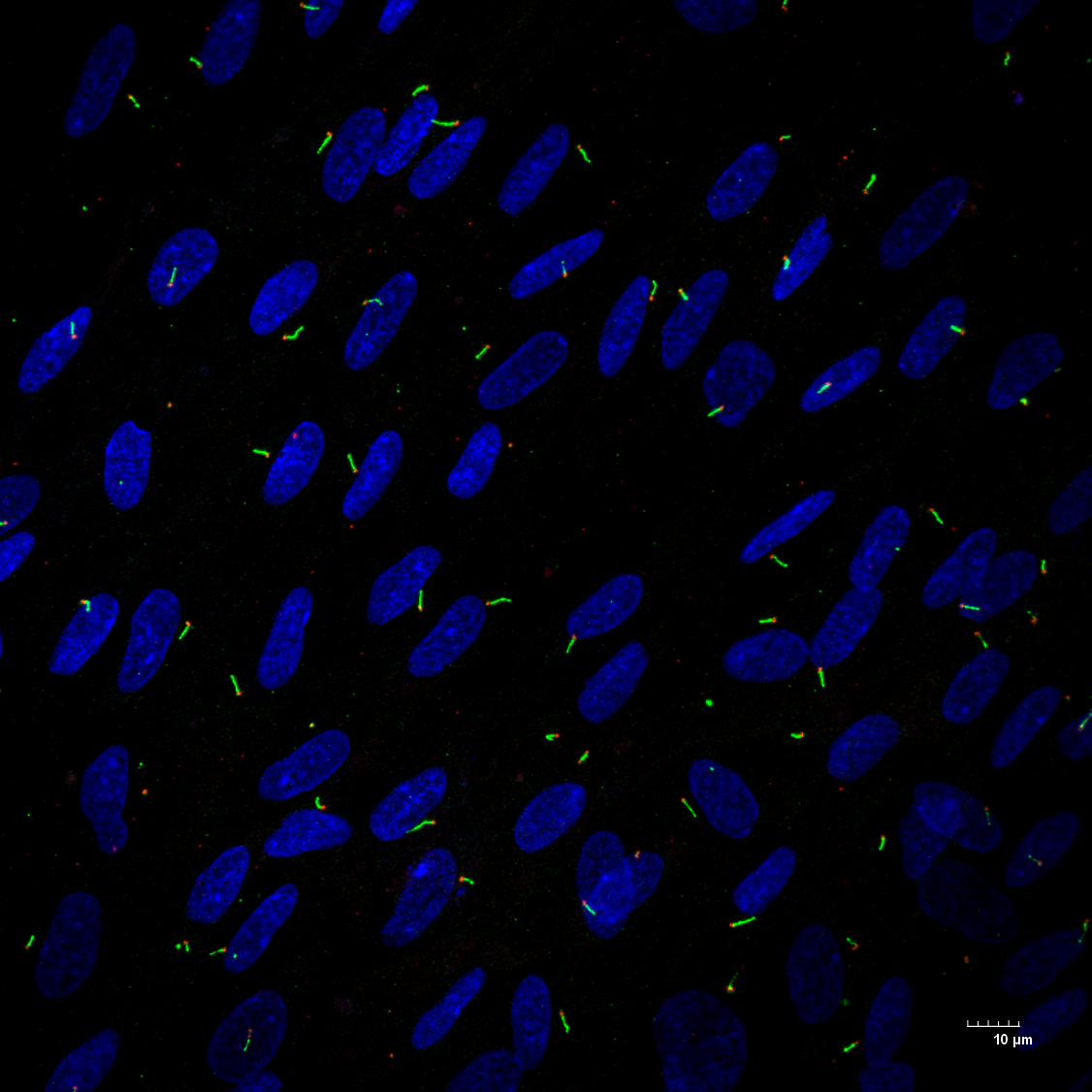

Supplement: Supplementary file 3 — Source data Fig. 2 [file 44319_2025_597_MOESM3_ESM.zip › Figure 2/2B/siNC.bmp]

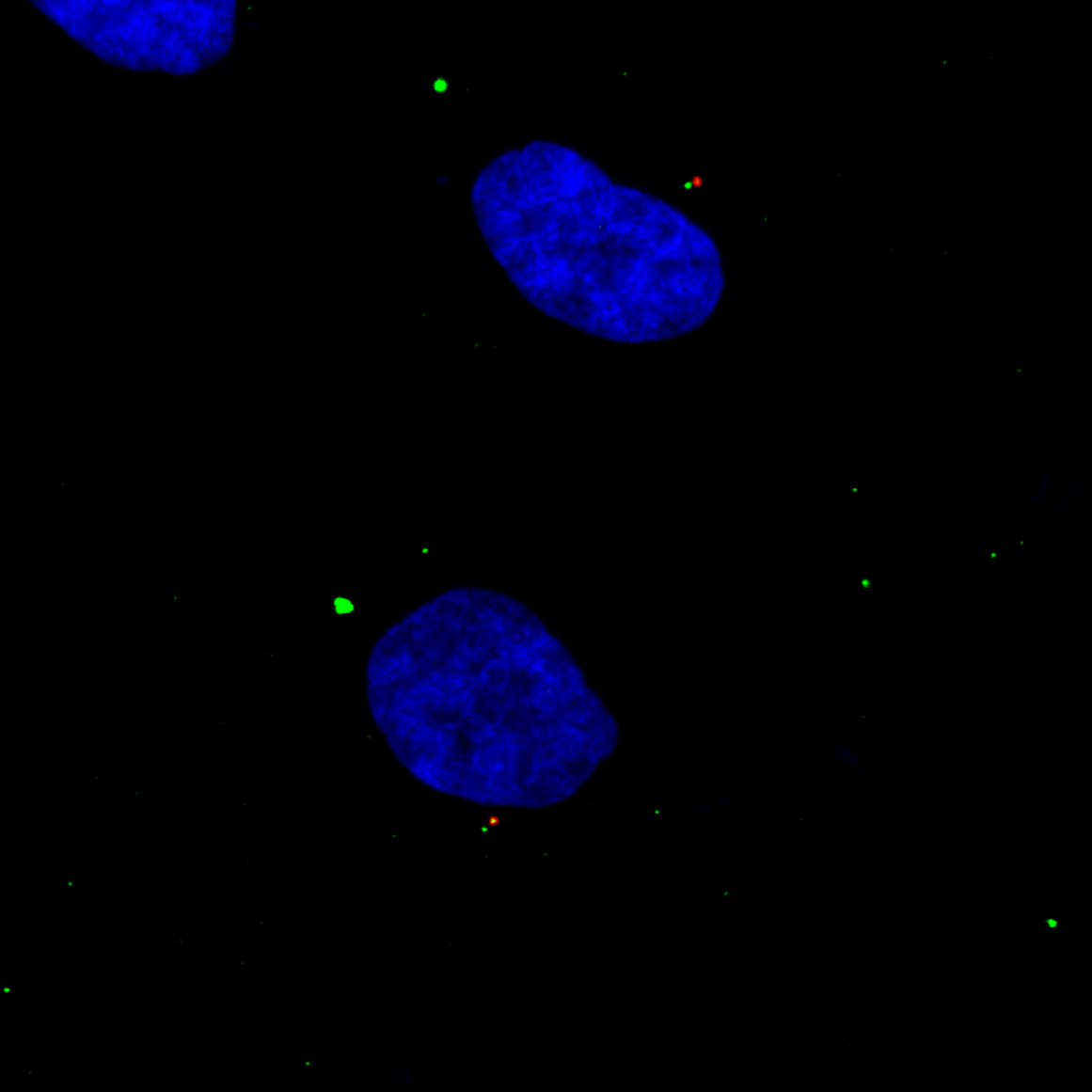

Supplement: Supplementary file 3 — Source data Fig. 2 [file 44319_2025_597_MOESM3_ESM.zip › Figure 2/2D/siBICD2-1.bmp]

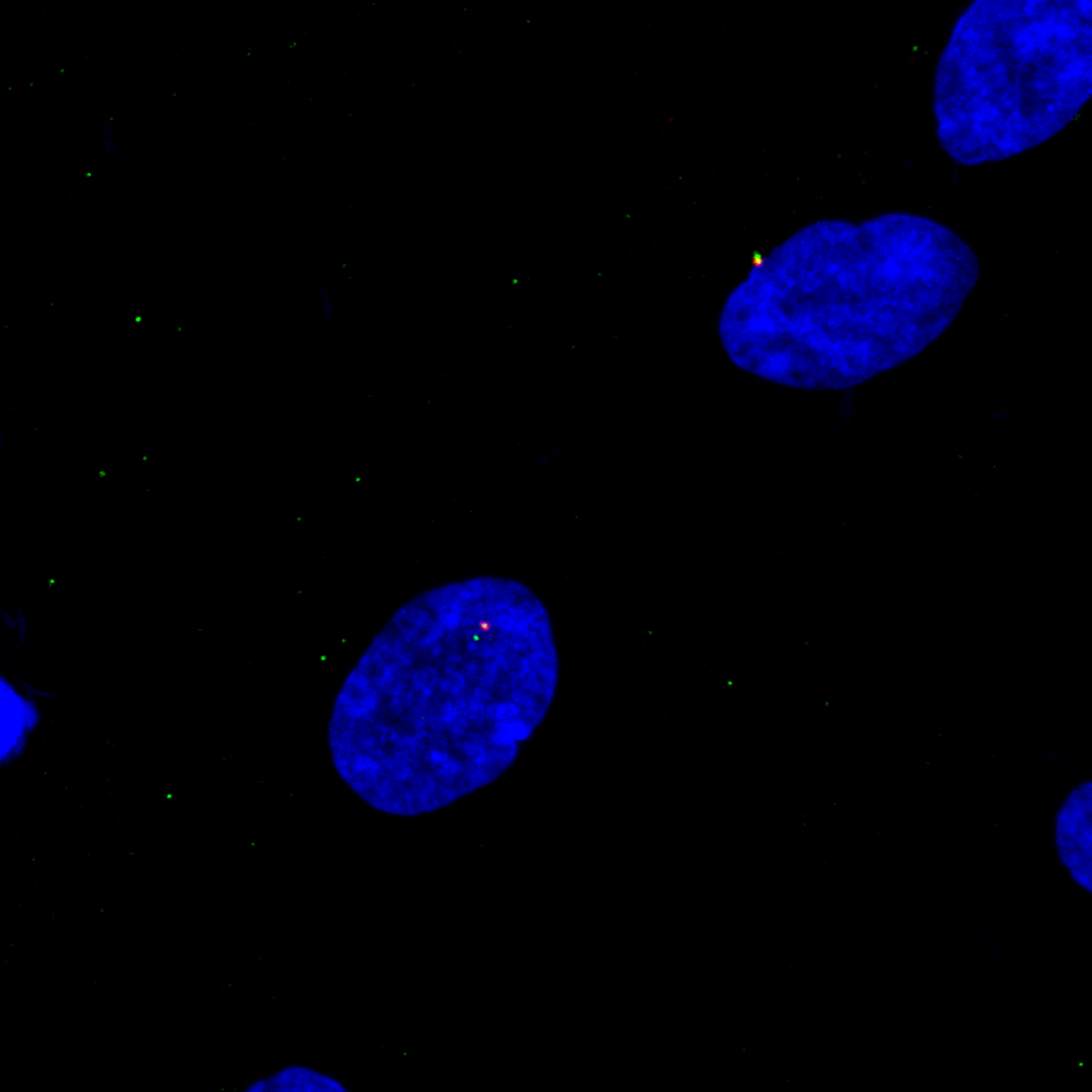

Supplement: Supplementary file 3 — Source data Fig. 2 [file 44319_2025_597_MOESM3_ESM.zip › Figure 2/2D/siBICD2-2.bmp]

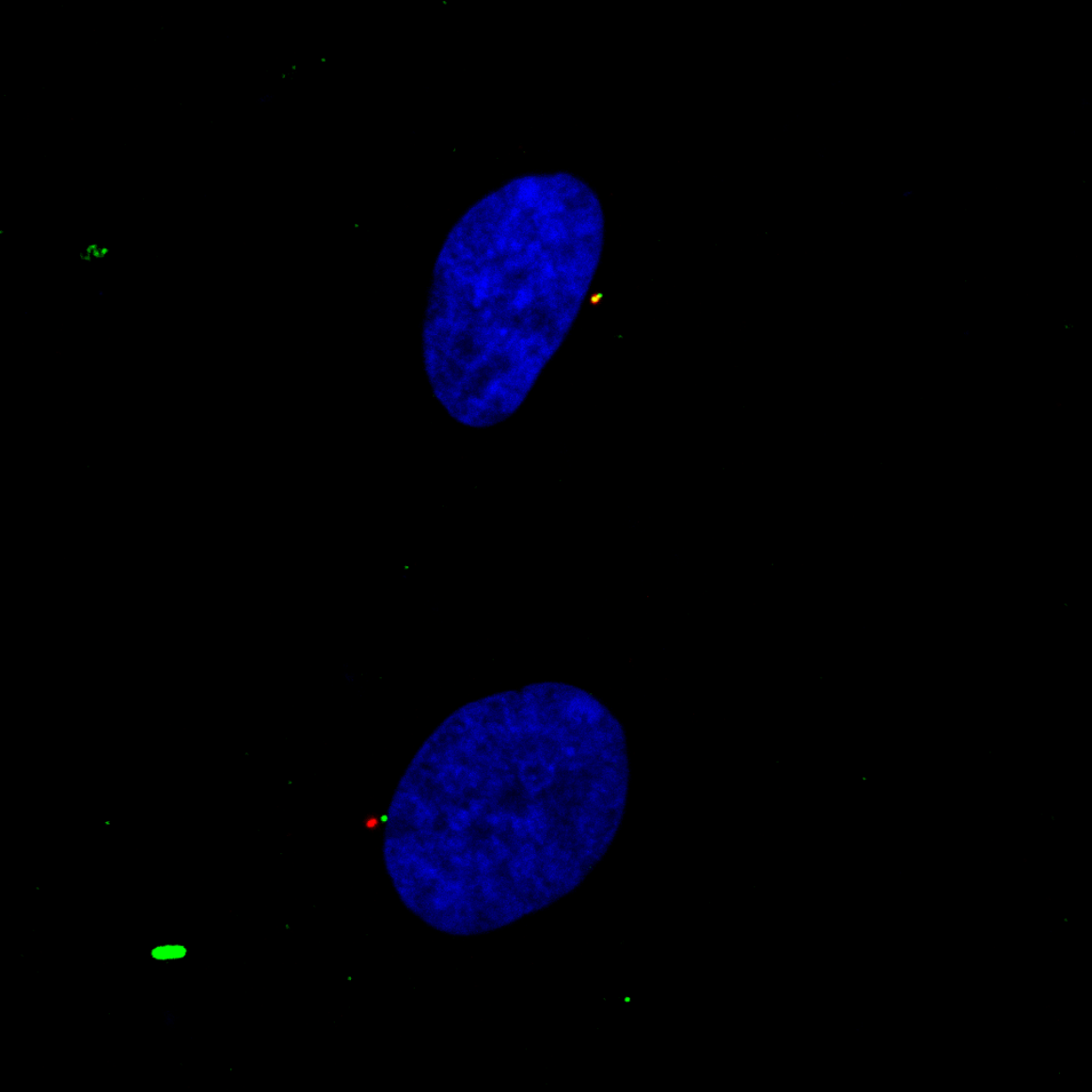

Supplement: Supplementary file 3 — Source data Fig. 2 [file 44319_2025_597_MOESM3_ESM.zip › Figure 2/2D/siNC.bmp]

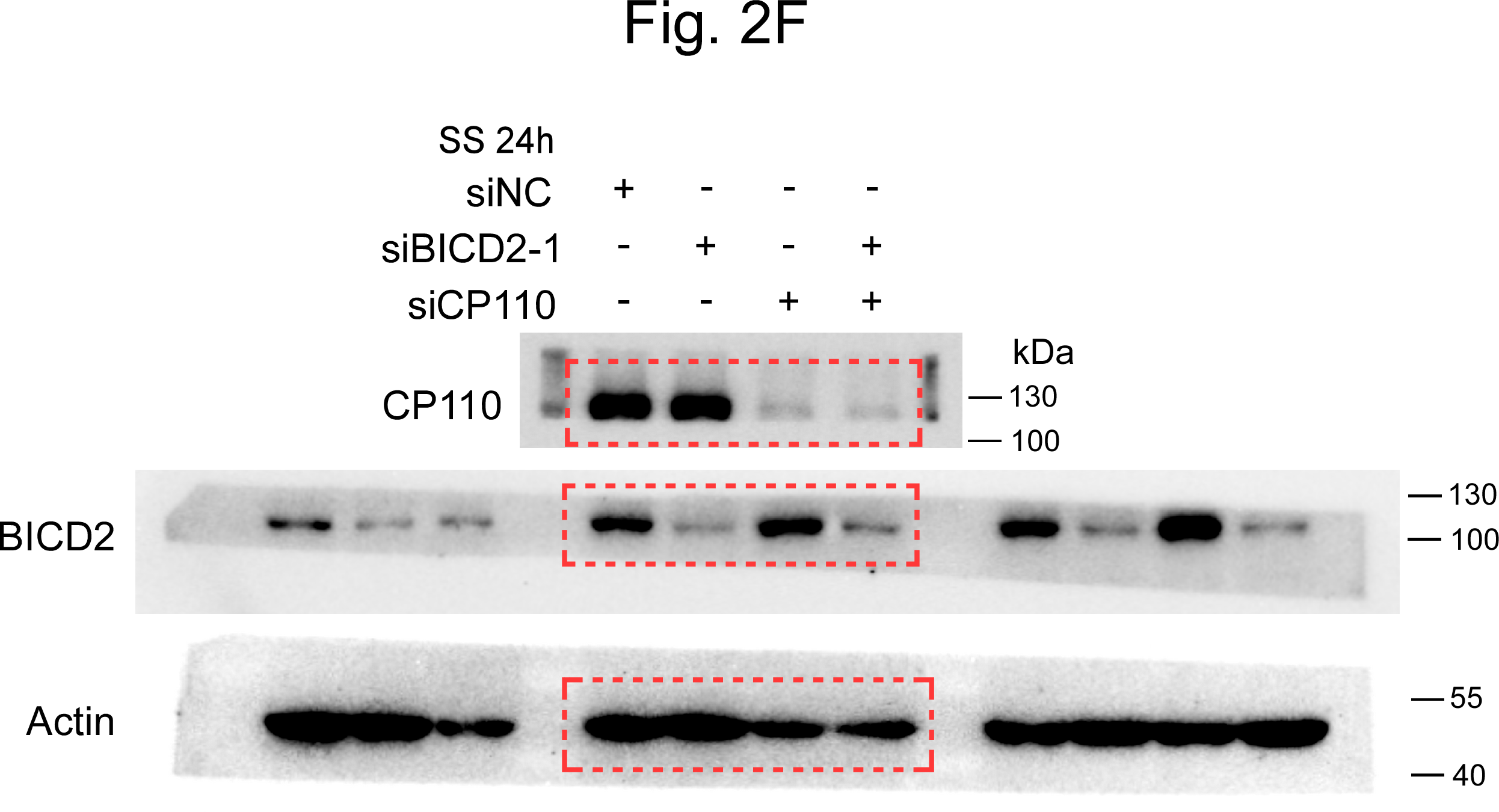

Supplement: Supplementary file 3 — Source data Fig. 2 [file 44319_2025_597_MOESM3_ESM.zip › Figure 2/2F.tif]

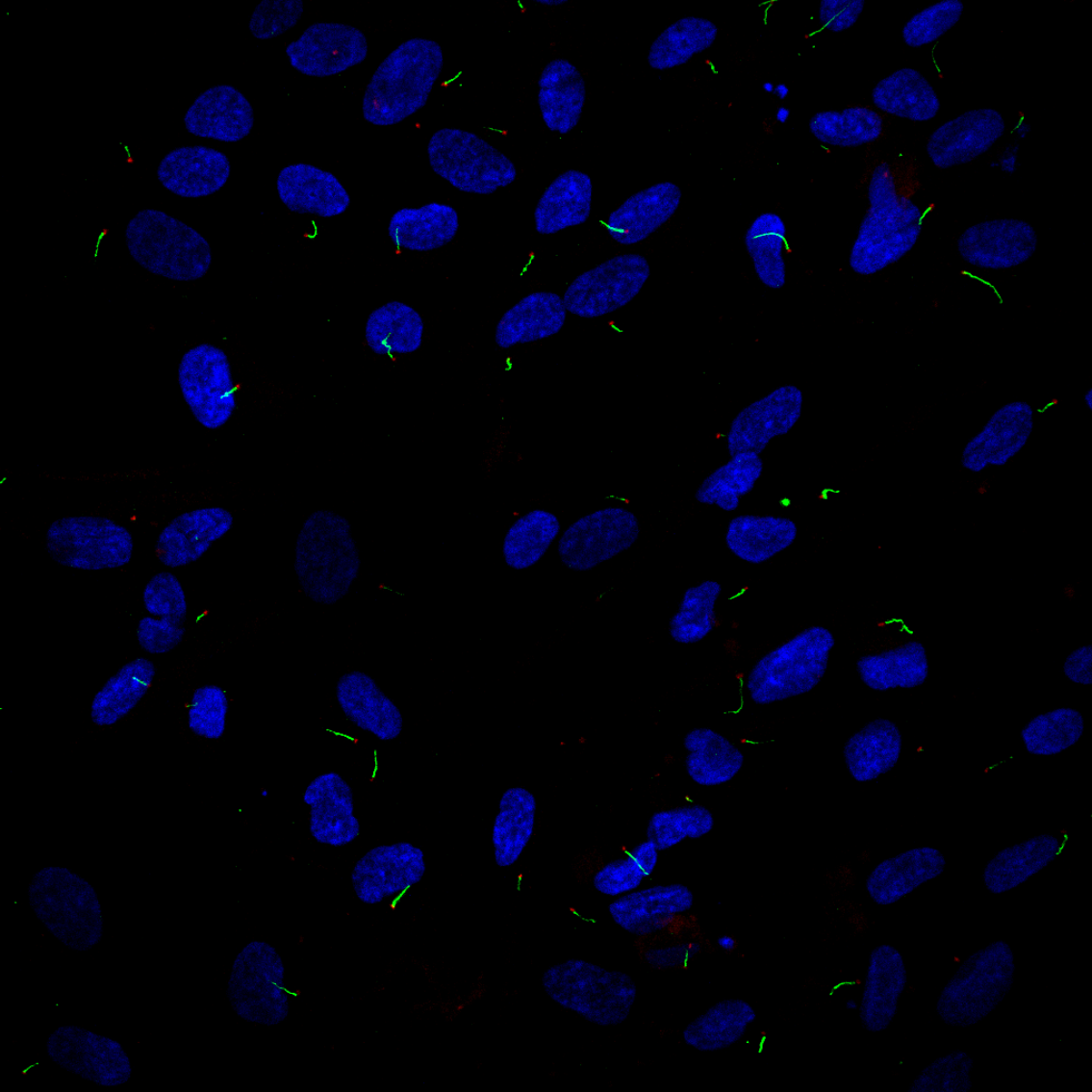

Supplement: Supplementary file 3 — Source data Fig. 2 [file 44319_2025_597_MOESM3_ESM.zip › Figure 2/2G/siBICD2-1+siCP110.bmp]

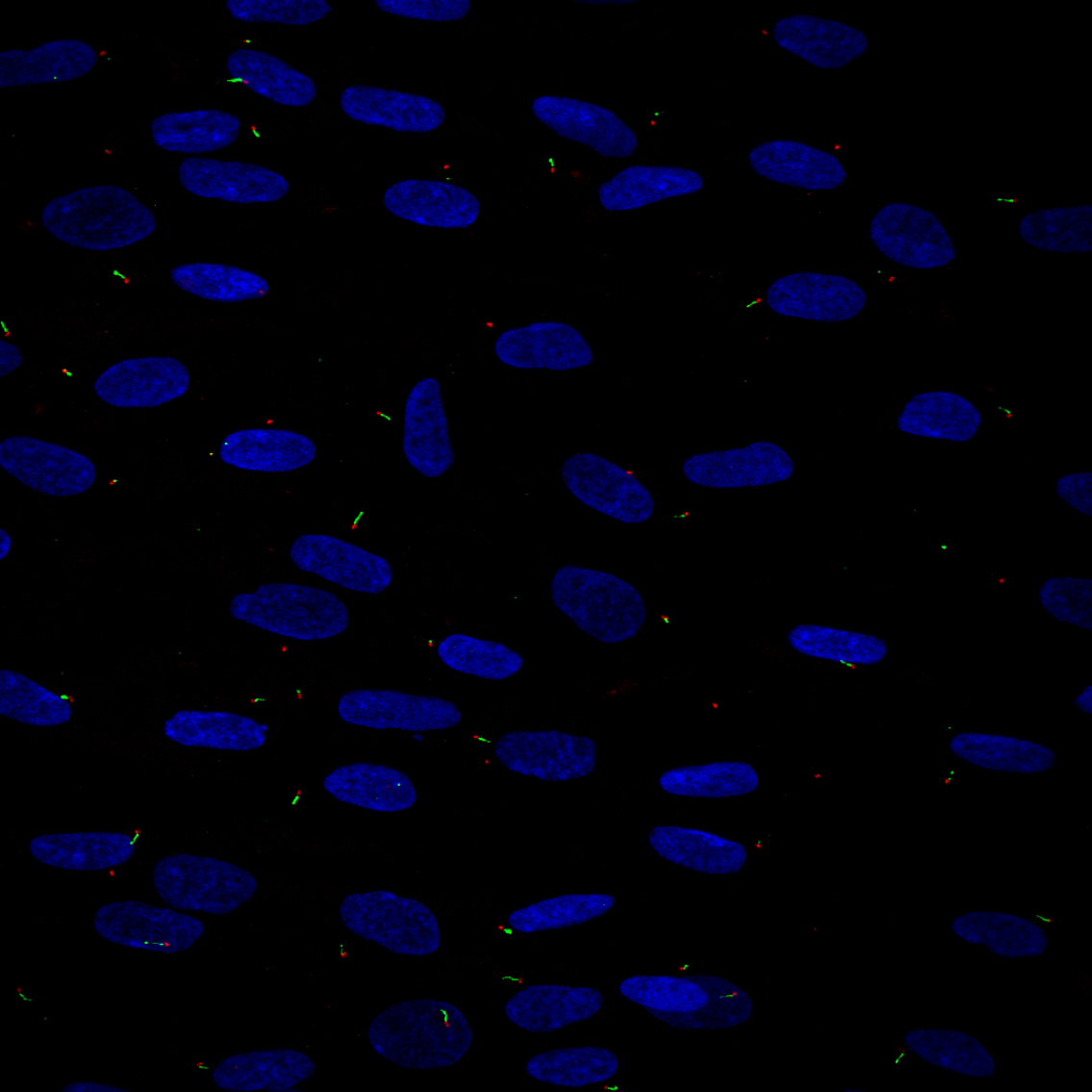

Supplement: Supplementary file 3 — Source data Fig. 2 [file 44319_2025_597_MOESM3_ESM.zip › Figure 2/2G/siBICD2-1.bmp]

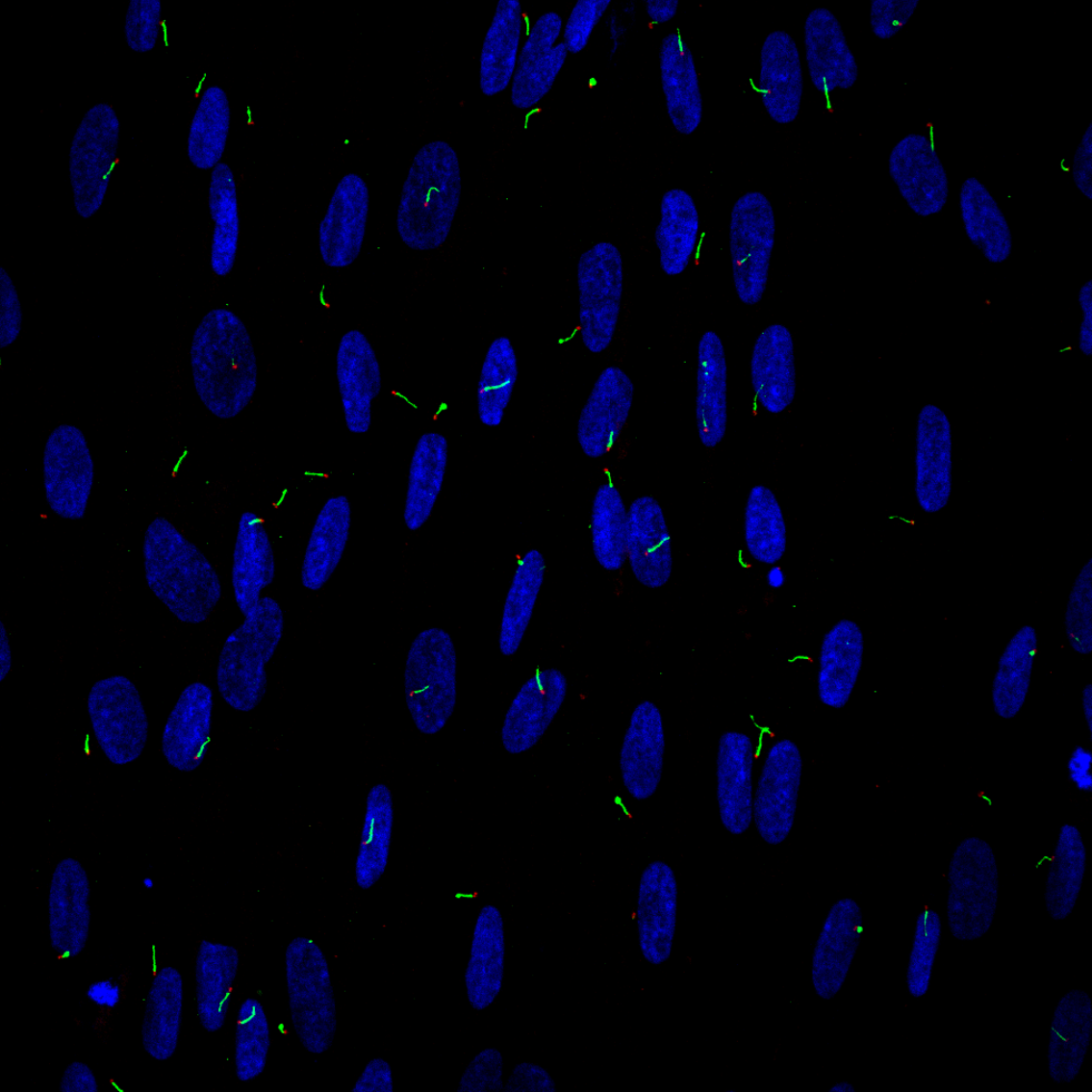

Supplement: Supplementary file 3 — Source data Fig. 2 [file 44319_2025_597_MOESM3_ESM.zip › Figure 2/2G/siCP110.bmp]

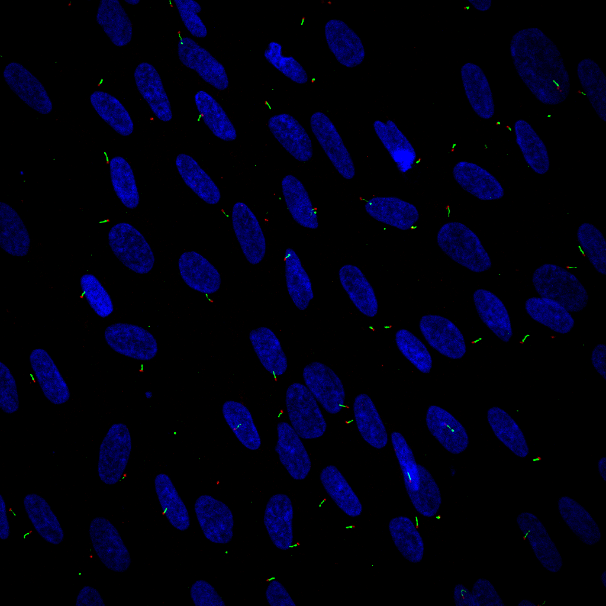

Supplement: Supplementary file 3 — Source data Fig. 2 [file 44319_2025_597_MOESM3_ESM.zip › Figure 2/2G/siNC.bmp]

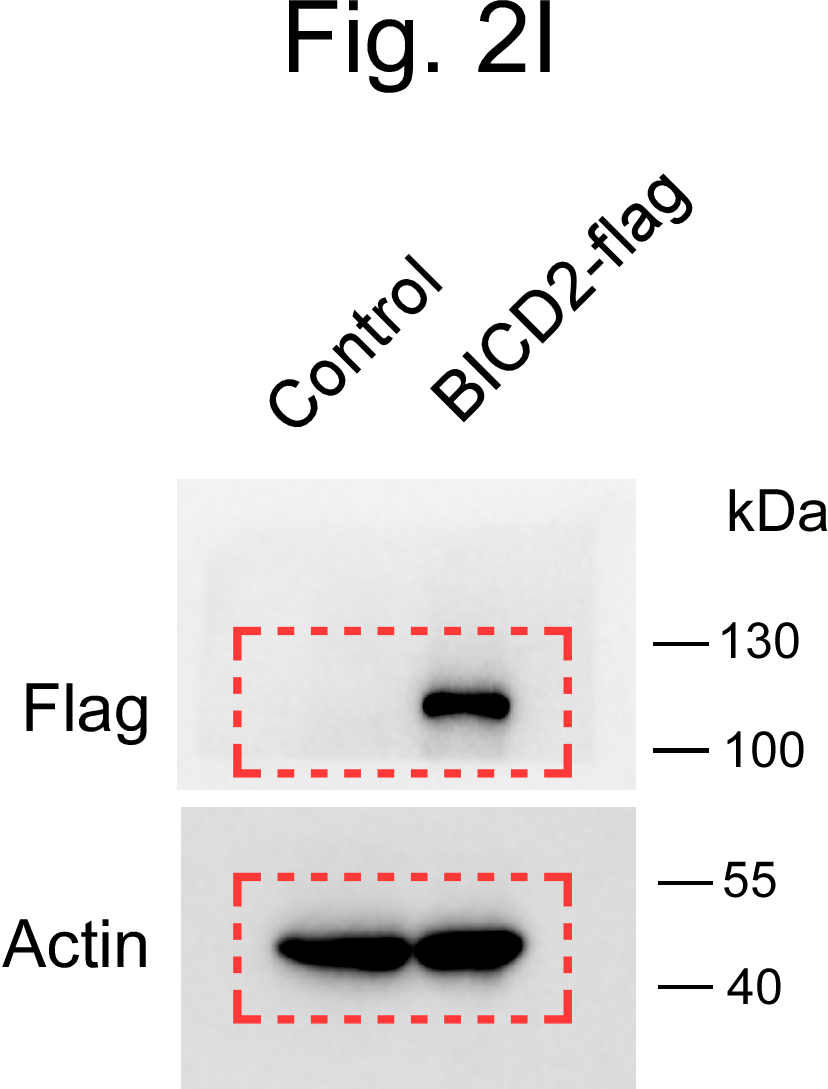

Supplement: Supplementary file 3 — Source data Fig. 2 [file 44319_2025_597_MOESM3_ESM.zip › Figure 2/2I.tif]

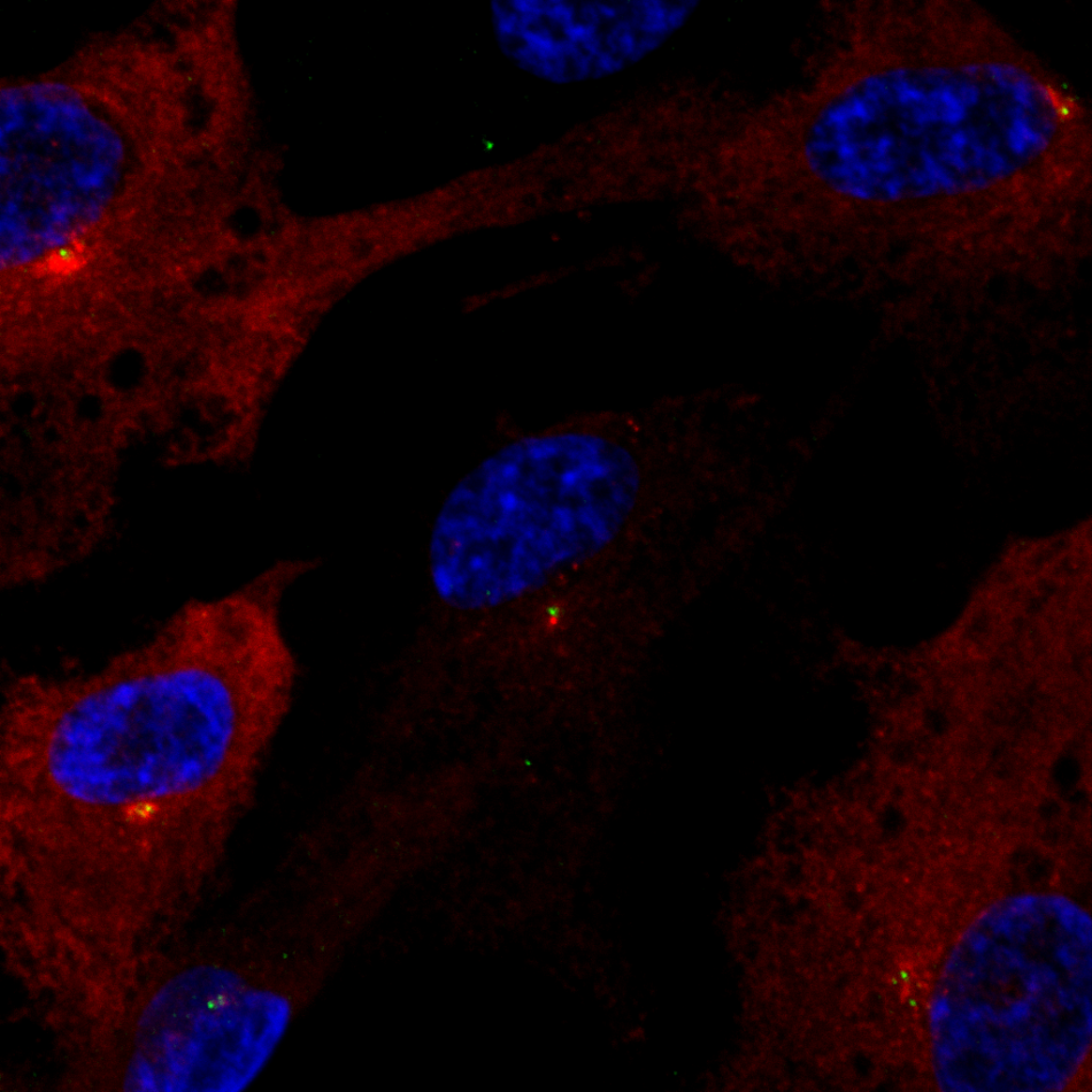

Supplement: Supplementary file 3 — Source data Fig. 2 [file 44319_2025_597_MOESM3_ESM.zip › Figure 2/2J/BICD2-flag.bmp]

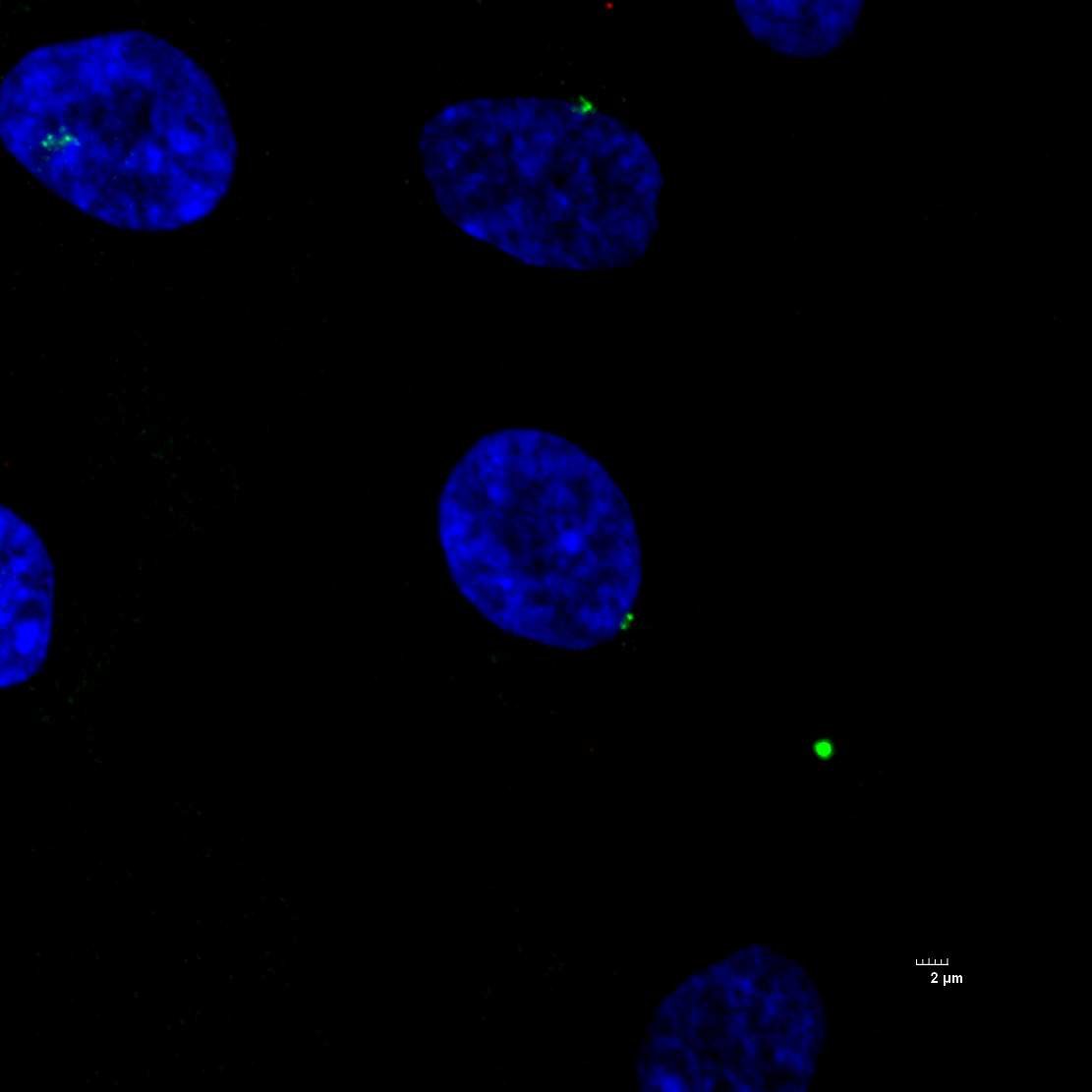

Supplement: Supplementary file 3 — Source data Fig. 2 [file 44319_2025_597_MOESM3_ESM.zip › Figure 2/2J/control.bmp]

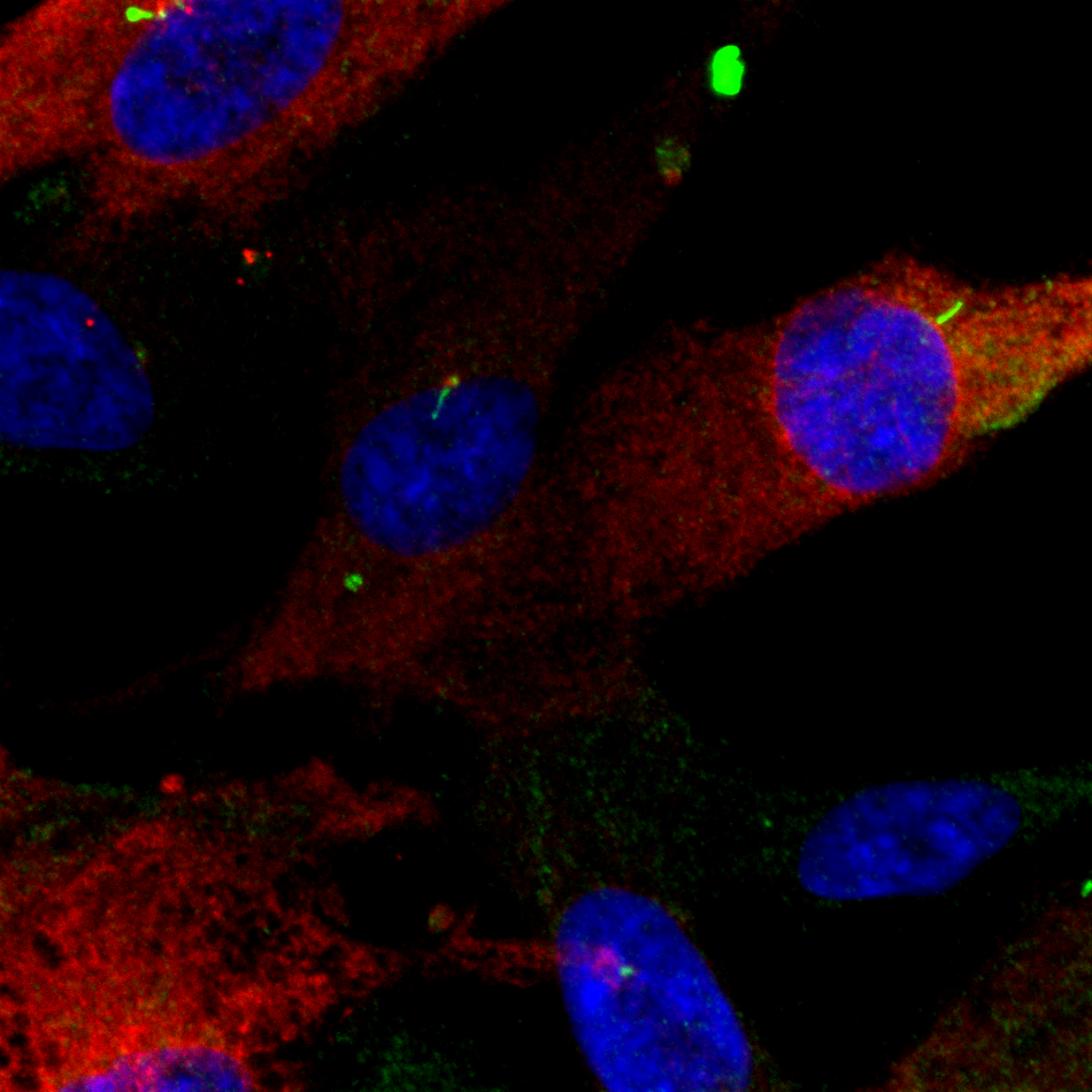

Supplement: Supplementary file 3 — Source data Fig. 2 [file 44319_2025_597_MOESM3_ESM.zip › Figure 2/2L/BICD2-flag.bmp]

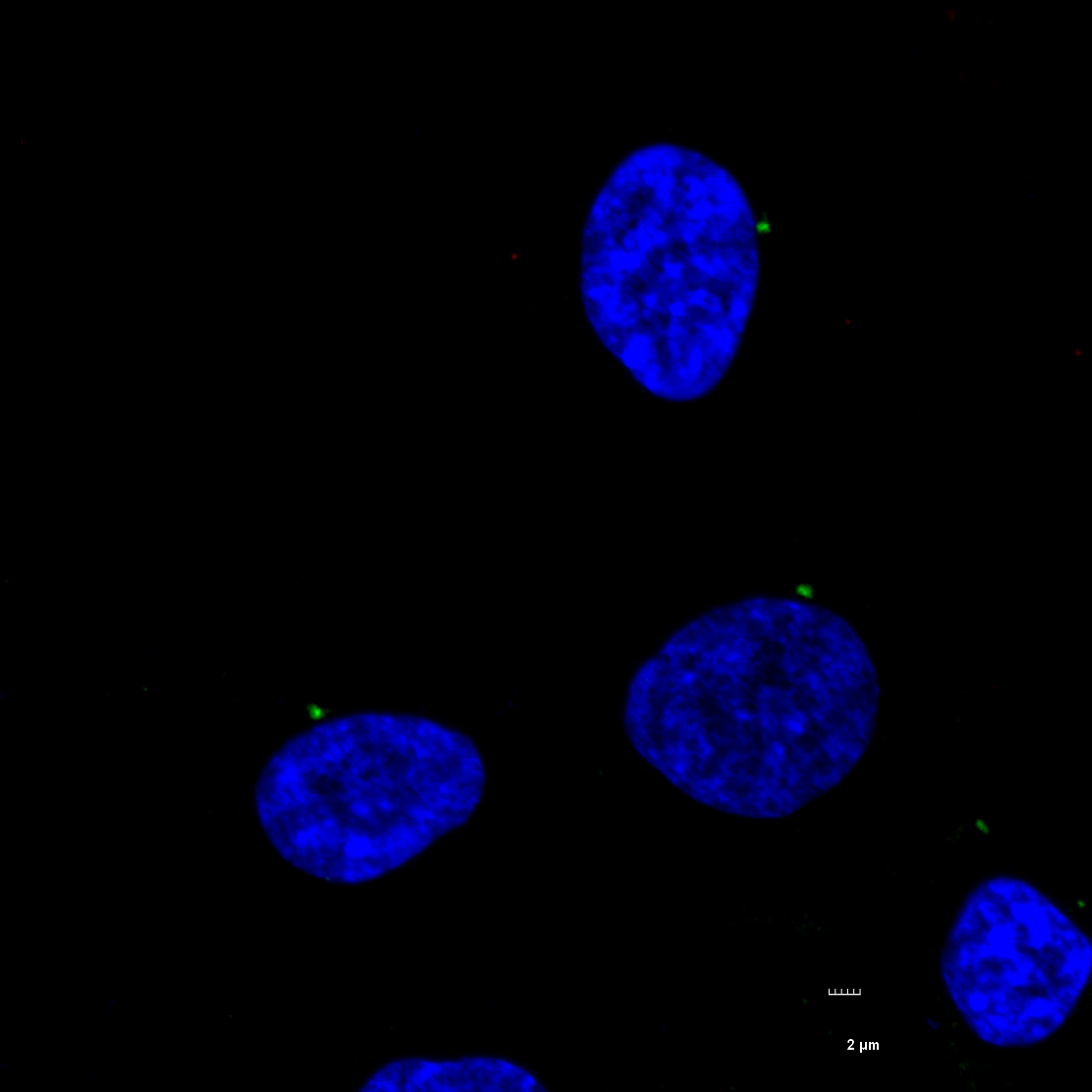

Supplement: Supplementary file 3 — Source data Fig. 2 [file 44319_2025_597_MOESM3_ESM.zip › Figure 2/2L/control.bmp]

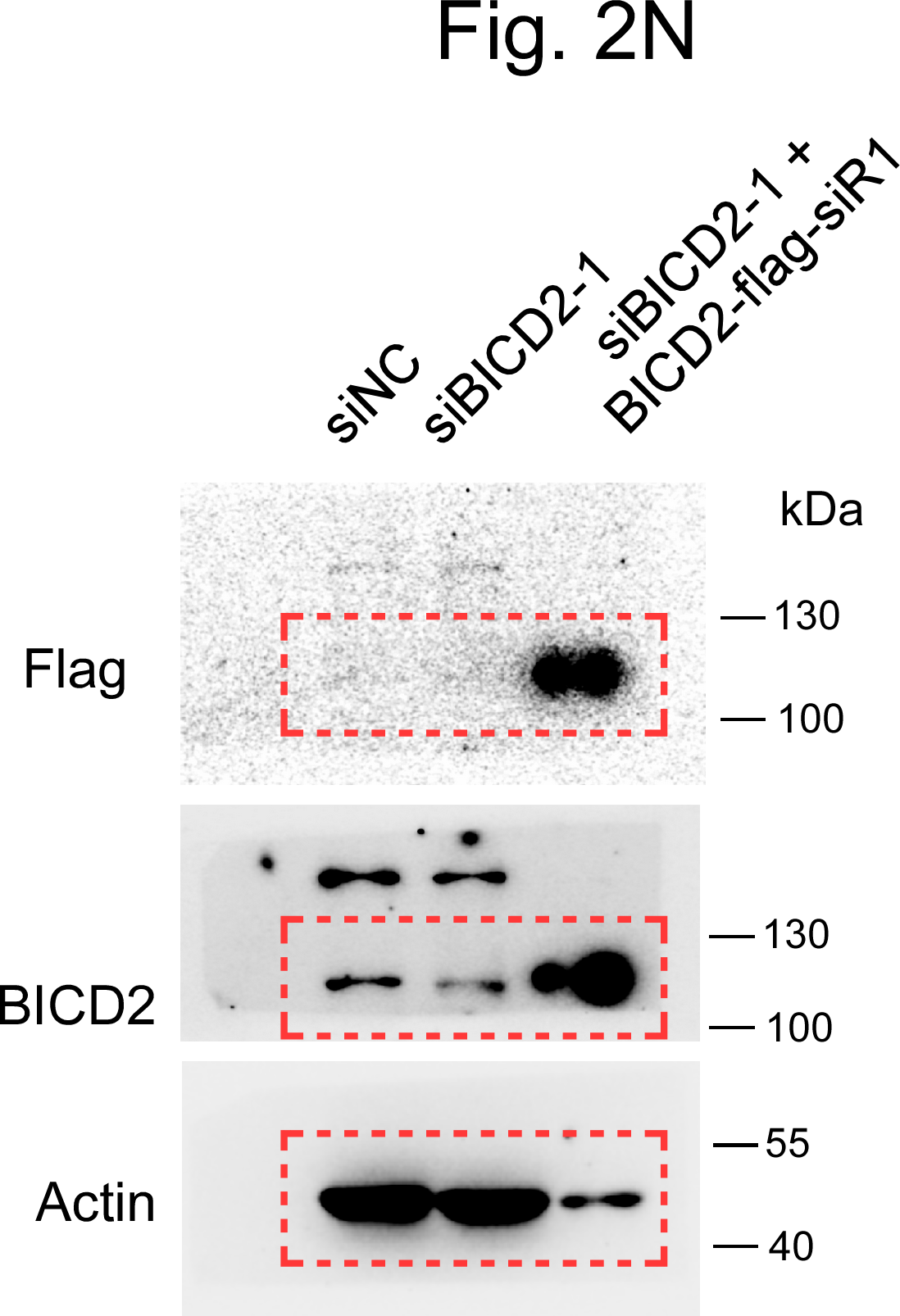

Supplement: Supplementary file 3 — Source data Fig. 2 [file 44319_2025_597_MOESM3_ESM.zip › Figure 2/2N.tif]

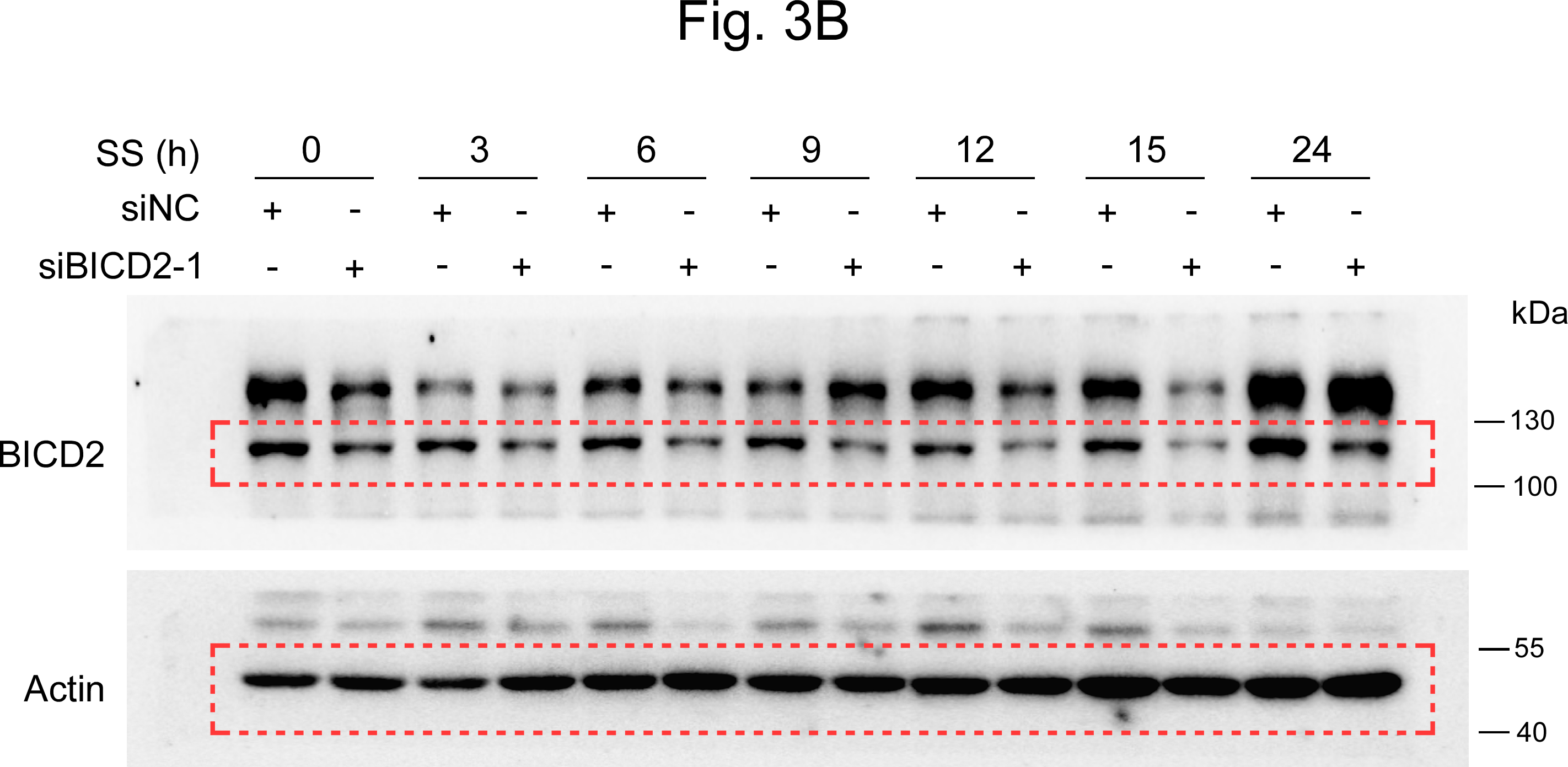

Supplement: Supplementary file 4 — Source data Fig. 3 [file 44319_2025_597_MOESM4_ESM.zip › Figure 3/3B_WB.tif]

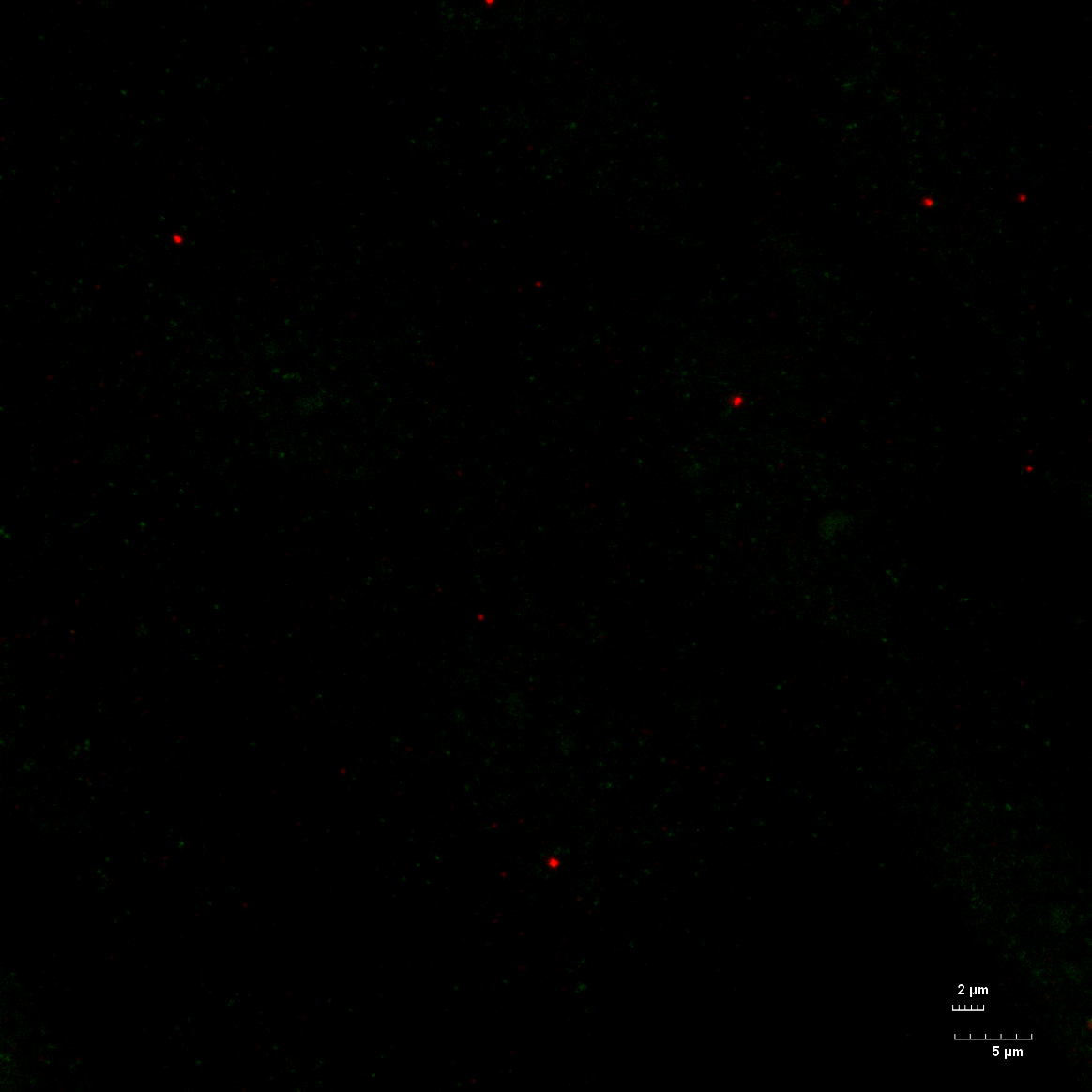

Supplement: Supplementary file 4 — Source data Fig. 3 [file 44319_2025_597_MOESM4_ESM.zip › Figure 3/3C/CEP164+BICD2/siBICD2-1/SS0h.bmp]

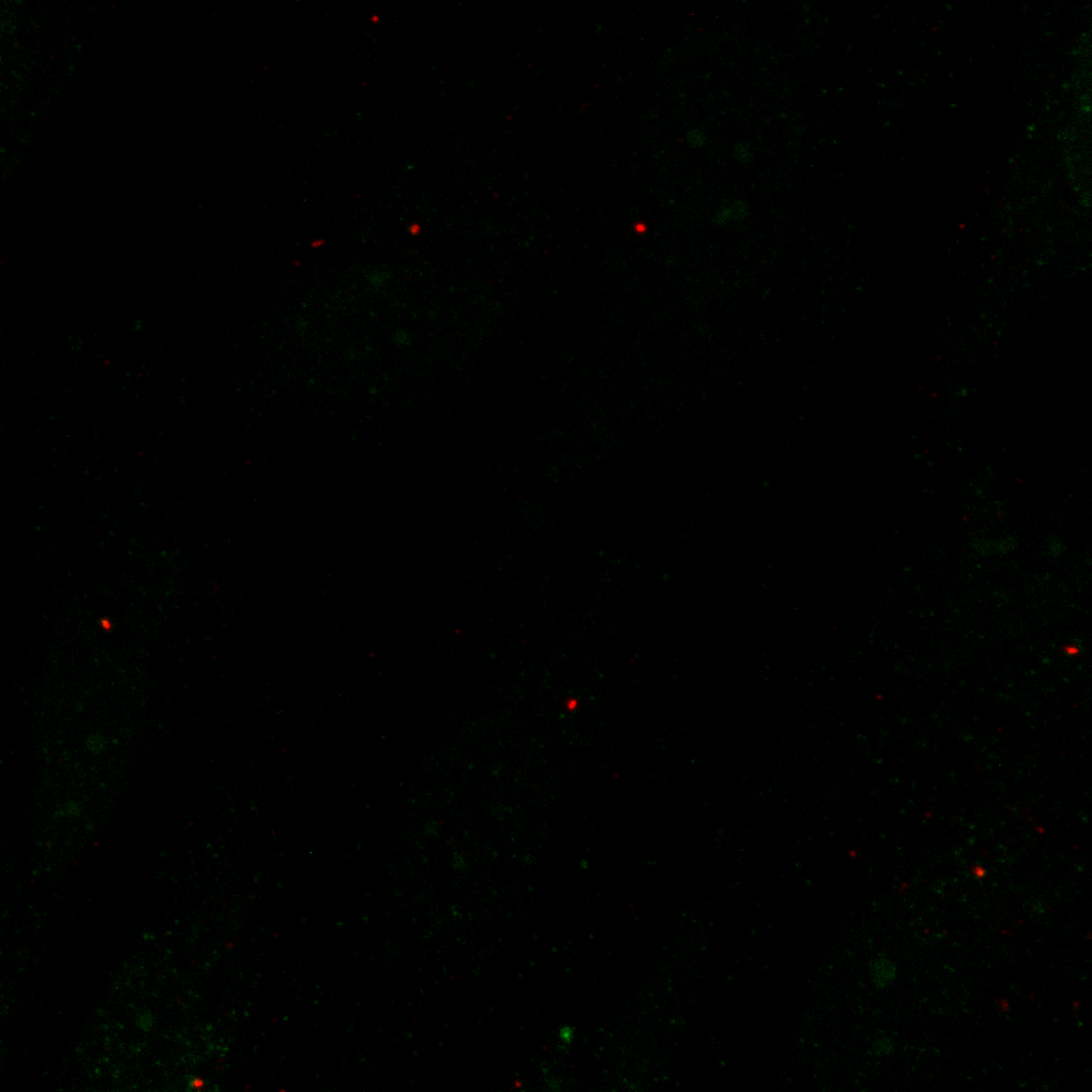

Supplement: Supplementary file 4 — Source data Fig. 3 [file 44319_2025_597_MOESM4_ESM.zip › Figure 3/3C/CEP164+BICD2/siBICD2-1/SS12h.bmp]

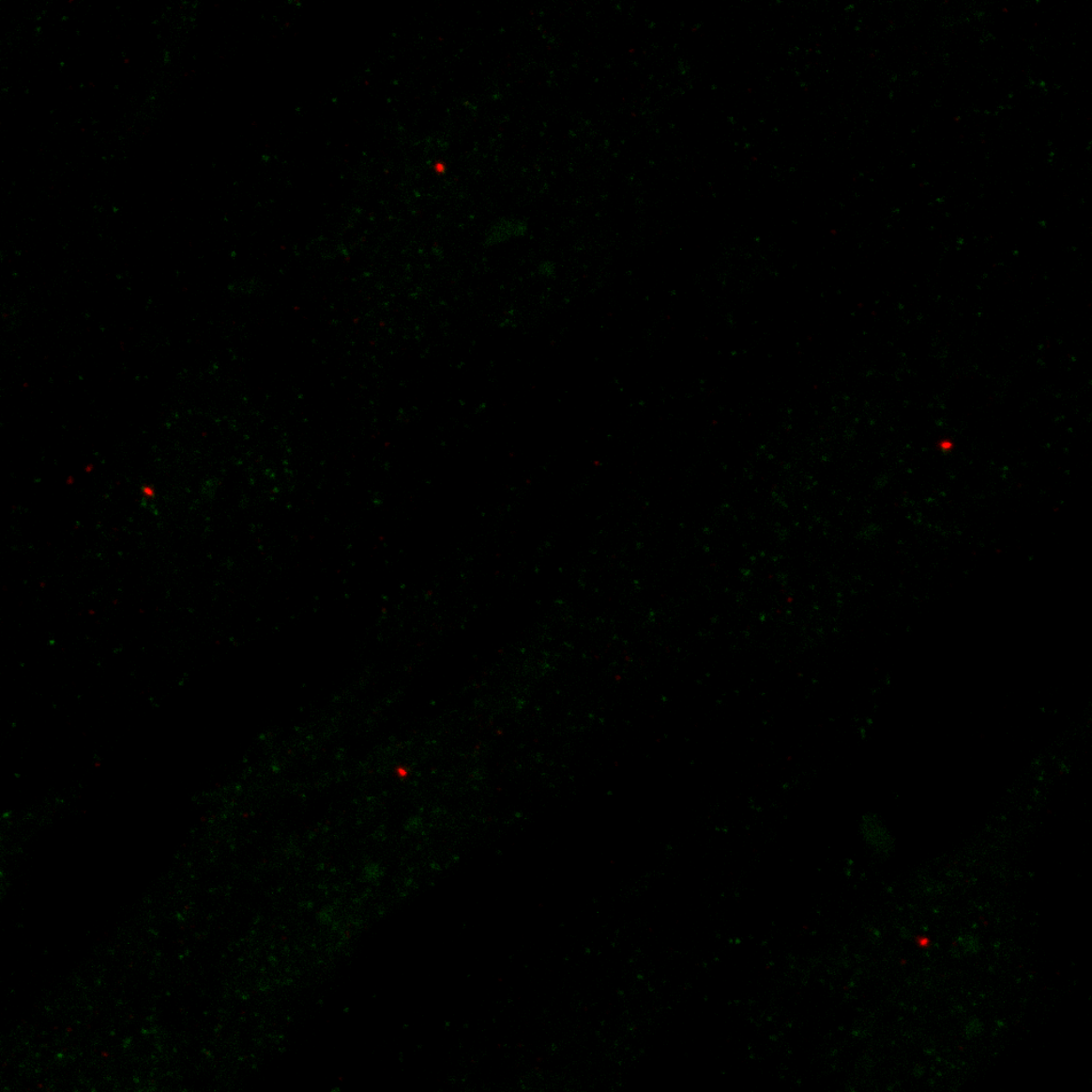

Supplement: Supplementary file 4 — Source data Fig. 3 [file 44319_2025_597_MOESM4_ESM.zip › Figure 3/3C/CEP164+BICD2/siBICD2-1/SS15h.bmp]

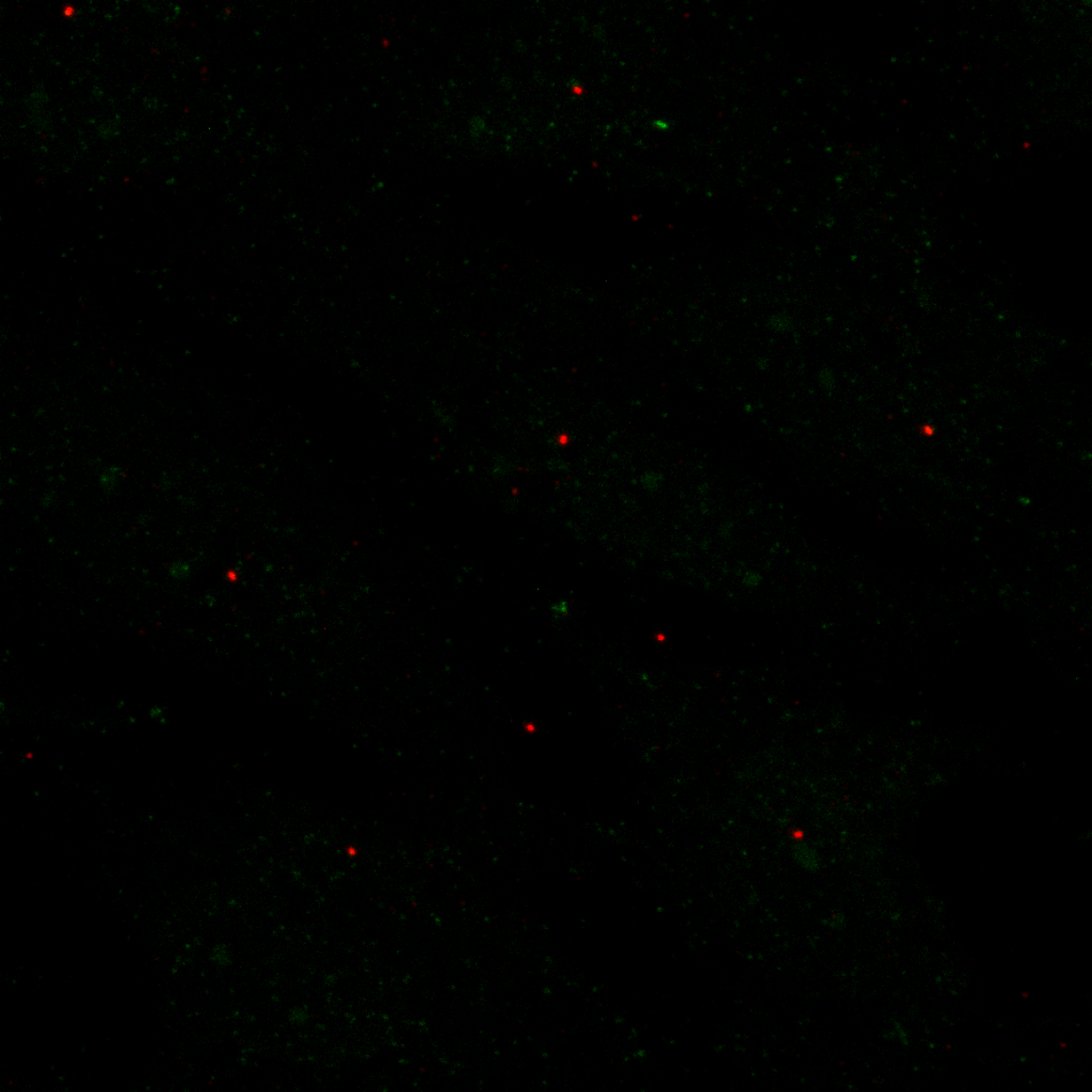

Supplement: Supplementary file 4 — Source data Fig. 3 [file 44319_2025_597_MOESM4_ESM.zip › Figure 3/3C/CEP164+BICD2/siBICD2-1/SS24h.bmp]

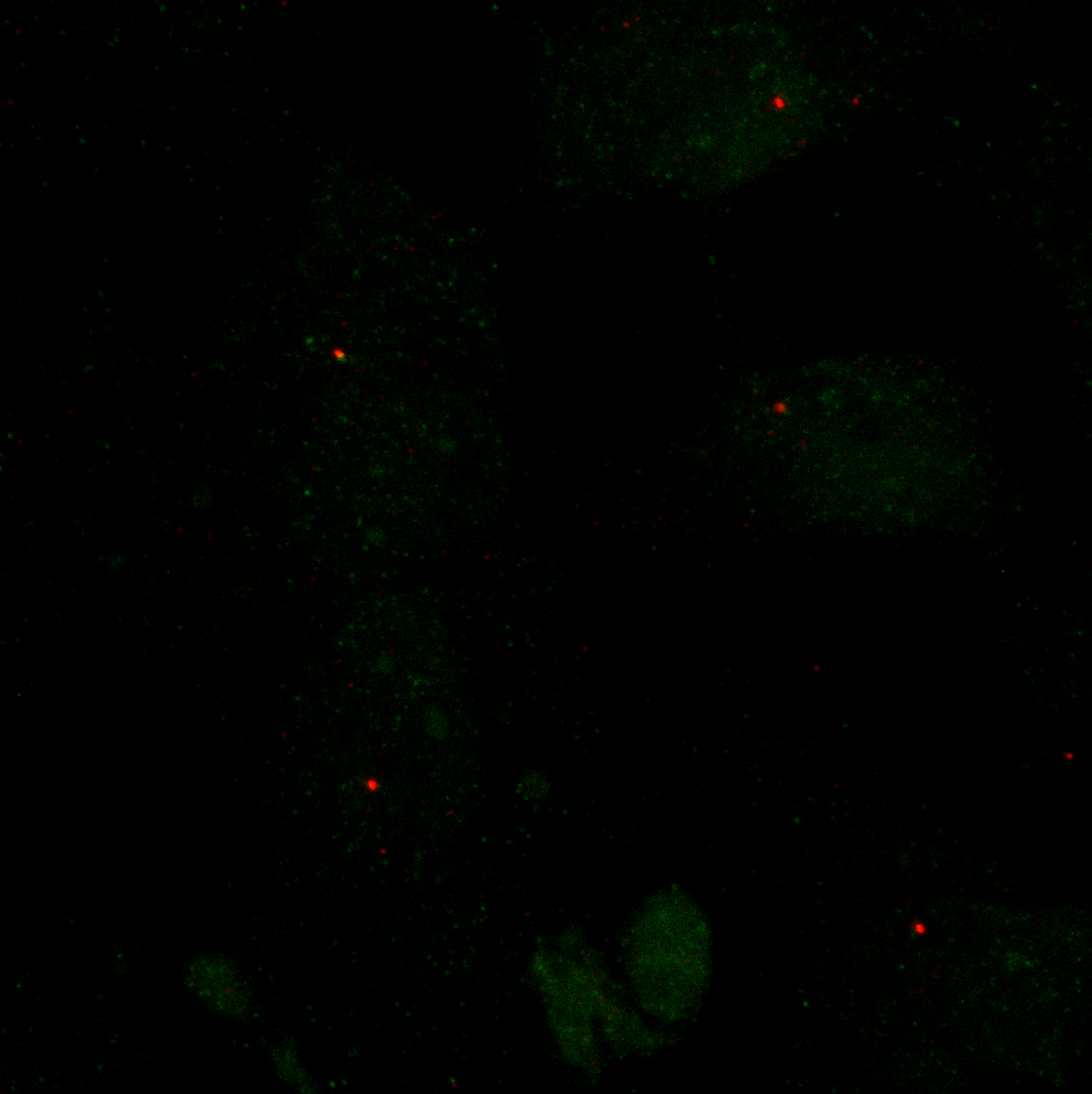

Supplement: Supplementary file 4 — Source data Fig. 3 [file 44319_2025_597_MOESM4_ESM.zip › Figure 3/3C/CEP164+BICD2/siBICD2-1/SS3h.bmp]

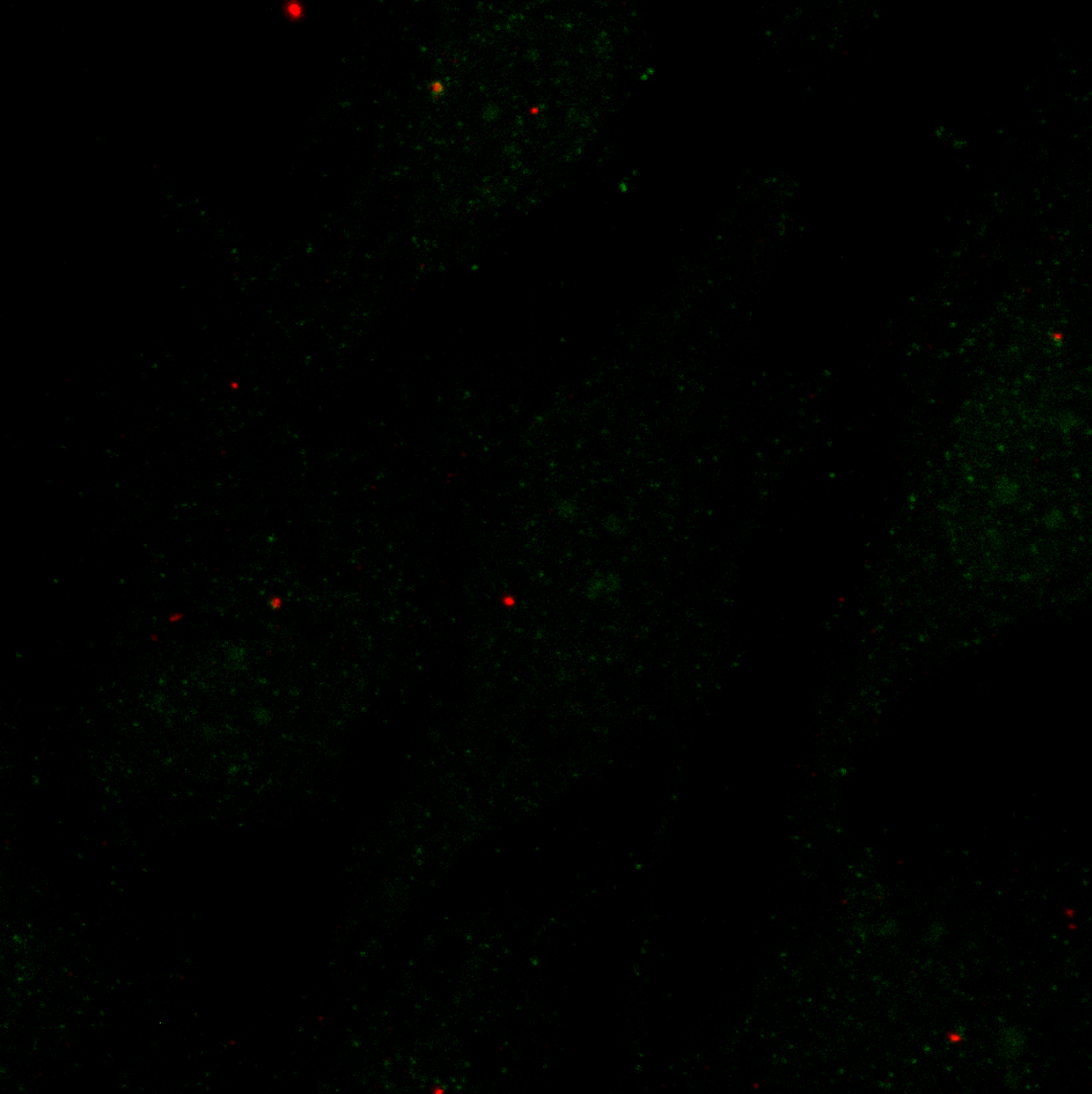

Supplement: Supplementary file 4 — Source data Fig. 3 [file 44319_2025_597_MOESM4_ESM.zip › Figure 3/3C/CEP164+BICD2/siBICD2-1/SS6h.bmp]

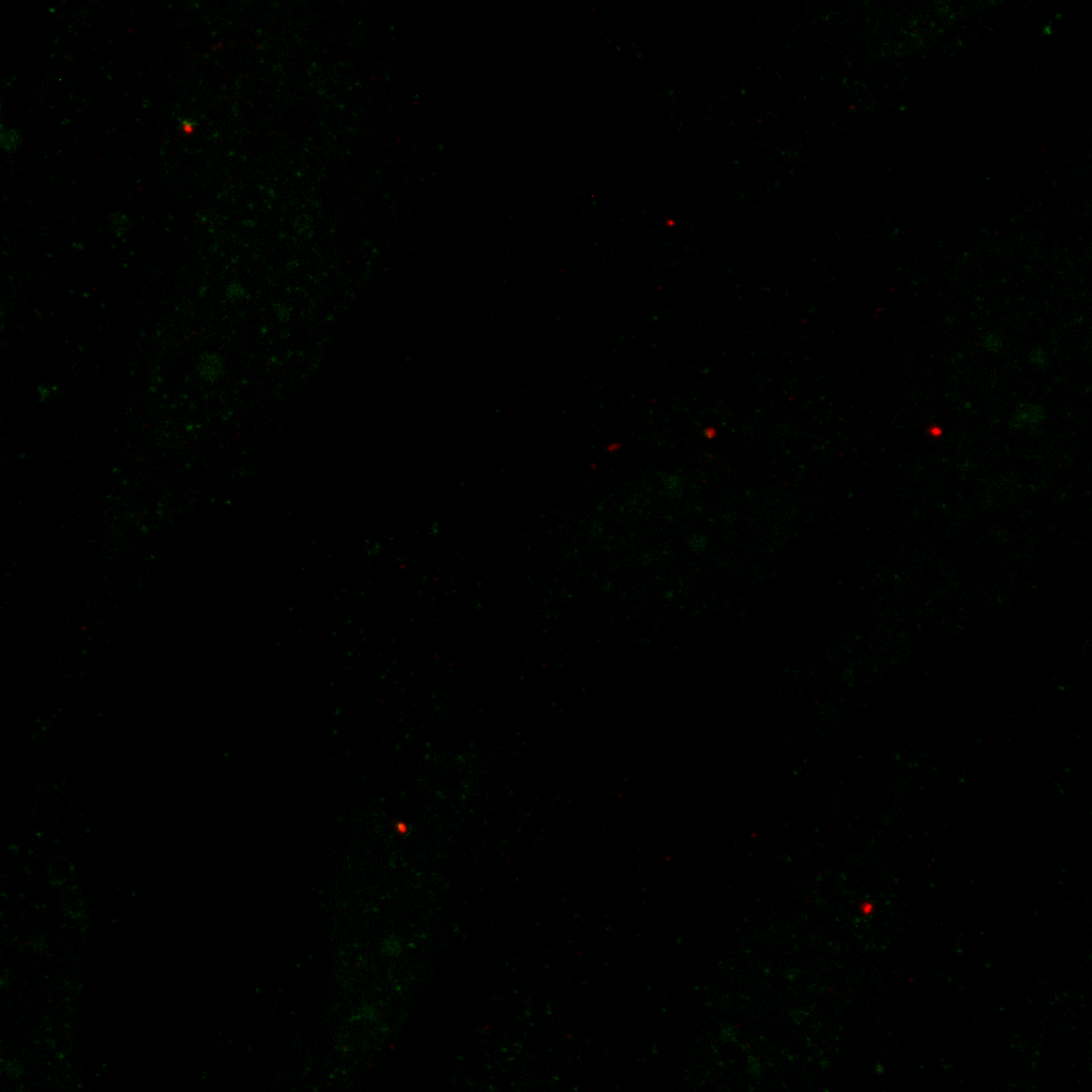

Supplement: Supplementary file 4 — Source data Fig. 3 [file 44319_2025_597_MOESM4_ESM.zip › Figure 3/3C/CEP164+BICD2/siBICD2-1/SS9h.bmp]

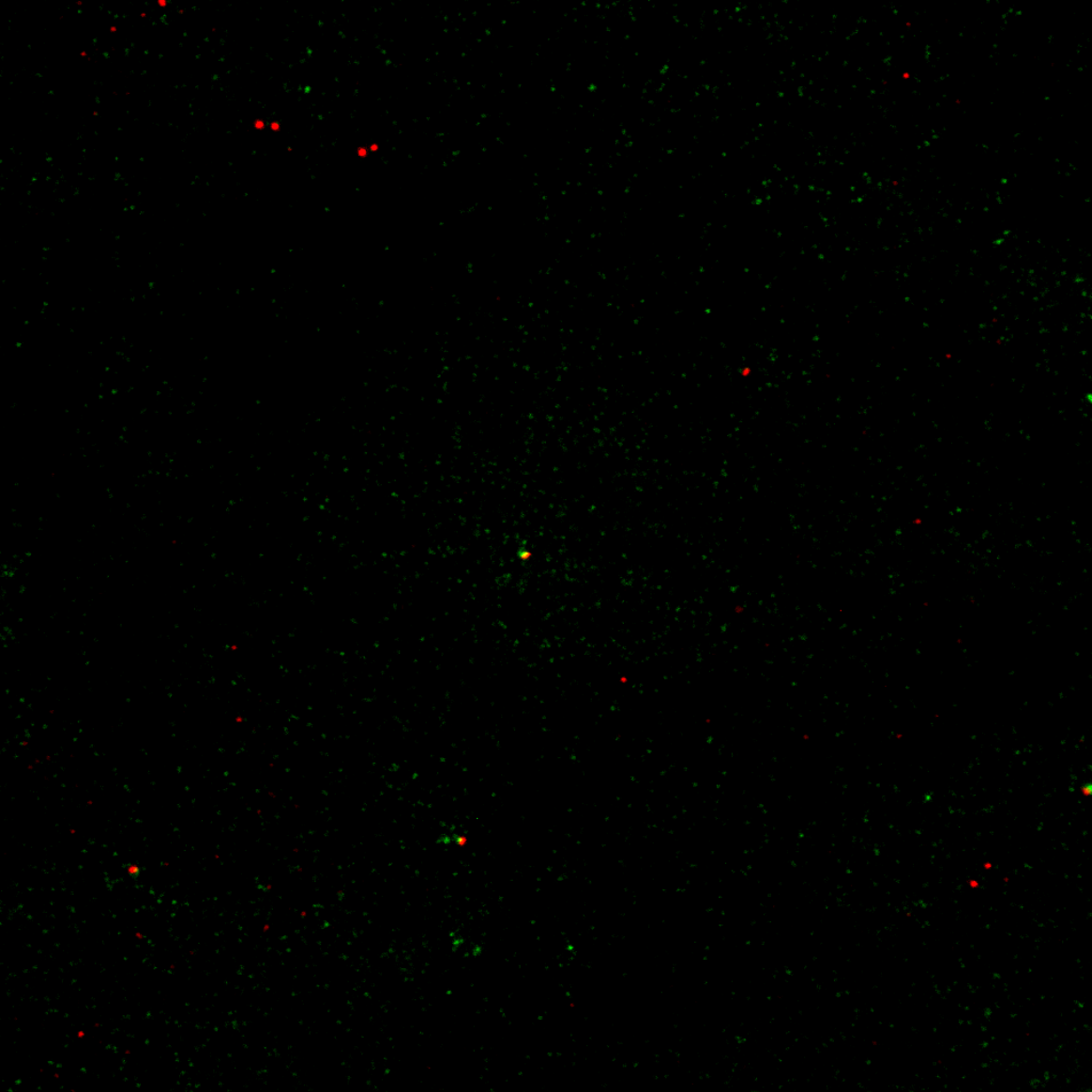

Supplement: Supplementary file 4 — Source data Fig. 3 [file 44319_2025_597_MOESM4_ESM.zip › Figure 3/3C/CEP164+BICD2/siNC/SS0h.bmp]

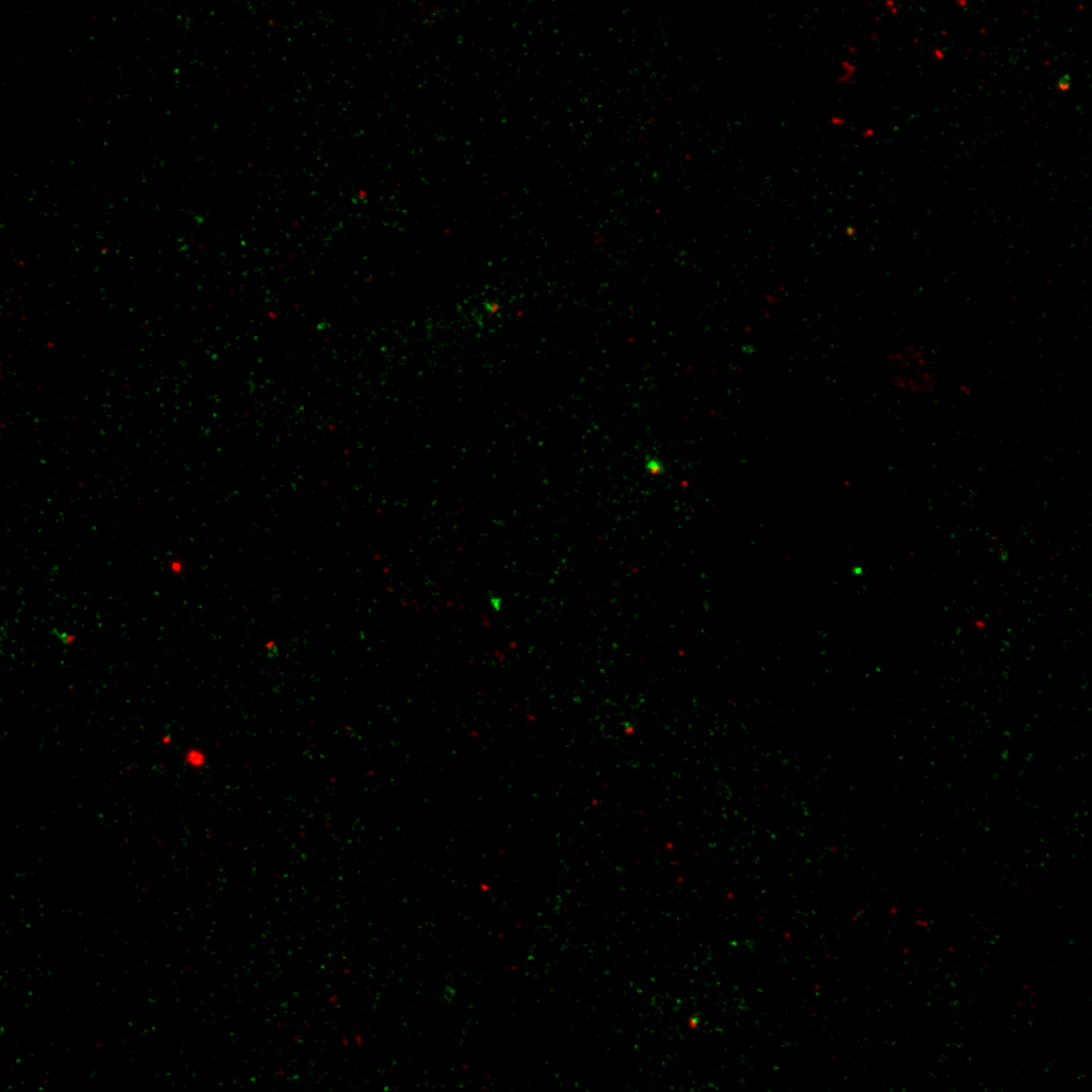

Supplement: Supplementary file 4 — Source data Fig. 3 [file 44319_2025_597_MOESM4_ESM.zip › Figure 3/3C/CEP164+BICD2/siNC/SS12h.bmp]

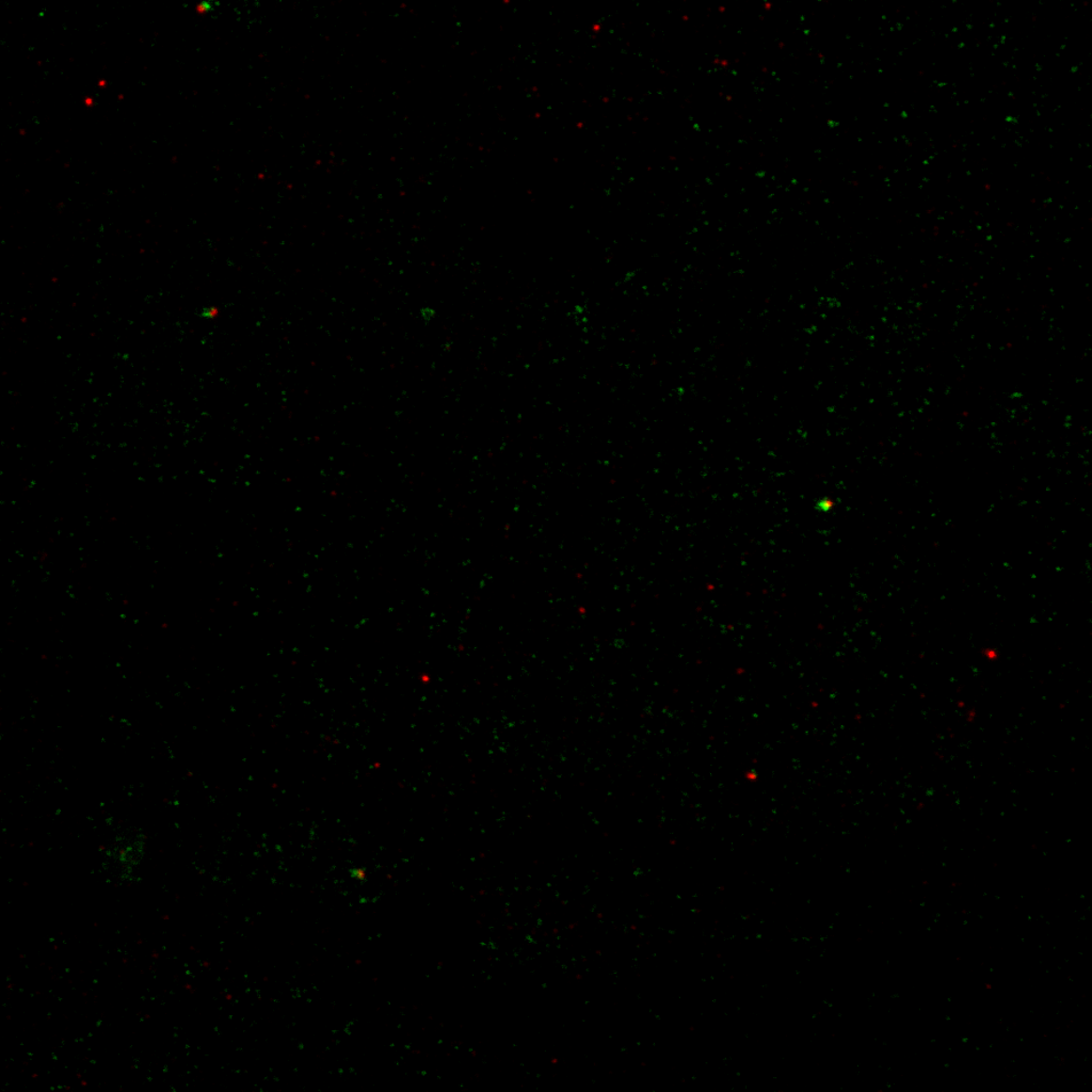

Supplement: Supplementary file 4 — Source data Fig. 3 [file 44319_2025_597_MOESM4_ESM.zip › Figure 3/3C/CEP164+BICD2/siNC/SS15h.bmp]

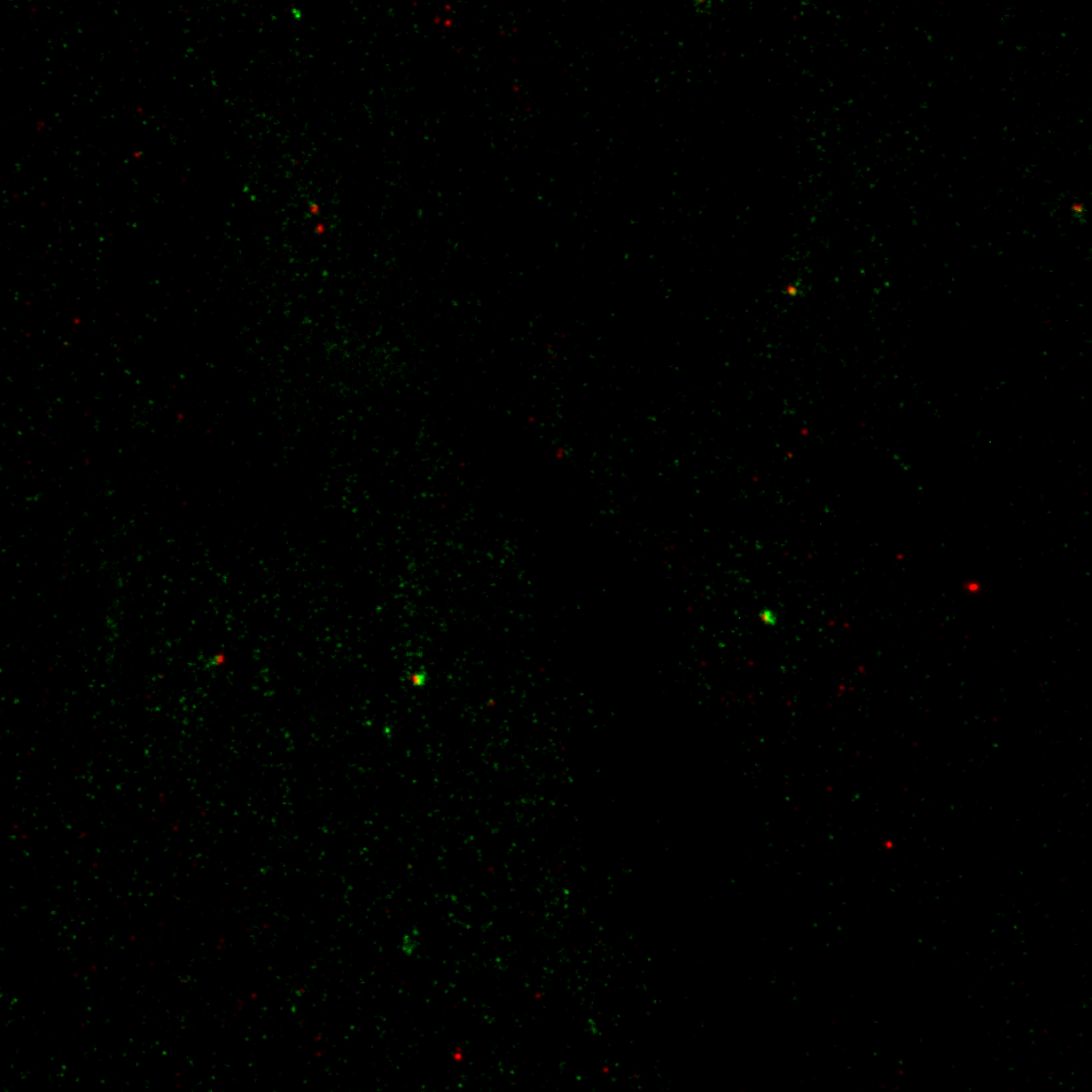

Supplement: Supplementary file 4 — Source data Fig. 3 [file 44319_2025_597_MOESM4_ESM.zip › Figure 3/3C/CEP164+BICD2/siNC/SS24h.bmp]

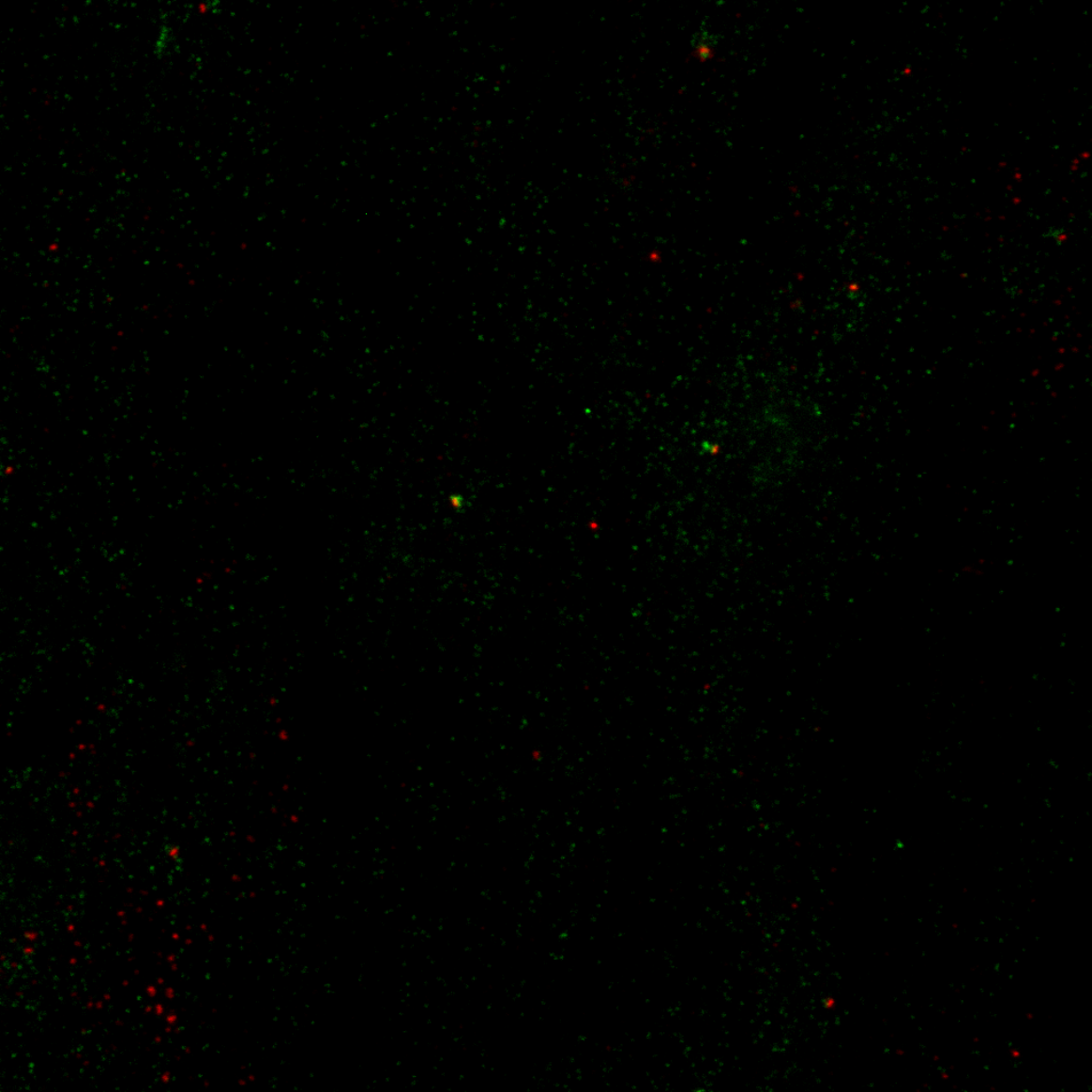

Supplement: Supplementary file 4 — Source data Fig. 3 [file 44319_2025_597_MOESM4_ESM.zip › Figure 3/3C/CEP164+BICD2/siNC/SS3h.bmp]

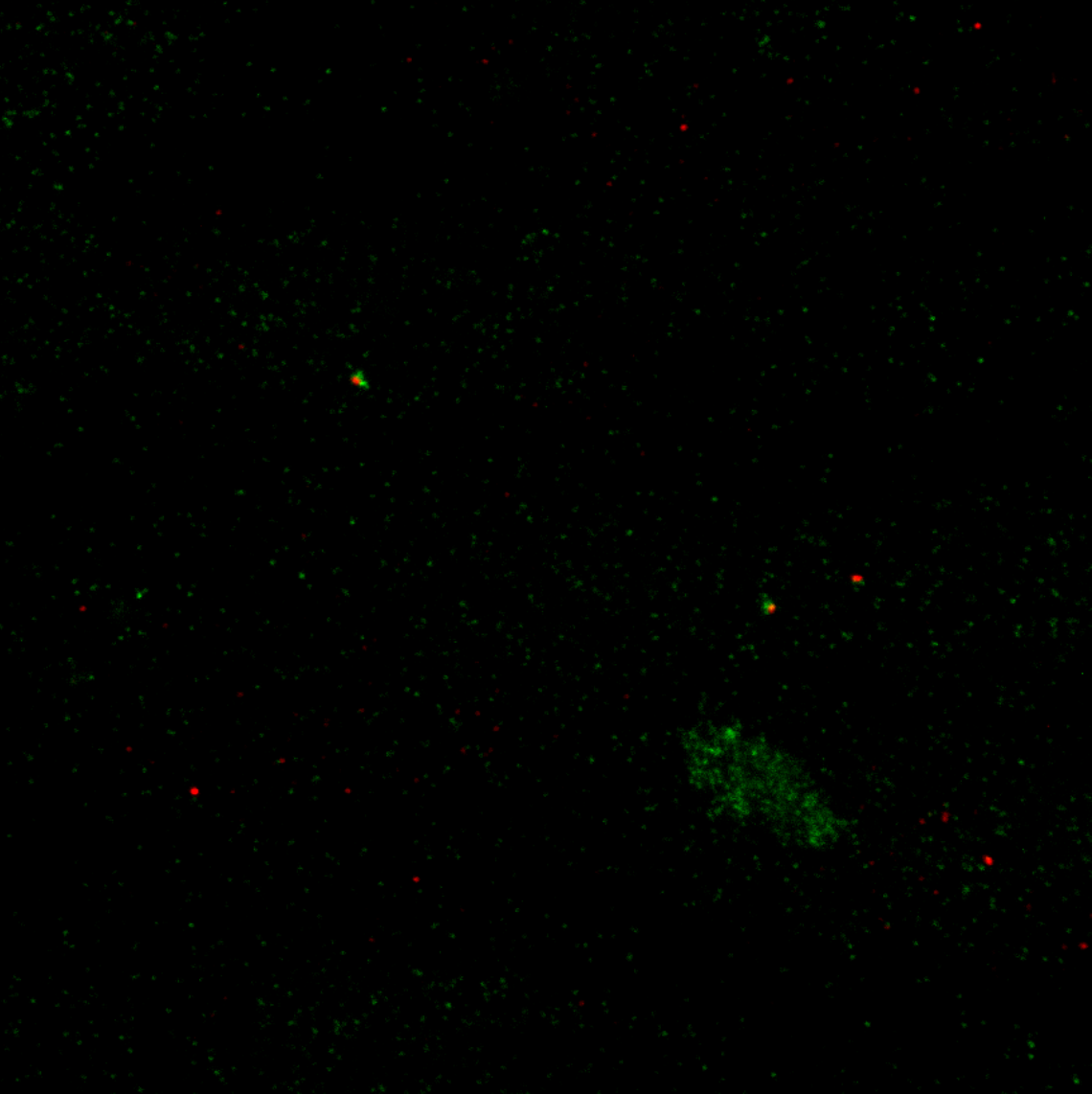

Supplement: Supplementary file 4 — Source data Fig. 3 [file 44319_2025_597_MOESM4_ESM.zip › Figure 3/3C/CEP164+BICD2/siNC/SS6h.bmp]

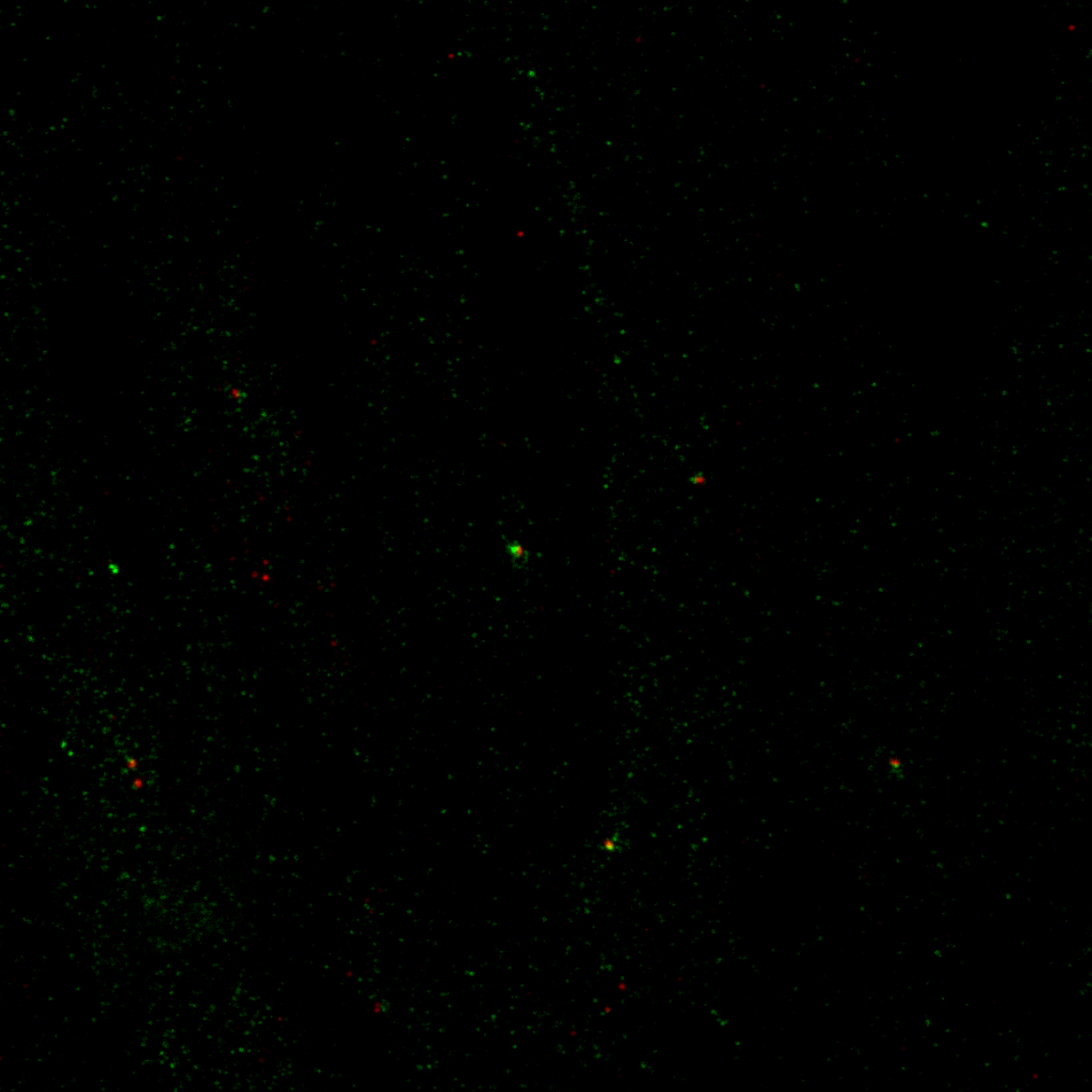

Supplement: Supplementary file 4 — Source data Fig. 3 [file 44319_2025_597_MOESM4_ESM.zip › Figure 3/3C/CEP164+BICD2/siNC/SS9h.bmp]

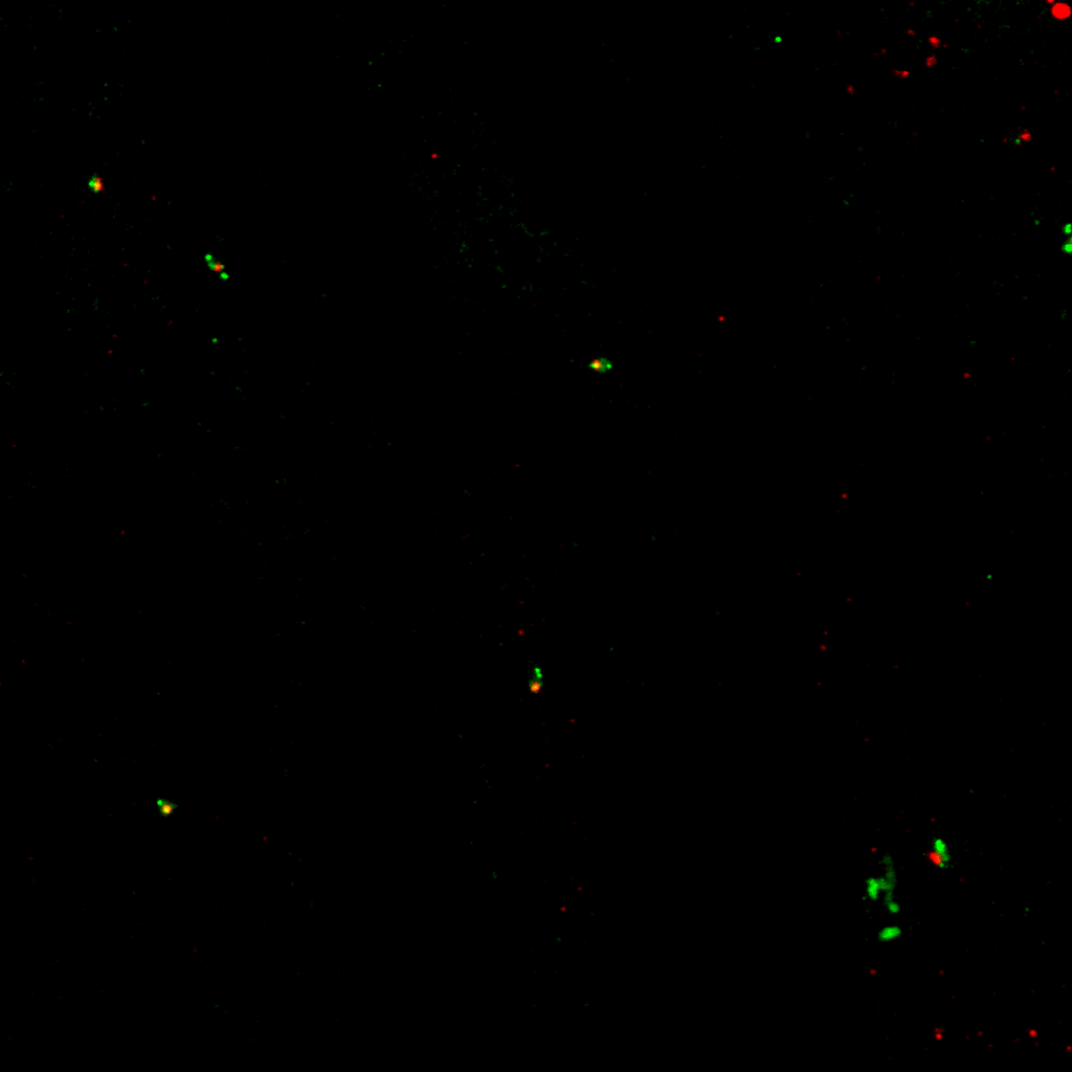

Supplement: Supplementary file 4 — Source data Fig. 3 [file 44319_2025_597_MOESM4_ESM.zip › Figure 3/3C/CEP164+CP110/siBICD2-1/SS0h.bmp]

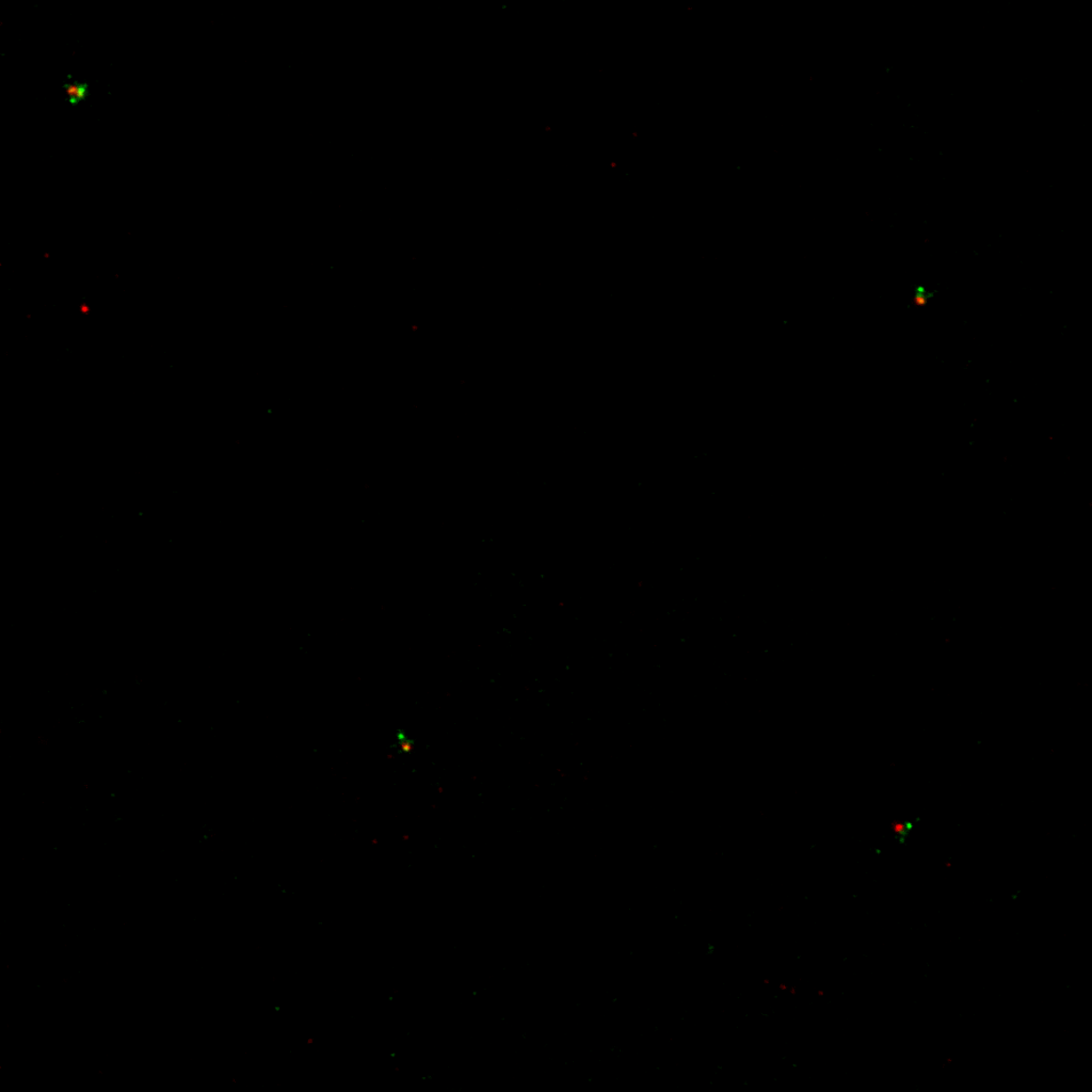

Supplement: Supplementary file 4 — Source data Fig. 3 [file 44319_2025_597_MOESM4_ESM.zip › Figure 3/3C/CEP164+CP110/siBICD2-1/SS12h.bmp]

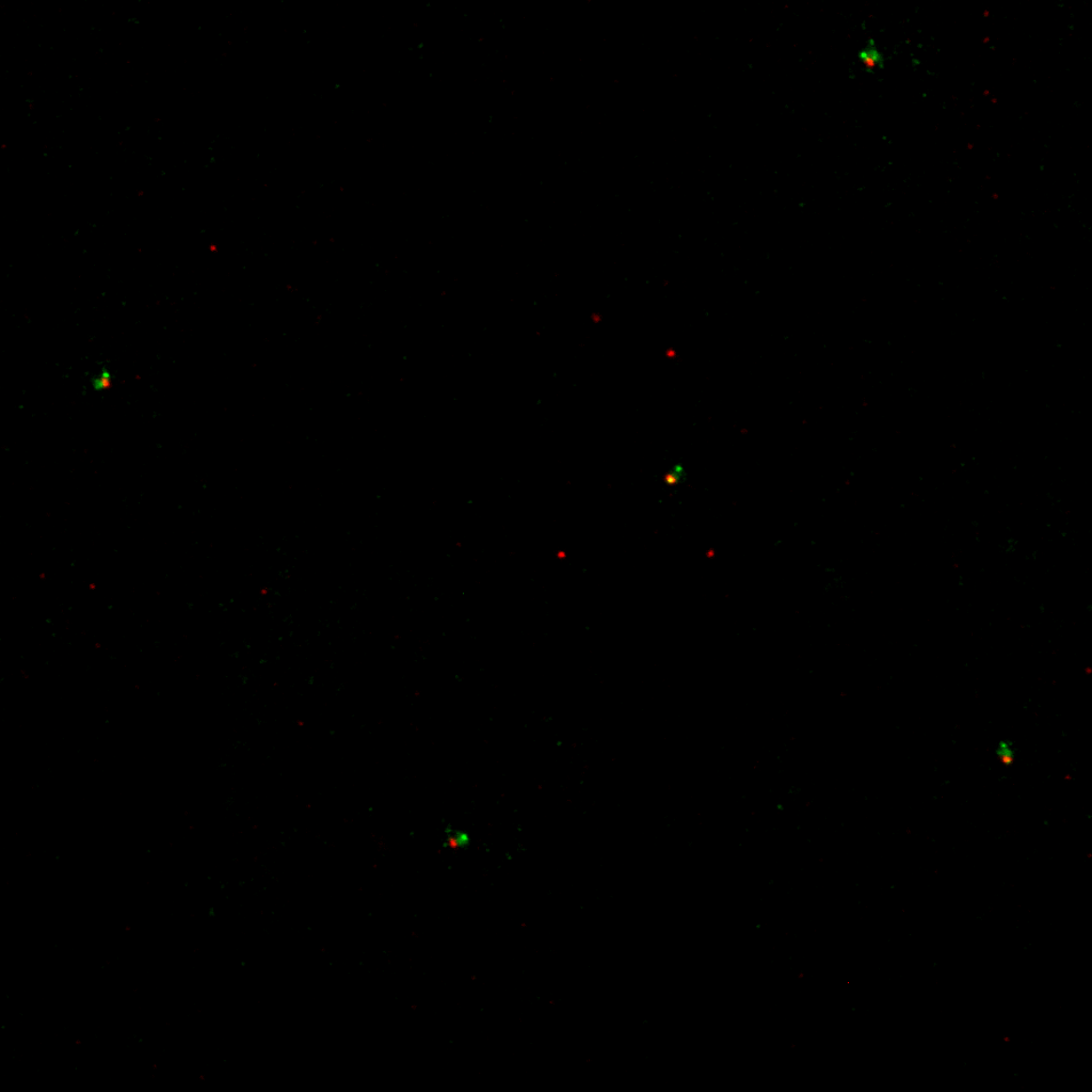

Supplement: Supplementary file 4 — Source data Fig. 3 [file 44319_2025_597_MOESM4_ESM.zip › Figure 3/3C/CEP164+CP110/siBICD2-1/SS15h.bmp]

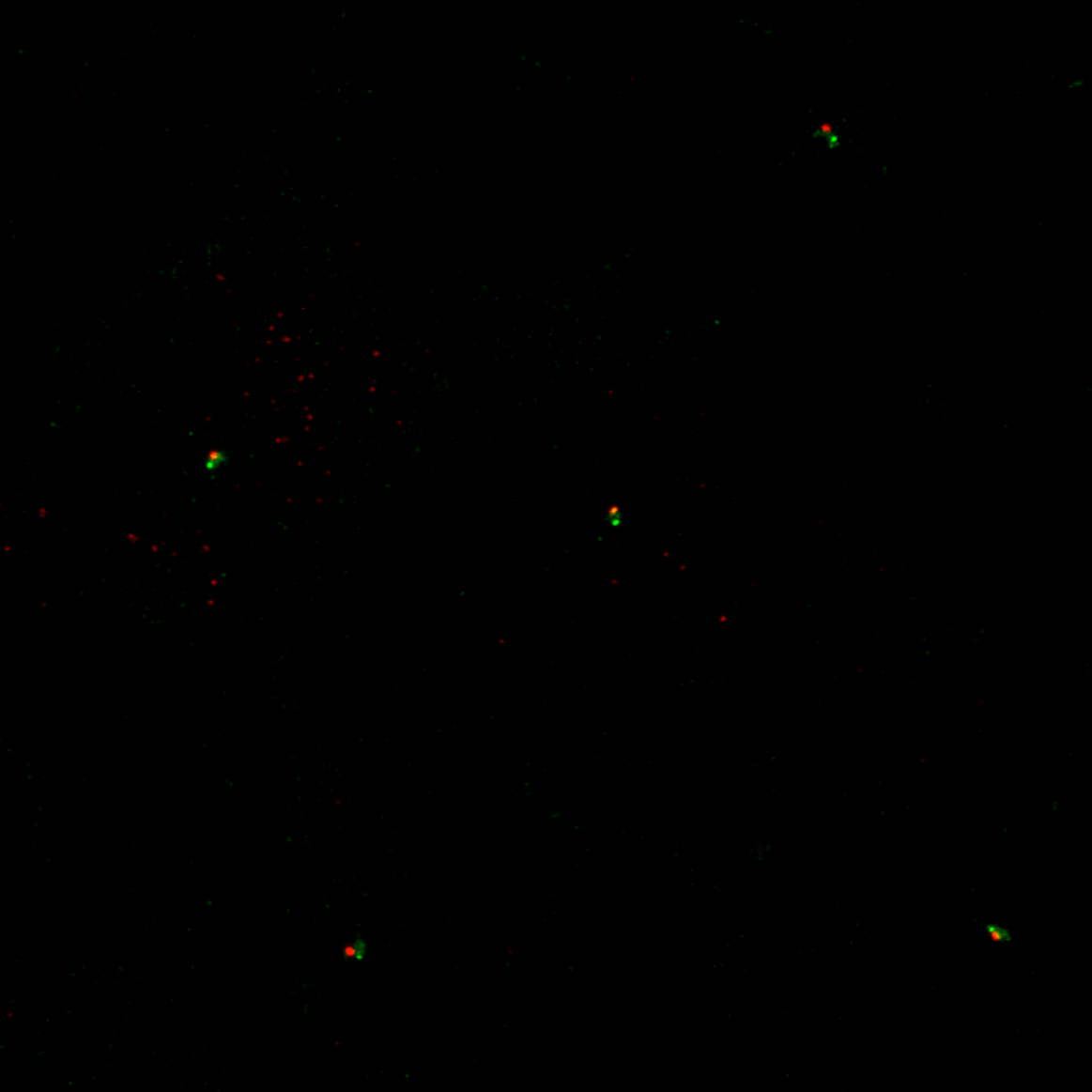

Supplement: Supplementary file 4 — Source data Fig. 3 [file 44319_2025_597_MOESM4_ESM.zip › Figure 3/3C/CEP164+CP110/siBICD2-1/SS24h.bmp]

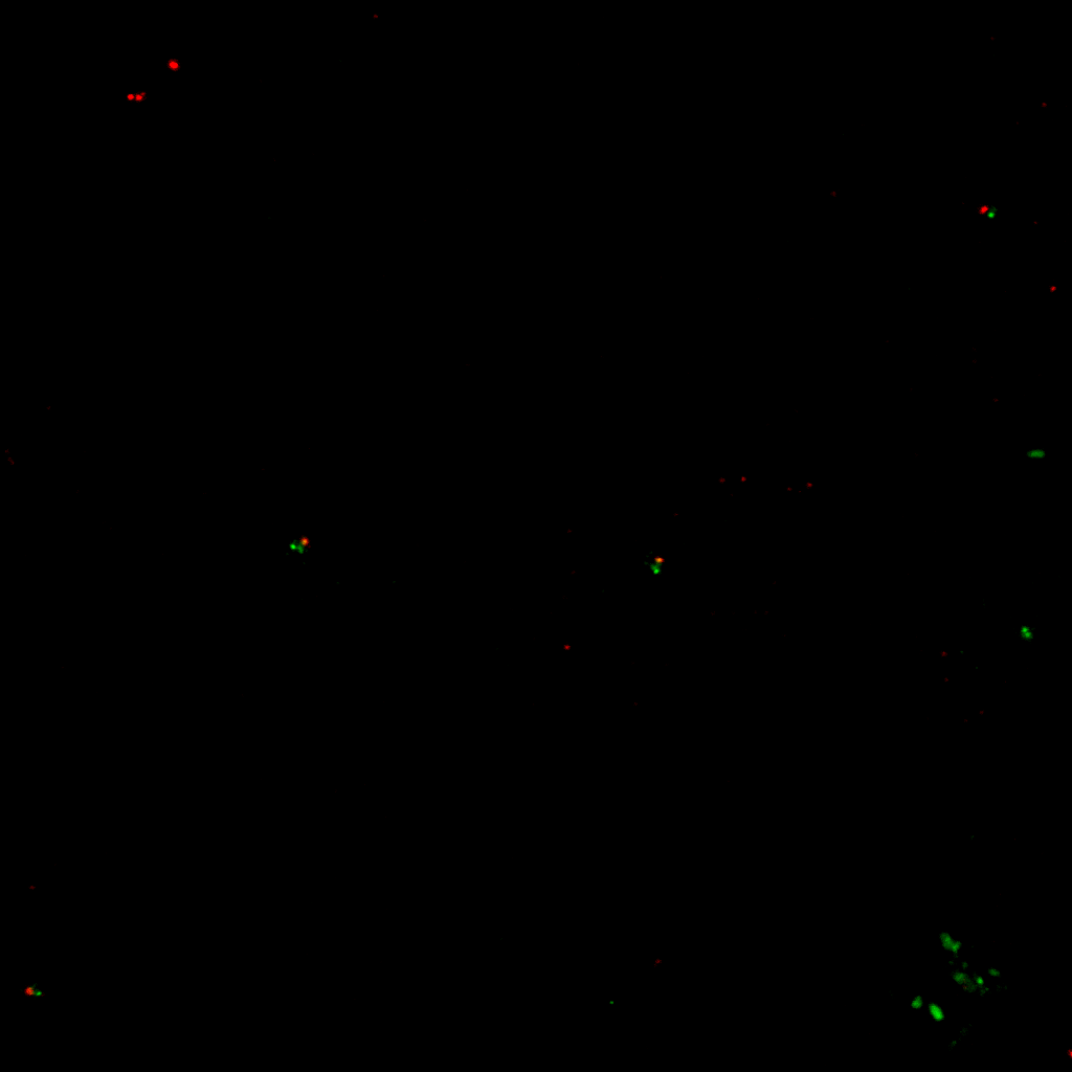

Supplement: Supplementary file 4 — Source data Fig. 3 [file 44319_2025_597_MOESM4_ESM.zip › Figure 3/3C/CEP164+CP110/siBICD2-1/SS3h.bmp]

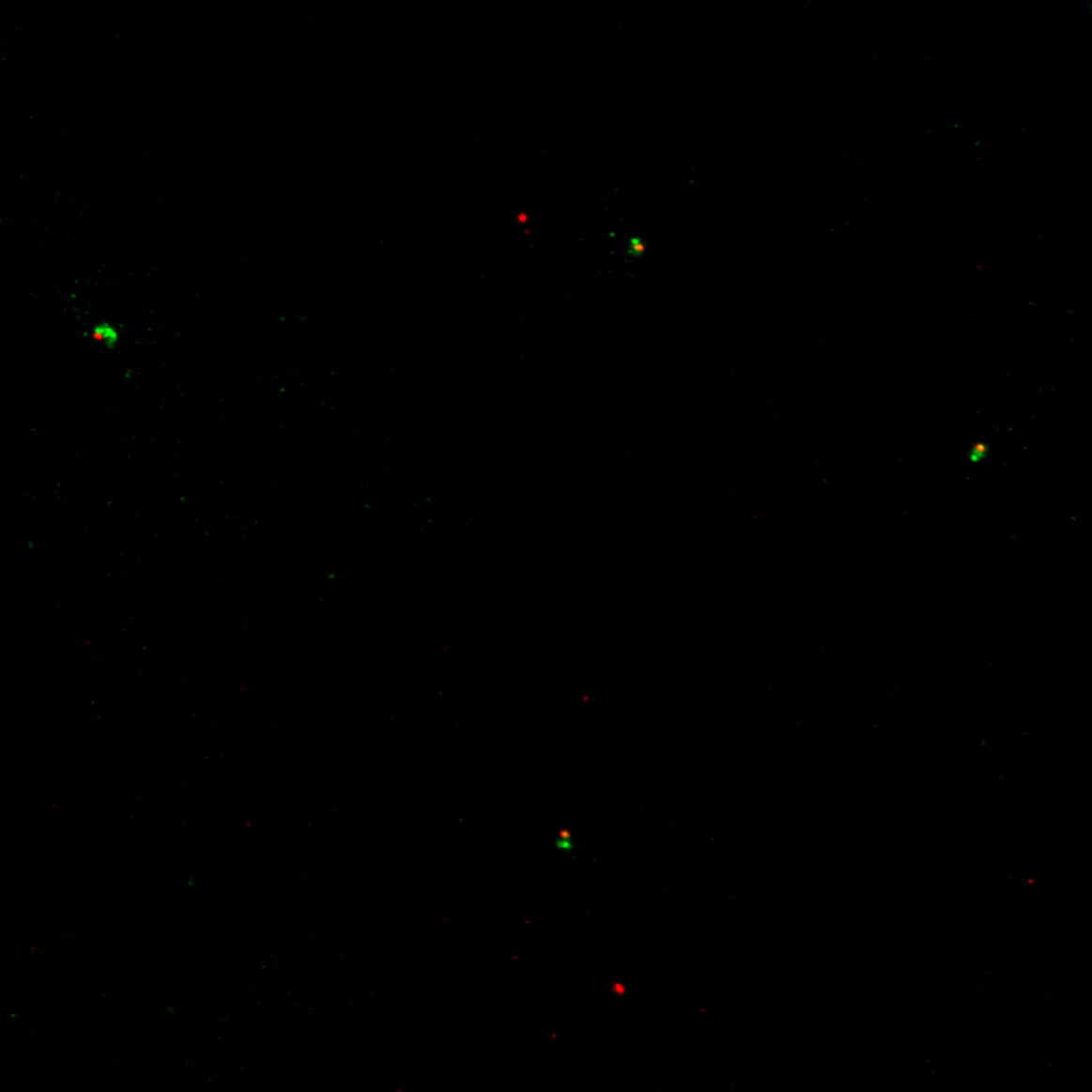

Supplement: Supplementary file 4 — Source data Fig. 3 [file 44319_2025_597_MOESM4_ESM.zip › Figure 3/3C/CEP164+CP110/siBICD2-1/SS6h.bmp]

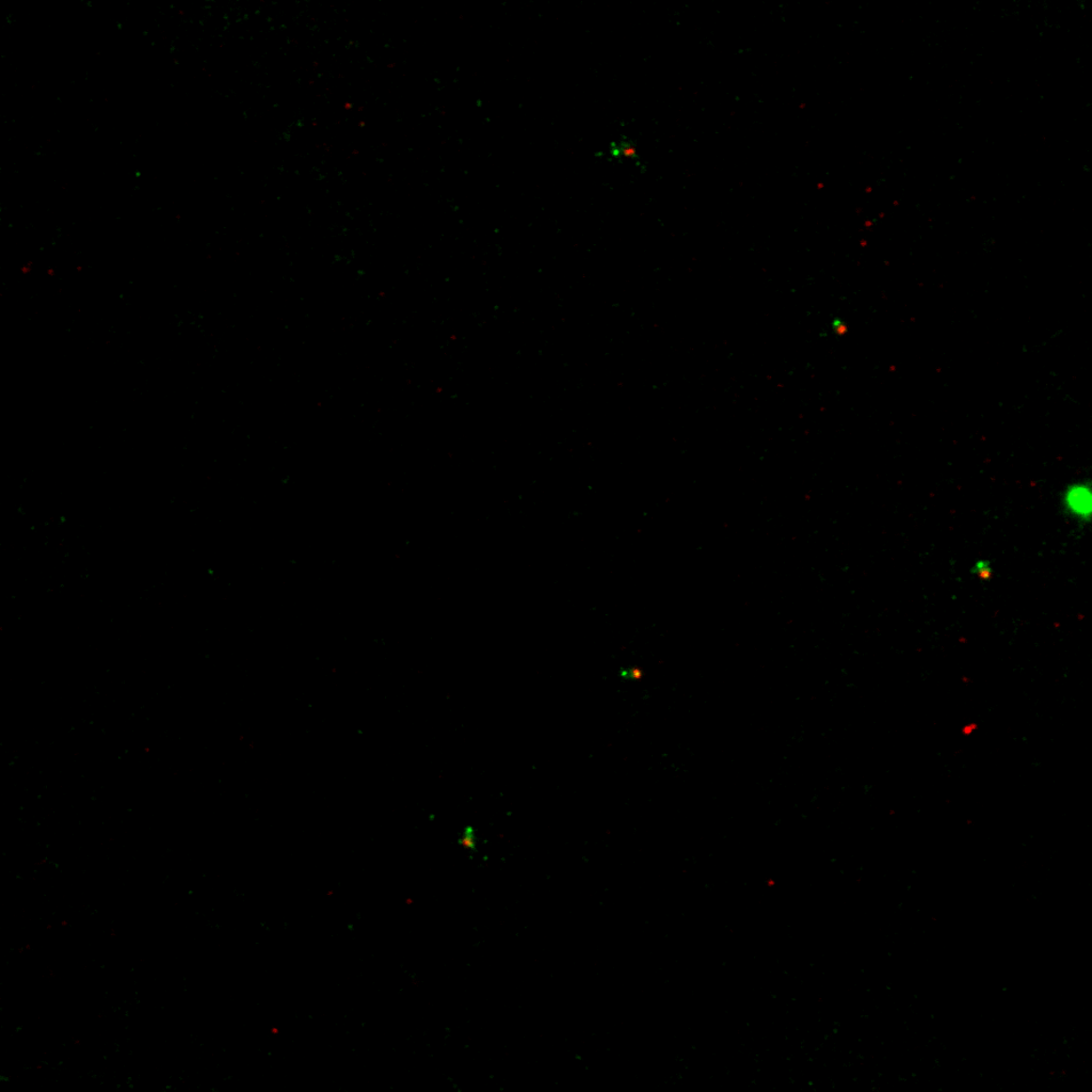

Supplement: Supplementary file 4 — Source data Fig. 3 [file 44319_2025_597_MOESM4_ESM.zip › Figure 3/3C/CEP164+CP110/siBICD2-1/SS9h.bmp]

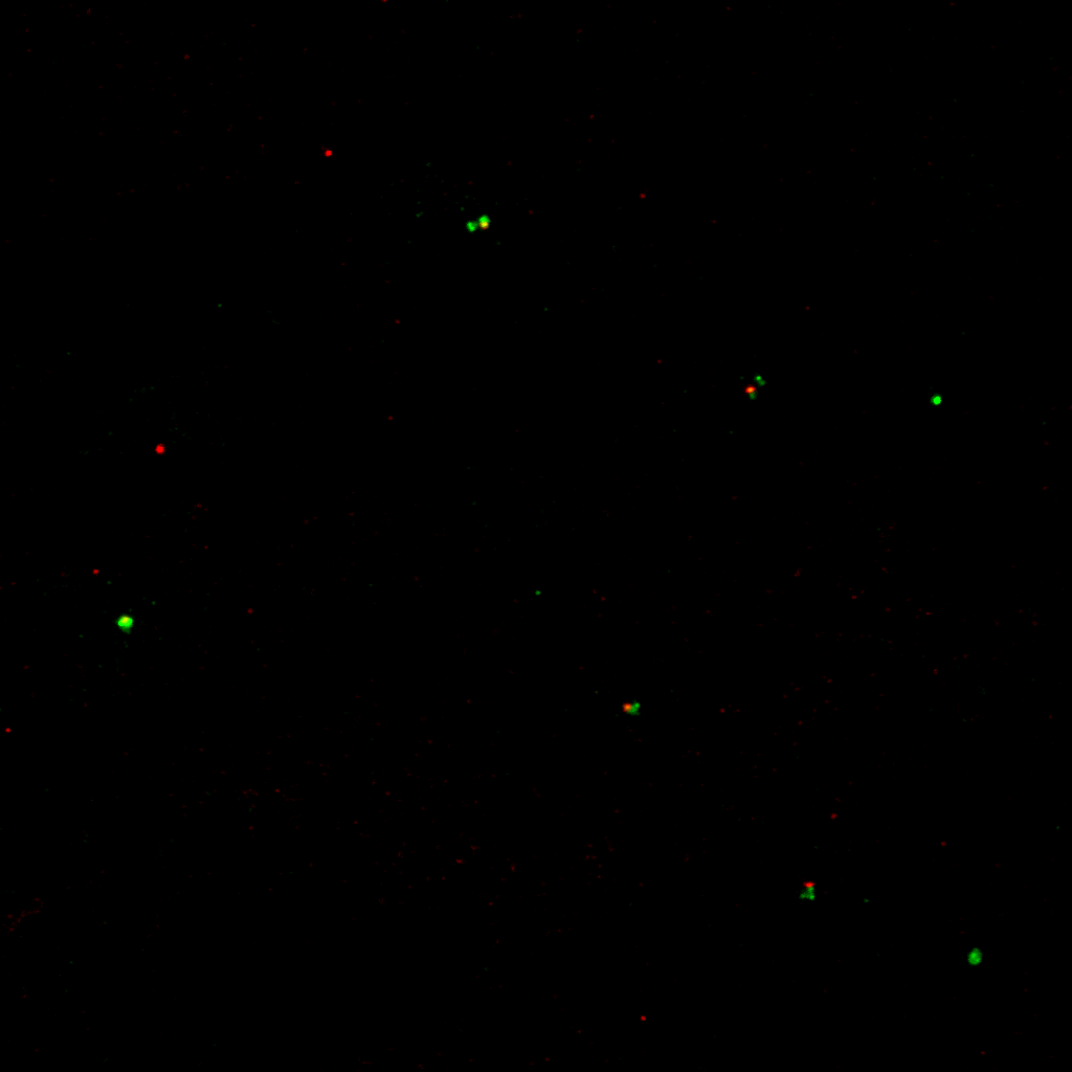

Supplement: Supplementary file 4 — Source data Fig. 3 [file 44319_2025_597_MOESM4_ESM.zip › Figure 3/3C/CEP164+CP110/siNC/SS0h.bmp]

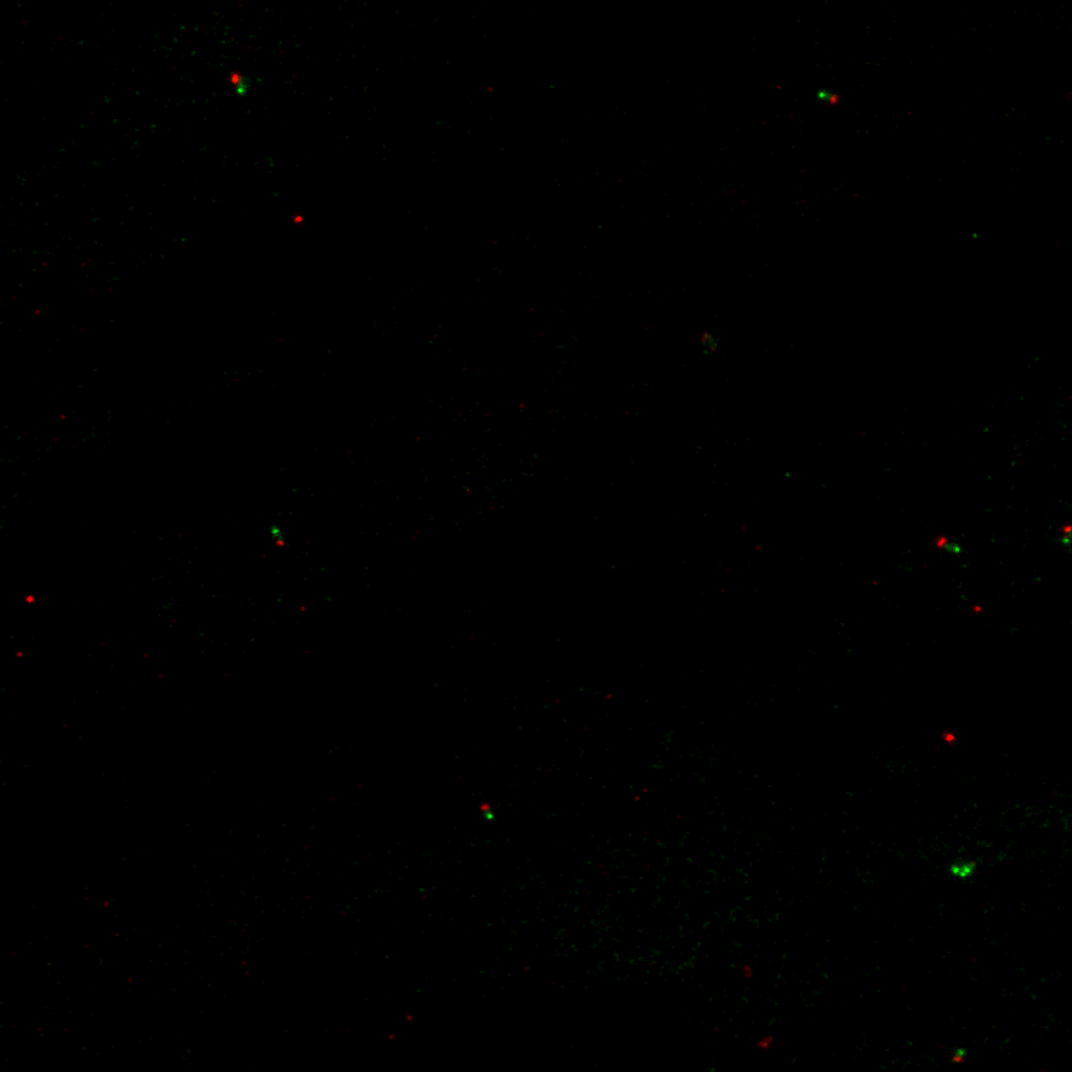

Supplement: Supplementary file 4 — Source data Fig. 3 [file 44319_2025_597_MOESM4_ESM.zip › Figure 3/3C/CEP164+CP110/siNC/SS12h.bmp]

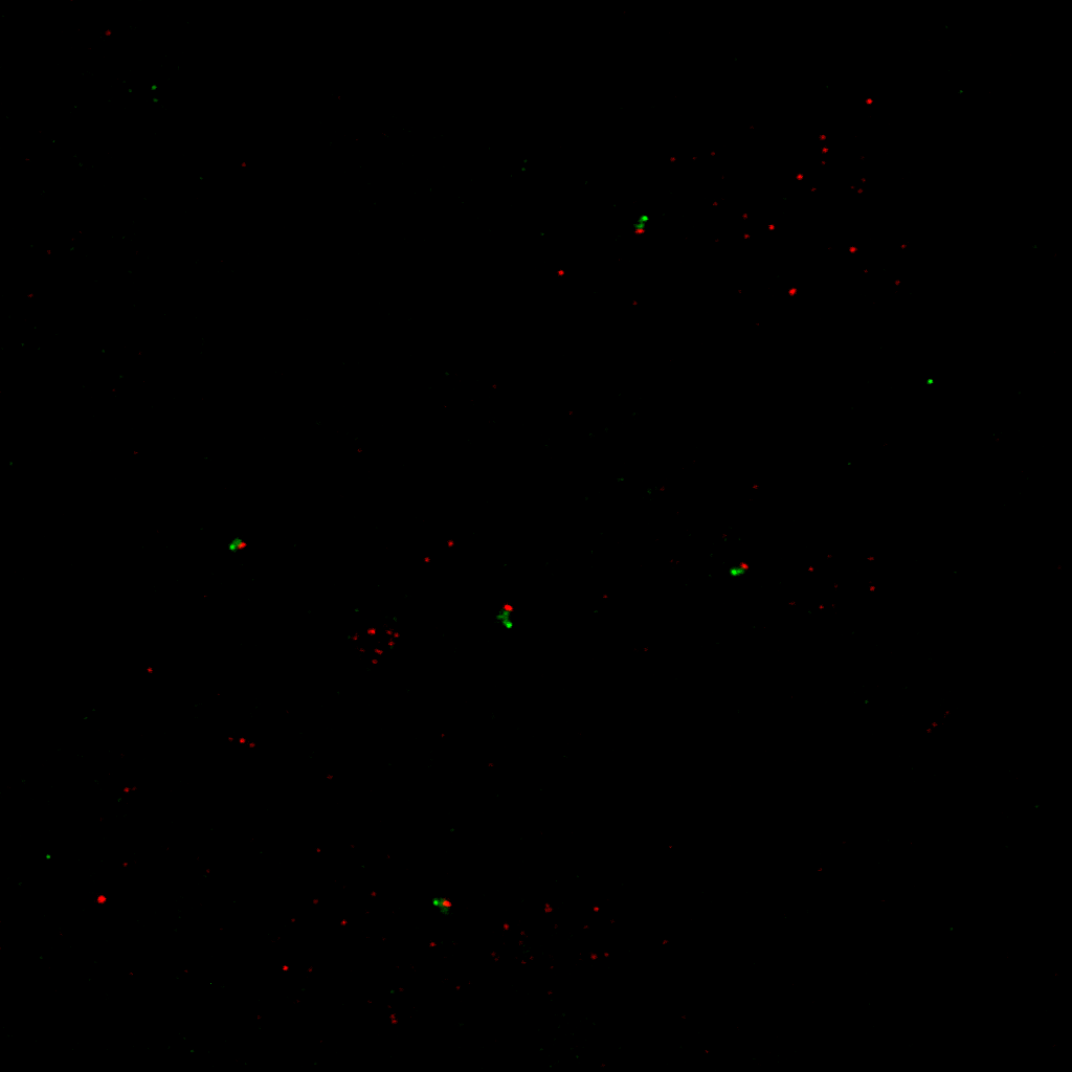

Supplement: Supplementary file 4 — Source data Fig. 3 [file 44319_2025_597_MOESM4_ESM.zip › Figure 3/3C/CEP164+CP110/siNC/SS15h.bmp]

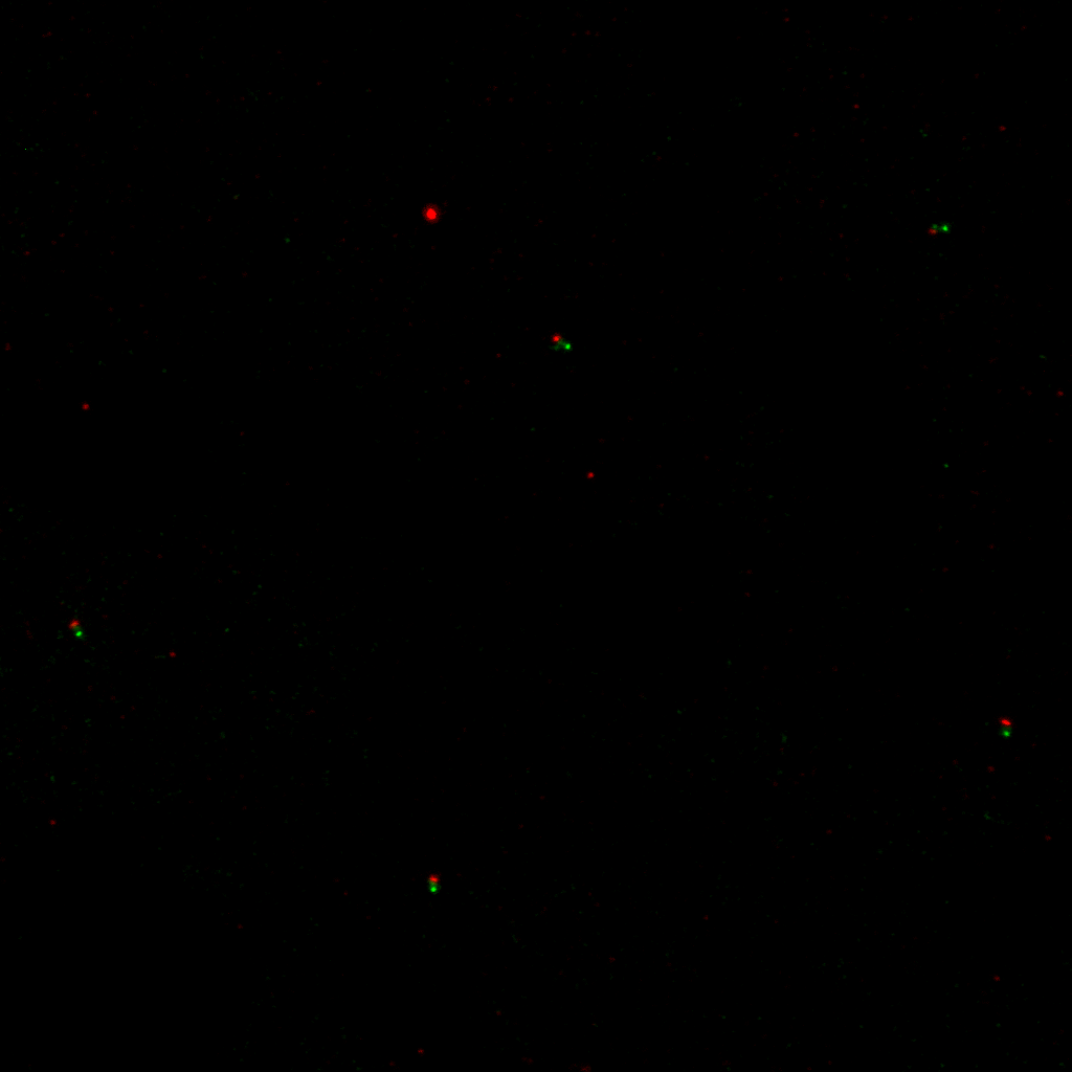

Supplement: Supplementary file 4 — Source data Fig. 3 [file 44319_2025_597_MOESM4_ESM.zip › Figure 3/3C/CEP164+CP110/siNC/SS24h.bmp]

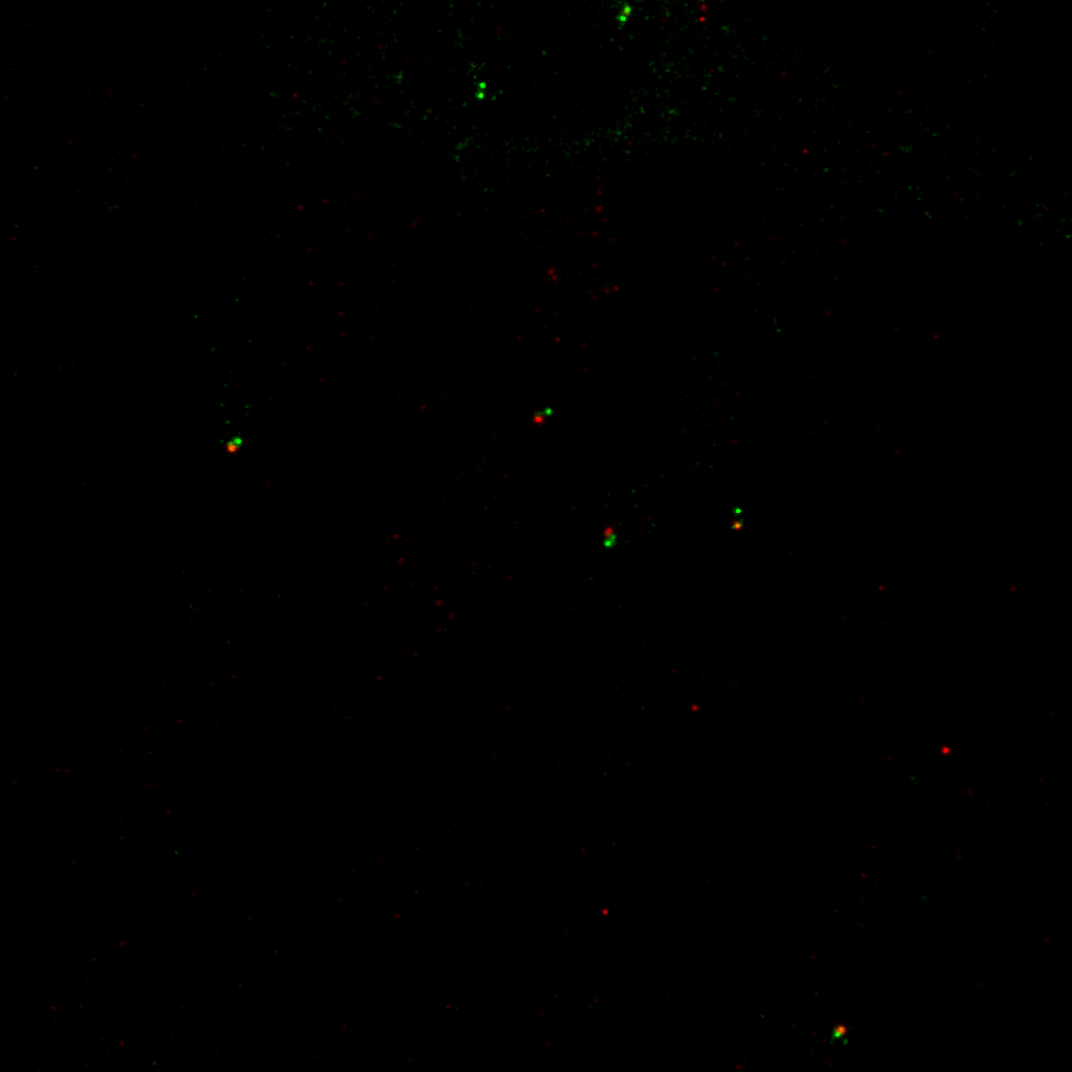

Supplement: Supplementary file 4 — Source data Fig. 3 [file 44319_2025_597_MOESM4_ESM.zip › Figure 3/3C/CEP164+CP110/siNC/SS3h.bmp]

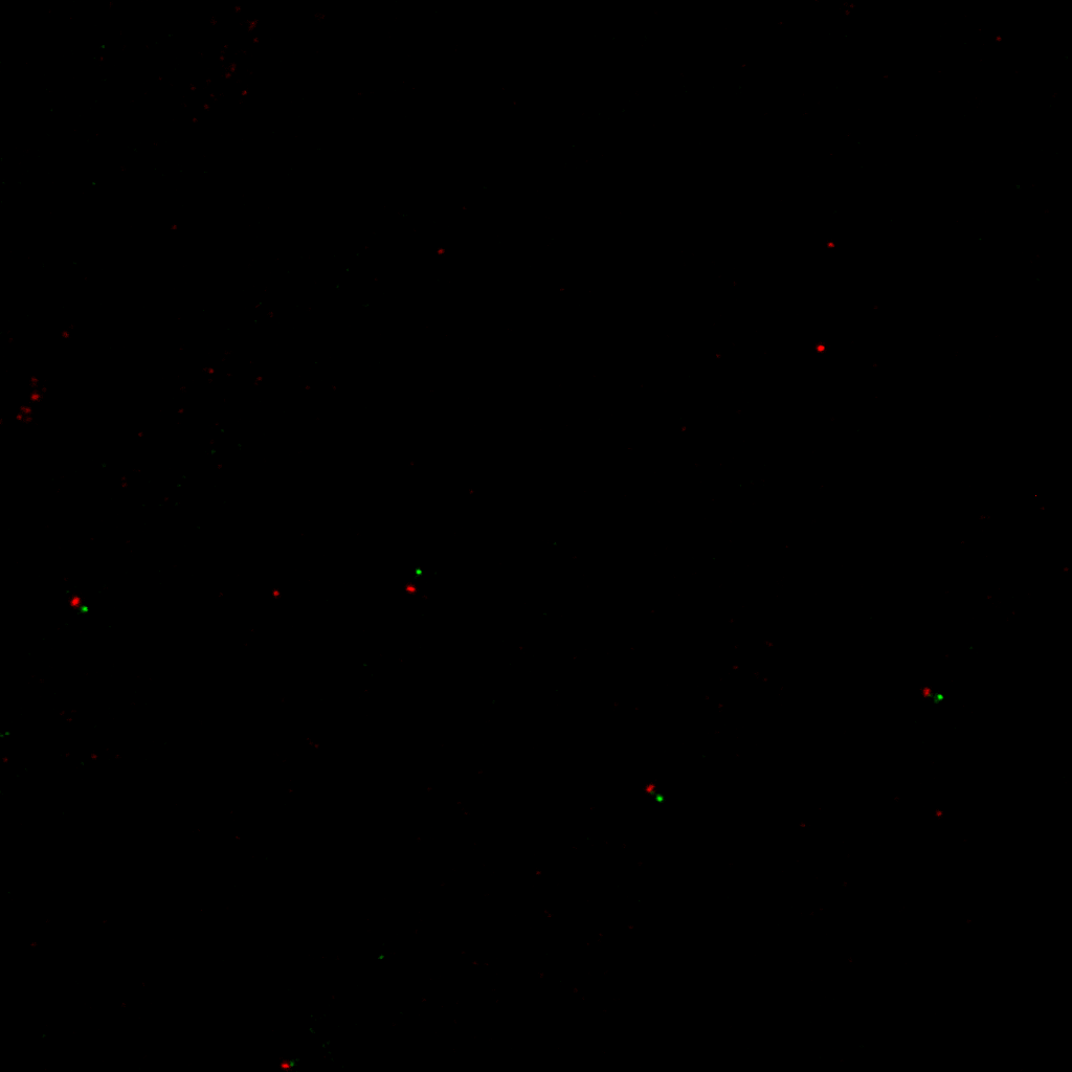

Supplement: Supplementary file 4 — Source data Fig. 3 [file 44319_2025_597_MOESM4_ESM.zip › Figure 3/3C/CEP164+CP110/siNC/SS6h.bmp]

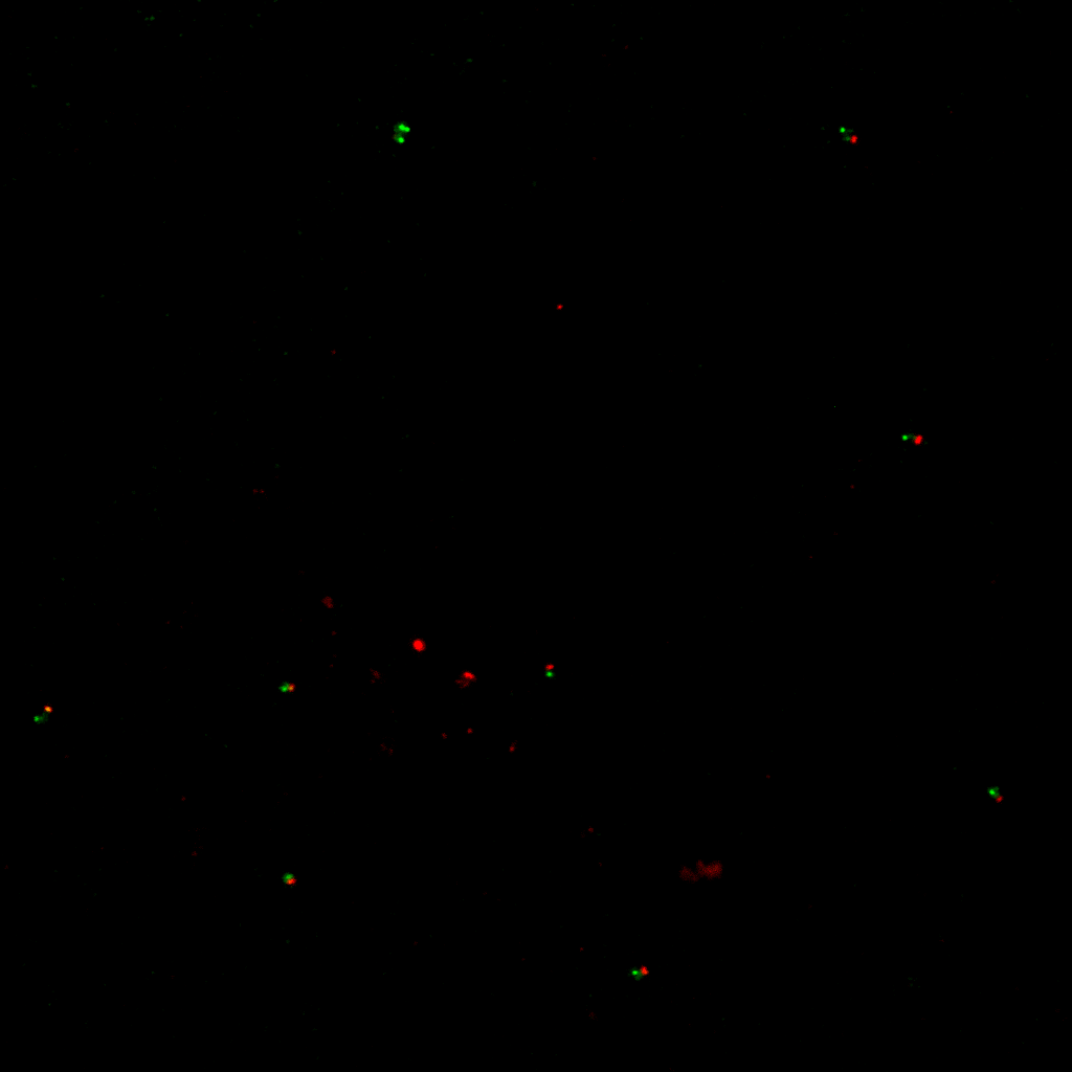

Supplement: Supplementary file 4 — Source data Fig. 3 [file 44319_2025_597_MOESM4_ESM.zip › Figure 3/3C/CEP164+CP110/siNC/SS9h.bmp]

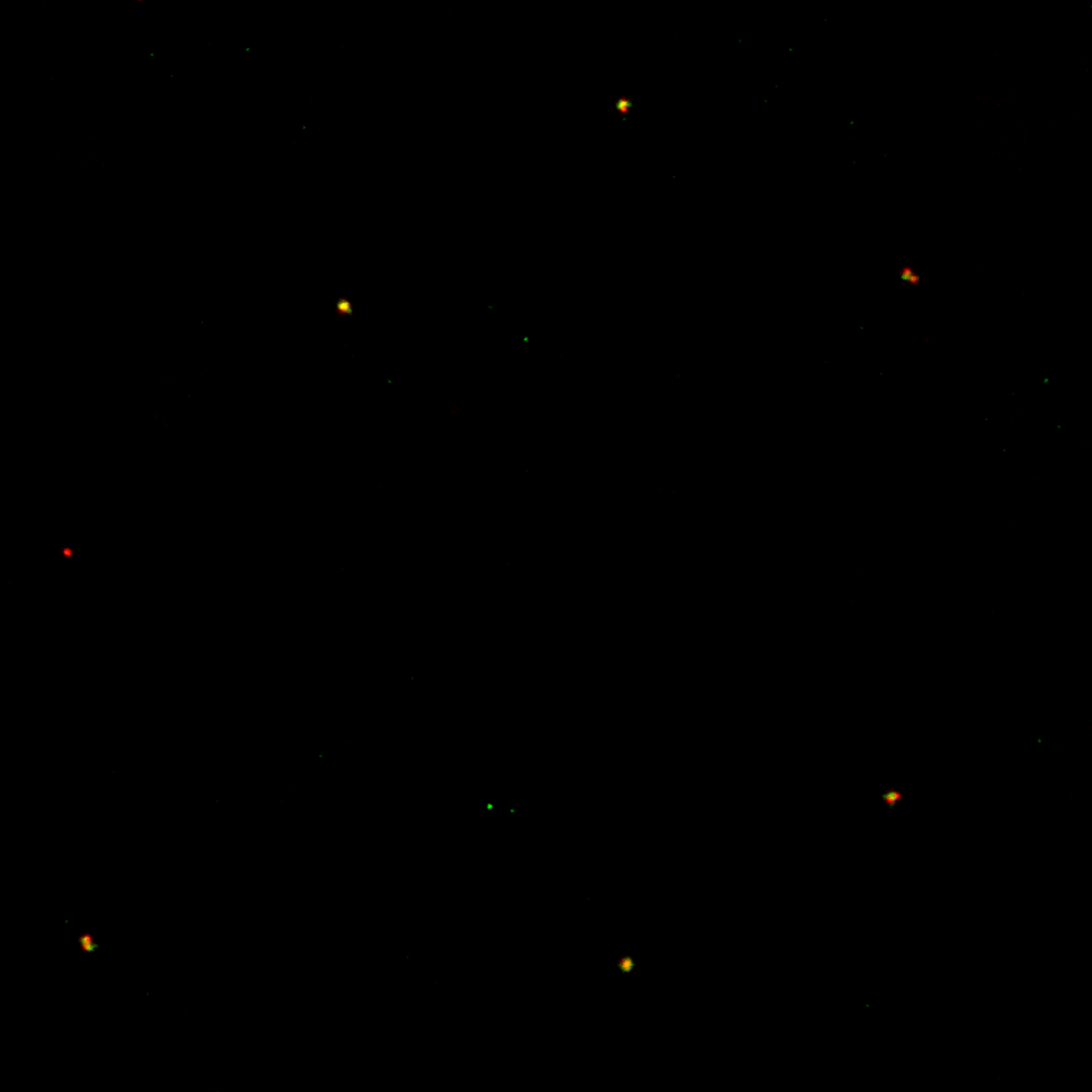

Supplement: Supplementary file 4 — Source data Fig. 3 [file 44319_2025_597_MOESM4_ESM.zip › Figure 3/3C/r-tu+Arl13b/siBICD2-1/SS0h.bmp]

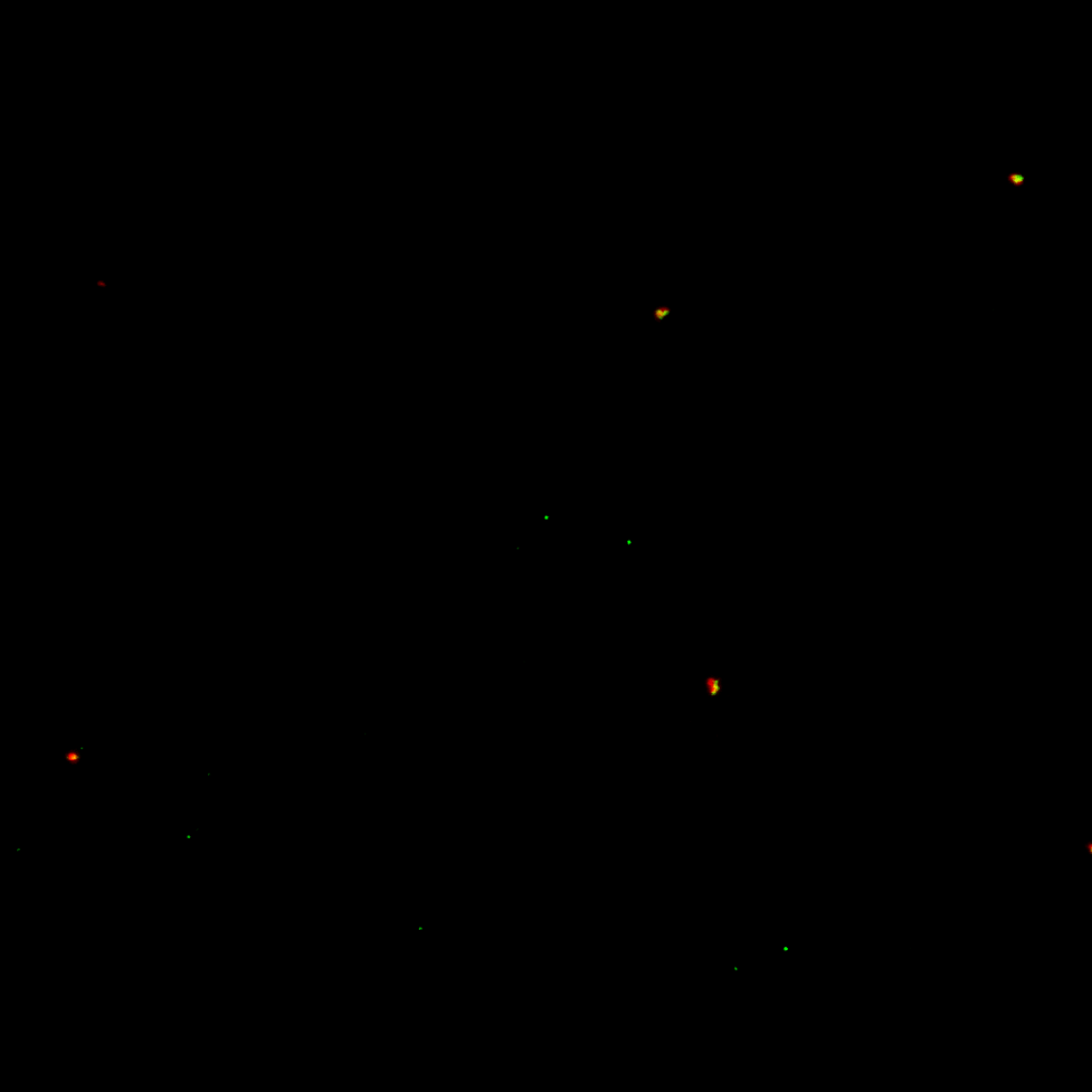

Supplement: Supplementary file 4 — Source data Fig. 3 [file 44319_2025_597_MOESM4_ESM.zip › Figure 3/3C/r-tu+Arl13b/siBICD2-1/SS12h.bmp]

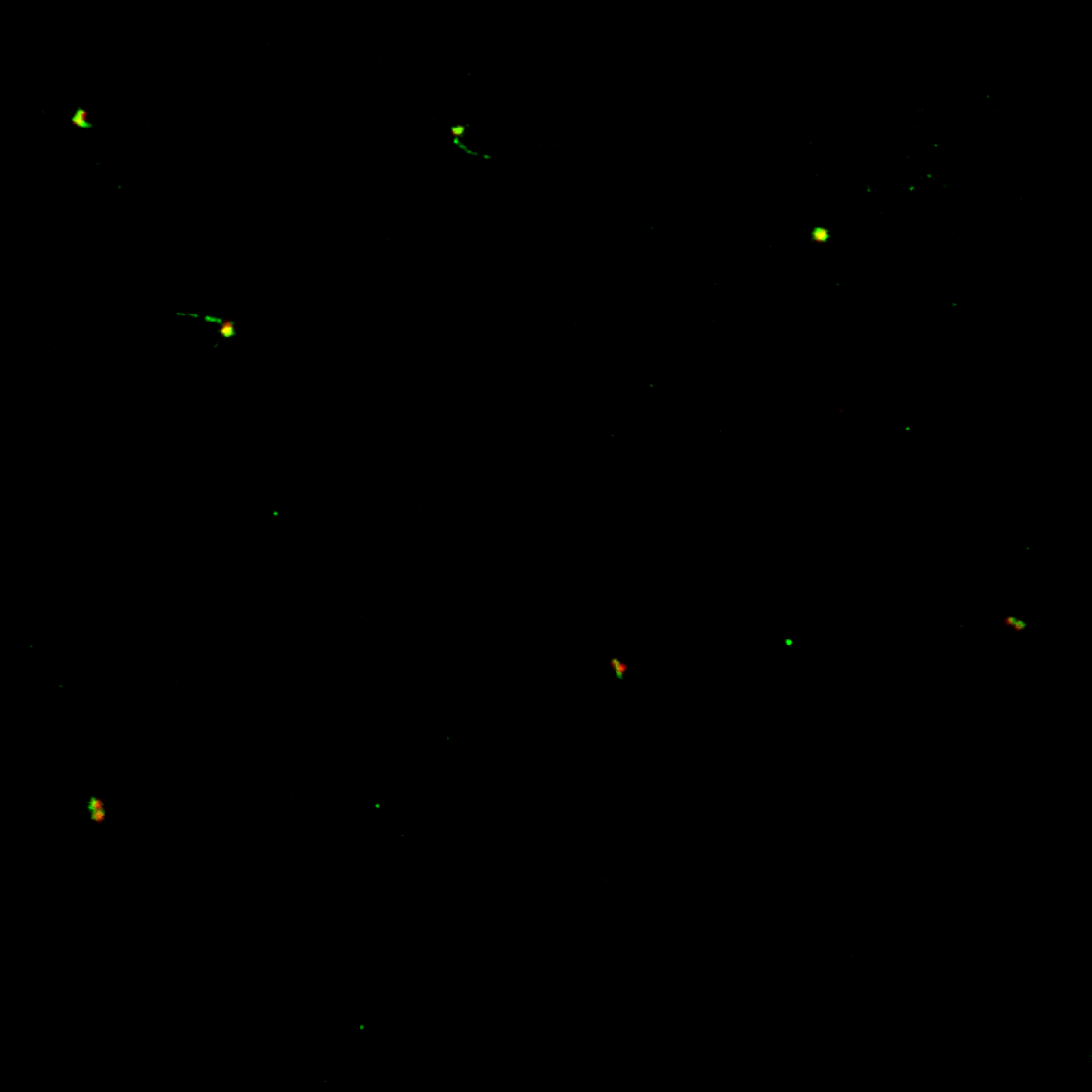

Supplement: Supplementary file 4 — Source data Fig. 3 [file 44319_2025_597_MOESM4_ESM.zip › Figure 3/3C/r-tu+Arl13b/siBICD2-1/SS15h.bmp]
