# Supplementary figures and images for: BICD2 promotes ciliogenesis by facilitating CP110 removal from the mother centriole (part 2 of 2)
Source: EMBO Rep. 2025 Oct 16;26(22):5567–88. doi: 10.1038/s44319-025-00597-0 (PMC12635215; doi:10.1038/s44319-025-00597-0)

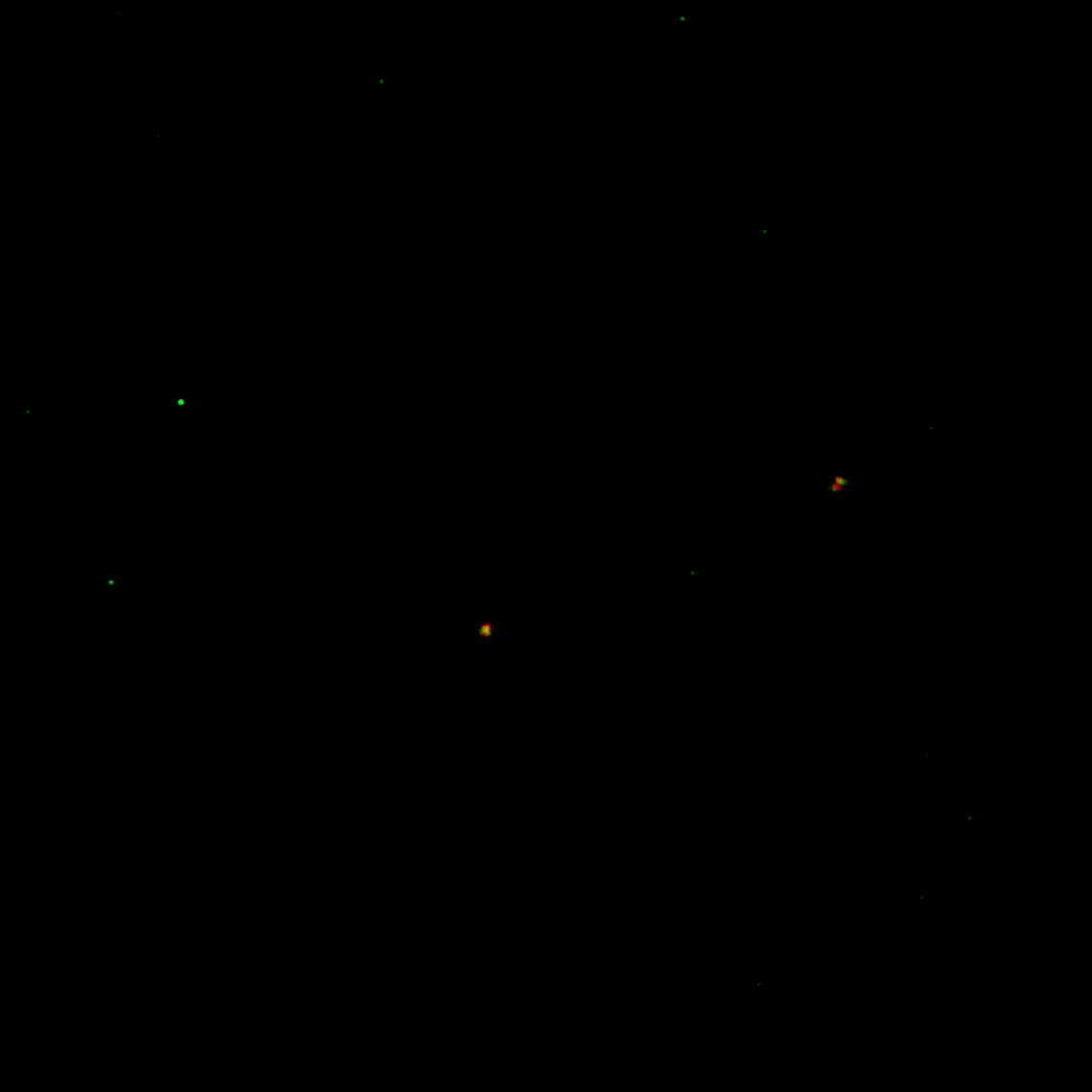

Supplement: Supplementary file 4 — Source data Fig. 3 [file 44319_2025_597_MOESM4_ESM.zip › Figure 3/3C/r-tu+Arl13b/siBICD2-1/SS24h.bmp]

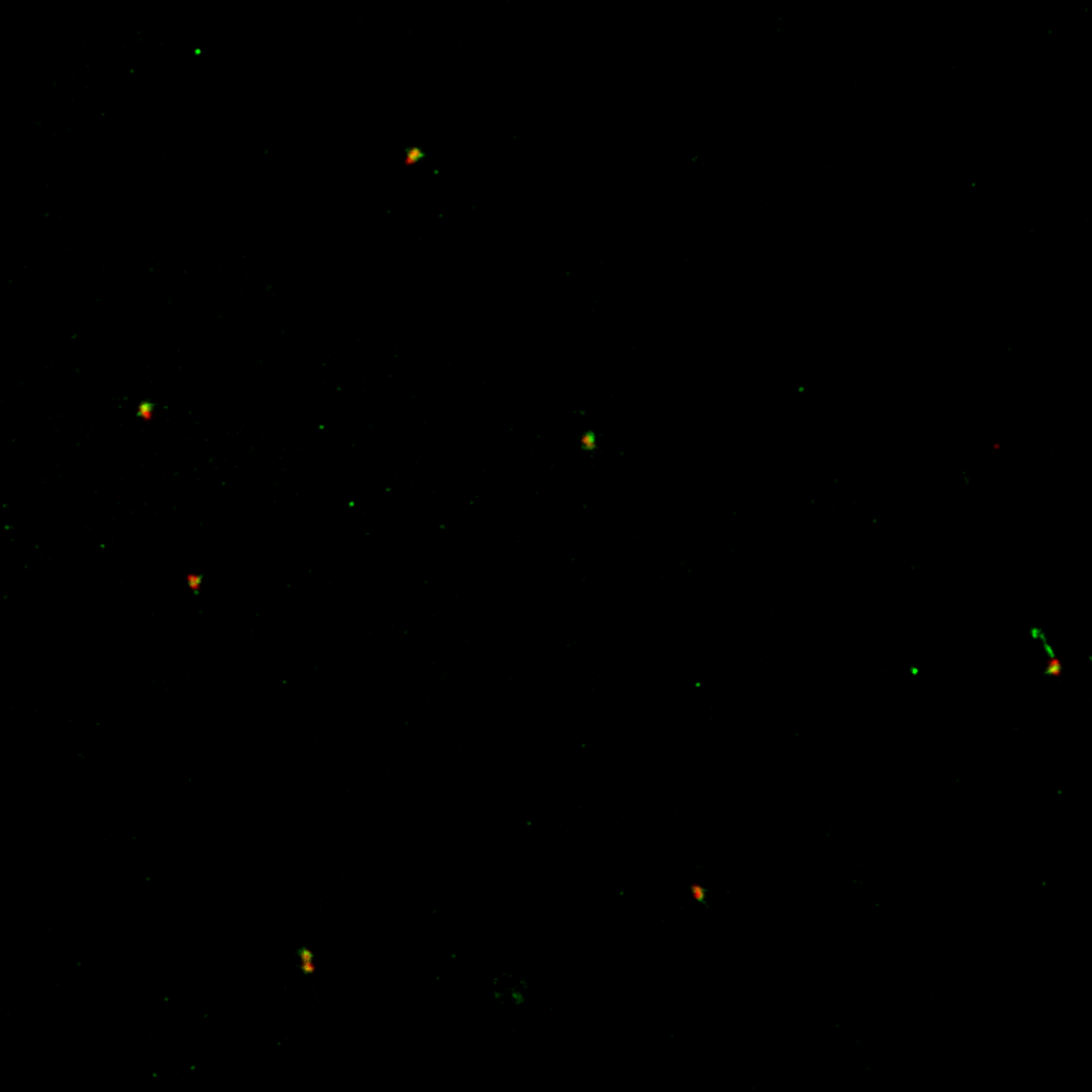

Supplement: Supplementary file 4 — Source data Fig. 3 [file 44319_2025_597_MOESM4_ESM.zip › Figure 3/3C/r-tu+Arl13b/siBICD2-1/SS3h.bmp]

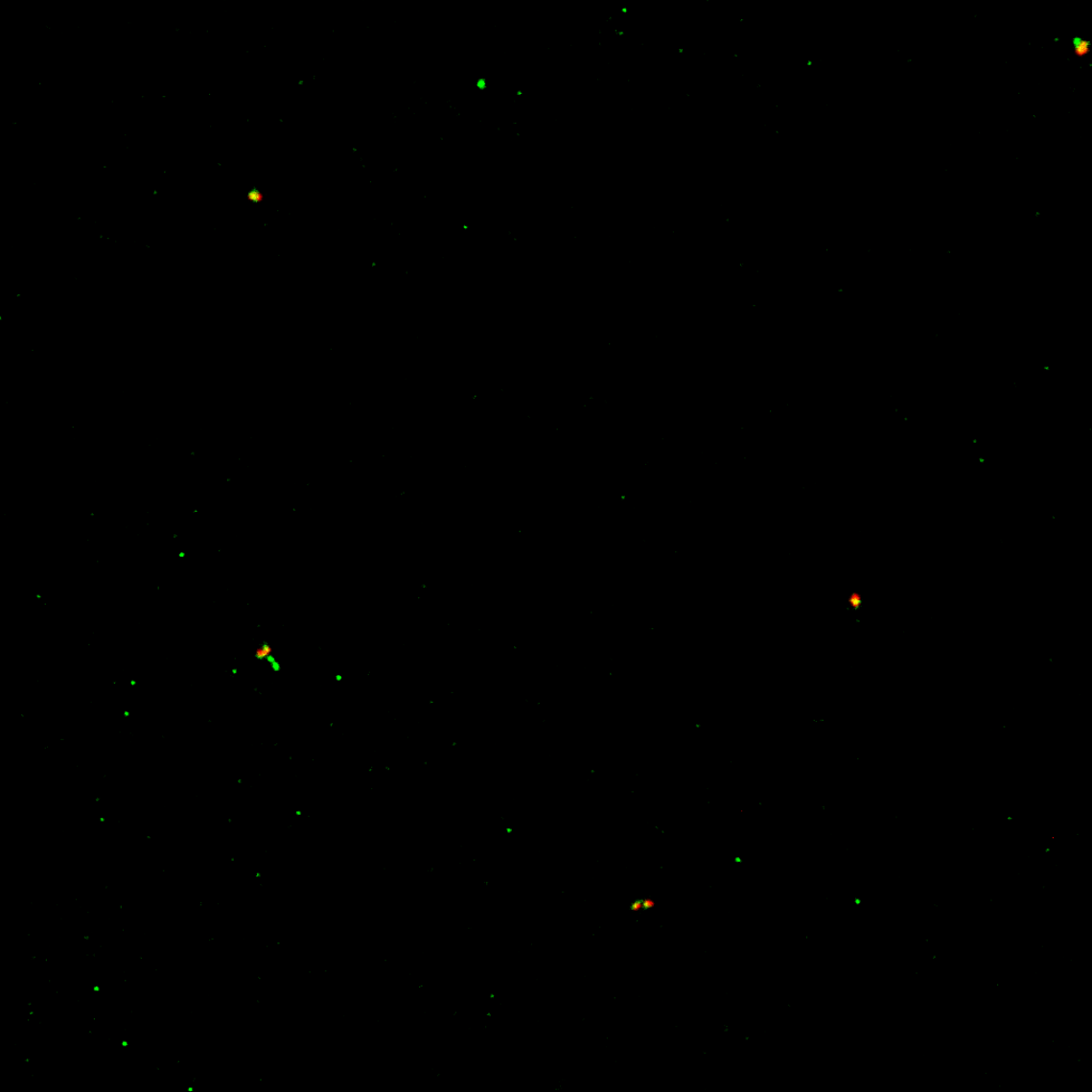

Supplement: Supplementary file 4 — Source data Fig. 3 [file 44319_2025_597_MOESM4_ESM.zip › Figure 3/3C/r-tu+Arl13b/siBICD2-1/SS6h.bmp]

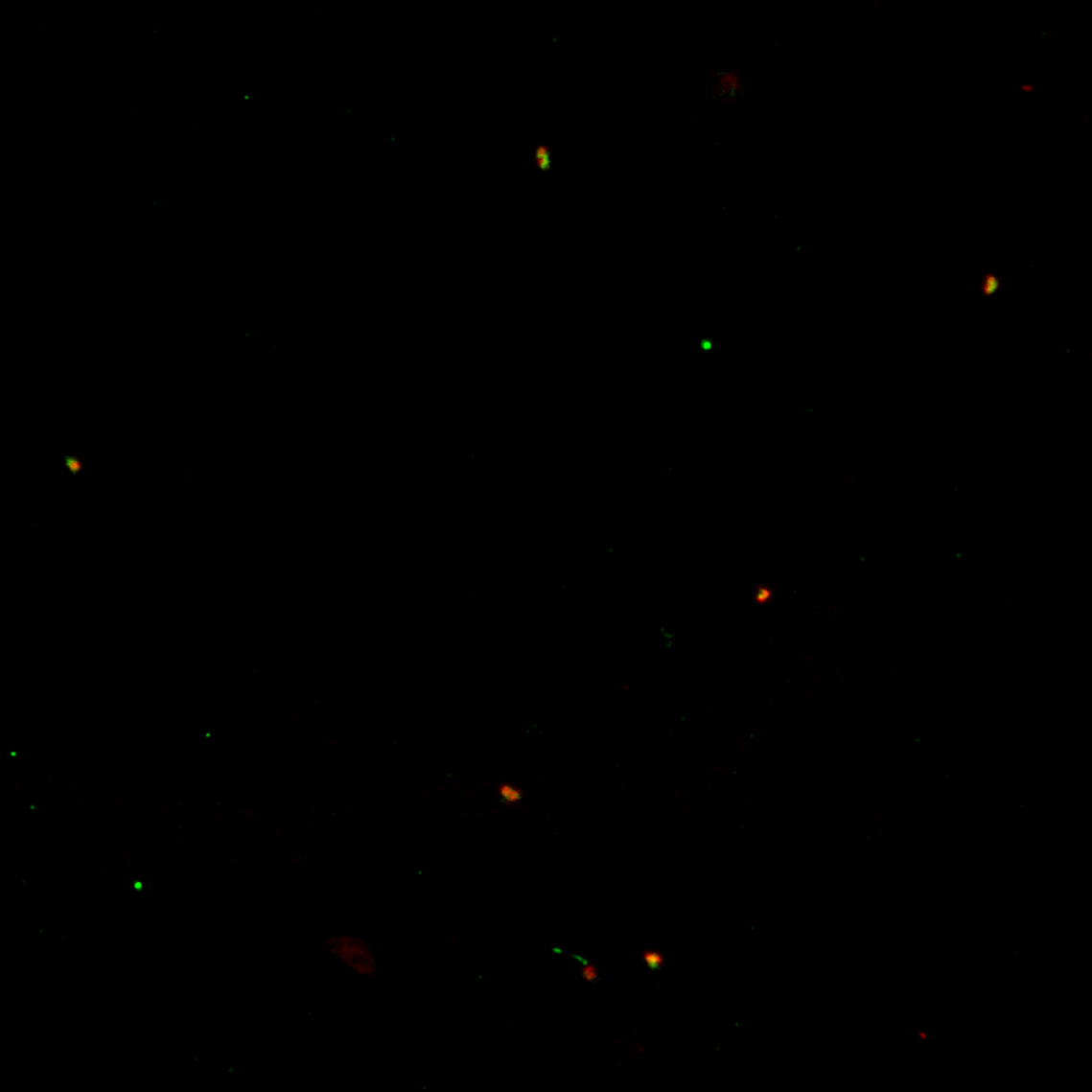

Supplement: Supplementary file 4 — Source data Fig. 3 [file 44319_2025_597_MOESM4_ESM.zip › Figure 3/3C/r-tu+Arl13b/siBICD2-1/SS9h.bmp]

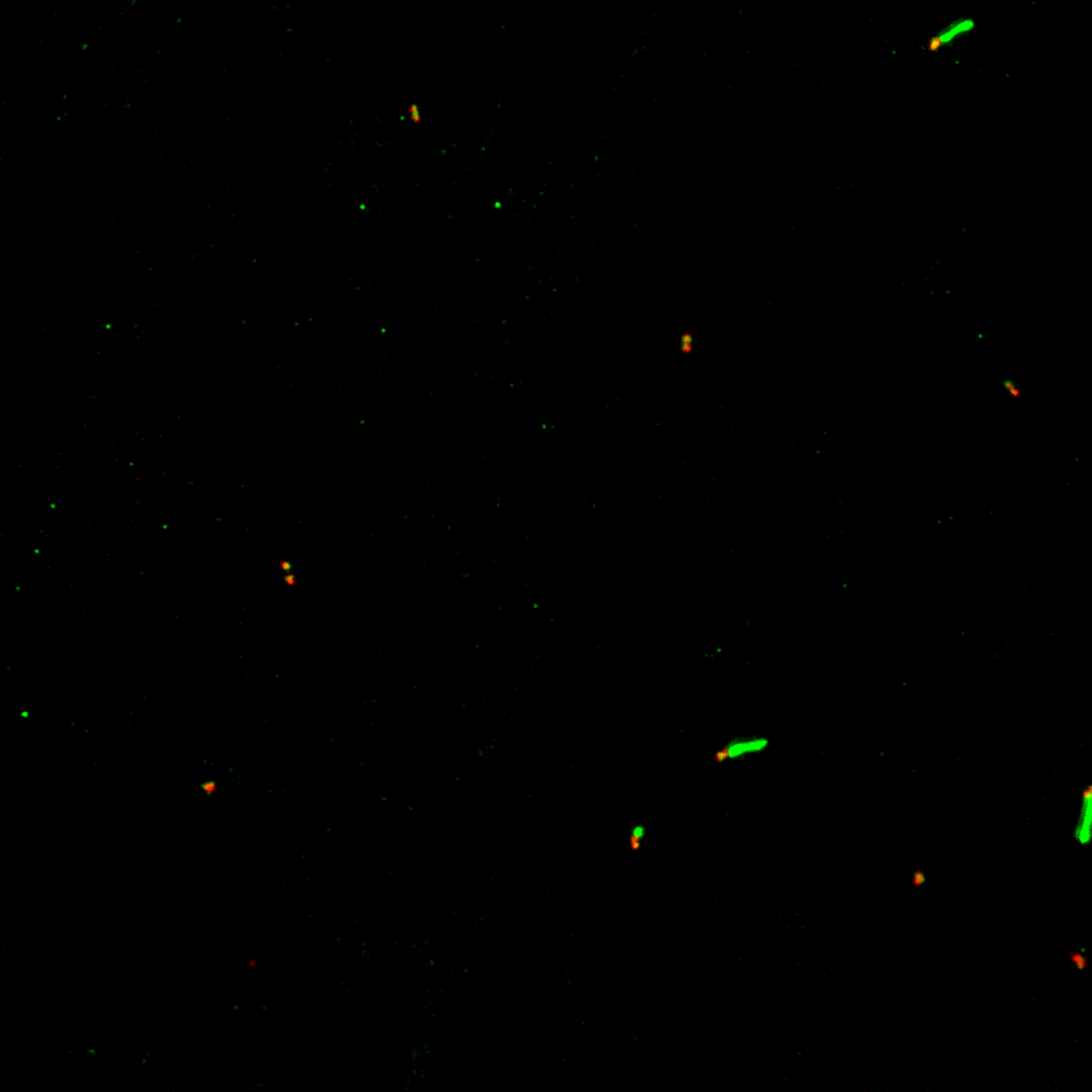

Supplement: Supplementary file 4 — Source data Fig. 3 [file 44319_2025_597_MOESM4_ESM.zip › Figure 3/3C/r-tu+Arl13b/siNC/SS0h.bmp]

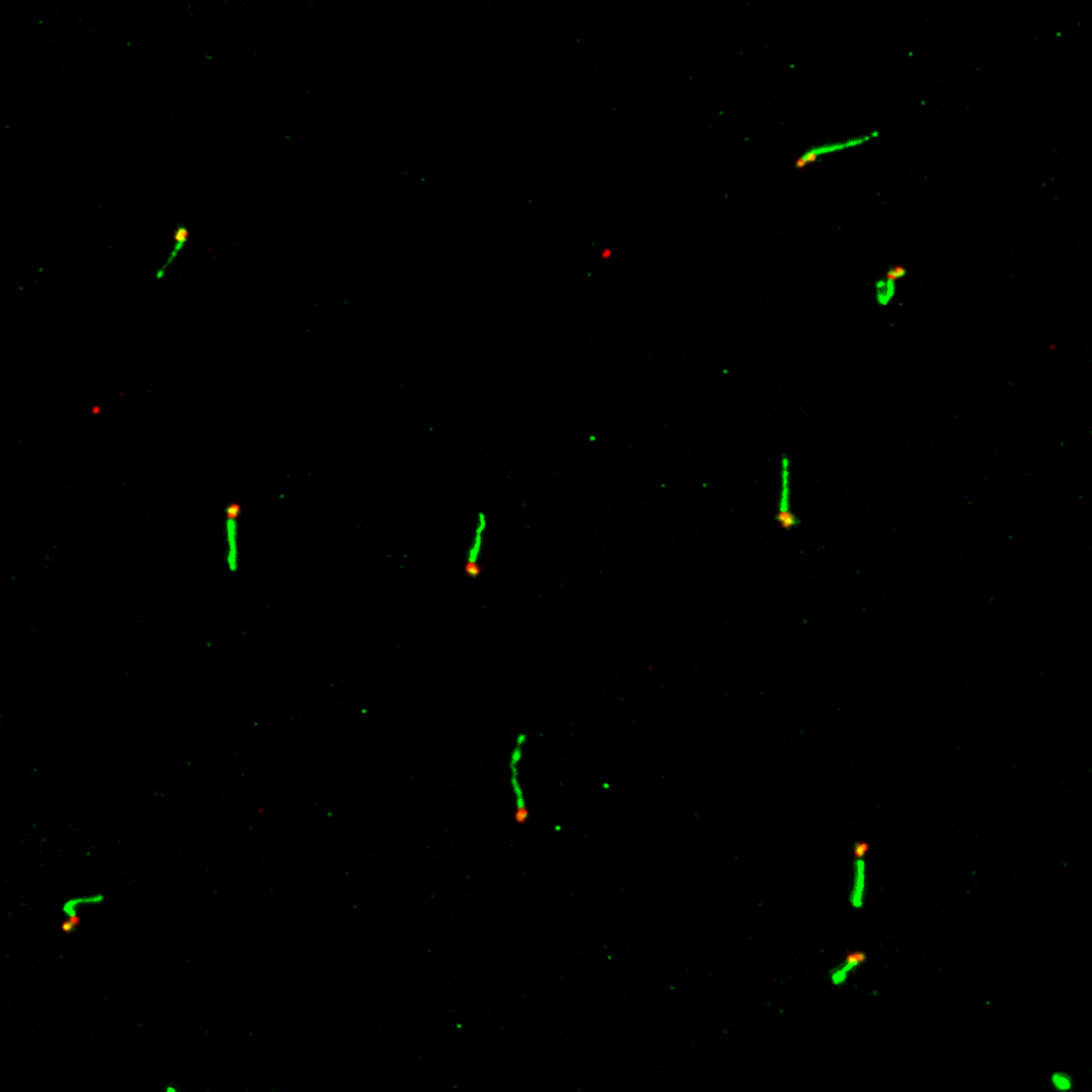

Supplement: Supplementary file 4 — Source data Fig. 3 [file 44319_2025_597_MOESM4_ESM.zip › Figure 3/3C/r-tu+Arl13b/siNC/SS12h.bmp]

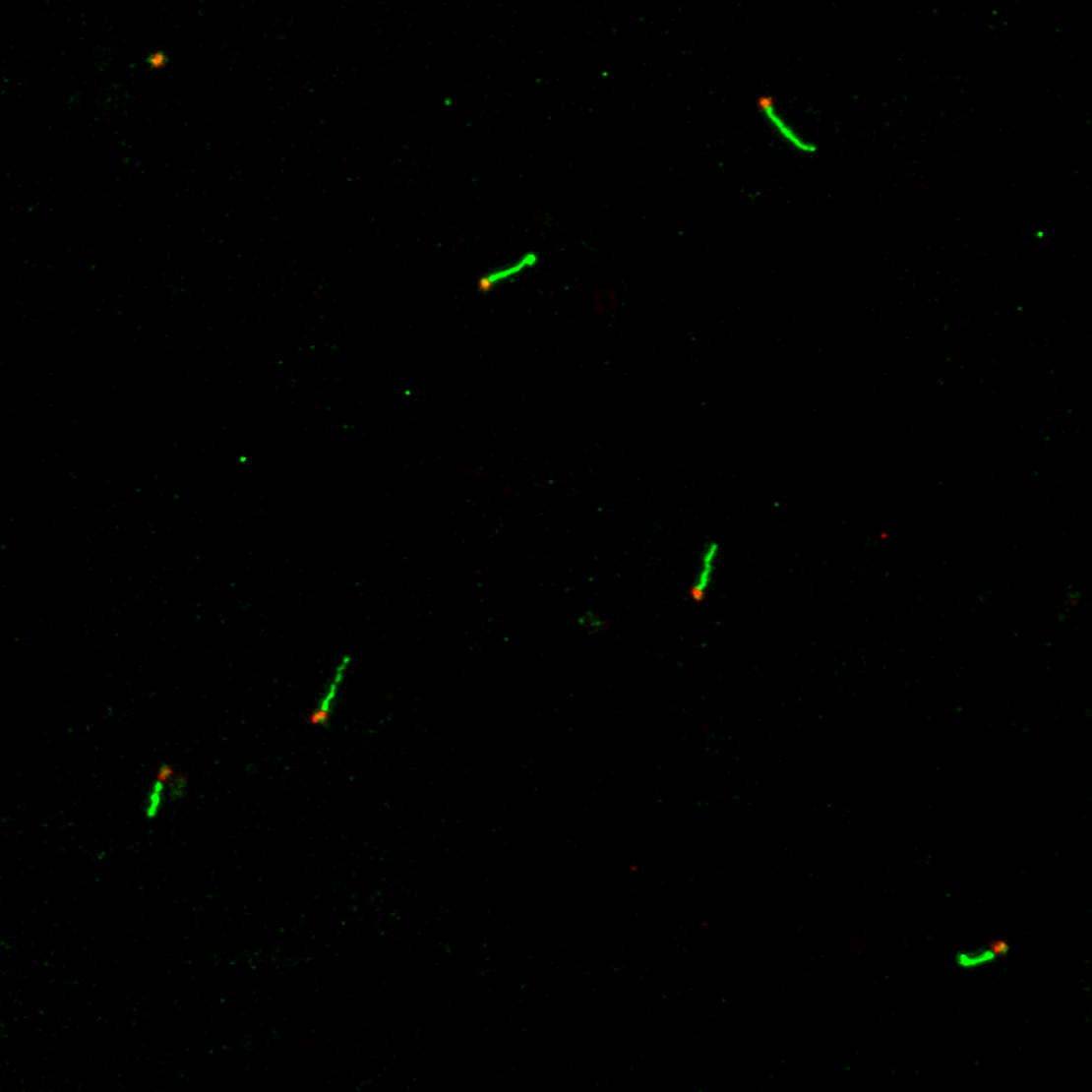

Supplement: Supplementary file 4 — Source data Fig. 3 [file 44319_2025_597_MOESM4_ESM.zip › Figure 3/3C/r-tu+Arl13b/siNC/SS15h.bmp]

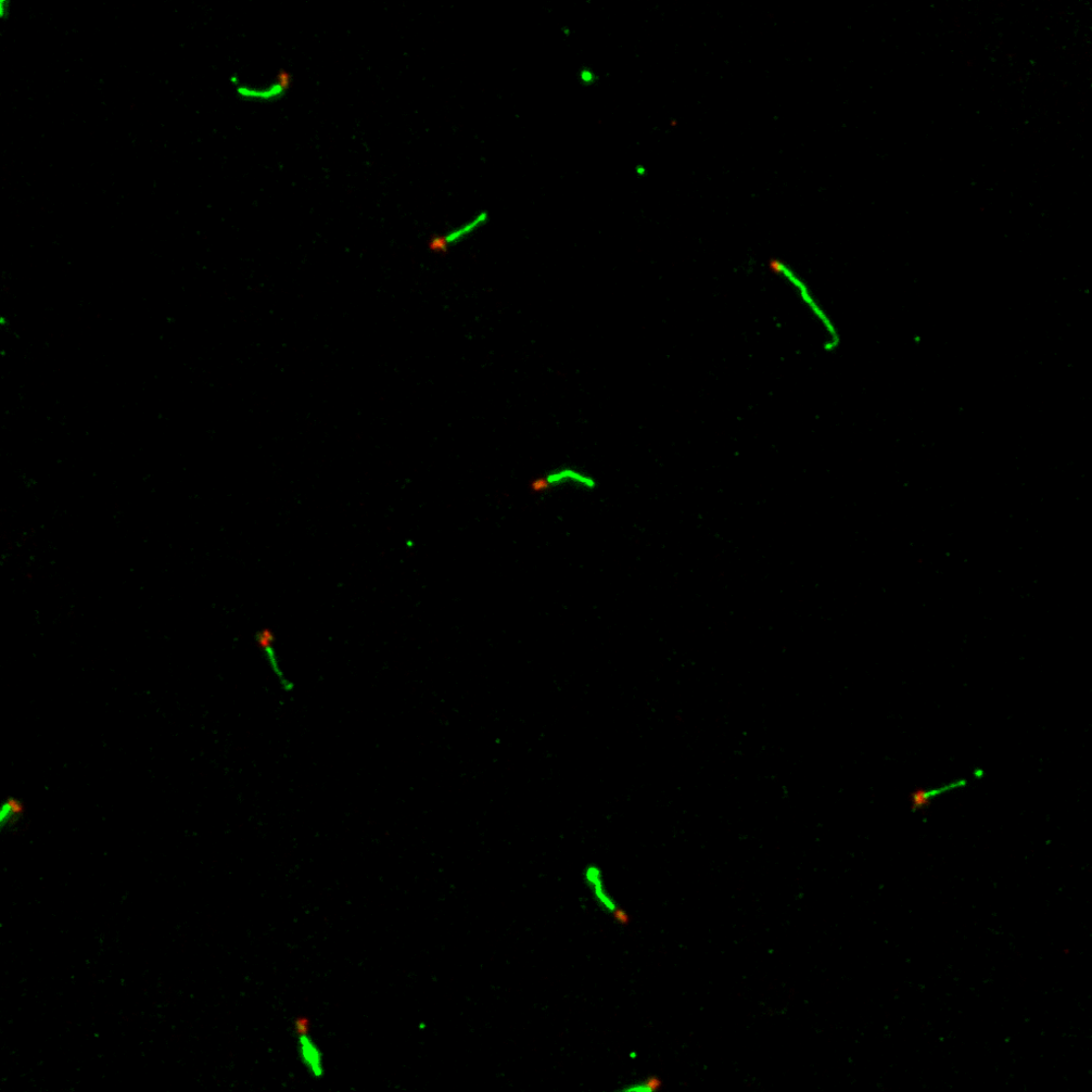

Supplement: Supplementary file 4 — Source data Fig. 3 [file 44319_2025_597_MOESM4_ESM.zip › Figure 3/3C/r-tu+Arl13b/siNC/SS24h.bmp]

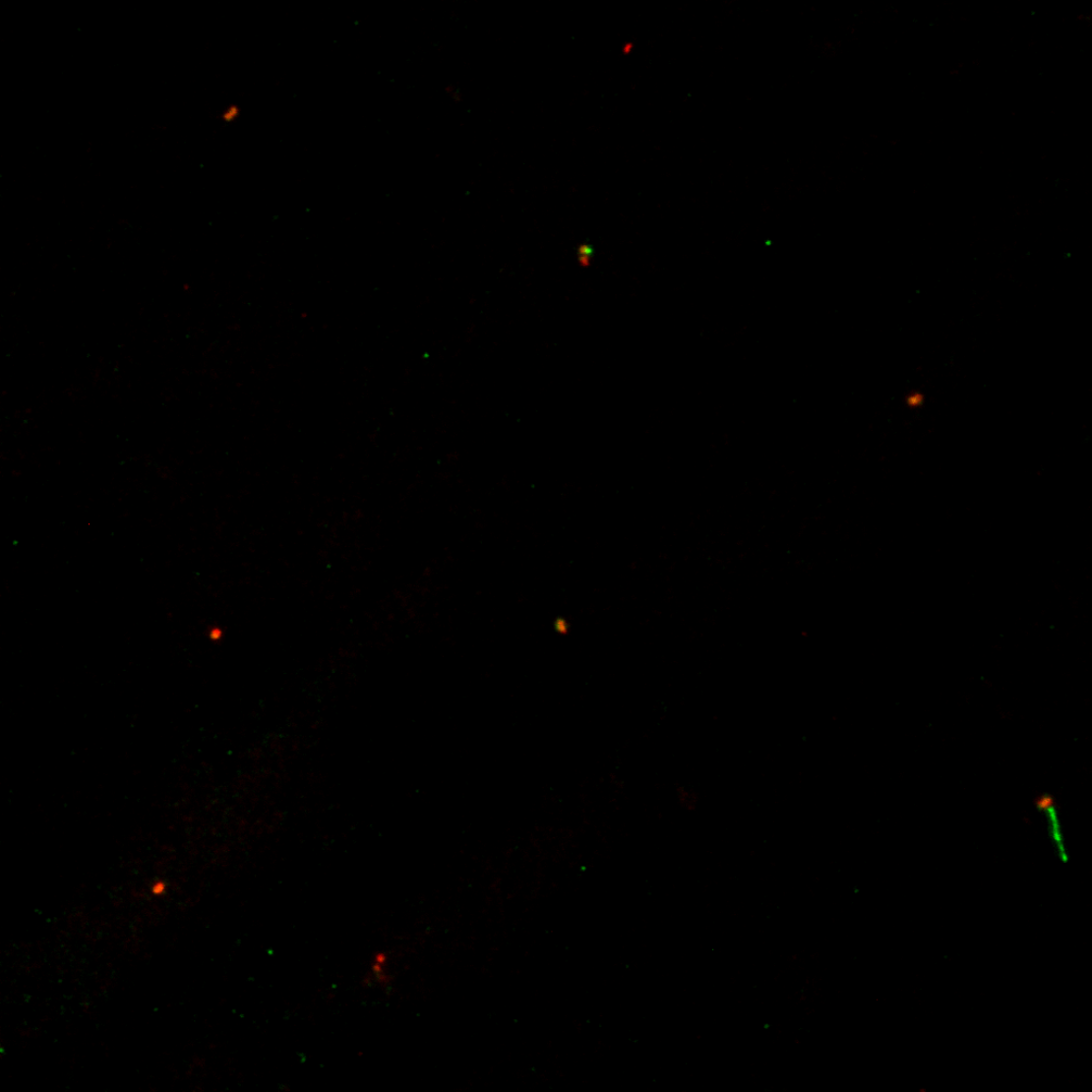

Supplement: Supplementary file 4 — Source data Fig. 3 [file 44319_2025_597_MOESM4_ESM.zip › Figure 3/3C/r-tu+Arl13b/siNC/SS3h.bmp]

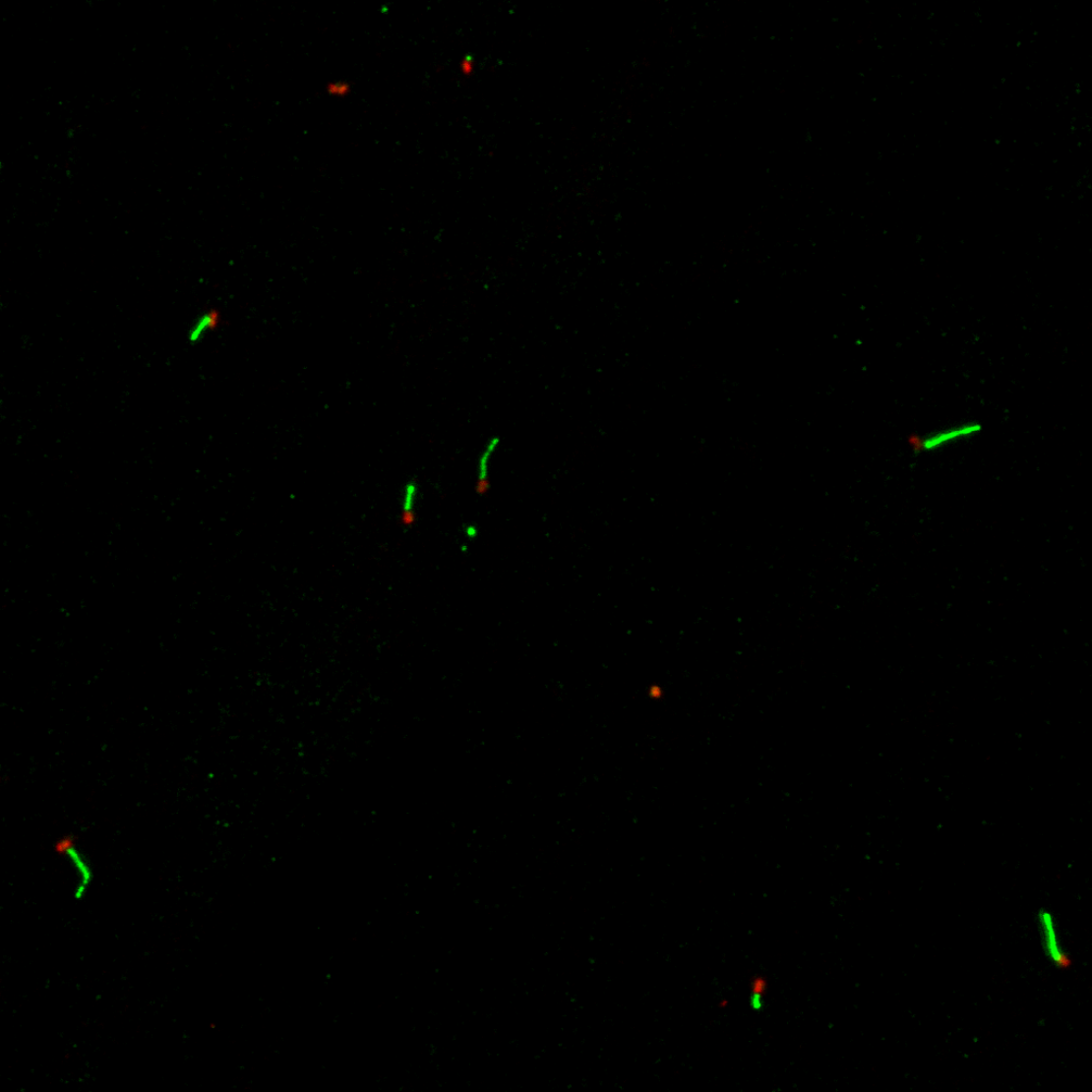

Supplement: Supplementary file 4 — Source data Fig. 3 [file 44319_2025_597_MOESM4_ESM.zip › Figure 3/3C/r-tu+Arl13b/siNC/SS6h.bmp]

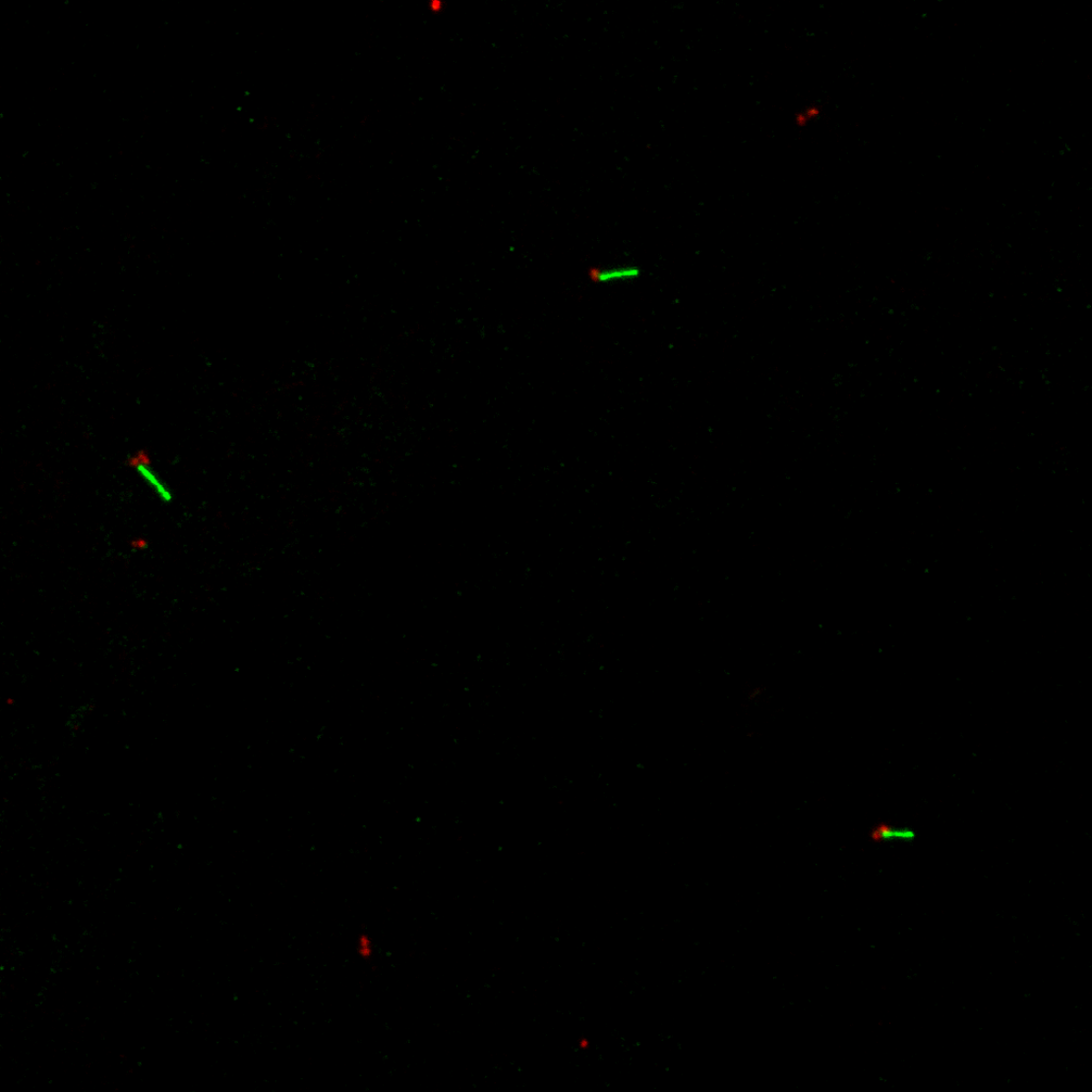

Supplement: Supplementary file 4 — Source data Fig. 3 [file 44319_2025_597_MOESM4_ESM.zip › Figure 3/3C/r-tu+Arl13b/siNC/SS9h.bmp]

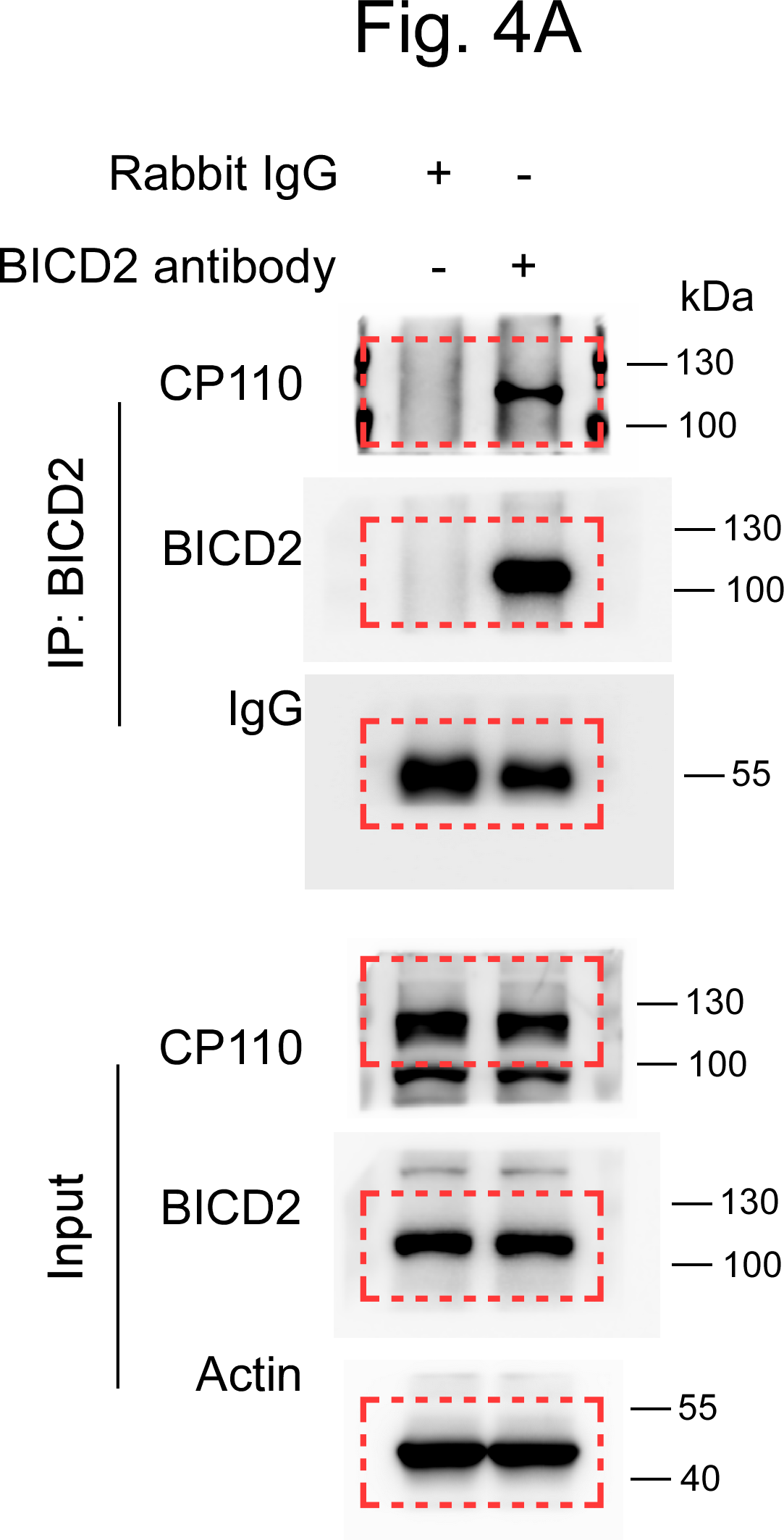

Supplement: Supplementary file 5 — Source data Fig. 4 [file 44319_2025_597_MOESM5_ESM.zip › Figure 4/4A.tif]

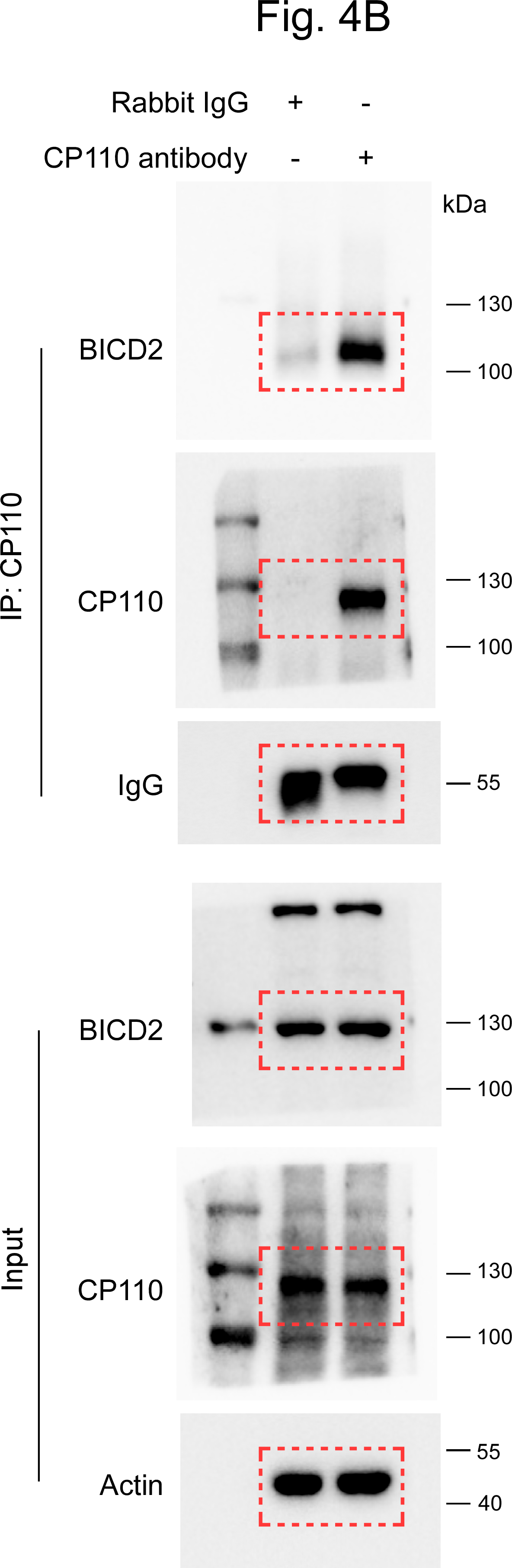

Supplement: Supplementary file 5 — Source data Fig. 4 [file 44319_2025_597_MOESM5_ESM.zip › Figure 4/4B.tif]

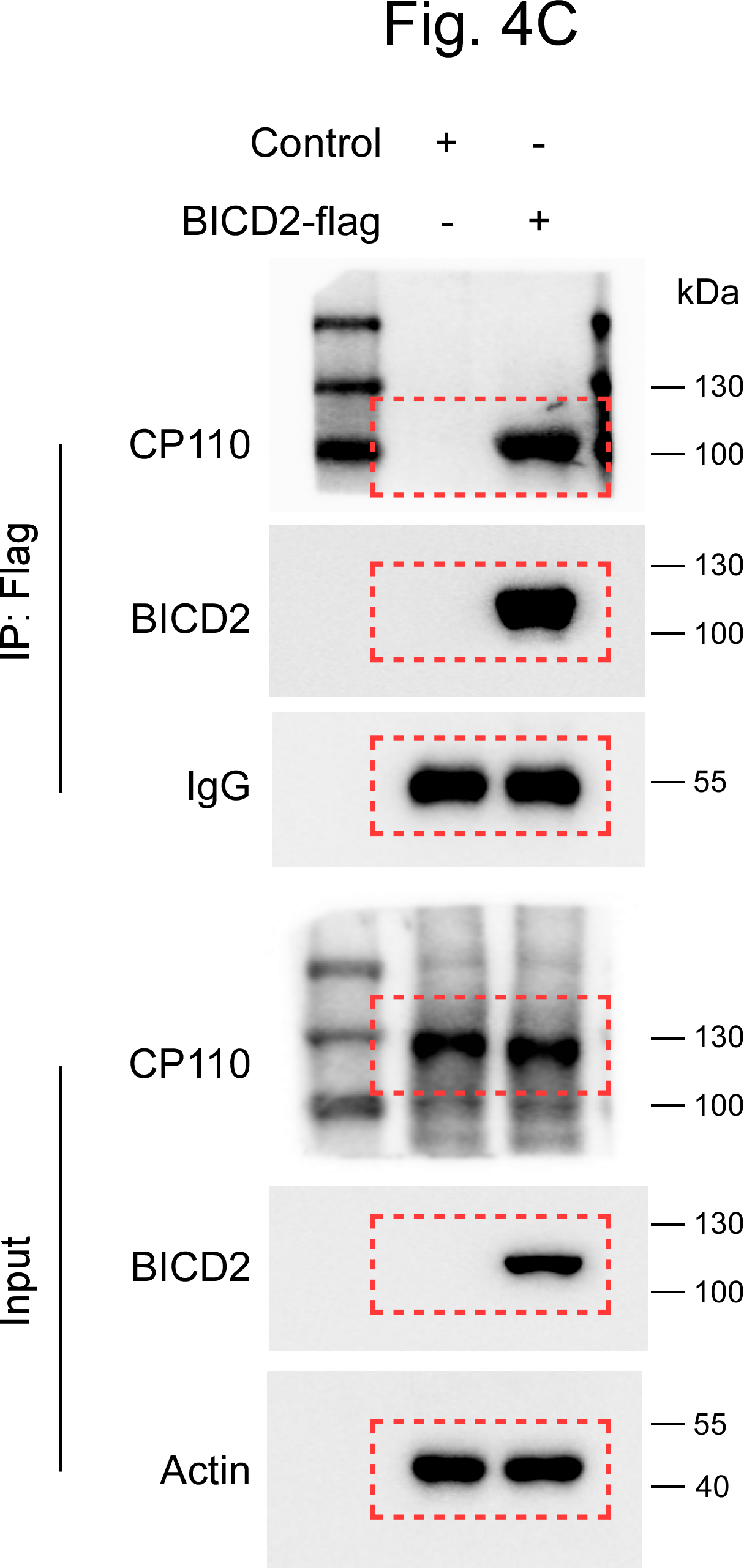

Supplement: Supplementary file 5 — Source data Fig. 4 [file 44319_2025_597_MOESM5_ESM.zip › Figure 4/4C.tif]

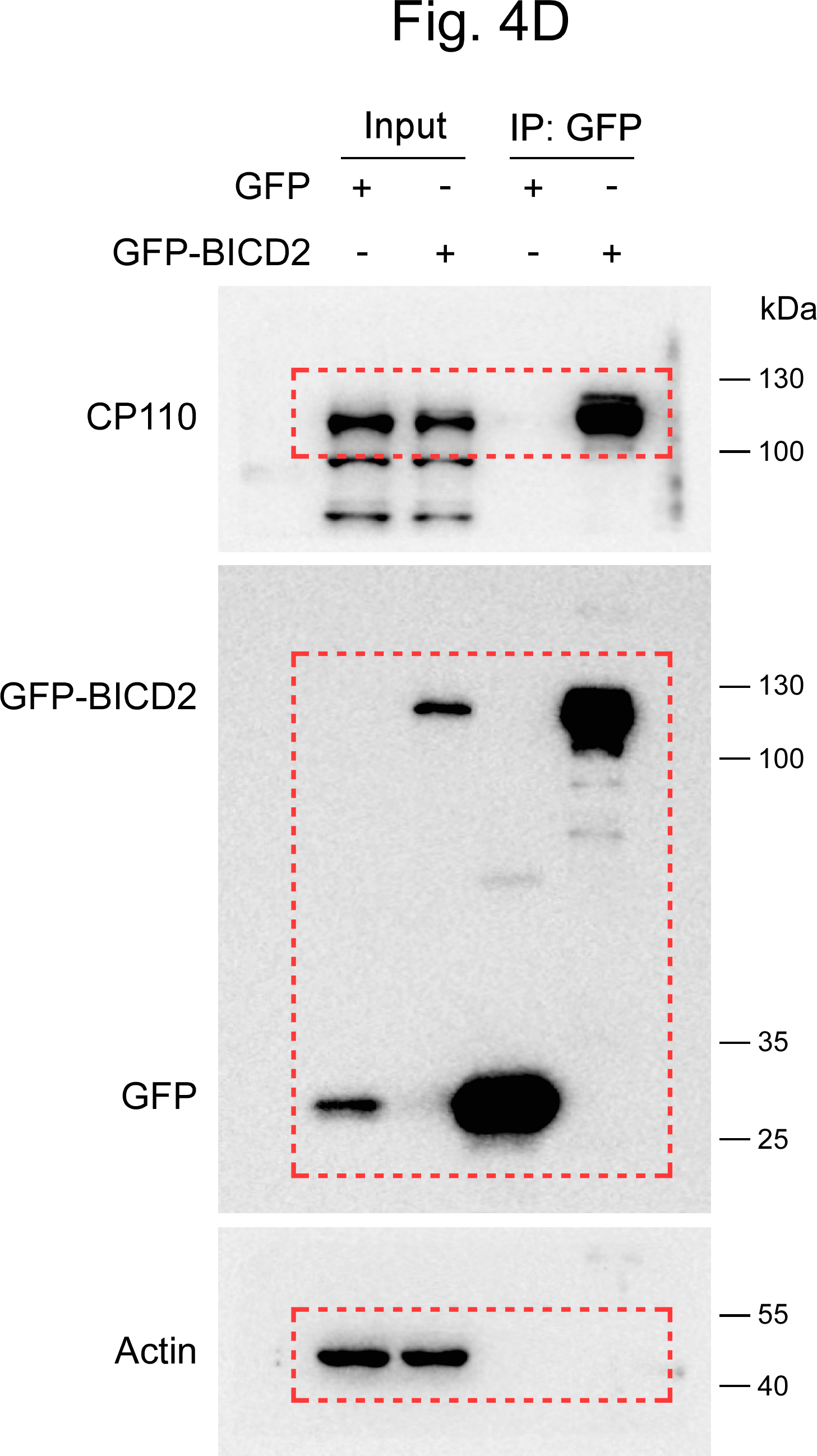

Supplement: Supplementary file 5 — Source data Fig. 4 [file 44319_2025_597_MOESM5_ESM.zip › Figure 4/4D.tif]

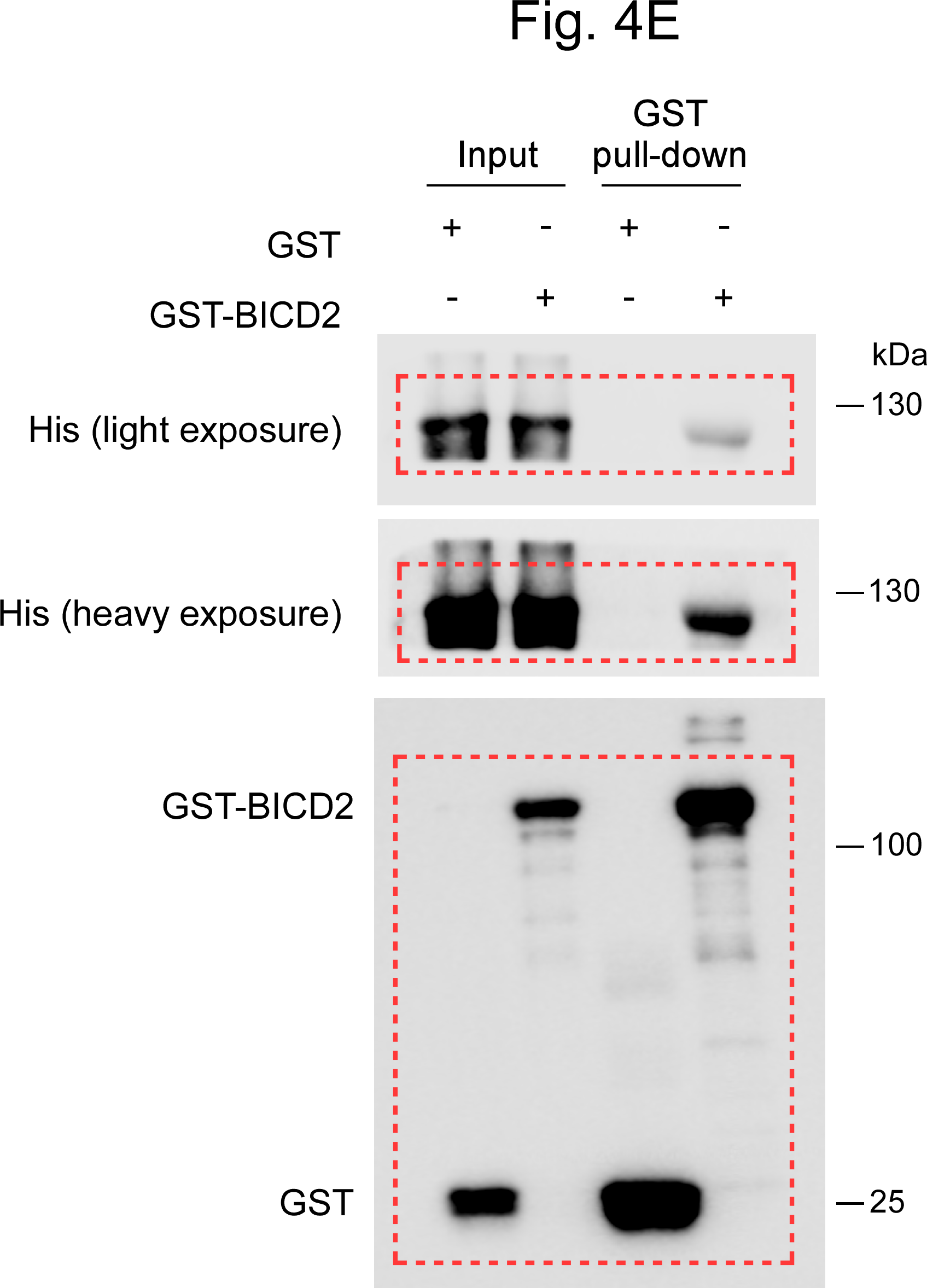

Supplement: Supplementary file 5 — Source data Fig. 4 [file 44319_2025_597_MOESM5_ESM.zip › Figure 4/4E.tif]

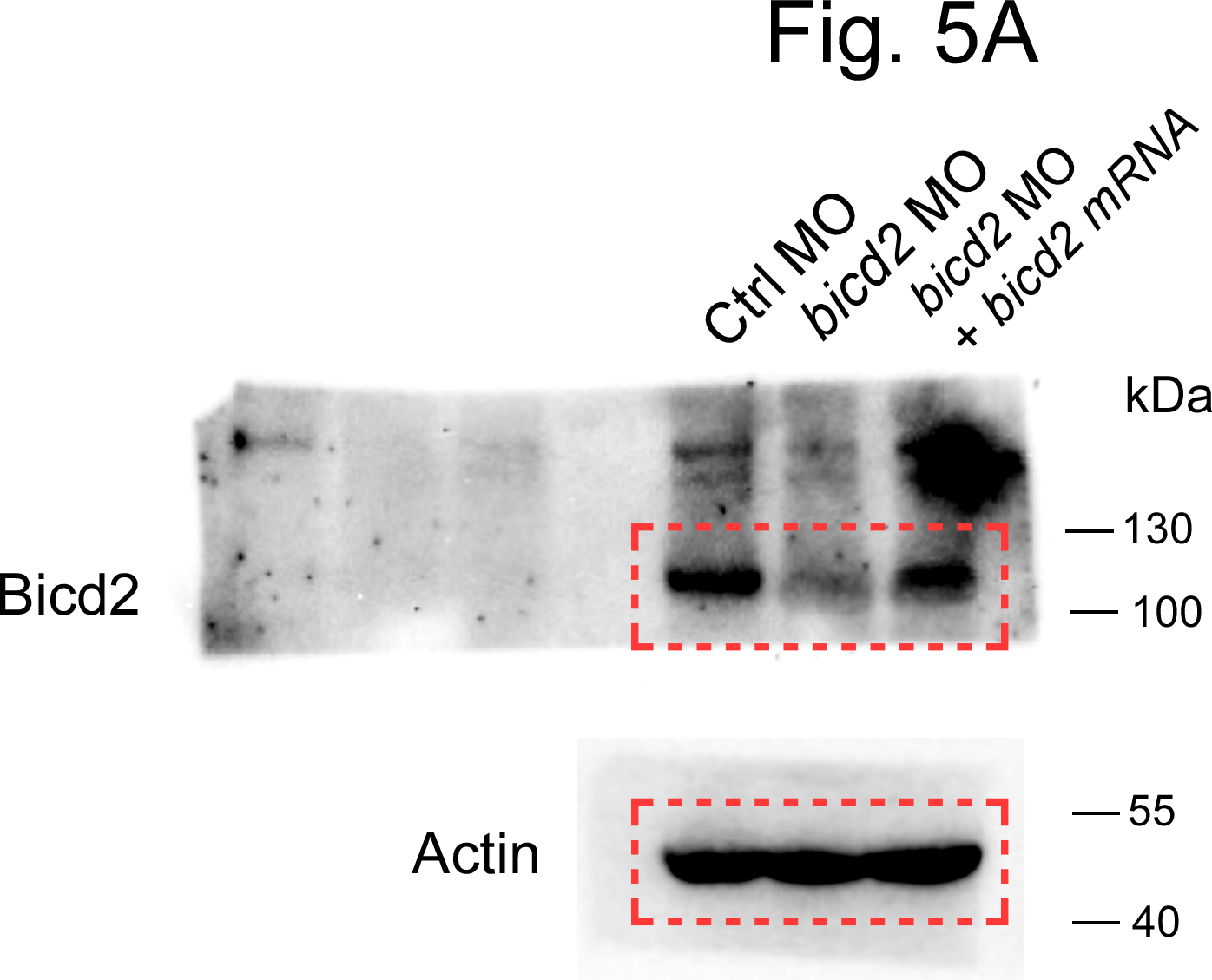

Supplement: Supplementary file 6 — Source data Fig. 5 [file 44319_2025_597_MOESM6_ESM.zip › Figure 5/5A.tif]

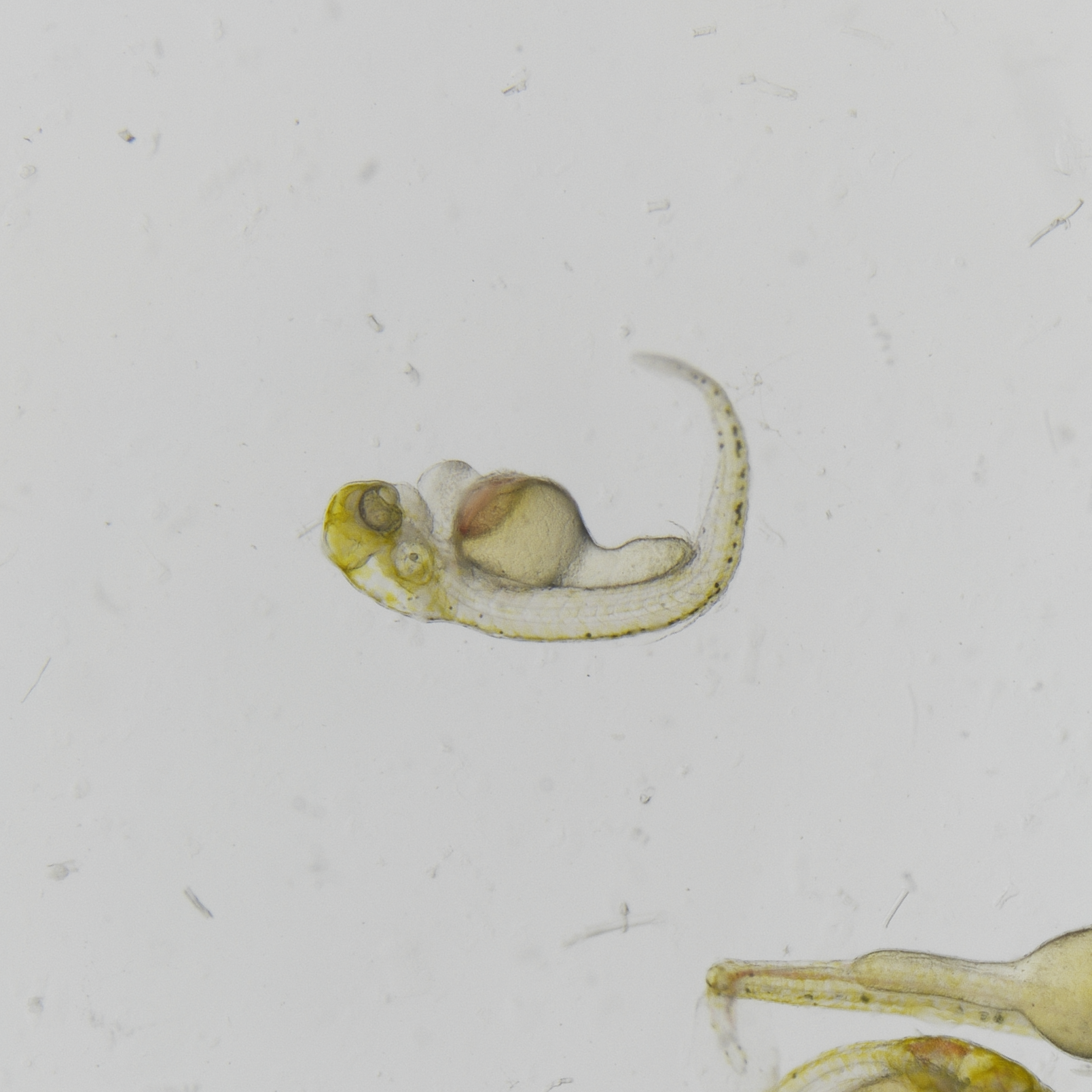

Supplement: Supplementary file 6 — Source data Fig. 5 [file 44319_2025_597_MOESM6_ESM.zip › Figure 5/5B/bicd2 mo (1).tif]

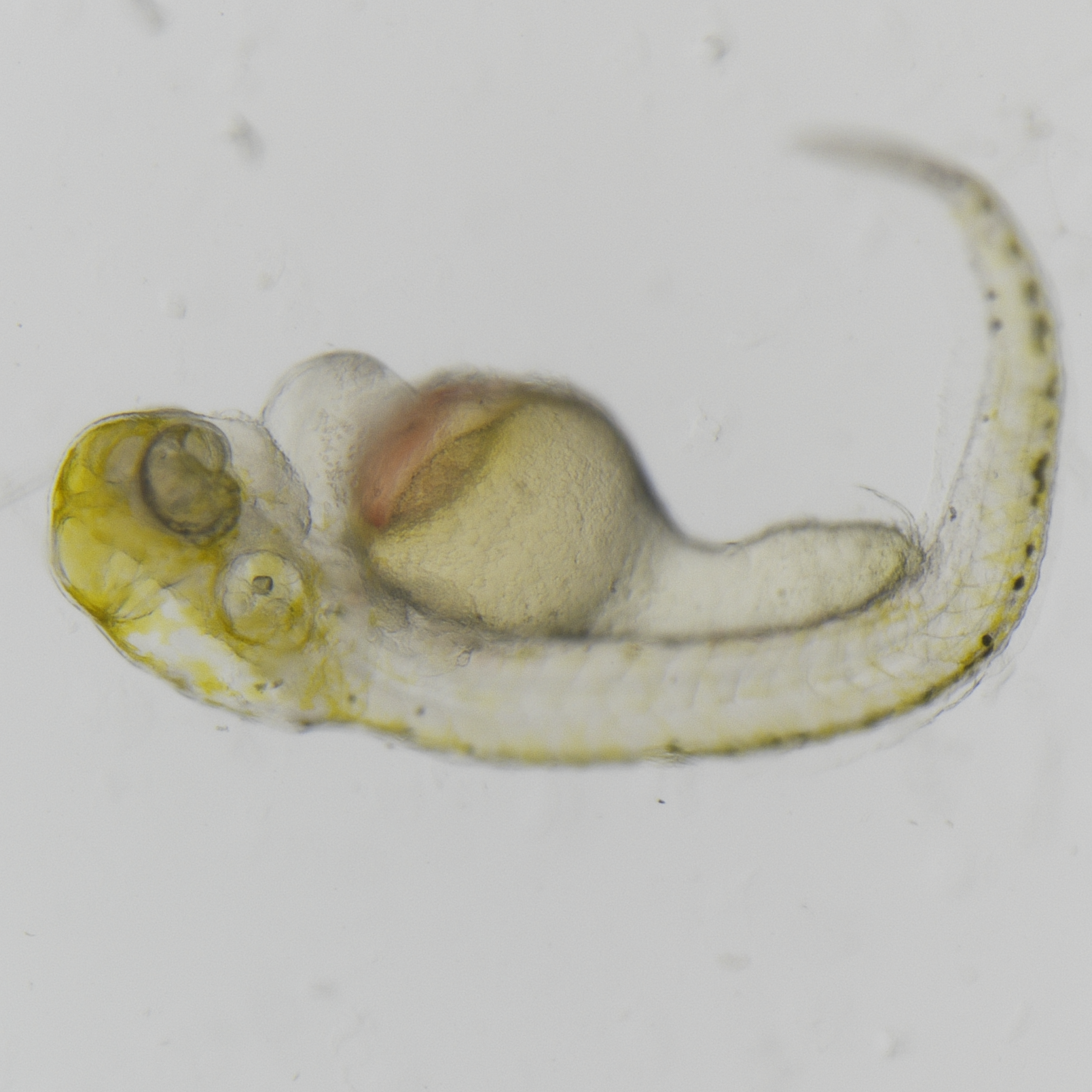

Supplement: Supplementary file 6 — Source data Fig. 5 [file 44319_2025_597_MOESM6_ESM.zip › Figure 5/5B/bicd2 mo (2).tif]

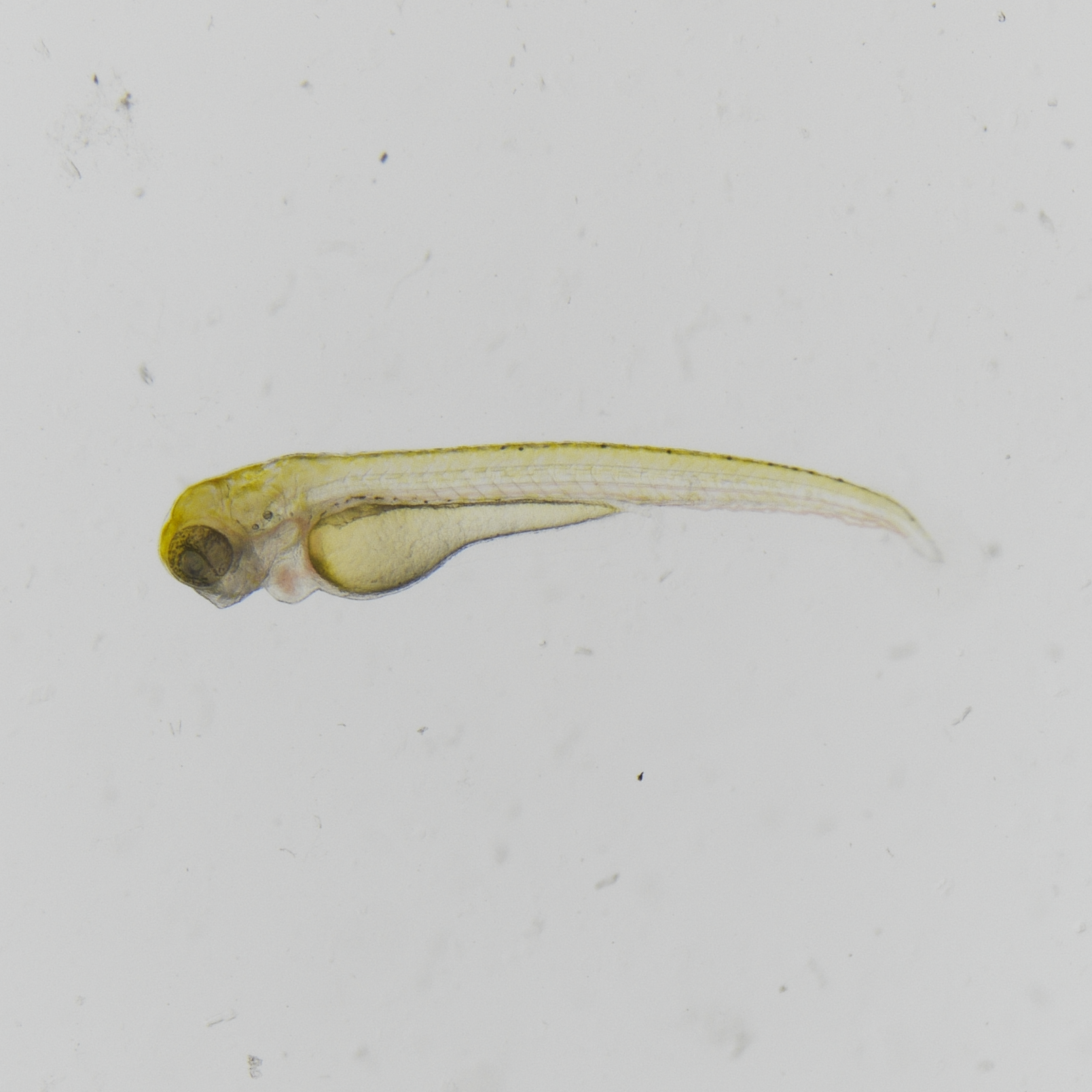

Supplement: Supplementary file 6 — Source data Fig. 5 [file 44319_2025_597_MOESM6_ESM.zip › Figure 5/5B/bicd2 mo+bicd2 mRNA (1).tif]

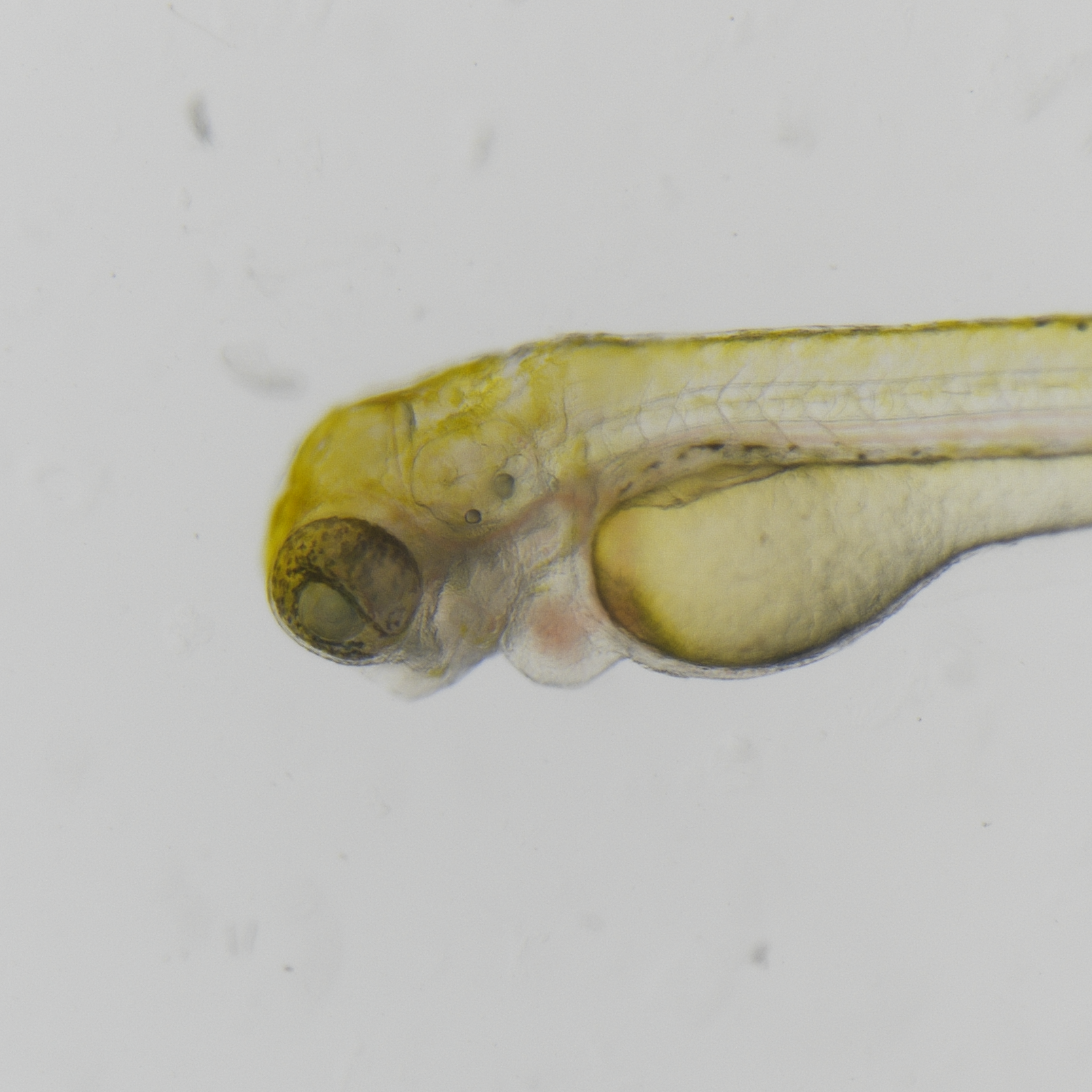

Supplement: Supplementary file 6 — Source data Fig. 5 [file 44319_2025_597_MOESM6_ESM.zip › Figure 5/5B/bicd2 mo+bicd2 mRNA (2).tif]

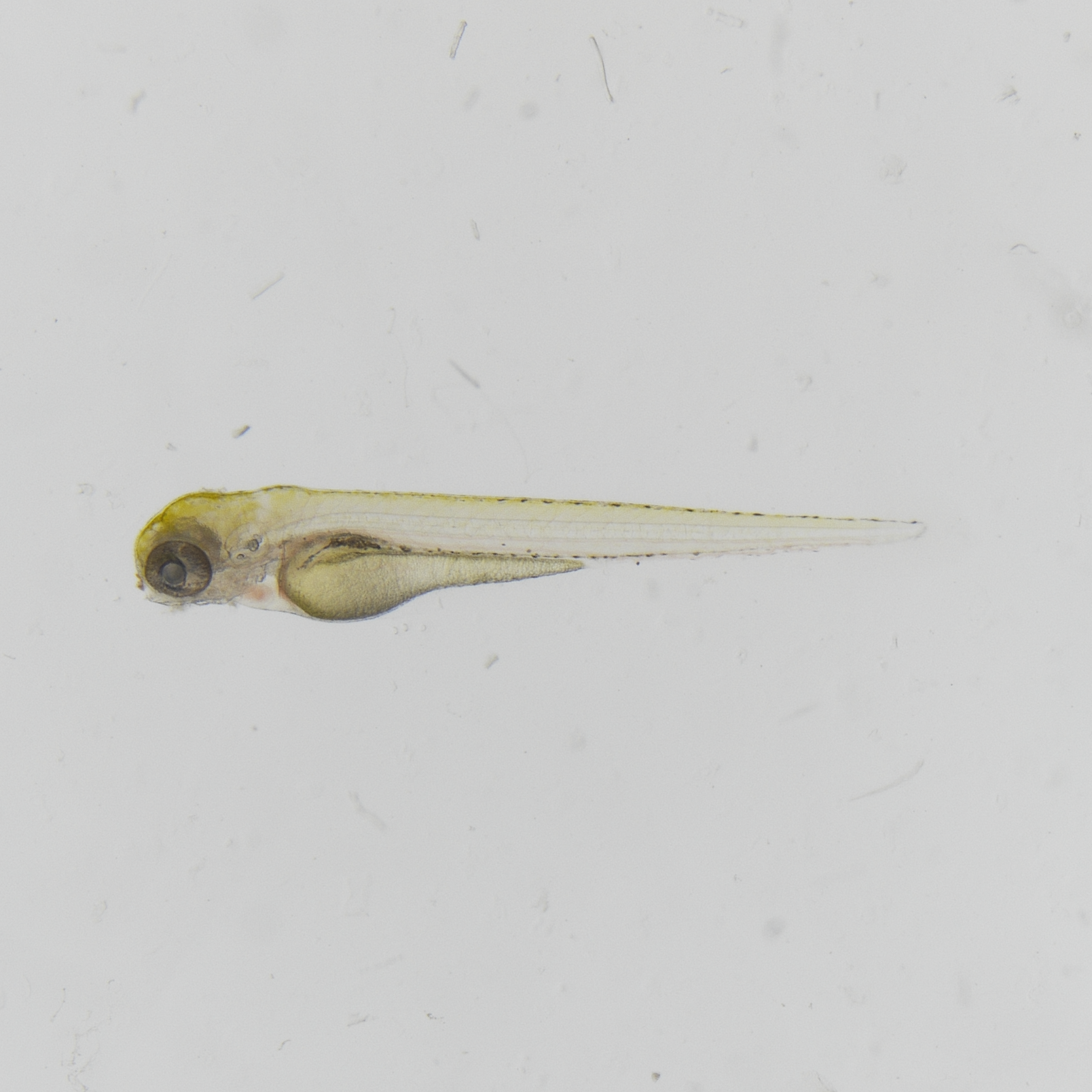

Supplement: Supplementary file 6 — Source data Fig. 5 [file 44319_2025_597_MOESM6_ESM.zip › Figure 5/5B/Ctrl MO (1).tif]

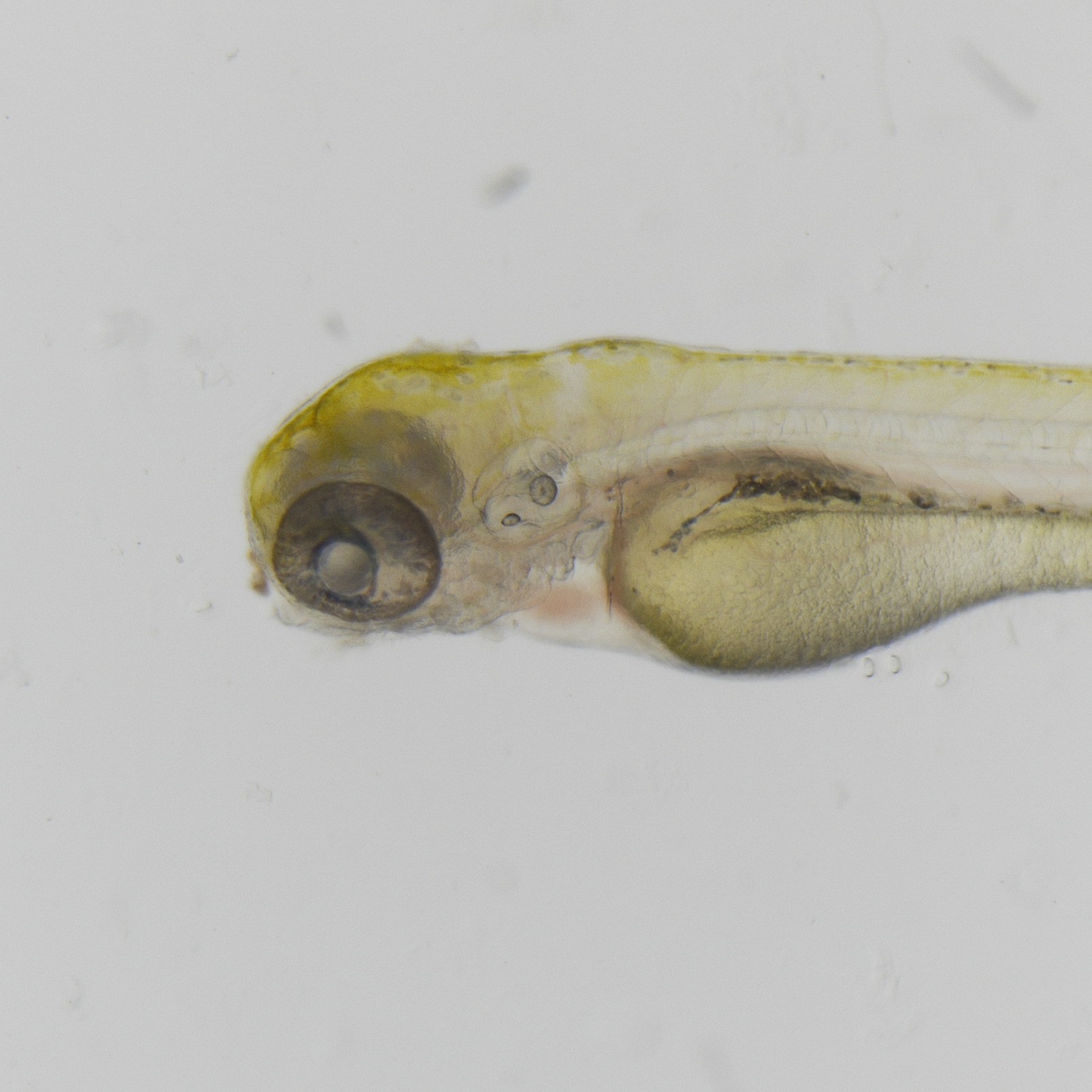

Supplement: Supplementary file 6 — Source data Fig. 5 [file 44319_2025_597_MOESM6_ESM.zip › Figure 5/5B/Ctrl MO (2).tif]

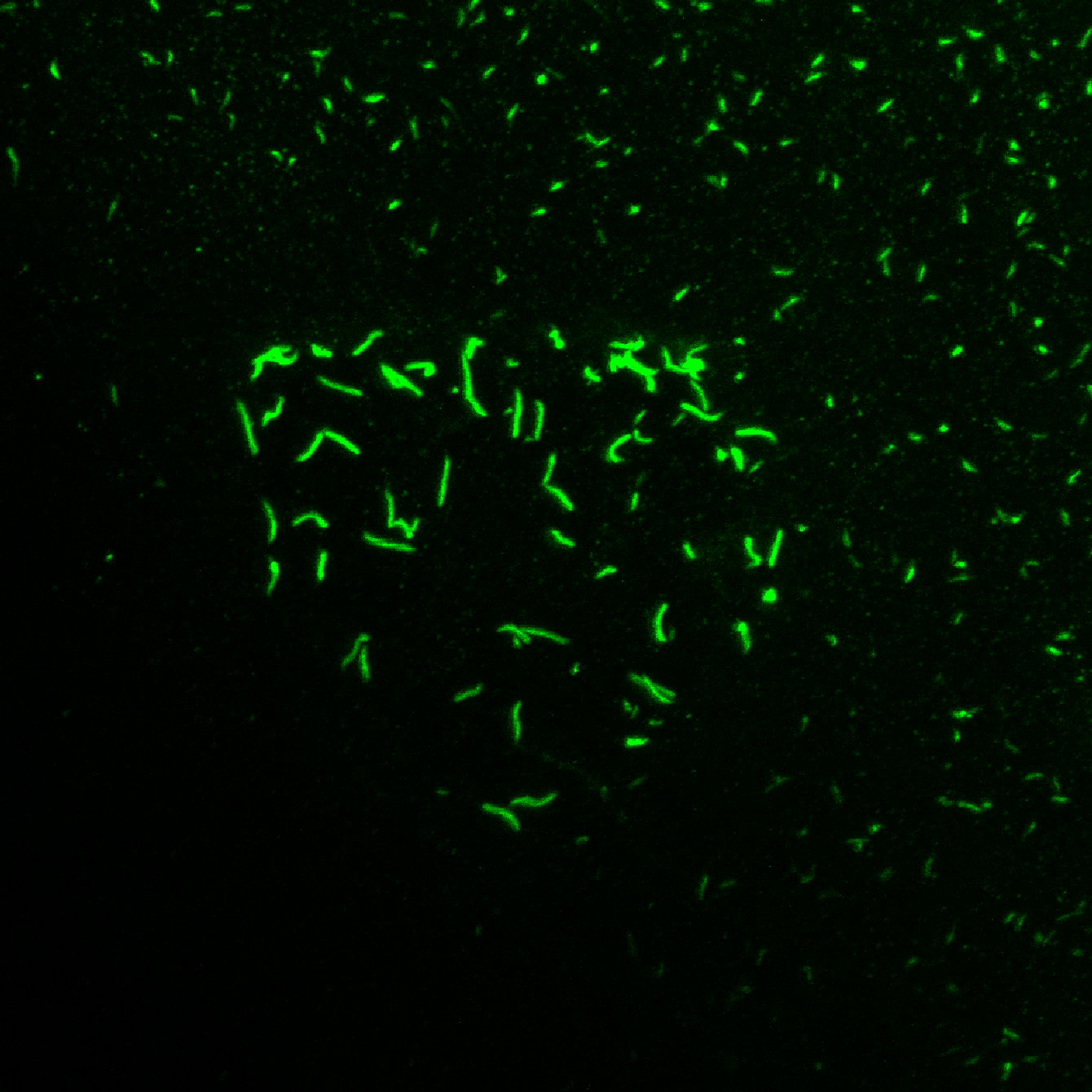

Supplement: Supplementary file 6 — Source data Fig. 5 [file 44319_2025_597_MOESM6_ESM.zip › Figure 5/5D/bicd2 MO+bicd2 mRNA.tif]

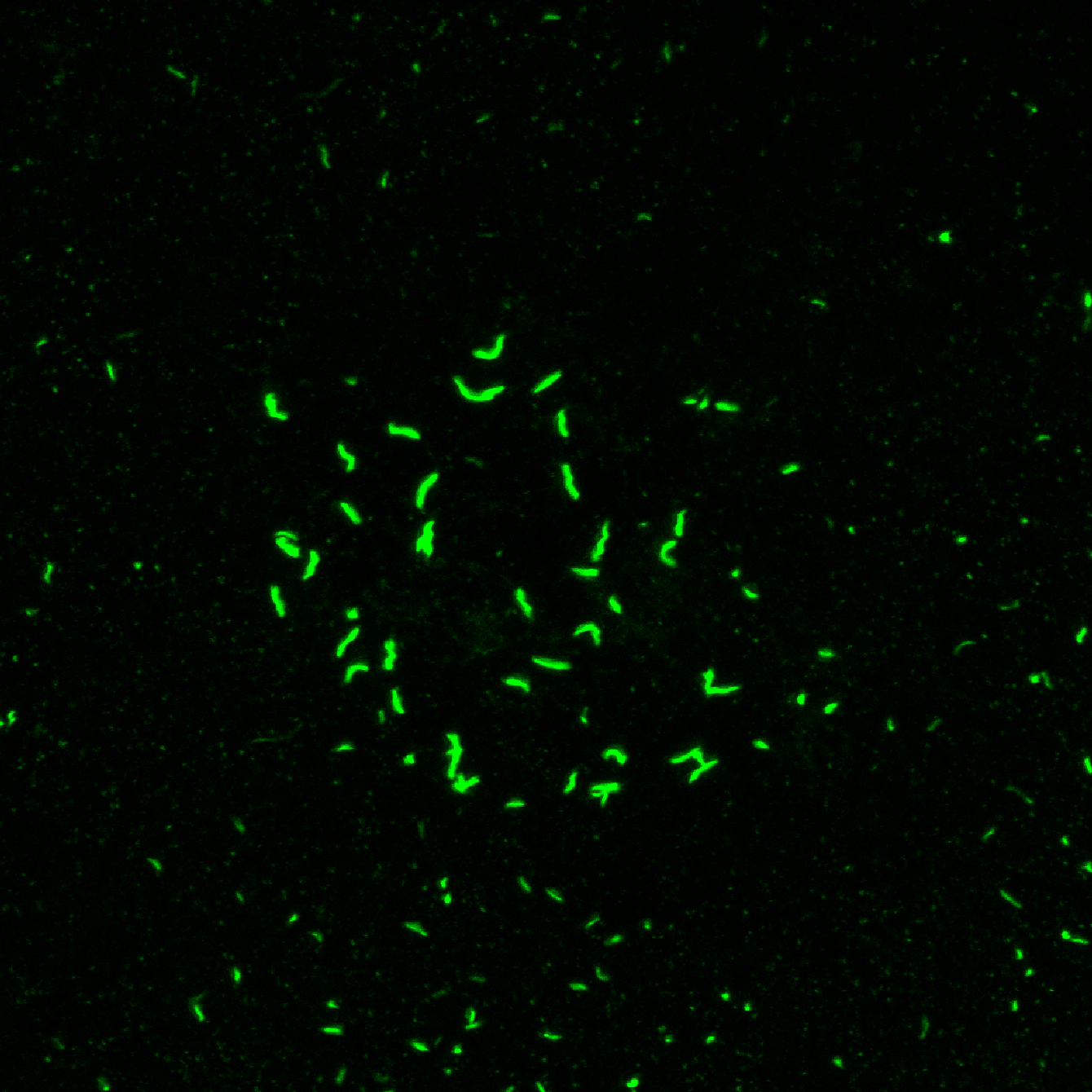

Supplement: Supplementary file 6 — Source data Fig. 5 [file 44319_2025_597_MOESM6_ESM.zip › Figure 5/5D/bicd2 MO.tif]

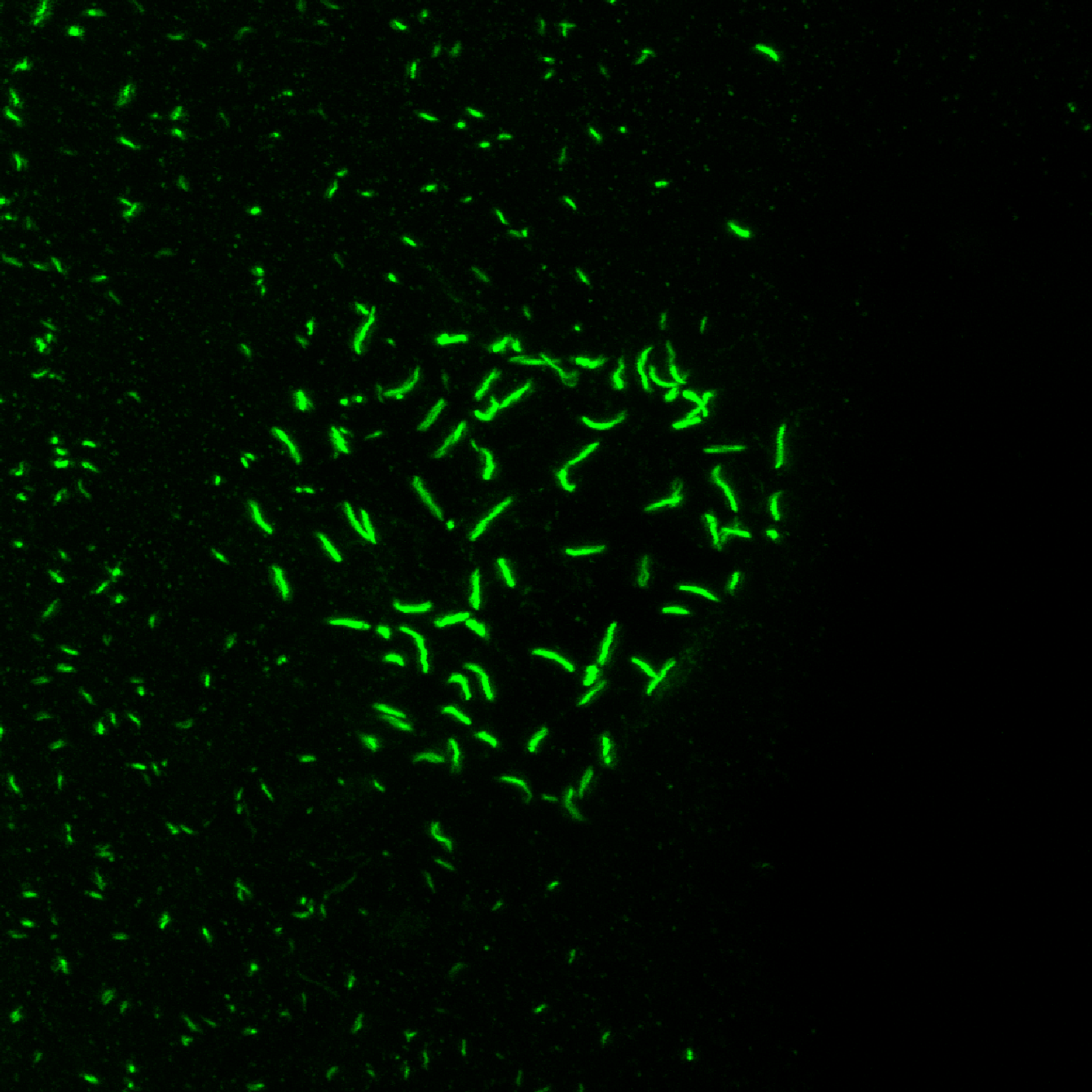

Supplement: Supplementary file 6 — Source data Fig. 5 [file 44319_2025_597_MOESM6_ESM.zip › Figure 5/5D/Ctrl MO.tif]

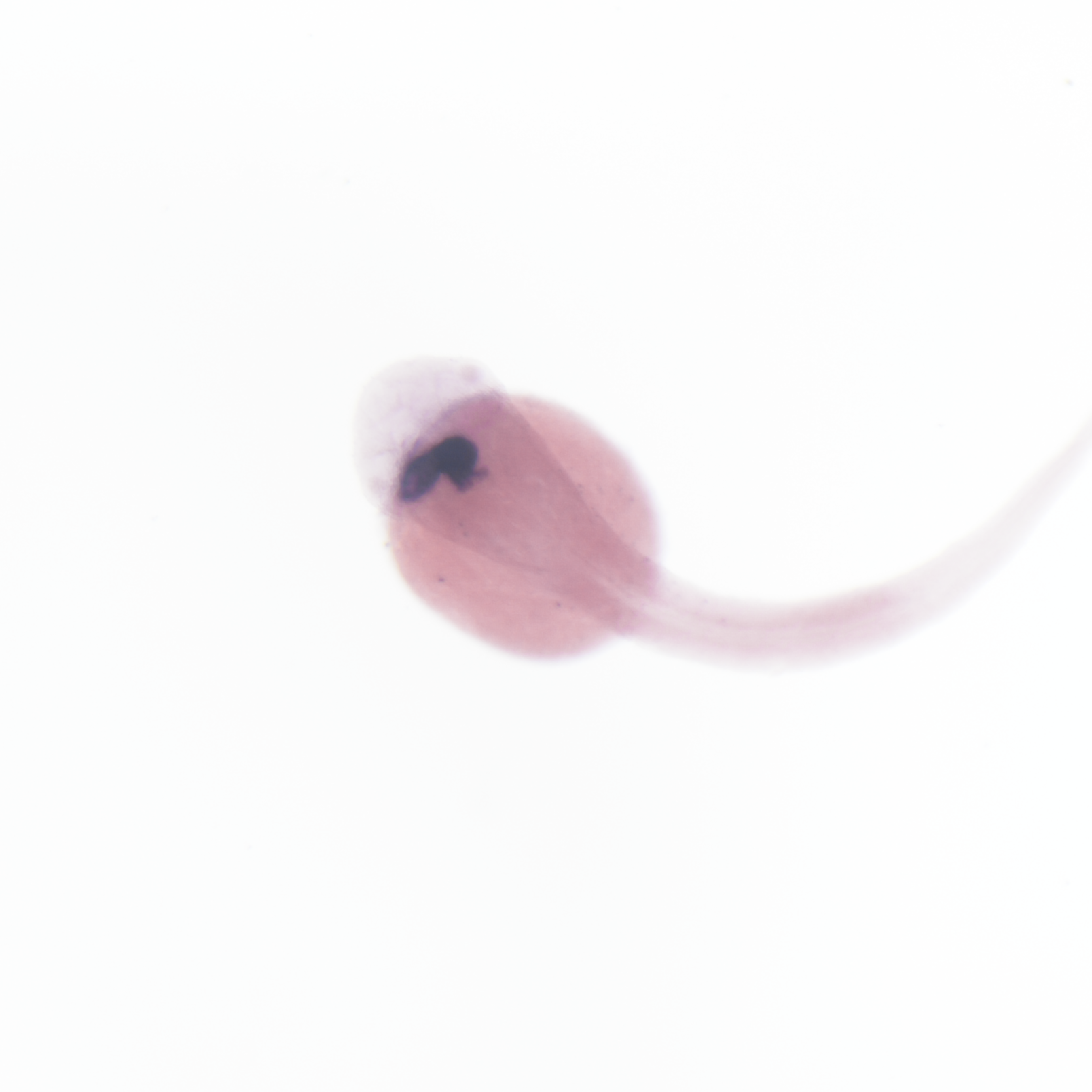

Supplement: Supplementary file 6 — Source data Fig. 5 [file 44319_2025_597_MOESM6_ESM.zip › Figure 5/5G/Left.tif]

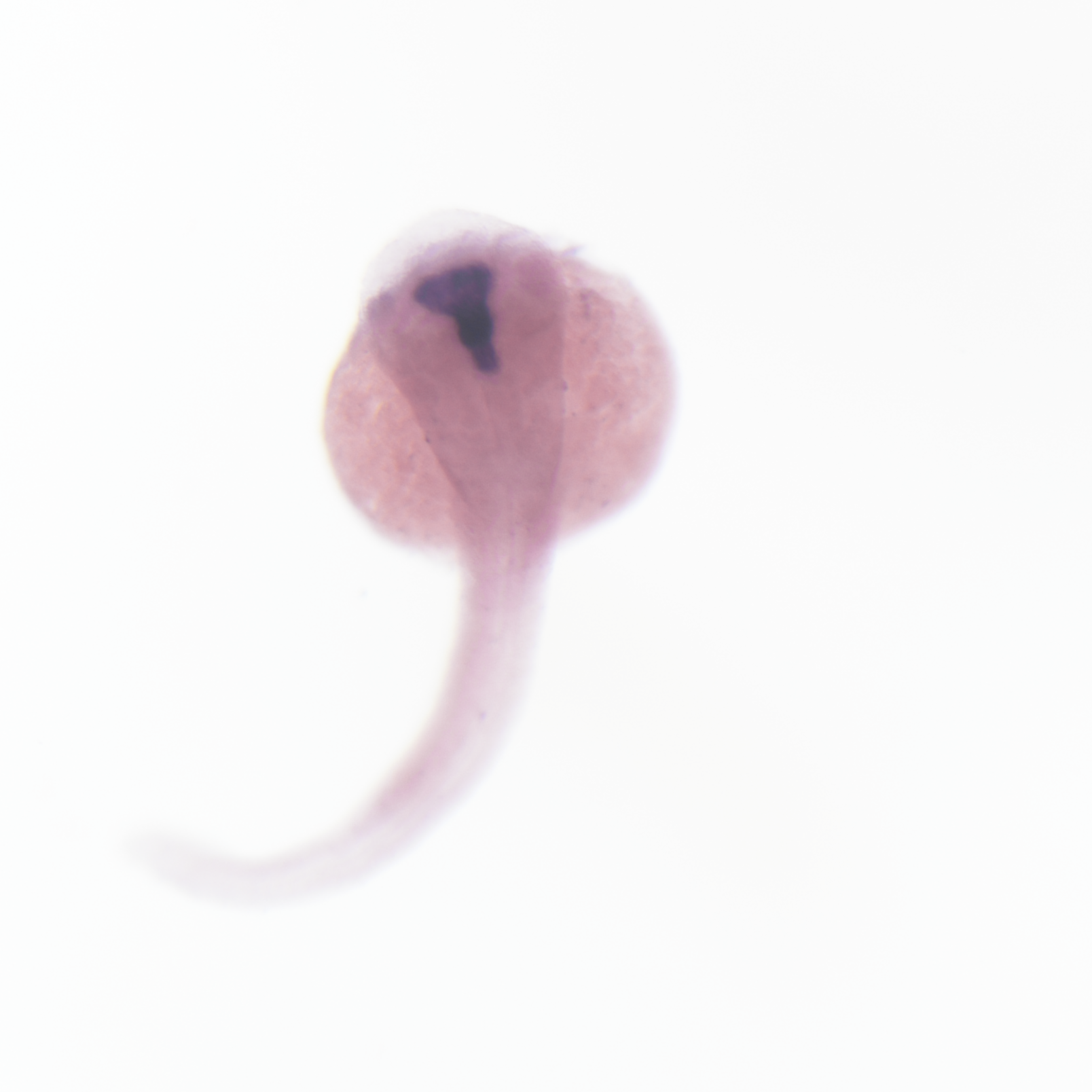

Supplement: Supplementary file 6 — Source data Fig. 5 [file 44319_2025_597_MOESM6_ESM.zip › Figure 5/5G/Middle.tif]

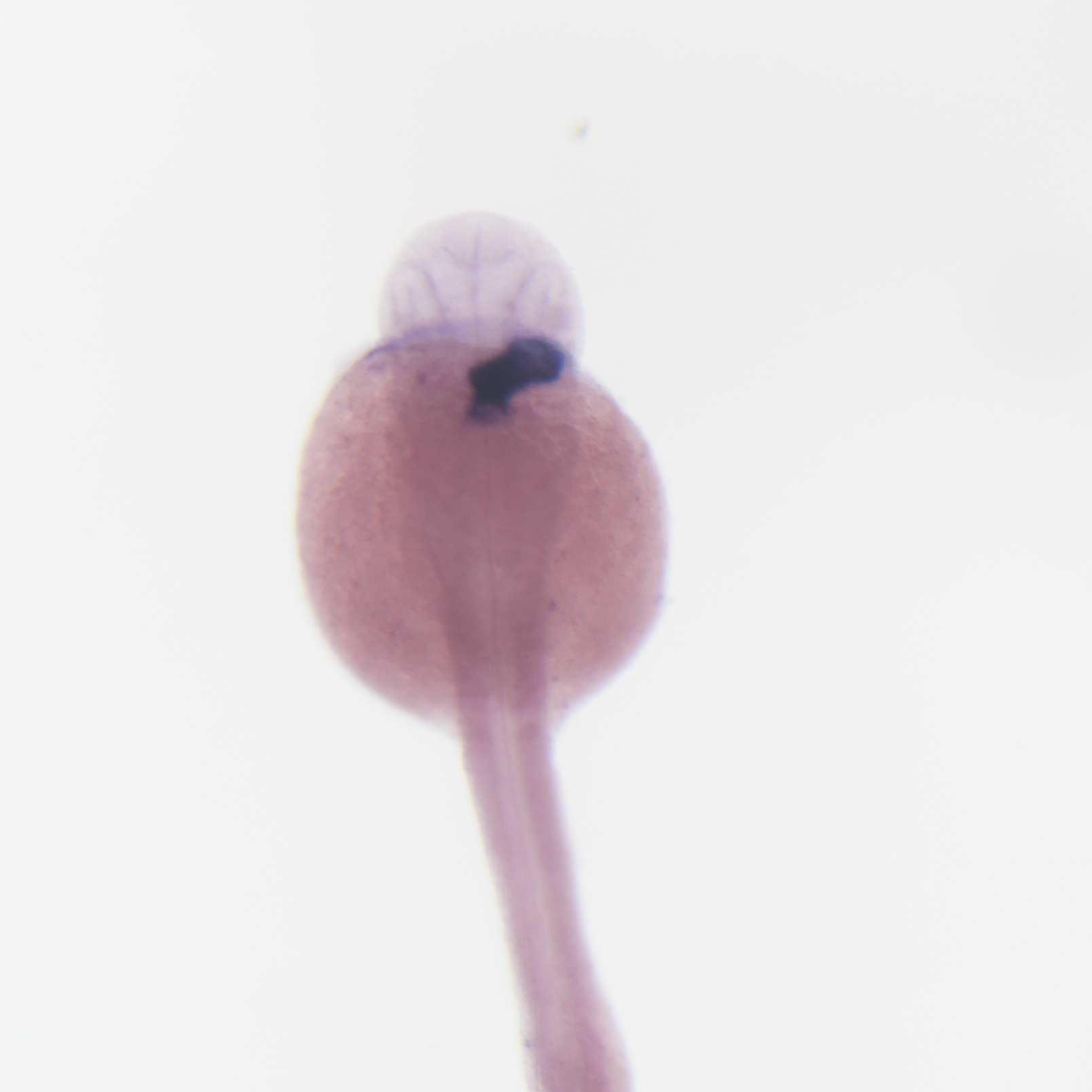

Supplement: Supplementary file 6 — Source data Fig. 5 [file 44319_2025_597_MOESM6_ESM.zip › Figure 5/5G/Right.tif]

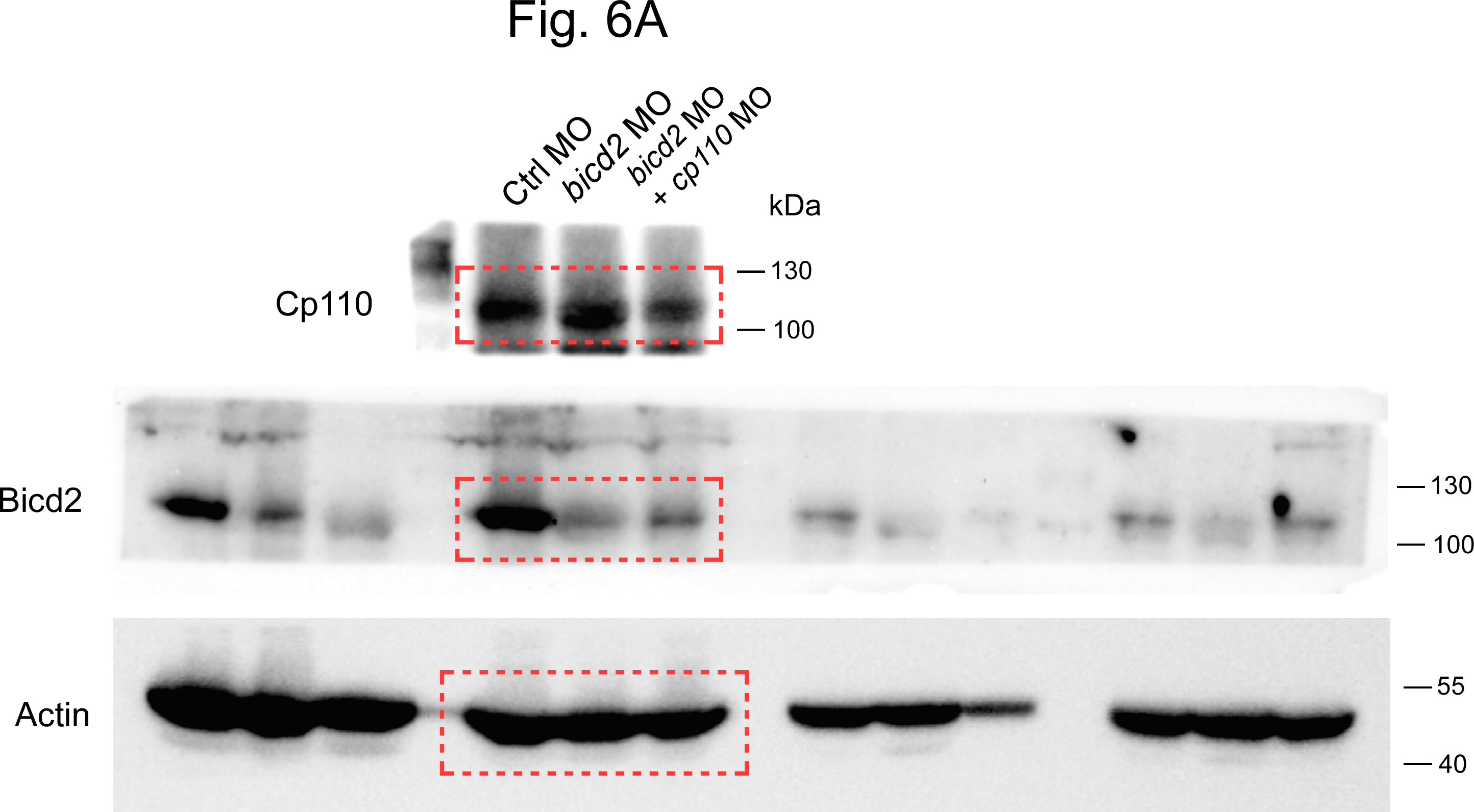

Supplement: Supplementary file 7 — Source data Fig. 6 [file 44319_2025_597_MOESM7_ESM.zip › Figure 6/6A.tif]

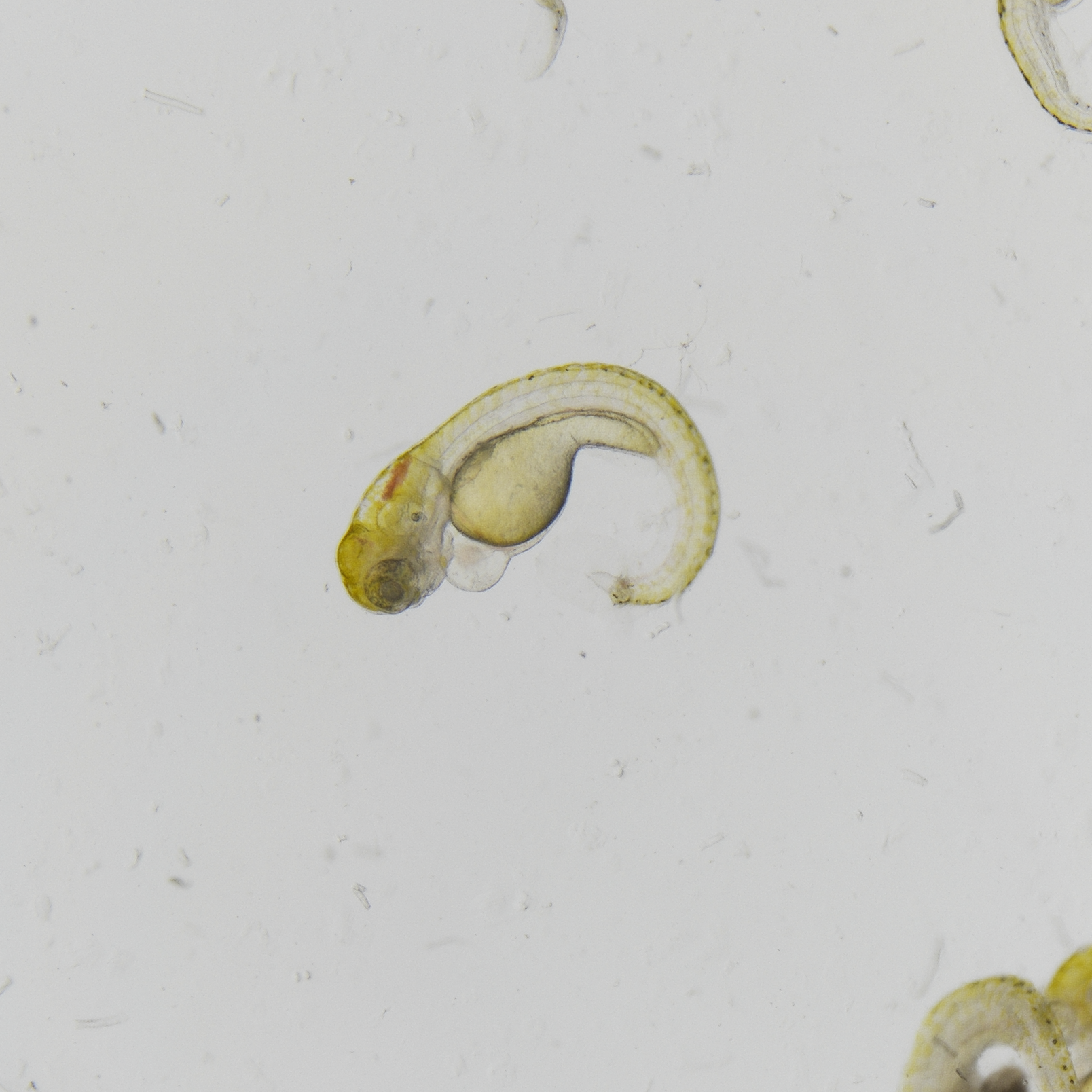

Supplement: Supplementary file 7 — Source data Fig. 6 [file 44319_2025_597_MOESM7_ESM.zip › Figure 6/6B/bicd2 MO (1).tif]

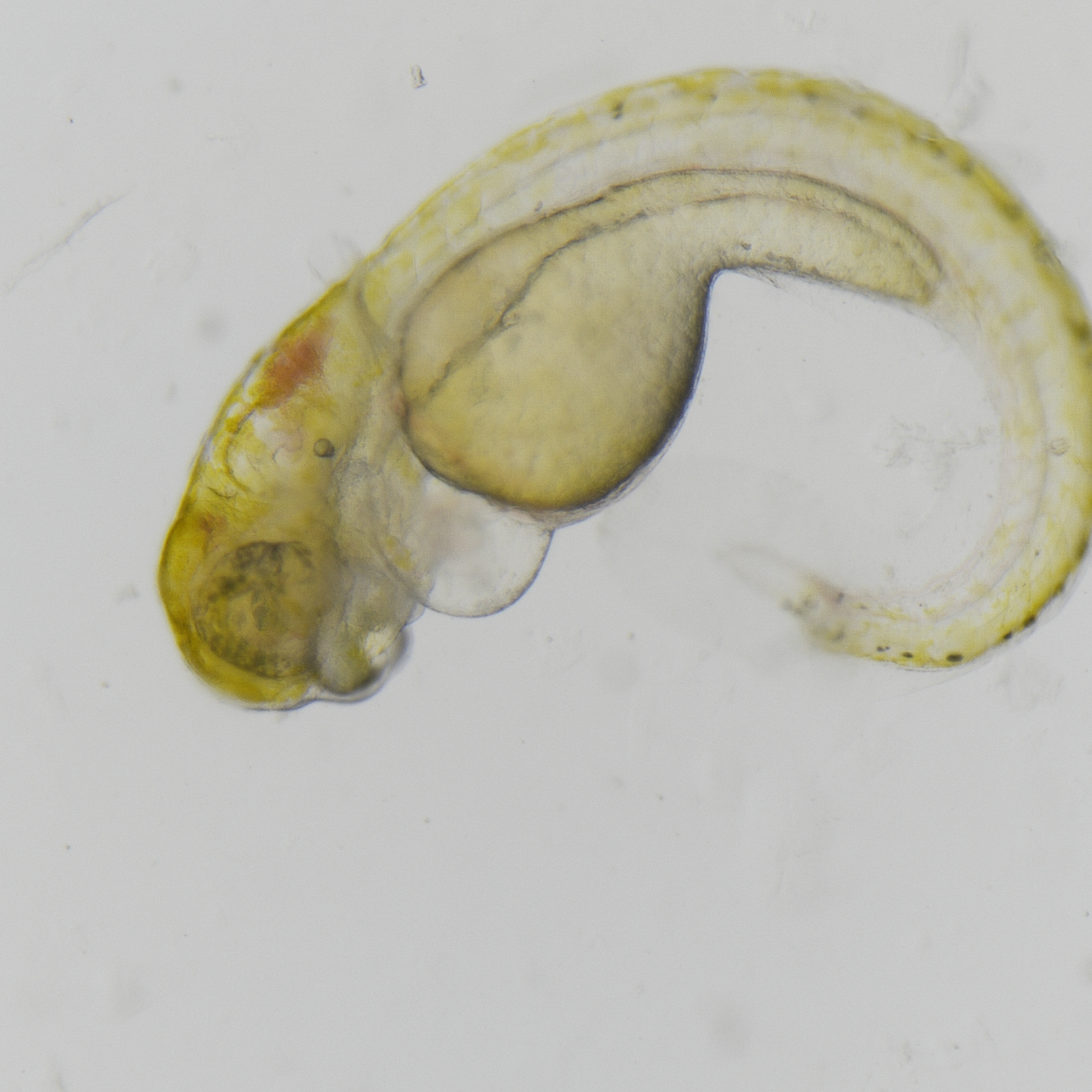

Supplement: Supplementary file 7 — Source data Fig. 6 [file 44319_2025_597_MOESM7_ESM.zip › Figure 6/6B/bicd2 MO (2).tif]

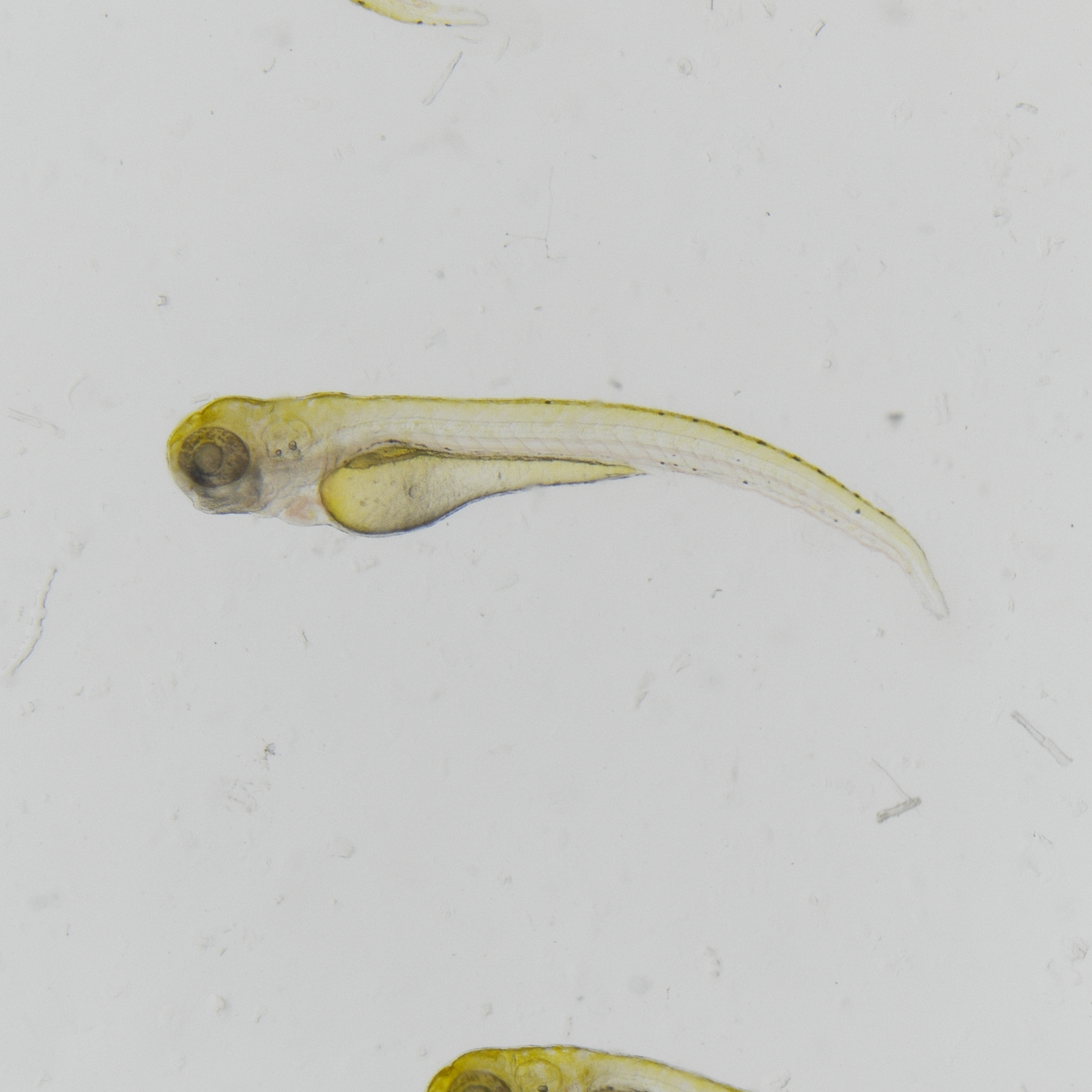

Supplement: Supplementary file 7 — Source data Fig. 6 [file 44319_2025_597_MOESM7_ESM.zip › Figure 6/6B/bicd2 MO+cp110 MO (1).tif]

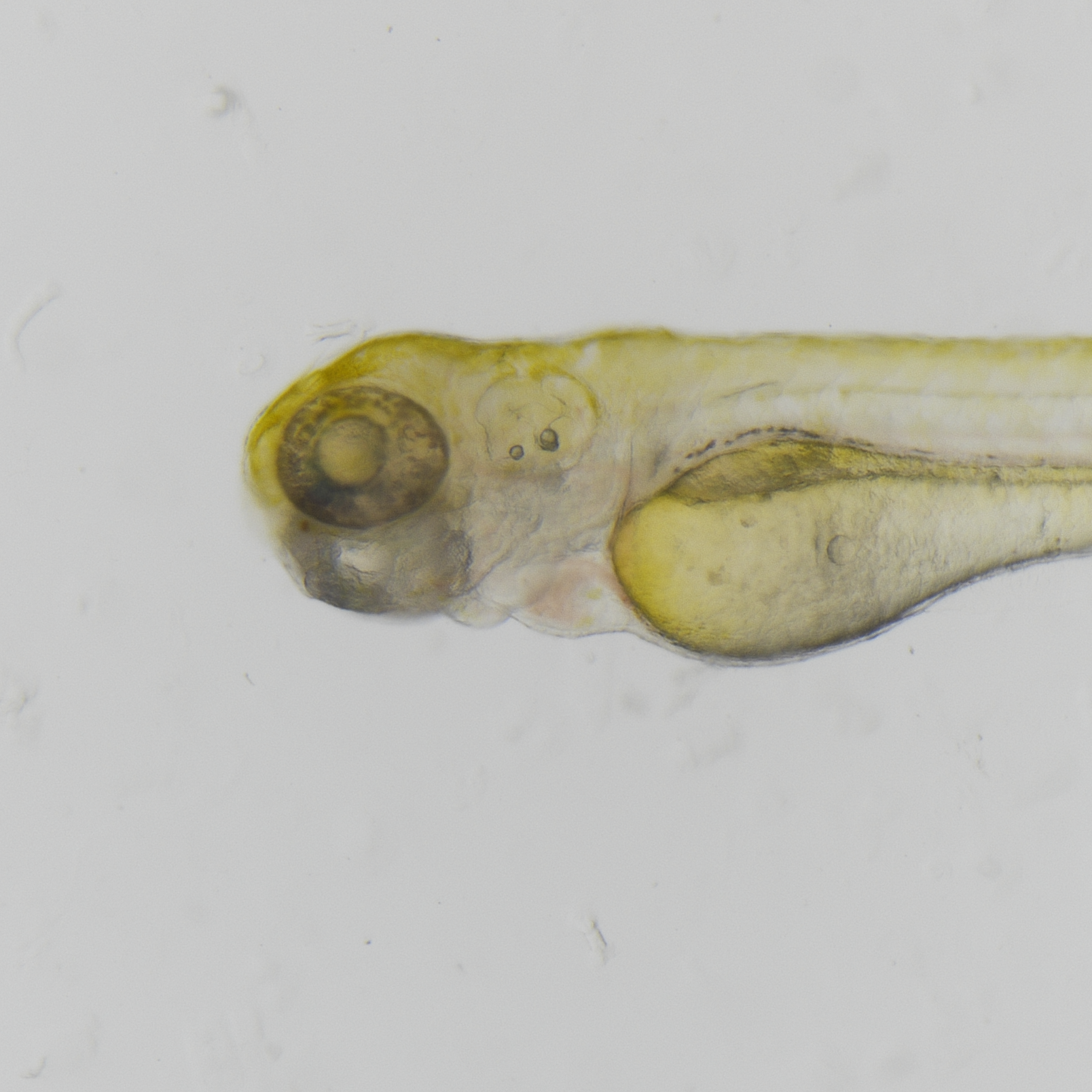

Supplement: Supplementary file 7 — Source data Fig. 6 [file 44319_2025_597_MOESM7_ESM.zip › Figure 6/6B/bicd2 MO+cp110 MO (2).tif]

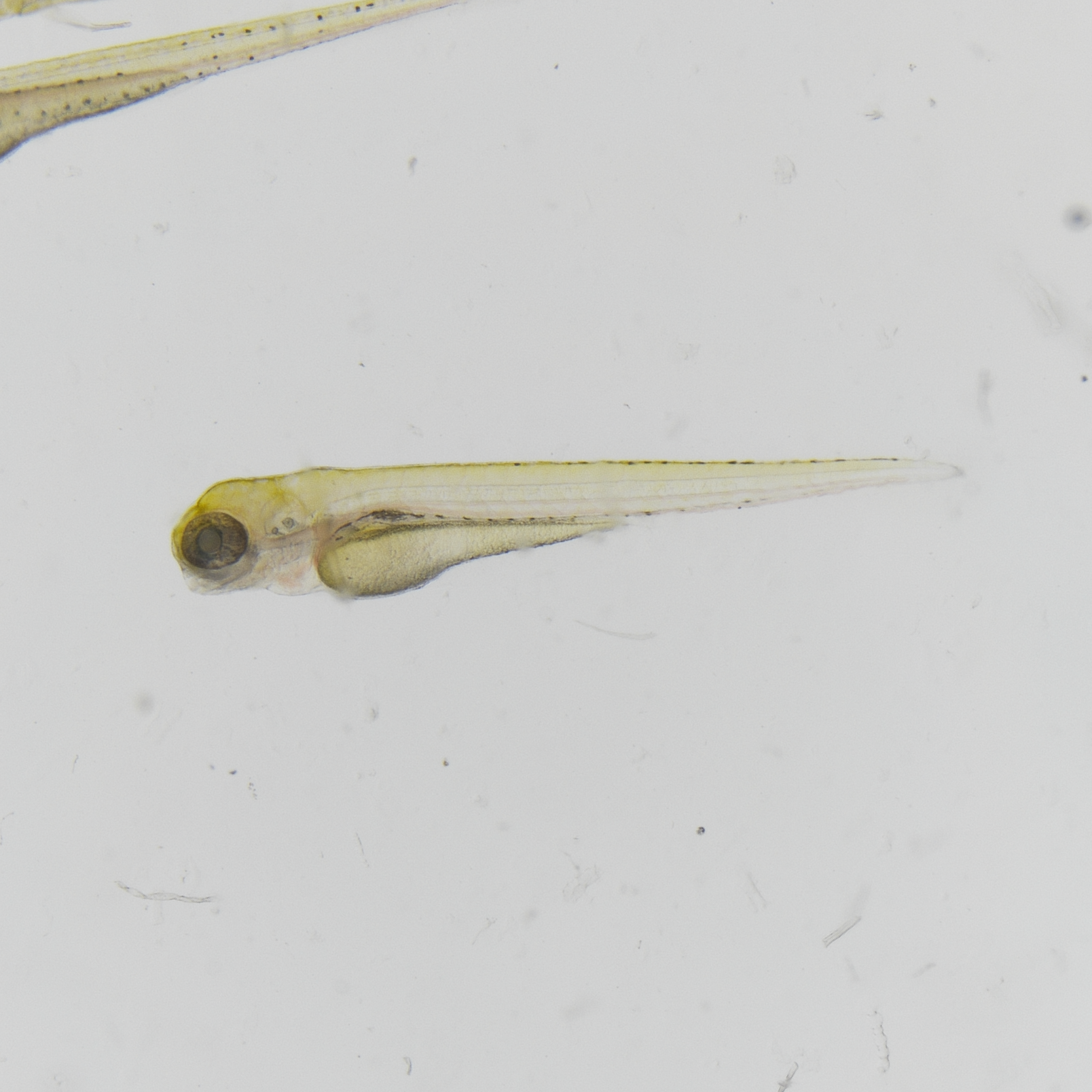

Supplement: Supplementary file 7 — Source data Fig. 6 [file 44319_2025_597_MOESM7_ESM.zip › Figure 6/6B/Ctrl MO (1).tif]

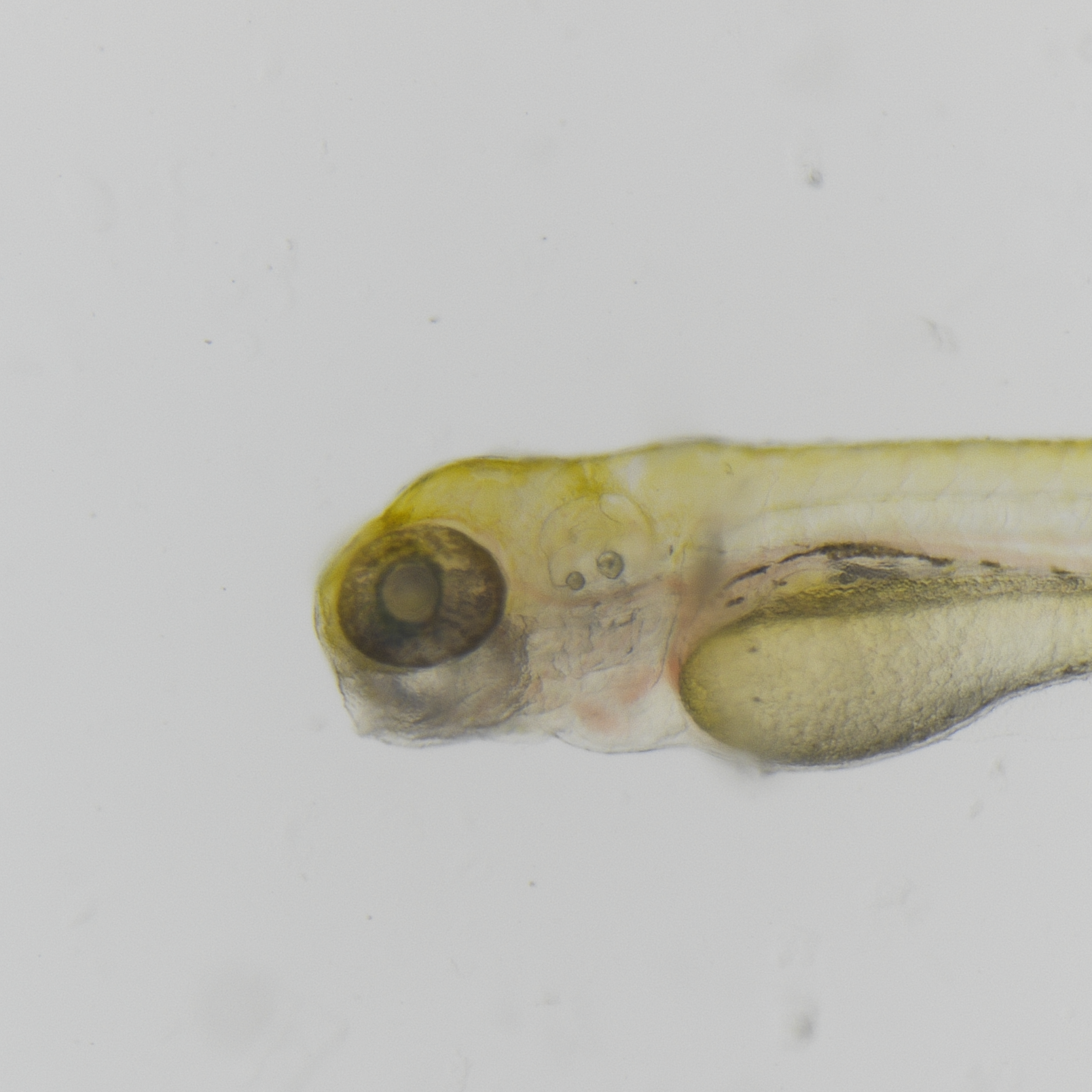

Supplement: Supplementary file 7 — Source data Fig. 6 [file 44319_2025_597_MOESM7_ESM.zip › Figure 6/6B/Ctrl MO (2).tif]

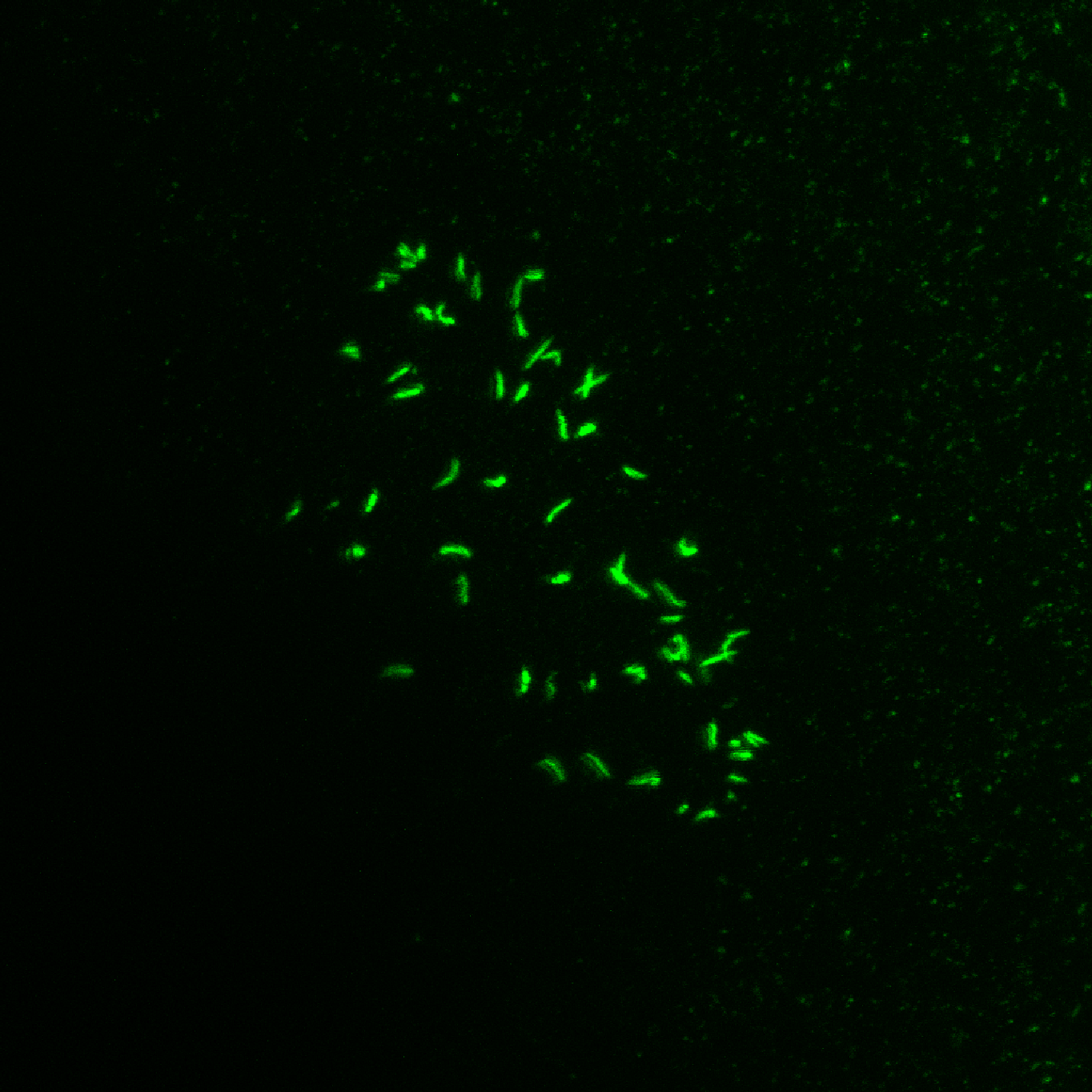

Supplement: Supplementary file 7 — Source data Fig. 6 [file 44319_2025_597_MOESM7_ESM.zip › Figure 6/6D/bicd2 MO+cp110 MO.tif]

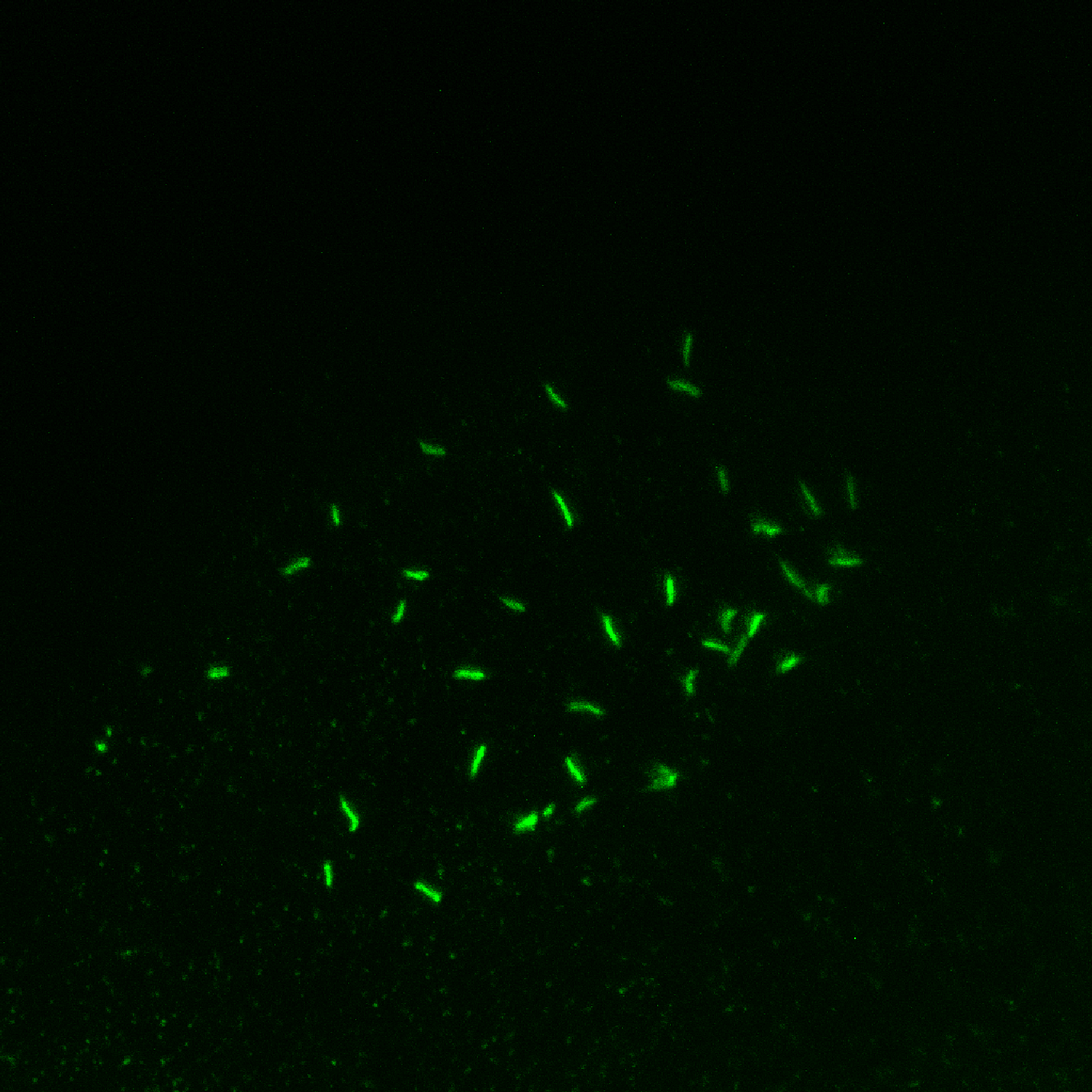

Supplement: Supplementary file 7 — Source data Fig. 6 [file 44319_2025_597_MOESM7_ESM.zip › Figure 6/6D/bicd2 MO.tif]

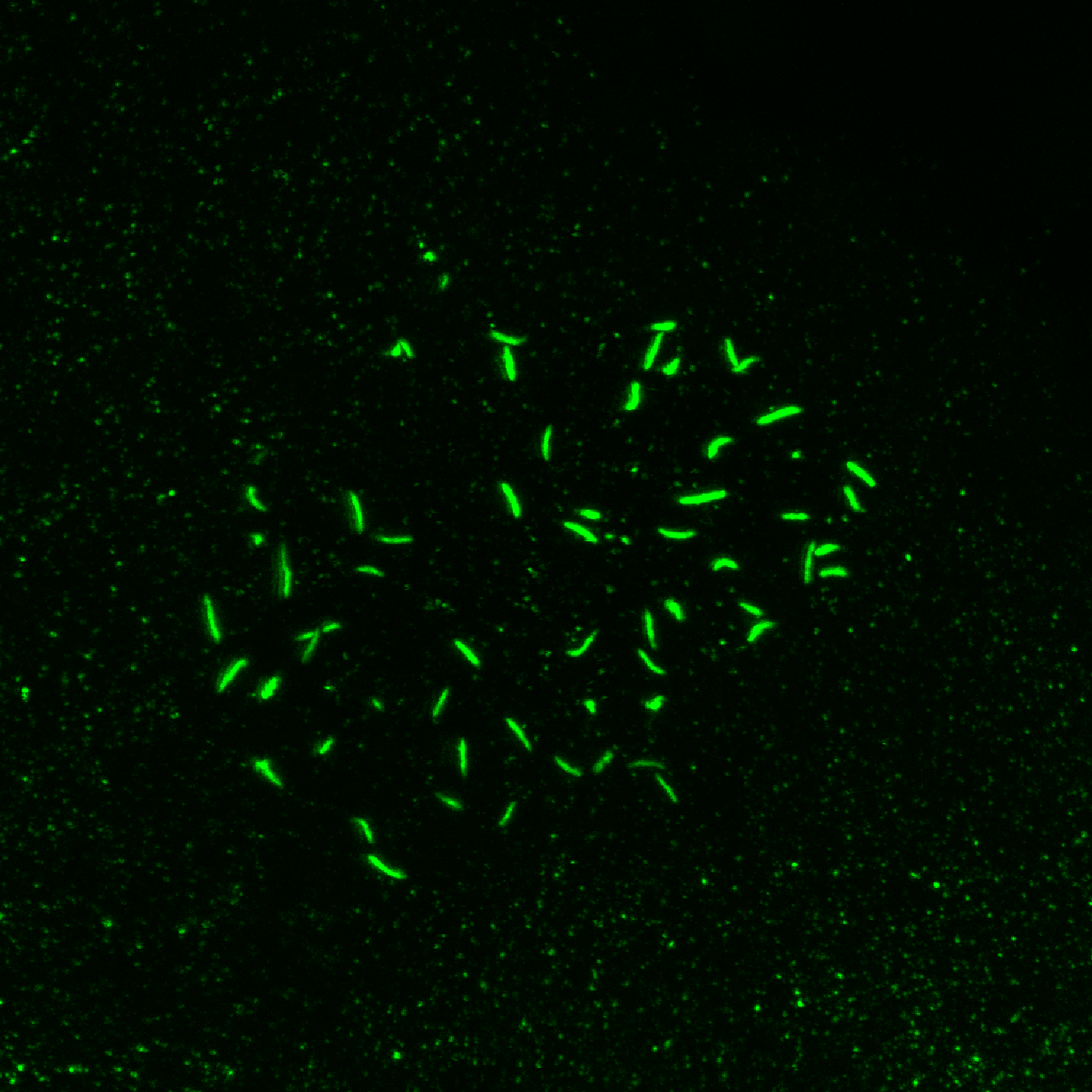

Supplement: Supplementary file 7 — Source data Fig. 6 [file 44319_2025_597_MOESM7_ESM.zip › Figure 6/6D/Ctrl MO.tif]

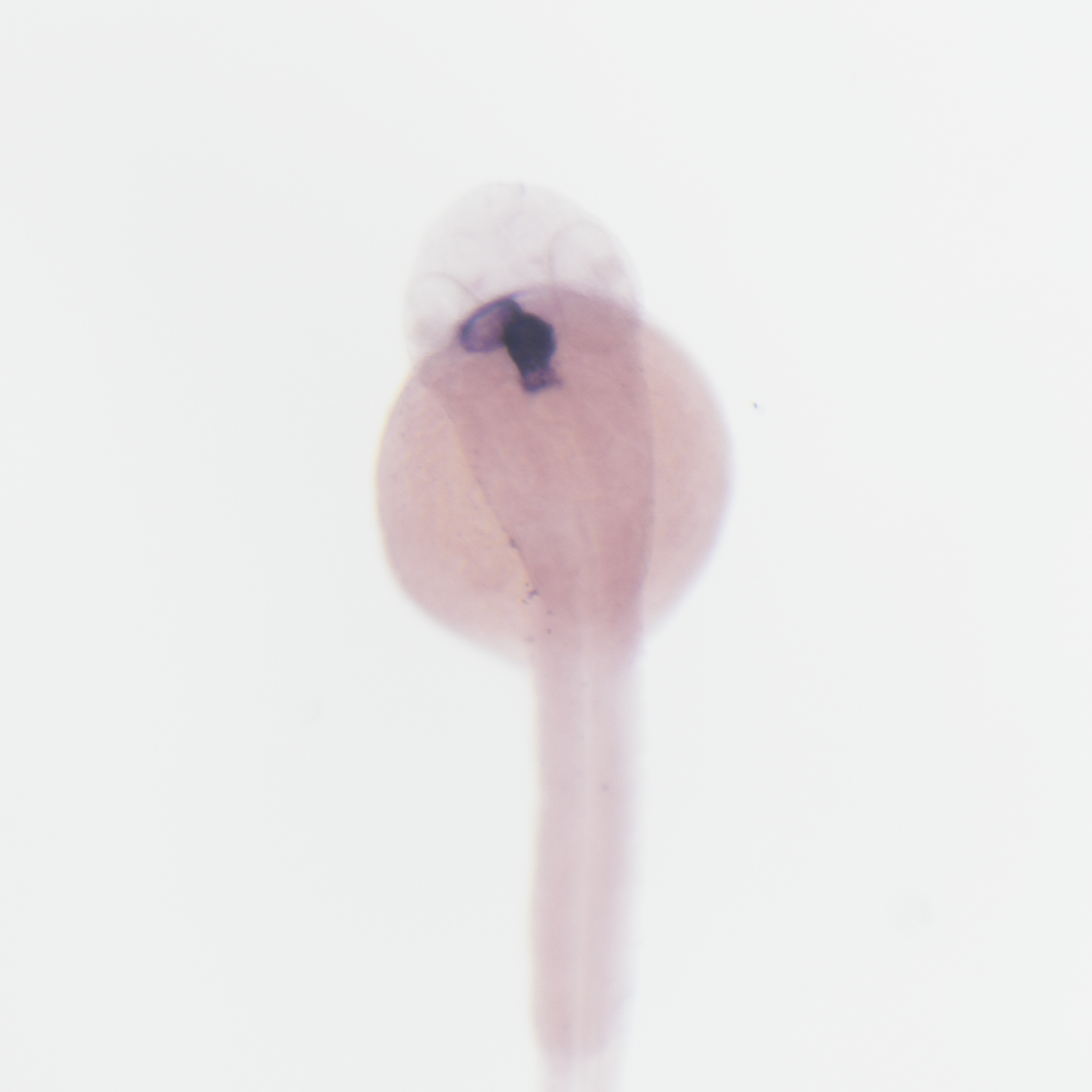

Supplement: Supplementary file 7 — Source data Fig. 6 [file 44319_2025_597_MOESM7_ESM.zip › Figure 6/6G/Left.tif]

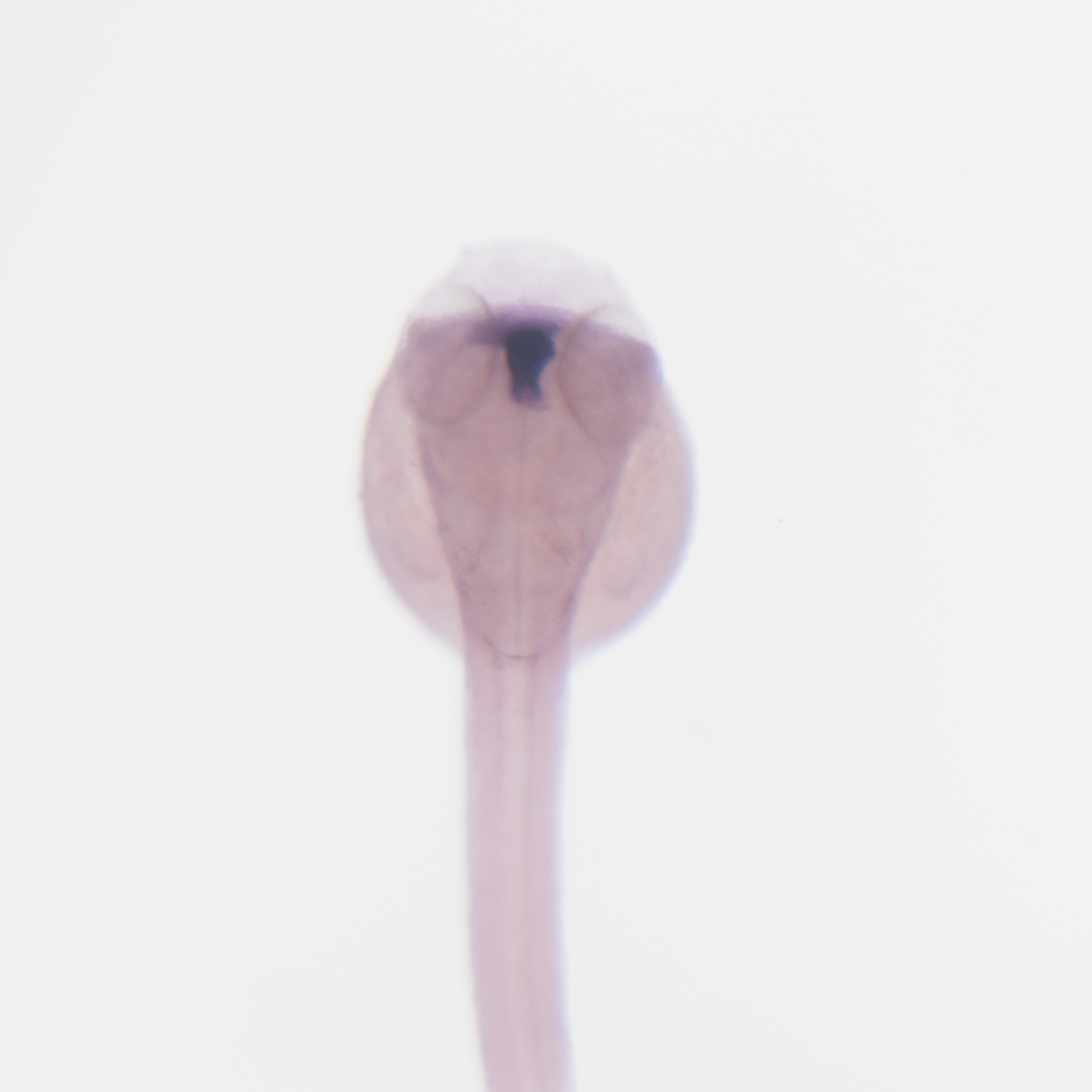

Supplement: Supplementary file 7 — Source data Fig. 6 [file 44319_2025_597_MOESM7_ESM.zip › Figure 6/6G/Middle.tif]

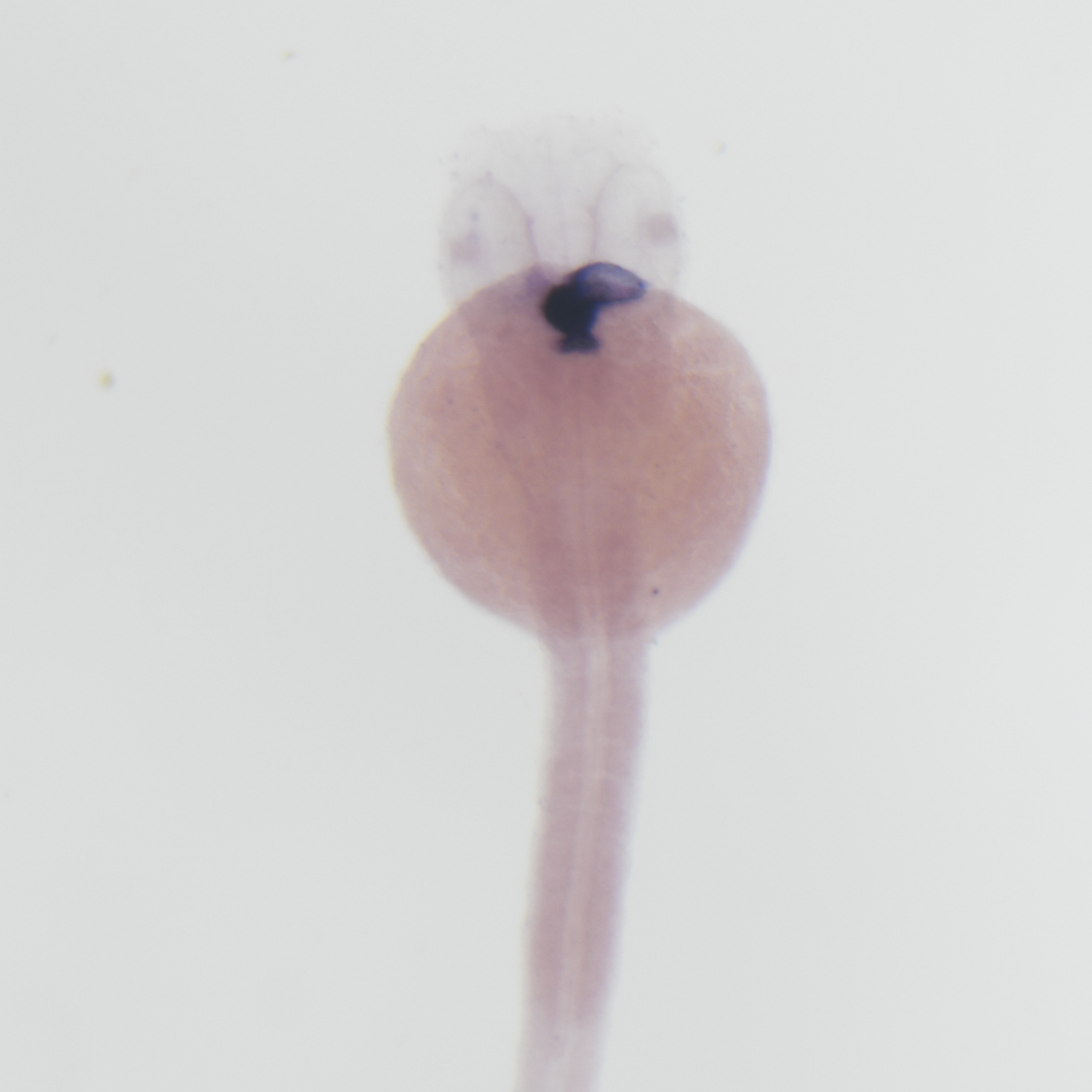

Supplement: Supplementary file 7 — Source data Fig. 6 [file 44319_2025_597_MOESM7_ESM.zip › Figure 6/6G/Right.tif]

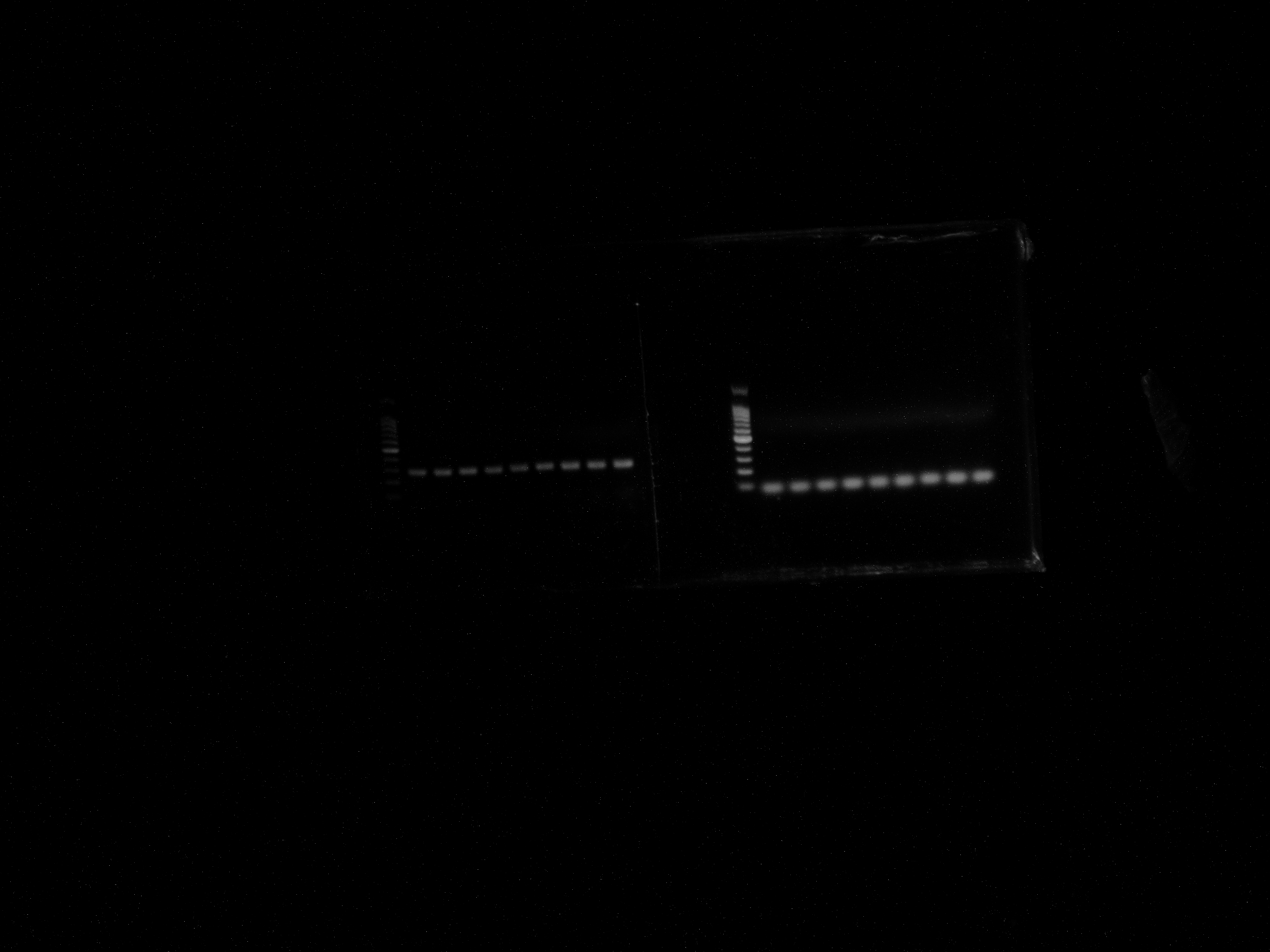

Supplement: Supplementary file 8 — Figure EV6D Source Data [file 44319_2025_597_MOESM8_ESM.bmp]
