# Supplementary material for: BICD2 promotes ciliogenesis by facilitating CP110 removal from the mother centriole
Source: EMBO Rep. 2025 Oct 16;26(22):5567–88. doi: 10.1038/s44319-025-00597-0 (PMC12635215; doi:10.1038/s44319-025-00597-0)
Supplement: Supplementary file 9 — Expanded View Figures [file 44319_2025_597_MOESM9_ESM.pdf]

Expanded View Figures

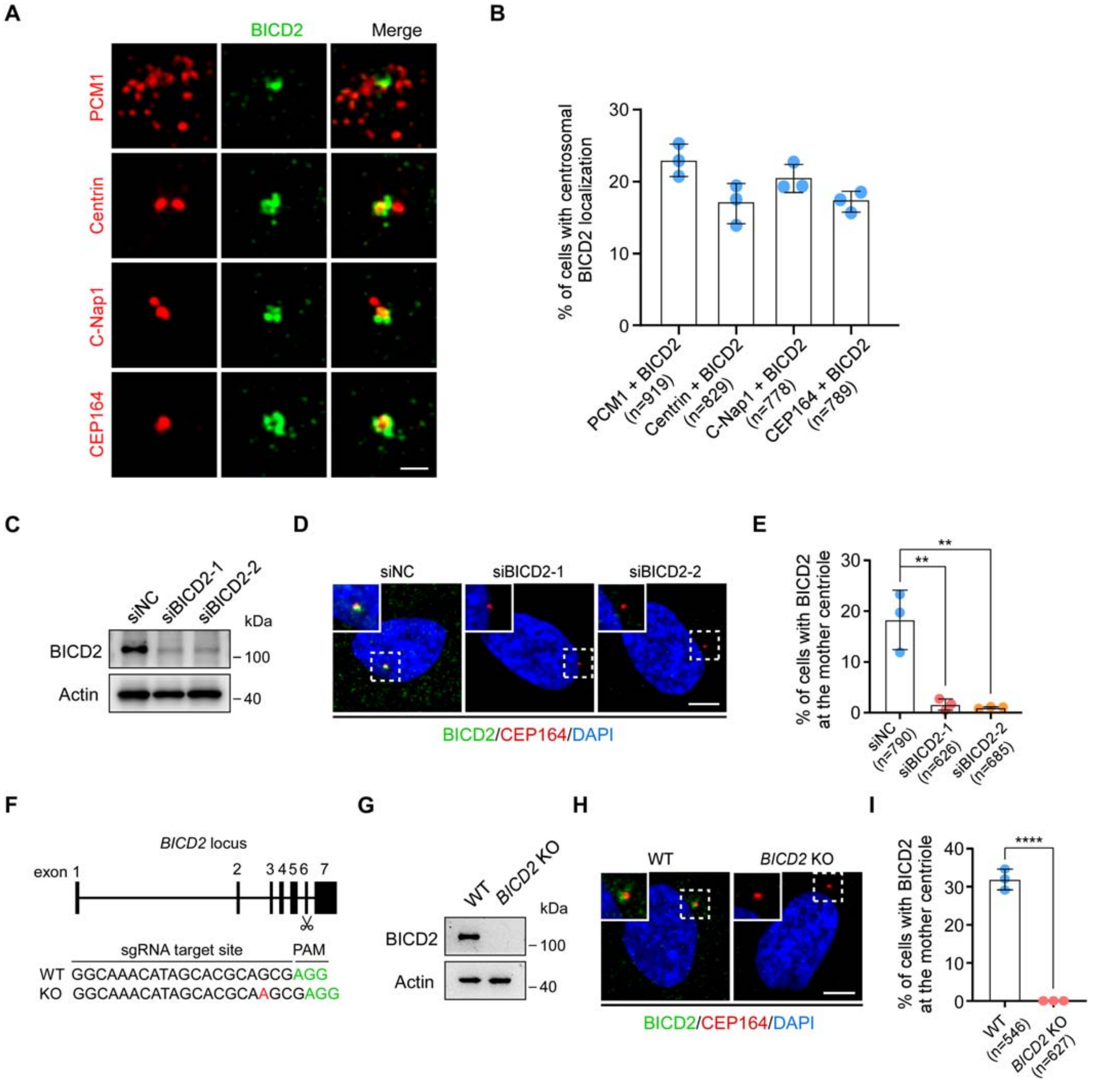

Figure EV1. Localization of BICD2 and verification of BICD2 antibody.

(**A**) Immunostaining images of RPE-1 cells stained with BICD2 antibody in conjunction with antibodies against PCM1, Centrin, C-Nap1, or CEP164. RPE-1 cells were cultured in normal serum medium. Scale bar, 1 µm. (**B**) Quantification analysis of the percentage of cells showing BICD2 localization to PCM1, Centrin, C-Nap1, or CEP164. (**C–E**) RPE-1 cells transfected with non-targeting control (NC) or *BICD2* siRNAs for 48 h were subjected to Western blotting or immunofluorescence. Western blot analysis of BICD2 protein (**C**). Confocal images of RPE-1 cells stained with anti-BICD2 and anti-CEP164 antibodies (**D**). Scale bar, 5 µm. Quantification analysis of the percentage of cells with BICD2 at the mother centriole (**E**). *P* (siNC vs. siBICD2-1) = 0.0083, *P* (siNC vs. siBICD2-2) = 0.0069. (**F**) Schematic representation of the CRISPR-Cas9 targeting site in the *BICD2* locus and the genotype of *BICD2* knockout RPE-1 cells. (**G–I**) Western blot analysis (**G**) and immunofluorescence analysis (**H**, **I**) of wild-type and *BICD2* knockout RPE-1 cells. Scale bar, 5 µm. *P* (WT vs. *BICD2* KO) < 0.0001. Actin was served as a loading control. DNA was stained by DAPI. n, the number of total cells calculated. Data were presented as mean ± SD from three independent biological repeats. Student's *t*-test; $^{**}P < 0.01$, $^{****}P < 0.0001$.

© The Author(s)

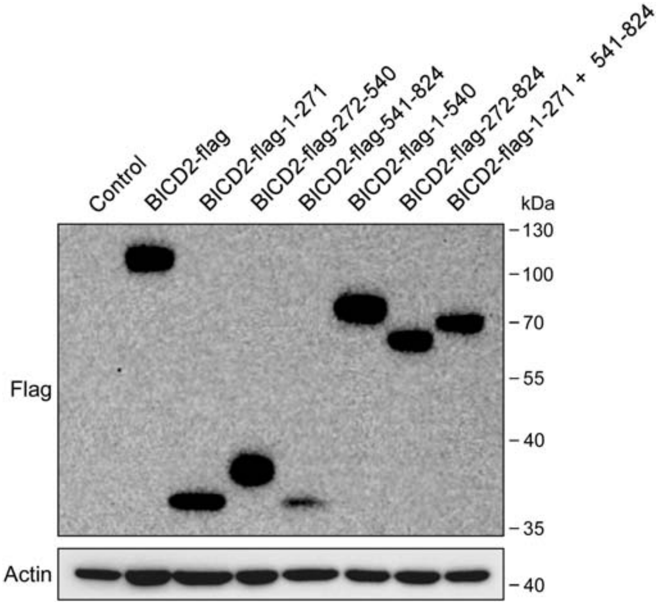

Figure EV2. The overexpression efficiency of full-length BICD2 and BICD2 truncation mutants.

Western blot analysis of the Flag protein in RPE-1 cells infected with lentiviruses carrying the indicated plasmids. Actin was used as a loading control.

© The Author(s)

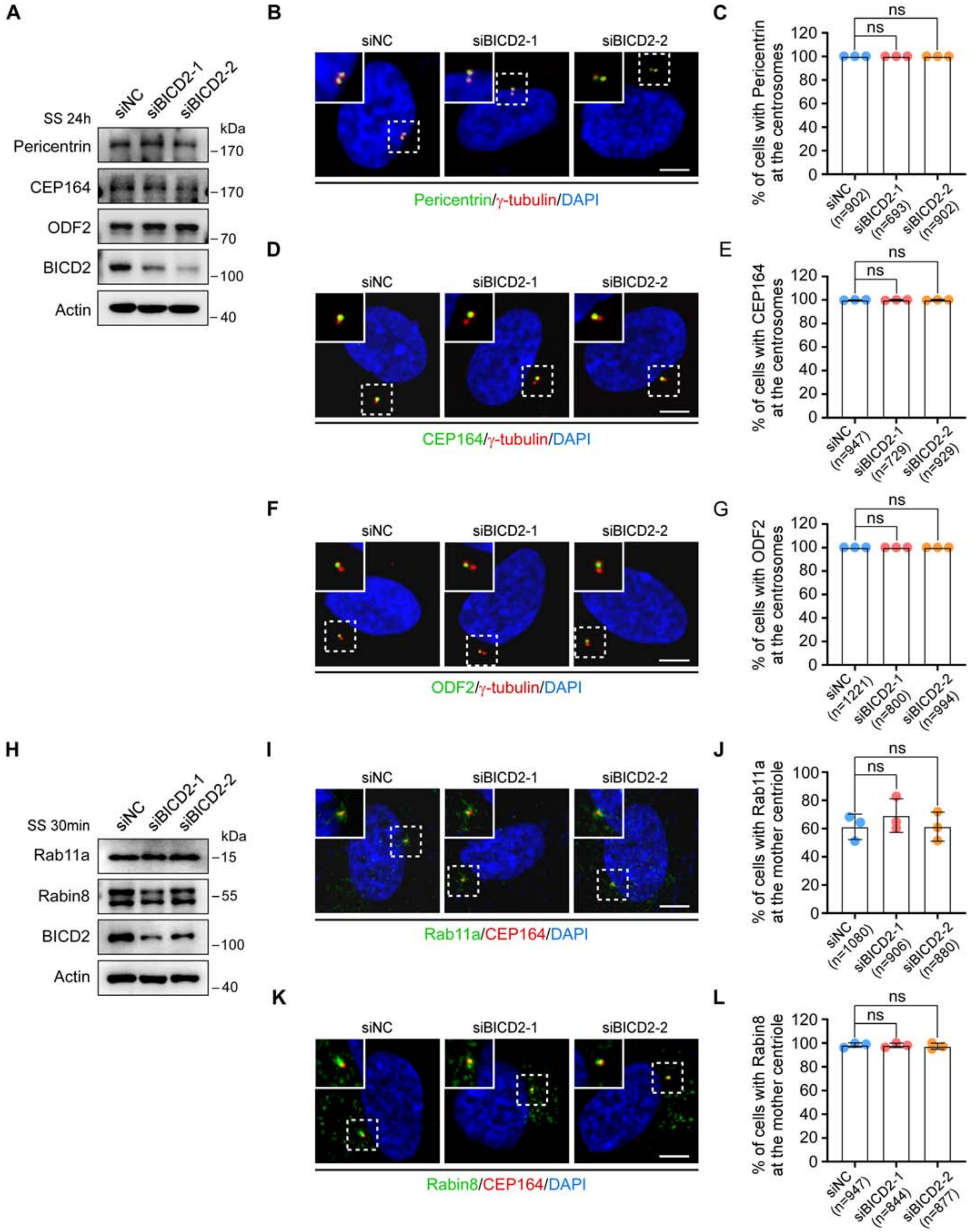
A
SS 24h
siNC
siBICD2-1
siBICD2-2
kDa
Pericentrin
CEP164
ODF2
BICD2
Actin
170
170
70
100
40
B
siNC
siBICD2-1
siBICD2-2
Pericentrin/γ-tubulin/DAPI
C
% of cells with Pericentrin at the centrosomes
ns
ns
siNC (n=902)
siBICD2-1 (n=693)
siBICD2-2 (n=902)
D
CEP164/γ-tubulin/DAPI
E
% of cells with CEP164 at the centrosomes
siNC (n=947)
siBICD2-1 (n=729)
siBICD2-2 (n=929)
F
ODF2/γ-tubulin/DAPI
G
% of cells with ODF2 at the centrosomes
siNC (n=1221)
siBICD2-1 (n=800)
siBICD2-2 (n=994)
H
SS 30min
Rab11a
Rabin8
BICD2
Actin
15
55
100
40
I
Rab11a/CEP164/DAPI
J
% of cells with Rab11a at the mother centriole
siNC (n=1080)
siBICD2-1 (n=906)
siBICD2-2 (n=880)
K
Rabin8/CEP164/DAPI
L
% of cells with Rabin8 at the mother centriole
siNC (n=947)
siBICD2-1 (n=844)
siBICD2-2 (n=877)

Figure EV3. BICD2 knockdown does not affect centrosome integrity, distal appendages assembly, or ciliary vesicle formation.

(**A–G**) RPE-1 cells transfected with control or *BICD2* siRNAs for 48 h were treated with serum starvation for an additional 24 h, and then subjected to Western blotting or immunofluorescence. (**H–L**) RPE-1 cells transfected with the indicated siRNAs for 48 h were treated with serum starvation for another 30 min, and then applied for Western blotting or immunofluorescence. Western blot analysis for the indicated proteins (**A**, **H**). Actin, a loading control. Confocal images of RPE-1 cells stained with antibodies against the indicated proteins (**B**, **D**, **F**, **I**, **K**). DNA was stained by DAPI. Scale bars, 5 µm. Quantification analyses of the percentage of cells with Pericentrin, CEP164, or ODF2 at the centrosomes (**C**, **E**, **G**), or cells with Rab11a or Rabin8 at the mother centriole (**J**, **L**). *n*, the number of total cells calculated. Data were from three independent biological repeats and presented as mean ± SD. Student's *t*-test; ns not significant.

 © The Author(s)

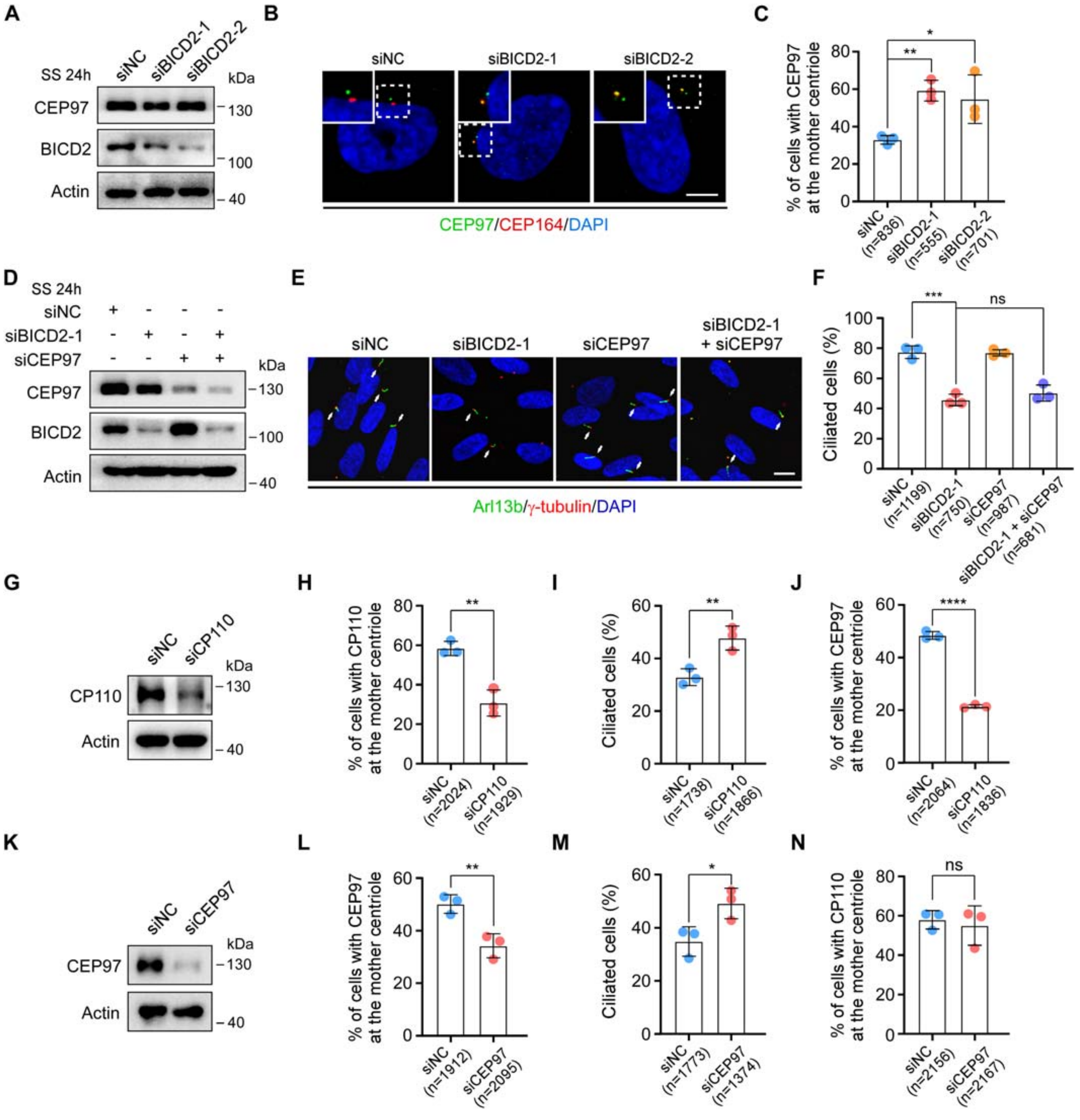

Figure EV4. CEP97 does not participate in the regulation of ciliogenesis mediated by BICD2.

(**A–F**) RPE-1 cells transfected with control or *BICD2* siRNAs for 48 h were treated with serum starvation for an additional 24 h, and then subjected to Western blotting or immunofluorescence. Western blot analysis of CEP97 and BICD2 proteins (**A**, **D**). Immunofluorescence images of RPE-1 cells stained with antibodies against CEP97 and CEP164 (**B**). Scale bar, 5 μm. Quantification analysis of the percentage of cells with CEP97 at the mother centriole (**C**). *P* (siNC vs. siBICD2-1) = 0.0015, *P* (siNC vs. siBICD2-2) = 0.0465. Confocal images of RPE-1 cells stained with anti-Arl13b and anti-γ-tubulin antibodies (**E**). Cilia are indicated by white arrows. Scale bar, 10 μm. Quantification analysis of the percentage of ciliated cells (**F**). *P* (siNC vs. siBICD2-1) = 0.0006, *P* (siBICD2-1 vs. siBICD2-1 + siCEP97) = 0.8818. (**G–N**) RPE-1 cells transfected with the indicated siRNAs for 48 h were applied for Western blotting or immunofluorescence. Immunoblotting of the indicated proteins (**G**, **K**). Quantification analyses of the percentage of cells with CP110 at the mother centriole (**H**, **N**), or ciliated cells (**I**, **M**), or cells with CEP97 at the mother centriole (**J**, **L**). CP110 at the mother centriole (**H**), *P* (siNC vs. siCP110) = 0.0031; ciliated cells (**I**, **M**), *P* (siNC vs. siCP110, siNC vs. siCEP97) = 0.0097, 0.036; CEP97 at the mother centriole (**J**, **L**), *P* ((**J**), siNC vs. siCP110) < 0.0001, *P* ((**L**), siNC vs. siCEP97) = 0.0089. Actin was used as a loading control. DNA was stained by DAPI. *n*, the number of total cells calculated. Data were presented as mean ± SD from three independent biological repeats. Student's *t*-test; ns not significant; *$P < 0.05$, **$P < 0.01$, ***$P < 0.001$, ****$P < 0.0001$.

© The Author(s)

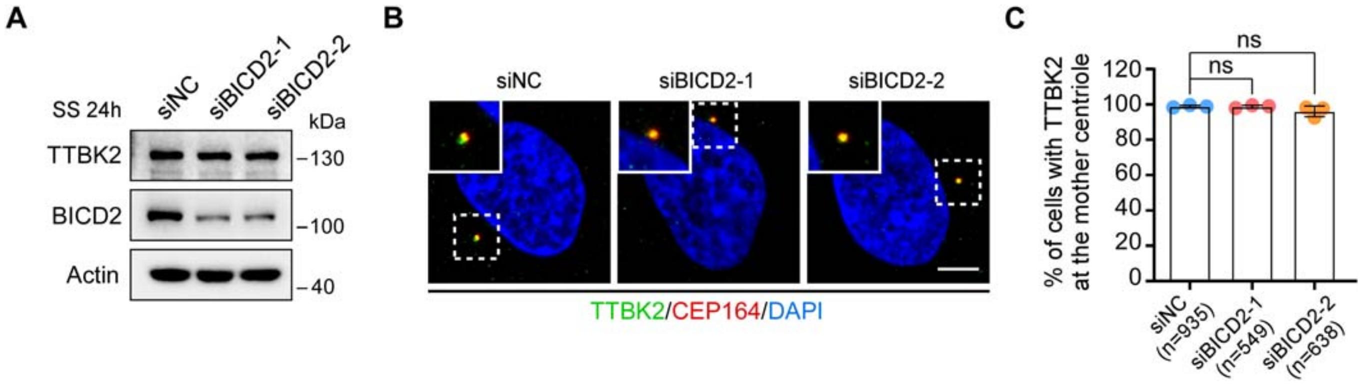

Figure EV5. Depletion of BICD2 does not affect the recruitment of TTBK2 to the mother centriole.

(**A**–**C**) RPE-1 cells transfected with control or *BICD2* siRNAs for 48 h were treated with serum starvation for an additional 24 h, and subsequently subjected to Western blotting or immunofluorescence. Western blot analysis of TTBK2 and BICD2 proteins (**A**). Actin, a loading control. Confocal images of RPE-1 cells stained with antibodies against TTBK2 and CEP164 (**B**). DNA was stained by DAPI. Scale bar, 5 µm. *n* the number of total cells calculated. Quantification analysis of the percentage of cells with TTBK2 at the mother centriole (**C**). Data were from three independent biological repeats and shown as mean ± SD. Student's *t*-test; ns not significant.

 © The Author(s)

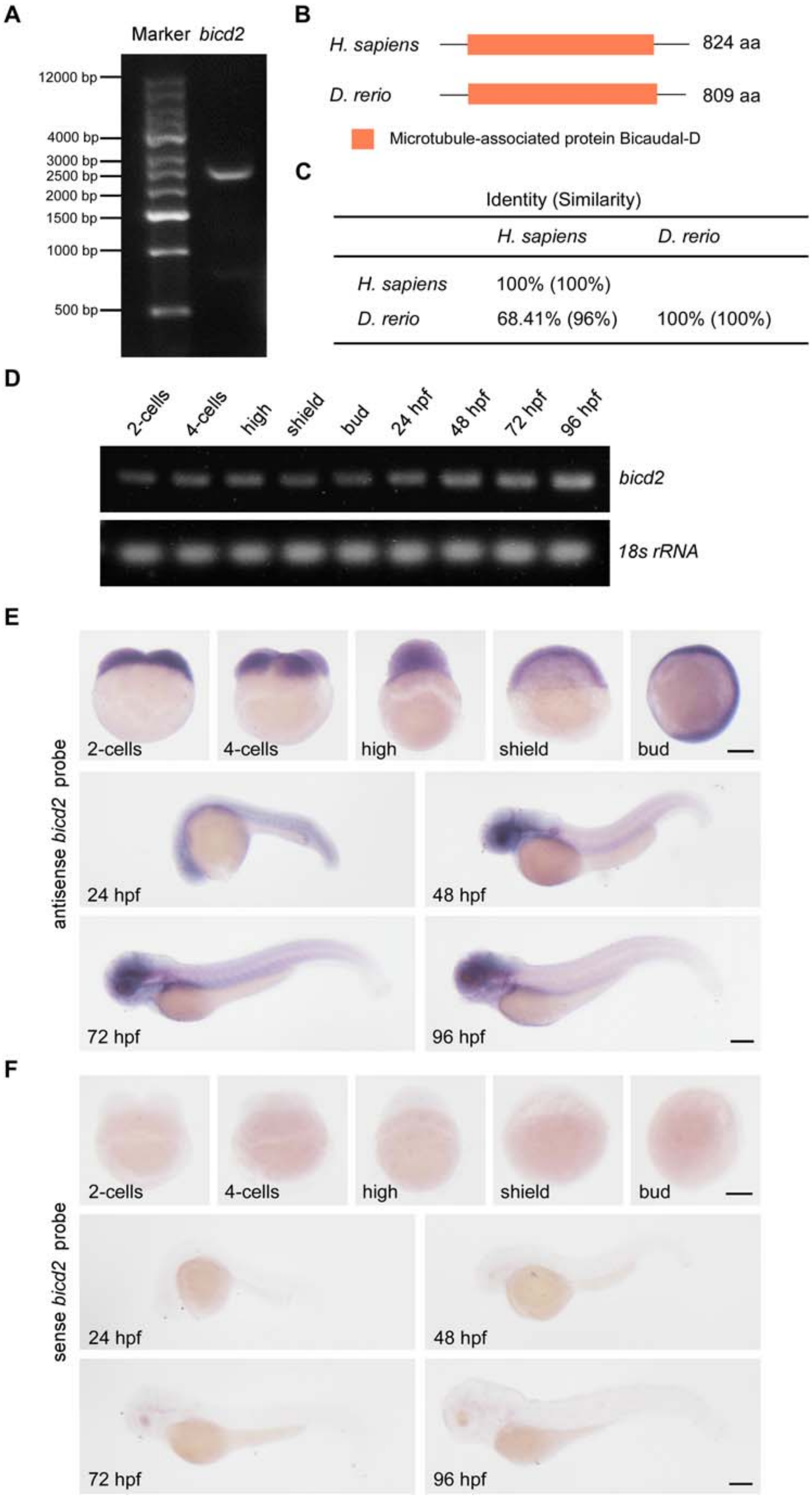
A
Marker bicd2
12000 bp
4000 bp
3000 bp
2500 bp
2000 bp
1500 bp
1000 bp
500 bp
B
H. sapiens
824 aa
D. rerio
809 aa
Microtubule-associated protein Bicaudal-D
C
Identity (Similarity)
H. sapiens
D. rerio
H. sapiens
100% (100%)
D. rerio
68.41% (96%)
100% (100%)
D
2-cells
4-cells
high
shield
bud
24 hpf
48 hpf
72 hpf
96 hpf
bicd2
18s rRNA
E
antisense bicd2 probe
2-cells
4-cells
high
shield
bud
24 hpf
48 hpf
72 hpf
96 hpf
F
sense bicd2 probe
2-cells
4-cells
high
shield
bud
24 hpf
48 hpf
72 hpf
96 hpf

Figure EV6. Molecular characterization of zebrafish *bicd2* gene.

(**A**) Cloning of zebrafish *bicd2* by reverse transcription PCR. (**B**, **C**) Schematic comparison of BICD2 amino acid sequences from the indicated species. The conserved microtubule-associated protein Bicaudal-D domains are shown in orange-filled bars. (**D**) Reverse transcription PCR analysis of *bicd2* mRNA at the different embryonic stages in zebrafish. 18s *rRNA* was used as a loading control. (**E**, **F**) Whole-mount in situ hybridization images of zebrafish embryos incubated with antisense (**E**) or sense (**F**) *bicd2* probes at the indicated developmental stages, shown in lateral view. hpf, hours post fertilization. Scale bars, 200 μm. Source data are available online for this figure.

 © The Author(s)
